# Emestrin‐Type Epidithiodiketopiperazines Inhibited Gasdermin D‐Mediated Pyroptosis via Caspase‐3/7 Activation

**DOI:** 10.1002/mco2.70548

**Published:** 2026-01-13

**Authors:** Bingchuan Geng, Shuang Lin, Wai Yen Yim, Weiguang Sun, Xiaotian Zhang, Cao Ma, Zhiwen Zhang, Quan Guo, Jie Gao, Hanxiao Zeng, Qingyi Tong, Yixuan Wang, Zhengfeng Fan, Jincheng Hou, Muwei Li, Yonghui Zhang, Zhengxi Hu

**Affiliations:** ^1^ Hubei Key Laboratory of Natural Medicinal Chemistry and Resource Evaluation, School of Pharmacy Tongji Medical College, Huazhong University of Science and Technology Wuhan China; ^2^ Fuwai Central China Cardiovascular Hospital Central China Fuwai Hospital of Zhengzhou University Zhengzhou China; ^3^ Henan Provincial Clinical Research Center For Cardiovascular Disease Zhengzhou China; ^4^ Department of Cardiovascular Surgery Union Hospital Tongji Medical College Huazhong University of Science and Technology Wuhan China; ^5^ Institute of Pharmaceutical Process Hubei Province Key Laboratory of Occupational Hazard Identification and Control School of Medicine Wuhan University of Science and Technology Wuhan China; ^6^ Hubei Shizhen Laboratory Wuhan China; ^7^ Hubei Jiangxia Laboratory Wuhan China

**Keywords:** apoptosis, gasdermin D, high‐throughput screening, pyroptosis, sepsis

## Abstract

Sepsis, a life‐threatening dysregulated host response to infection, is frequently exacerbated by pyroptosis—a programmed, proinflammatory cell death process mediated by Gasdermin D (GSDMD) activation. Using high‐throughput screening, we identified emestrin‐type epidithiodiketopiperazines (ETPs) as potent inhibitors of GSDMD cleavage during pyroptosis in Tohoku Hospital Pediatrics‐1 (THP‐1, a human acute monocytic Leukemia cell line)‐derived macrophages. Combined surface plasmon resonance and western blotting analyses demonstrated that these ETPs activate caspase‐3/7, which in turn cleaves GSDMD at aspartic acid residue 87 to generate a p10 fragment. This process prevents the formation of the pore‐forming p30 fragment, thereby mitigating its associated inflammatory effects. Building on these results, in vivo studies showed that a low dose of the lead emestrin‐type ETP (compound **2**) protected against lethal lipopolysaccharide (LPS)‐induced septic shock and attenuated lung inflammation. This protective effect was further validated in the clinically relevant cecal ligation and puncture (CLP) model, where compound **2** significantly enhanced survival by suppressing the infiltration of GSDMD‐positive neutrophils and monocytes. scRNA‐seq of murine lung tissue showed that compound **2** suppressed LPS‐induced systemic inflammation by inhibiting moDC maturation. Collectively, these findings establish the therapeutic potential of targeting GSDMD‐driven pyroptosis with ETPs in sepsis and suggest their promise for clinical translation.

## Graphical abstract

This study demonstrates that emestrin‐type ETPs suppress GSDMD‐mediated pyroptosis through caspase‐3/7 activation, thereby attenuating inflammatory responses and conferring protection against sepsis. In THP‐1 macrophages, ETPs significantly reduced pyroptosis and secretion of the proinflammatory cytokines IL‐1β and IL‐18. In mouse models, compound **2** (emestrin) alleviated acute lung injury, and inhibited systemic inflammation by restraining the maturation of monocyte‐derived dendritic cells.

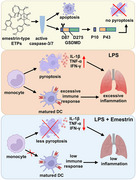

## 1 Introduction

Sepsis is a life‐threatening condition arising from a dysregulated host response to infection [1]. A 2020 study based on 2017 data estimated approximately 49 million global sepsis cases and 11 million sepsis‐related deaths, accounting for nearly 20% of all global mortality [1, 2]. Sepsis triggers a systemic inflammatory response, characterized by excessive proinflammatory cytokine release and often culminating in rapid acute organ dysfunction [3, 4]. Despite advancements in understanding the pathogenesis of sepsis and the development of modern therapeutic strategies, effective targeted treatments remain an urgent and unmet medical need.

In the early phase of sepsis, the dysregulated host immune response induces programmed cell death in immune cells, including T lymphocytes and macrophages; apoptosis and pyroptosis play pivotal, yet opposing, roles in this process [4, 5, 6]. Pyroptosis, a lytic and inflammatory form of programmed cell death mediated by caspase‐1/11‐dependent cleavage of Gasdermin D (GSDMD) at residue D275, serves as an initial defense against pathogens [7]. However, when excessive, it exacerbates tissue damage, potentially culminating in septic shock and multiorgan failure [7, 8, 9]. In stark contrast, apoptosis is a noninflammatory, caspase‐3/7/8/9‐dependent process that preserves plasma membrane integrity and facilitates the release of anti‐inflammatory metabolites. However, this pathway can also promote immunosuppression by depleting essential immune cells [10, 11, 12, 13]. The delicate equilibrium between pyroptosis‐driven inflammation and apoptosis‐induced immunosuppression is critically disrupted in sepsis, and dysregulation of either pathway can adversely affect patient outcomes [6].

GSDMD‐mediated pyroptosis is now recognized as a central driver of septic pathology [14, 15]. Genetic [16, 17] or pharmacological inhibition of GSDMD (e.g., by disulfiram, necrosulfonamide, dimethyl fumarate, or NU6300) [18, 19, 20, 21] reduces pyroptosis and improves survival in sepsis models, validating its therapeutic potential. However, existing inhibitors directly target GSDMD cysteine residues, leaving unexplored the strategy of modulating pyroptosis‐apoptosis balance via upstream caspase regulation—a gap our study addresses by discovering caspase‐3/7‐activating emestrin‐type ETPs that divert cells from pyroptotic to apoptotic death.

Natural products derived from plants, microorganisms, and marine organisms have long been invaluable sources of therapeutic agents, as exemplified by aspirin and penicillin [22, 23]. Despite challenges associated with their isolation and production, natural products continue to play a pivotal role in high‐throughput drug discovery platforms. Capitalizing on this strategy, we developed a 96‐well high‐throughput screening (HTS) system to systematically screen 536 compounds from an in‐house natural product library—curated from fungi and medicinal plants—for inhibitors of GSDMD‐mediated pyroptosis. This screening campaign led to the identification of emestrin‐type ETPs from the arthropod‐associated fungus *Aspergillus* sp. TJ403‐G07. These compounds feature a distinctive transannular disulfide bridge‐modified cyclodipeptide scaffold and demonstrate potent inhibitory activity against pyroptotic cell death.

Following this initial discovery, we comprehensively validated the antipyroptotic efficacy of emestrin‐type ETPs in THP‐1‐derived macrophages. Using western blotting and surface plasmon resonance (SPR) analyses, we identified caspase‐3 and ‐7 as the direct molecular targets of these compounds. In murine models, a low dose (2 mg/kg) of compound **2** (namely emestrin) significantly improved survival rates in both lipopolysaccharide (LPS)‐induced septic shock and the clinically relevant cecal ligation and puncture (CLP) model, demonstrating robust efficacy against lethal sepsis. Single‐cell RNA sequencing (scRNA‐seq) of murine lung tissue indicated that compound **2** treatment led to an expansion of a monocyte population characterized by reduced GSDMD expression and attenuated pyroptosis in a model of LPS‐induced acute lung injury (ALI). Furthermore, trajectory analysis suggested that compound **2** treatment impaired the antigen‐presenting capacity of monocytes and suppressed their maturation into dendritic cells (DCs). Collectively, our findings identify emestrin‐type ETPs as a novel class of pyroptosis inhibitors that function by directly targeting caspase‐3/7, thereby demonstrating their potential as a therapeutic intervention for sepsis.

## 2 Results

### 2.1 High‐Throughput Compound Screening for Pyroptosis Inhibition in THP‐1 Differentiated Macrophages (HitID)

**FIGURE 1:**
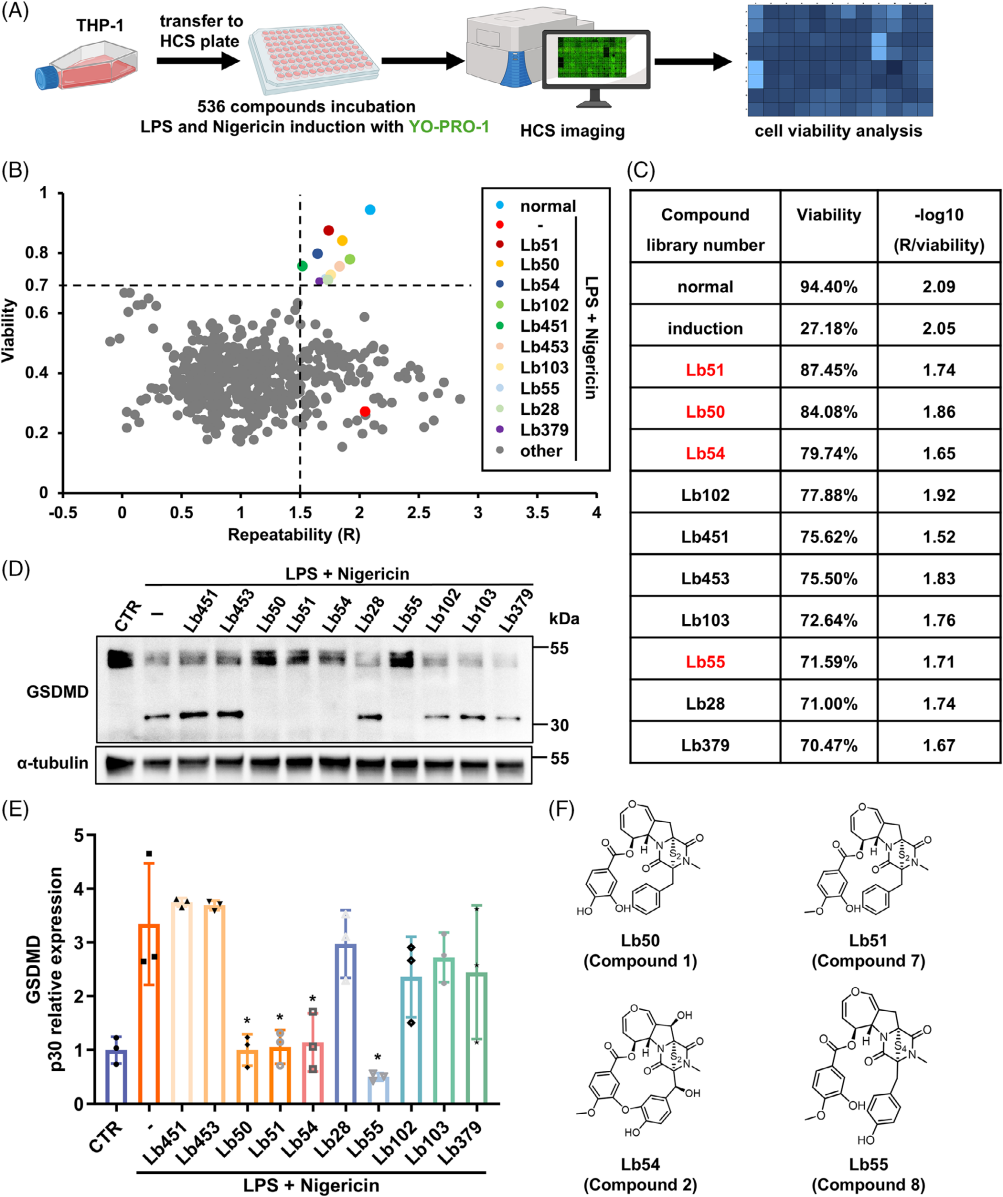
Identification of compounds that inhibited pyroptosis in THP‐1 differentiated macrophages. (A) Workflow diagram for high‐content screening of 536 compound library. (B and C) Cell viability after compound treatment (1 µM) measured by YO‐PRO‐1 (green) imaging in THP‐1 differentiated macrophages. Repeatability (*R*) is defined as the absolute difference in viability between two independent experiments. The repeatability score −log_10_ (*R*/viability) quantifies experimental consistency, where higher values indicate superior repeatability. The dashed line (−log_10_ (*R*/viability) = 1.5) marks the threshold for high repeatability (<3.2% relative variability). Viability was calculated as the percentage of YO‐PRO‐1^−^ cells. (D and E) Western blotting analysis of GSDMD and α‐tubulin in the THP‐1 differentiated macrophages treated with each compound (1 µM) 4 h after pyroptosis induction. (F) The chemical structures of Lb51 (compound **7**), Lb50 (compound **1**), Lb54 (compound **2**), and Lb55 (compound **8**).

Utilizing scalable and homogeneous THP‐1 differentiated macrophages, we developed a 96‐well format high‐content screening (HCS) assay to identify potential pyroptosis inhibitors from our in‐house natural products library (Figure 1A). Pyroptotic cell death was induced by LPS and nigericin treatment as previously described [17]. Membrane‐impermeable YO‐PRO‐1 dye (green) was used to quantify pyroptotic cells, as it selectively enters cells through plasma membrane pores formed by oligomerized, cleaved GSDMD [24].

In total, 536 compounds were screened across 12 plates in two independent experiments (Figure S1A). Each plate included positive (pyroptosis induction without compounds) and negative (no induction or compounds) controls. Cell viability in each well was determined by dividing the number of YO‐PRO‐1‐negative (live) cells by the total cell number. The average cell viability in the positive and negative control wells was 94.40 and 27.18%, respectively (Figure 1B, C). Repeatability of the experiments was calculated using –log_10_ (*R*/viability). From this primary screen, 10 compounds were identified that increased cell viability up to 70% with high repeatability (Figure 1B, C).

**FIGURE 2:**
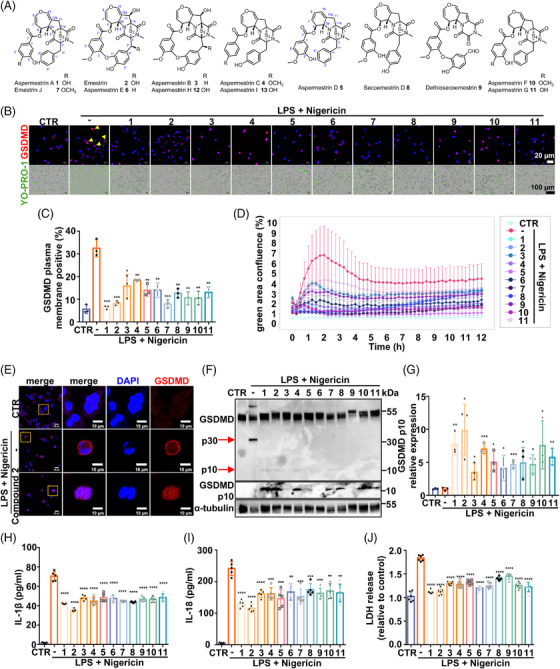
Compounds **1**–**11** inhibited pyroptosis in THP‐1 differentiated macrophages. (A) The plane structures of compounds **1**–**13**. (B) Immunofluorescence imaging of GSDMD (red) and YO‐PRO‐1 (green) in the THP‐1 differentiated macrophages treated with each compound (1 µM) 4 h after pyroptosis induction with LPS and nigericin. Yellow arrows showed GSDMD plasma membrane positive cells. (C) GSDMD plasma membrane positive analysis of immunofluorescence staining (mean ± SD, *n* = 3 per group). (D) YO‐PRO‐1 (green) area confluence analysis over 12 h live cell imaging (mean ± SD, *n* = 3 per group). (E) High‐magnification images of GSDMD immunofluorescence staining in the THP‐1 differentiated macrophages treated with indicated conditions. (F and G) Western blotting analysis of GSDMD and α‐tubulin in the THP‐1 differentiated macrophages treated with each compound (1 µM) 4 h after induction with LPS and nigericin. (H and I) IL‐1β and IL‐18 secretion in media supernatants measured by Elisa 4 h postpyroptosis induction (mean ± SD, *n* = 5 per group). (J) LDH release 4 h postpyroptosis induction (mean ± SD, *n* = 6 per group). For C, G, H, I, and J, data were analyzed by *t*‐test against pyroptosis induction only group (LPS + nigericin), *p < 0.05, **p < 0.01, ***p < 0.001, ****p < 0.0001.

The top 10 candidates were subsequently validated in a secondary screening using a six‐well plate format, which also allowed western blotting analysis. THP‐1 differentiated macrophages were induced for pyroptosis and treated with each compound. Western blotting revealed that four of these ten compounds markedly reduced GSDMD cleavage upon LPS/nigericin stimulation (Figure 1D, E). Detailed HCS data for these four compounds are presented in Figure S1B. Notably, these four hits (Lb51, Lb50, Lb54, and Lb55) share highly similar chemical scaffolds, belonging to emestrin‐type ETPs isolated from *Aspergillus* sp. TJ403‐G07 (Figure 1F). As a Hit expansion strategy, secondary metabolites of *Aspergillus* sp. TJ403‐G07 were further investigated, leading to the isolation of nine additional derivatives (Figures 2A and S2). In this study, these thirteen compounds are collectively referred to as compounds **1–13**.

### 2.2 Structural Elucidation of Thirteen Emestrin‐Type ETPs Isolated from *Aspergillus* sp. TJ403‐G07

The fungal strain *Aspergillus* sp. TJ403‐G07 was isolated from a grasshopper collected in Wuhan City, Hubei Province, P. R. China. After fermentation on rice solid medium, the culture was extracted with ethanol (EtOH). The crude extract was subsequently subjected to silica gel, octadecylsilane, Sephadex LH‐20, and preparative/semi‐preparative high‐performance liquid chromatography, which led to the isolation of nine new emestrin‐type ETPs, named aspermestrins A–I (**1**, **3–6**, and **10–13**), along with four known congeners (**2** and **7–9**) (Figure 2A). Notably, compounds **3** and **12** represent the first examples of emestrin‐type ETPs featuring a *β*‐oriented sulfur bridge. The structures of these nine new compounds were unequivocally established through extensive spectroscopic analysis, including mainly NMR spectra (Figures S2 and S3 and Tables S1 and S2), quantum chemical NMR calculations (Figures S6 and S7), ECD comparisons and calculations (Figure S5 and Tables S3 and S4), and single‐crystal X‐ray diffraction analyses (Figure S4). Detailed descriptions of the structural elucidation of compounds **1–13** are provided in the Supporting Information.

The four compounds (Lb51, Lb50, Lb54, and Lb55) identified in the HCS assay correspond to compounds **7**, **1**, **2**, and **8**, respectively (Figure 1F). Due to the purification challenges associated with compounds **12** and **13**, compounds **1–11** were selected to further investigate their protective effects during the induction of pyroptosis.

### 2.3 The Eleven Lead Compounds Inhibited Pyroptosis in THP‐1 Differentiated Macrophages

Given the structural similarities among the eleven lead compounds, we hypothesized that they would exert comparable protective effects during pyroptosis induction. Immunofluorescent staining confirmed a marked reduction in GSDMD‐positive pores in the plasma membrane after compound treatment (Figure 2B,C). High‐magnification imaging of GSDMD staining revealed the translocation of the GSDMD signal into the nucleus following treatment with compound **2** (Figure 2E), suggesting the possibility of atypical cleavage of GSDMD at other amino acid sites.

To investigate the dynamics of their protective effects, live‐cell imaging was performed to monitor the ratio of YO‐PRO‐1‐positive cells to total cells after nigericin stimulation. In the induction group, YO‐PRO‐1‐positive cells increased dramatically within 3 h and remained elevated for 12 h. In contrast, treatment with these eleven compounds significantly reduced cell death within 12 h of pyroptosis induction (Figure 2D). YO‐PRO‐1 staining at 4 h primarily reflects pyroptosis inhibition, while delayed signal accumulation (>8 h) indicates compound‐induced apoptosis (see real‐time kinetics in Figure 2E). This temporal resolution confirms that compounds **1–11** inhibit early pyroptosis while promoting late apoptosis, fully consistent with the YO‐PRO‐1 data.

Western blotting analysis further revealed that all eleven compounds reduced the p30 cleavage of GSDMD during pyroptosis induction in THP‐1 differentiated macrophages (Figure 2F). Interestingly, instead of the expected p30 N‐terminal fragments of GSDMD, a relatively weak p10 band was detected in compound‐treated cells (Figure 2F,G), indicating an alternative cleavage event that did not trigger pyroptosis. Consistently, treatment with these compounds reduced IL‐1β and IL‐18 secretion as well as dehydrogenase (LDH) release into the culture medium (Figure 2H–J).

**FIGURE 3:**
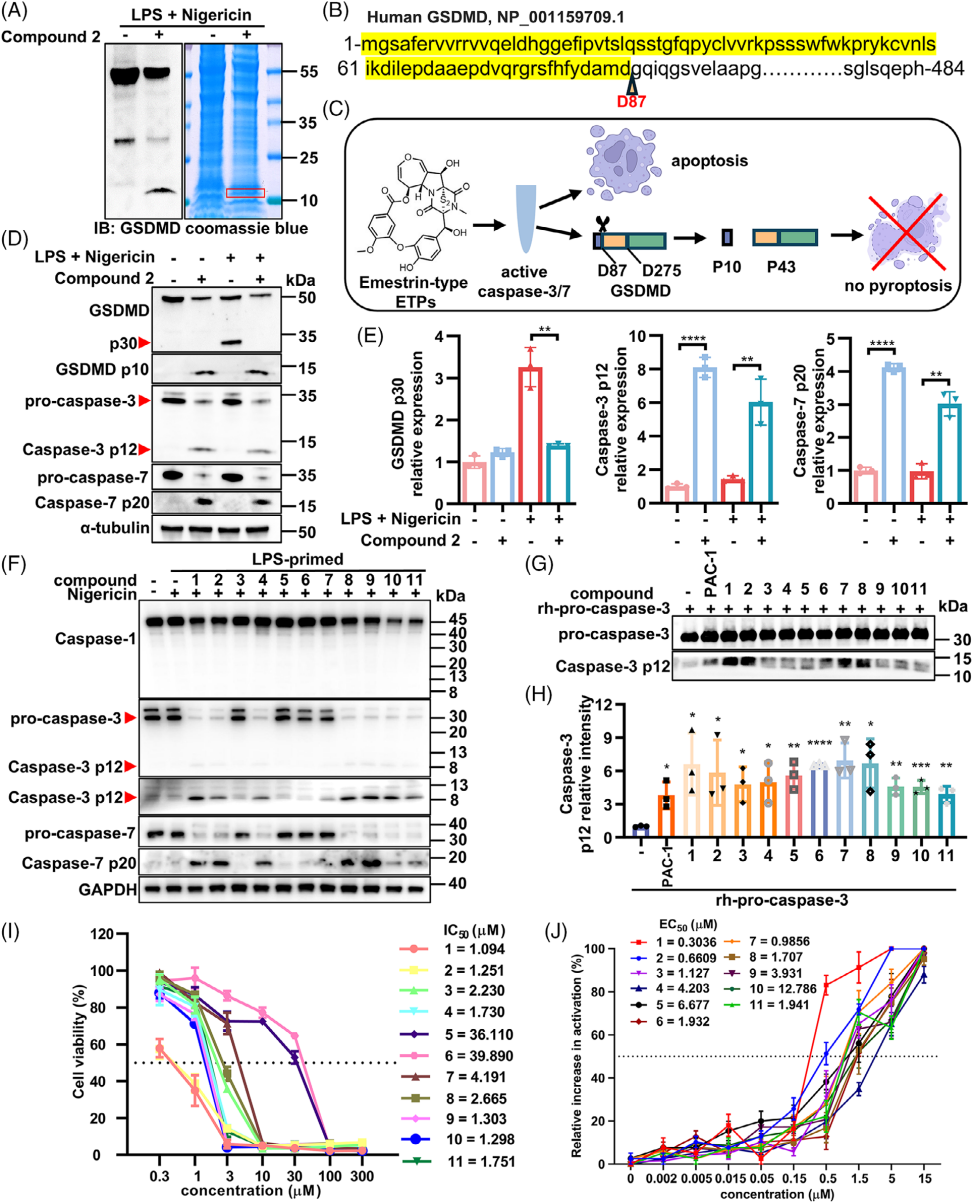
Emestrin‐type ETPs inhibited pyroptosis in THP‐1 differentiated macrophages through activation of procaspase‐3/7. (A) Immunoblotting and Coomassie blue staining of cell lysates from THP‐1 differentiated macrophages treated with or without compound **2** (1 µM) 4 h post‐LPS and nigericin induction. P10 band (red rectangle) was sent for mass spectrum analysis. (B) N‑terminal sequence analysis result of p10 band. (C) Mechanism hypothesis of compounds **1**–**11** inhibiting pyroptosis. (D and E) Western blotting analyses of GSDMD, caspase‐3, caspase‐7 cleavage, and α‐tubulin in THP‐1 macrophages treated compound **2** (1 µM) prior and after 4 h pyroptosis induction with LPS and nigericin (mean ± SD; *n* = 3 per group, *t*‐test, **p < 0.01, ****p < 0.0001). (F) Western blotting analyses of caspase‐1, caspase‐3, caspase‐7 cleavage, and α‐tubulin in the THP‐1 differentiated macrophages treated with each compound (1 µM) 4 h after pyroptosis induction with LPS and nigericin. (G and H) Western blotting analyses of recombinant human procaspase‐3 protein 12 h postincubation with PAC‐1 and each compound (5 µM) respectively at 37 ℃. (I) The IC_50_ result of these eleven compounds (*n* = 3 per group). (J) In vitro activation of recombinant human procaspase‐3 protein by eleven compounds respectively (*n* = 3 per group) and corresponding EC_50_ values.

To characterize the specific sequence of the p10 fragment, protein bands were visualized by Coomassie blue staining, excised, and subjected to mass spectrometry (Figure 3A). LC–MS/MS analysis confirmed that the p10 fragment corresponded to GSDMD residues 1–87 (Figure 3B). Given that GSDMD can undergo alternative cleavage during apoptosis [25, 26], we hypothesized that these compounds might promote apoptosis through the activation of caspase‐3 and caspase‐7 in THP‐1 differentiated macrophages (Figure 3C). Western blotting results supported this hypothesis, showing that compound **2** activated procaspase‐3/7 while suppressing p30 fragment generation of GSDMD during early pyroptosis induction (Figure 3D,E). To elucidate the specificity of these compounds in modulating pyroptosis pathways, we assessed their impact on caspase‐1 activation. These compounds demonstrated no significant effect on caspase‐1 activation, as evidenced by unaltered expression and cleavage patterns in western blotting analysis (Figure 3F).

### 2.4 Emestrin‐Type ETPs Inhibited Pyroptosis in THP‐1 Differentiated Macrophages Through Activation of Procaspase‐3/7

We next evaluated the effects of emestrin‐type ETPs on procaspase‐3/7 activation. Western blotting revealed that all eleven compounds induced activation of both caspase‐3 and caspase‐7 (Figure 3F). To directly assess their ability to catalyze the maturation of procaspase‐3 to active caspase‐3, the recombinant human procaspase‐3 protein with 5 µM of various compounds was incubated at 37°C, with PAC‐1 serving as a positive control [27]. All test compounds activated procaspase‐3 (Figure 3G,H), among which compounds **1**, **2**, **7**, and **8** showed stronger catalytic activity than PAC‐1 at the same concentration. The half‐maximal inhibitory concentration (IC_50_) values of the eleven compounds ranged from 1.094 to 39.890 µM (Figure 3I), whereas the half‐maximal activation concentration (EC_50_) values for procaspase‐3 ranged from 0.3036 to 12.786 µM (Figure 3J).

**FIGURE 4:**
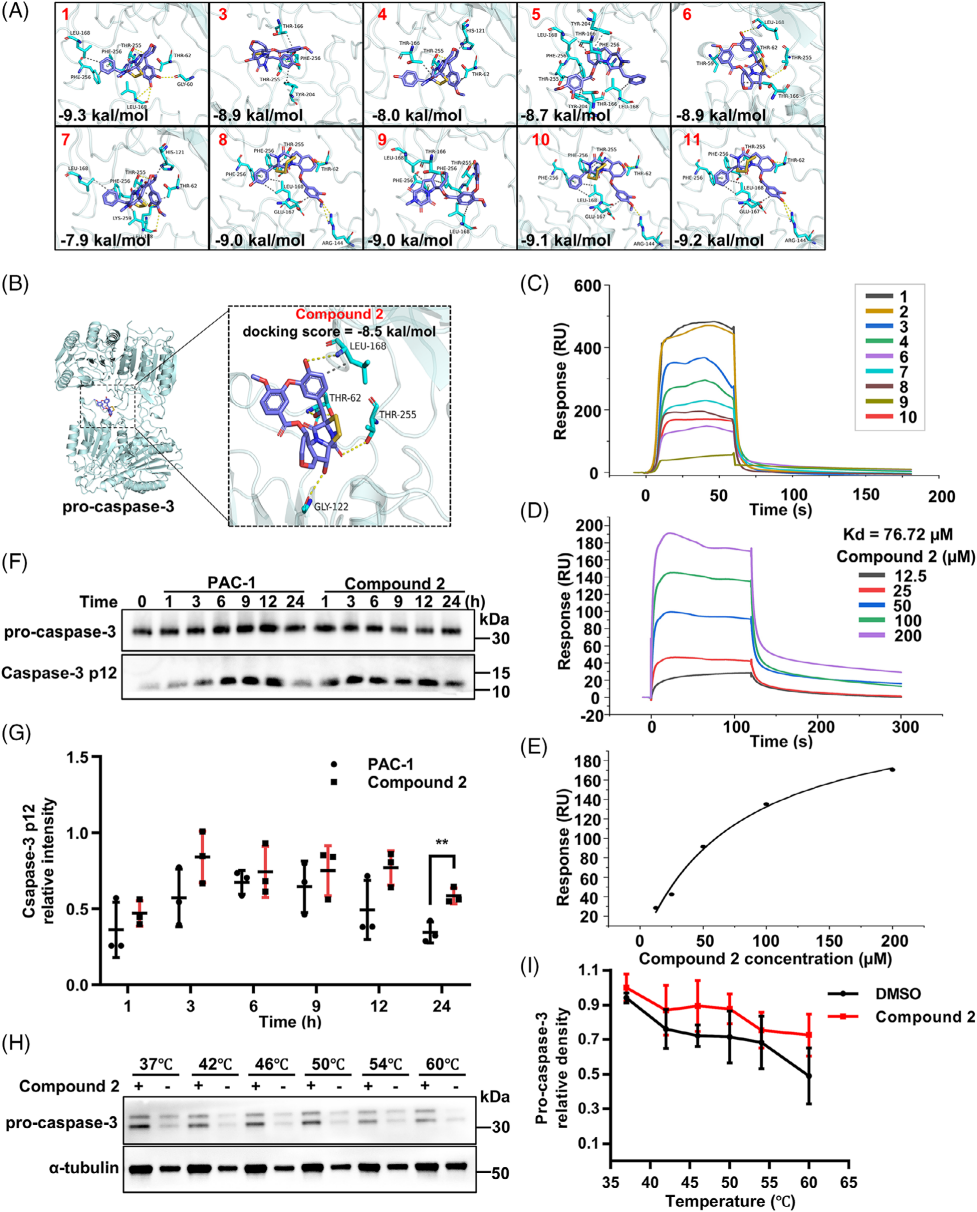
Emestrin‐type ETPs directly bound to human procaspase‐3 protein. (A) Molecular docking analyses of compounds **1** and **3**–**11** with human procaspase‐3 protein (PDB ID: 3DEK). (B) Molecular docking analysis of compound **2** with human procaspase‐3 protein. (C) Compound binding activity to recombinant human procaspase‐3 protein measured by SPR. (D and E) SPR test of compound **2** interacting with recombinant human procaspase‐3 protein. The *K*_d_ value is 76.72 µM. (F and G) Western blotting analyses of recombinant human procaspase‐3 protein different time points after incubation with PAC‐1 (5 µM) and compound **2** (5 µM) (mean ± SD; *n* = 3 per group, *t*‐test, **p < 0.01). (H and I) CETSA results for compound **2** (5 µM) in THP‐1 differentiated macrophage lysates.

To investigate binding interactions with procaspase‐3, molecular docking was performed using AutoDock Vina software with the crystal structure of human procaspase‐3 (PDB ID: 3DEK). All eleven compounds achieved docking scores below –6 (Figure 4A). High‐resolution docking analysis of compound **2** with caspase‐3 identified four potential interaction residues: Leu‐168, Thr‐62, Thr‐255, and Gly‐122 (Figure 4B). SPR experiments further confirmed that compounds **1** and **2** exhibited stronger binding affinity to recombinant human procaspase‐3 protein than other compounds; insufficient amounts of compounds **5** and **11** precluded their testing (Figure 4C). Based on both potency and purification yield, compound **2** (emestrin) was selected as the lead candidate for further investigation. Western blotting showed that both PAC‐1 and compound **2** induced the cleavage of recombinant human procaspase‐3 protein over time (Figure 4F,G). The dissociation constant (*K*_d_) value of compound **2** was determined to be 76.72 µM (Figure 4D,E). Cellular thermal shift assay (CETSA) further demonstrated that compound **2** (5 µM) markedly increased the thermal stability of procaspase‐3 protein in THP‐1 differentiated macrophage lysates as the temperature increased (Figure 4H,I). To validate the binding sites of compound **2** and procaspase‐3 protein, mutagenesis studies were performed. A single mutation of His‐121 and Gly‐122 in procaspase‐3, which may be involved in the interaction with compound **2**, was investigated. Overexpression of mutant caspase‐3 (H121A and G122A) in HEK293T cells significantly reduced the potency of compound **2** in activating mutant procaspase‐3 (Figure S8A). Notably, these two amino acids are evolutionarily conserved (Figure S8B).

### 2.5 In Vivo Efficacy of Compound 2 on LPS‐Induced Sepsis Mouse Model

**FIGURE 5:**
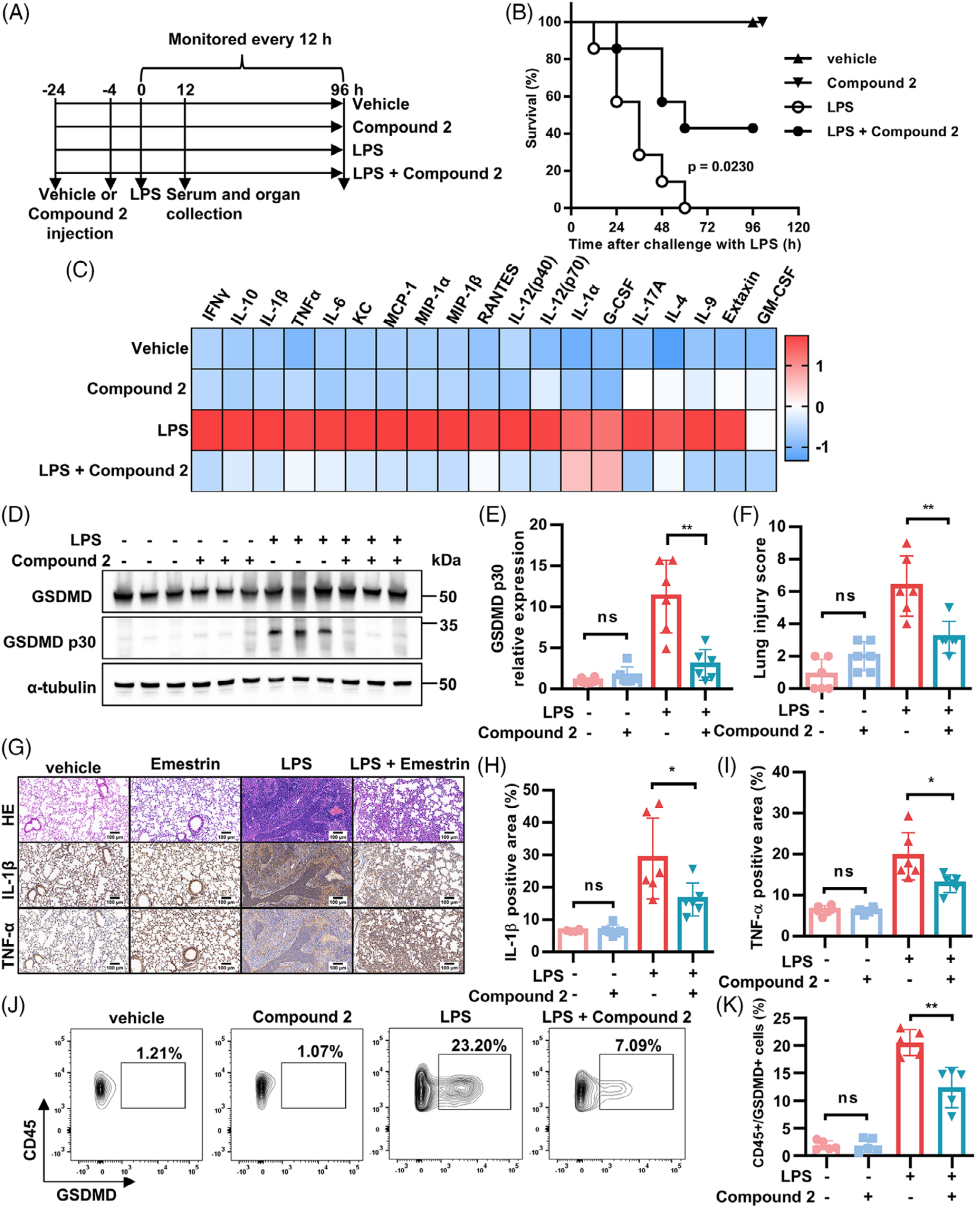
Compound **2** protected against LPS‐induced sepsis in mice. (A) Workflow diagram for in vivo experiment design. (B) Kaplan–Meier survival plot of mice pretreated with compound **2** (2 mg/kg) or vehicle by intraperitoneal injection 24 and 4 h before intraperitoneal challenge with 25 mg/kg LPS (*n* = 7 per group, log rank test, *p* = 0.0230). (C) Serum inflammatory chemokines were measured by luminex liquid suspension chip. (D and E) Western blotting analysis of GSDMD cleavage and α‐tubulin in mouse lung tissue samples 12 h post 25 mg/kg LPS injection. (F and I) HE staining and IHC staining of IL‐1β, TNF‐α in mouse lung tissue samples. Lung injury score and IL‐1β, TNF‐α positive area were analyzed. For E–I, data were compared by *t*‐test (mean ± SD; *n* = 6 per group, ns means not significant, *p < 0.05; **p < 0.01). (J and K) Flow cytometry analysis of CD45+ and GSDMD+ cells in lung samples from mice model 12 h post 25 mg/kg LPS injection (mean ± SD, *n* = 5 per group, ns means not significant, **p < 0.01).

The in vivo efficacy of compound **2** was evaluated in a C57BL/6J mouse model of LPS‐induced sepsis. Mice were pretreated intraperitoneally with either vehicle or compound **2** (2 mg/kg) twice before challenge with a lethal dose of LPS (25 mg/kg) (Figure 5A). In the vehicle group (LPS only), all seven mice succumbed within 60 h after LPS injection. By contrast, pretreatment with compound **2** significantly improved survival, with three out of seven mice remaining alive (*p* = 0.0230). Intraperitoneal administration of compound **2** (2 mg/kg) alone showed no observable toxicity for up to 96 h posttreatment (Figure 5B).

Moreover, serum levels of IL‐1β, TNF‐α, and IFN‐γ were significantly reduced in compound **2**‐treated mice compared with the vehicle group at 12 h after LPS challenge, prior to lethality (Figure 5C). Since compound **2** treatment can protect against LPS‐induced sepsis, we next determined the major organs affected by LPS treatment. Western blotting revealed GSDMD p30 cleavage in the liver, spleen, and lung (Figure S9A).

ALI in LPS‐induced sepsis models provides critical insights into the pulmonary sequelae of sepsis and serves as a foundational platform for developing targeted therapies [28]. Subsequently, we assessed the extent of lung injury in our murine model of sepsis. Consistent with this, treatment with compound **2** significantly attenuated the cleavage of GSDMD p30 in lung tissues from LPS‐challenged mice (Figure 5D,E). Accordingly, the lung injury score, along with immunohistochemical staining for IL‐1β and TNF‐α, was markedly attenuated in mice treated with LPS plus compound **2**, compared with those receiving LPS alone (Figure 5F–I). To extend our findings, we performed H&E staining on liver and spleen sections (Figure S9B–D), which revealed a concomitant reduction in inflammatory damage in these organs upon administration of compound **2**. The proportion of CD45 and GSDMD double‐positive cells in murine lung tissue was significantly lower in the group receiving LPS and compound **2** than in the LPS group (Figure 5J,K). Administration of compound **2** also led to a significant reduction in the proportions of both GSDMD‐positive monocytes and neutrophils in LPS‐challenged mice, a finding consistent with its overarching anti‐inflammatory effects (Figure S9E–G).

To better mimic the clinical scenario of sepsis, we employed the CLP model in subsequent experiments. Administration of compound **2** significantly enhanced survival rates, with 30% of treated mice surviving at the 96‐h time point compared with none in the vehicle control group (Figure S10A; *p* = 0.0152). Furthermore, flow cytometric analysis of lung tissue revealed a marked reduction in the infiltration of GSDMD‐positive neutrophils and monocytes (Figure S10B–D). Consequently, the lung injury score was significantly lower in mice subjected to CLP and treated with compound **2** than in those that underwent CLP alone (Figure S10E,F).

### 2.6 Expression Profiles of Lung Immune Cells with Compound 2 in Response to LPS‐Induced Injury

To determine the immune cell populations undergoing pyroptosis following LPS challenge, we analyzed cell suspensions of PBMCs, spleen, and lung tissues. GSDMD‐positive cells were predominantly F4/80+ macrophages (Figure S11A–C). Pyroptosis of immune cells in LPS‐induced lung injury exacerbates inflammation and tissue damage by activating the NLRP3 inflammasome, which subsequently triggers caspase‐1 activation, cytokine release, and a self‐amplifying cycle of cell death and immune cell recruitment [29, 30]. Alveolar macrophages, neutrophils, and epithelial cells were the major populations undergoing pyroptosis after LPS stimulation, thereby driving inflammatory responses and tissue damage [31, 32].

**FIGURE 6:**
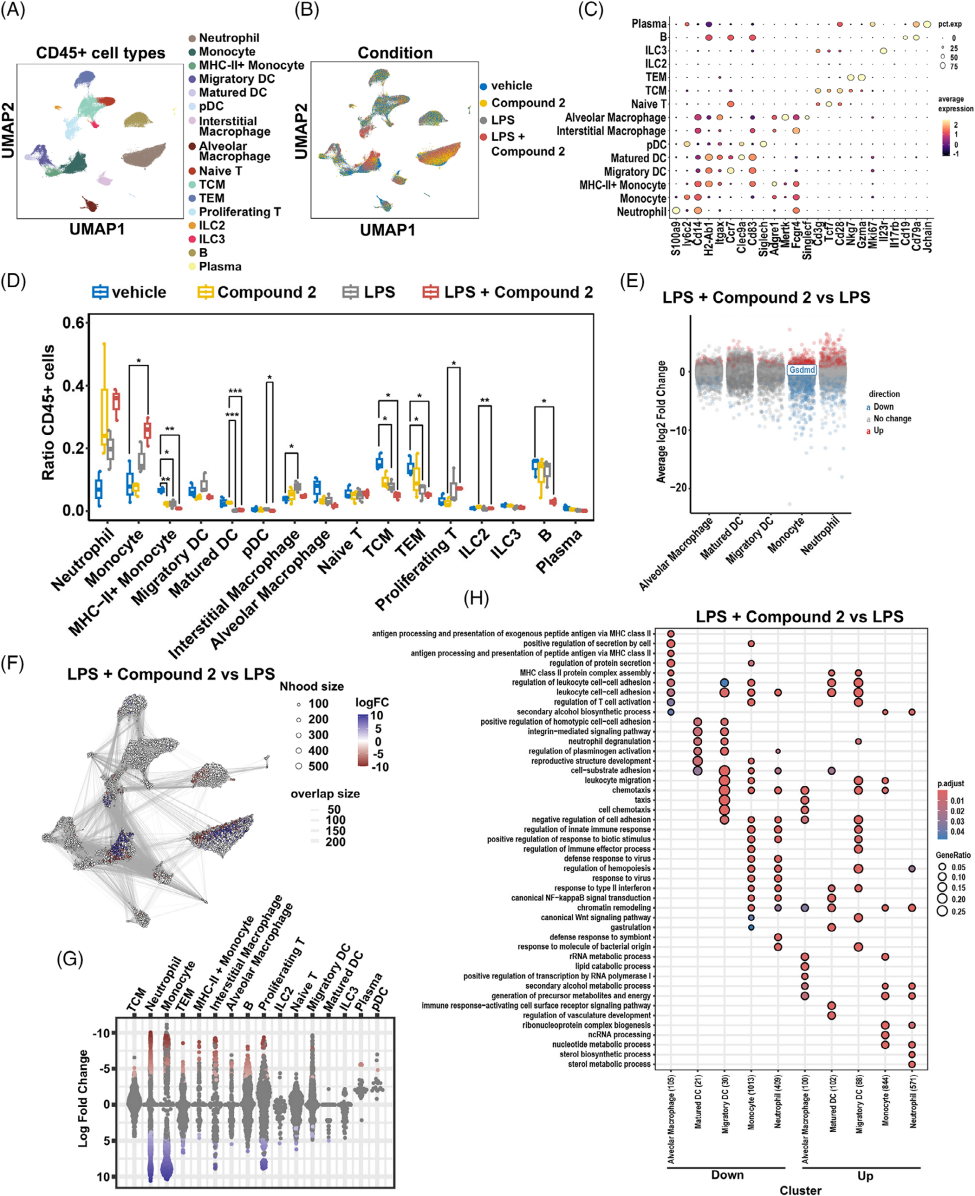
Expression profiles of lung immune cells with compound **2** and response to LPS‐induced injury. (A) Uniform manifold approximation and projection (UMAP) map of 94,383 lung immune cells grouped by cell types. (B) UMAP map of lung immune cells colored by experimental conditions: vehicle, compound **2**, LPS, and LPS with compound **2**. (C) Dotplot showing the expression of representative markers for each cell type across experimental conditions. (D) Boxplot illustrating the percentage of cell types in vehicle (*n* = 3), compound **2** (*n* = 3), LPS (*n* = 3), and LPS with compound **2** (*n* = 3) conditions; exact *p* values from paired *t*‐tests for all conditions are indicated: *p < 0.05, **p < 0.01, ***p < 0.001. Boxes represent the median ± interquartile range. (E) Scatterplot displaying pseudobulk differential expressed genes of myeloid cell types comparing LPS with compound **2** to LPS alone. Grey dots indicate nonsignificant differences (*p* > 0.05); red dots denote genes significantly upregulated (*p* < 0.05, log_2_FC > 0.6); blue dots denote genes significantly downregulated (*p* < 0.05, log_2_FC < –0.6). Statistical significance was determined using DESeq2. (F) Neighborhood graph depicting results from Milo differential abundance testing. Nodes represent neighborhoods colored by log fold change between LPS‐induced with and without compound **2**. Nondifferentially abundant neighborhoods (FDR 10%) are shown in white, with node size proportional to the number of cells. Graph edges represent shared cells between neighborhoods, and node layout corresponds to UMAP cell position. (G) Beeswarm plot displaying log fold change distributions across neighborhoods containing cells from different cell type clusters comparing LPS with compound **2** to LPS alone. Differentially abundant neighborhoods at FDR 10% are color coded. Cell types identified as differentially abundant are labeled. (H) Gene ontology analysis of up‐ and downregulated differentially expressed genes in myeloid cells between LPS with compound **2** and LPS alone conditions. *Abbreviations*: UMAP: uniform manifold approximation and projection; FDR: false discovery rate; log_2_FC: logarithm of fold change.

To further characterize the immune subpopulations responsive to LPS and compound **2** treatments, we performed scFAST‐seq on CD45+ enriched cells from LPS‐injured and control mouse lung samples, treated with either compound **2** or vehicle, in triplicate. Quality control excluded 52,344 cells, including low‐quality cells, doublets, high mitochondrial content cells, and outliers (Figure S12A), leaving 94,383 high‐quality cells for downstream analysis. These cells were clustered based on transcriptional similarity into 26 distinct clusters (Figure S12B). Marker genes for each cluster were identified, and cluster identity scores were estimated using the Immunological Genome Project (ImmGen) reference (Figure S12C) [33]. Based on marker gene expression and identity scores, cell‐type annotations were assigned to each cluster (Figure 6A,B).

Consistent with previous findings, the major myeloid populations included neutrophils (*S100a9*), monocytes (*Ly6c2*, *MHC‐II* high or low), macrophages (interstitial: *Fcgr4*, alveolar: *Siglecf*), and monocyte‐derived DCs (*Itgax*) with migratory (*Ccr7*) and mature (*Cd83*) states (Figure 6C). The identified T lymphoid population included naive T (*Tcf7*), central and effector memory T (TCM: *Cd28*, TEM: *Nkg7*, *Gzma*), type 2 and 3 innate lymphoid cells (ILC2, ILC3: *Il23r*), and proliferative T cells (*Mki67*) (Figure 6C). Additionally, B lymphocytes (*Cd19*, *Cd79a*), plasma cells (*Jchain*), and plasmacytoid DCs (*Siglech*) were identified (Figure 6C).

Compound **2** treatment induced the greatest number of differentially expressed genes (DEGs) in monocytes and neutrophils (Figure S13A–C). Upregulated genes in both cell types were enriched in glycolytic pathways, suggesting enhanced energy demand and activation of nonspecific trained immunity (Figure S13D,F) [34]. Conversely, downregulated genes were related to cell‐cycle regulation in both monocytes and neutrophils (Figure S13E,G), with enrichment in chromatin organization, heterochromatin processes, and CD8+ T cell activation in monocytes.

Upon LPS challenge, the relative proportions of neutrophils, monocytes, and interstitial macrophages were significantly increased, whereas TCM and TEM cells were reduced (Figure 6D). Treatment with compound **2** markedly decreased the frequency of MHC‐II monocytes, while migratory DCs showed a downward trend without statistical significance (Figure 6D). Notably, compound **2** reversed the LPS‐induced expansion of interstitial macrophages (Figure 6D). Interestingly, in LPS‐challenged mice, neutrophils and monocytes showed further expansion upon compound **2** treatment (Figure 6D), suggesting that both cell types were prone to pyroptosis under LPS stimulation and were potentially protected by compound **2**. This was corroborated by significantly reduced GSDMD expression in monocytes and neutrophils upon compound **2** treatment compared with vehicle controls (Figure 6E). Consistently, monocytes and neutrophils exhibited the most pronounced transcriptional alterations between LPS‐injured mice treated with compound **2** and vehicle.

As anticipated, genes related to immune activation, such as leukocyte adhesion, T cell activation, response to type II interferon, and canonical NF‐κB signal transduction, were downregulated in monocytes and neutrophils following compound **2** treatment (Figure 6H). Similarly, migratory and mature DCs exhibited reduced expression of genes associated with integrin signaling, leukocyte migration, and chemotaxis (Figure 6H). In contrast, genes upregulated in monocytes and neutrophils, regardless of compound **2** treatment or vehicle, were enriched in energy metabolism pathways (Figure 6H).

### 2.7 Cellular Immune Changes with Compound 2 Treatment in LPS‐Induced Injury

To investigate the immune‐modulatory effect of compound **2**, we applied Milo differential abundance testing [35] to track compositional shifts in immune cell populations. This approach partitions cells into small, overlapping neighborhoods based on transcriptional similarity and detects significant enrichment in each condition using generalized linear models, thereby minimizing confounding and clustering biases. The most pronounced alterations induced by compound **2** were observed within the monocyte cluster (Figure 6F,G). Gene ontology enrichment of up‐ and downregulated genes across cell types, comparing LPS‐induced injury with or without compound **2** treatment, is summarized in Figure 6H.

To correlate in vitro and in vivo findings, we assessed chemokine secretion and transcriptomic changes in THP‐1 differentiated macrophages treated with compound **2** following LPS stimulation. At 12 h post‐LPS and nigericin challenge, compound **2** markedly reduced inflammatory chemokine release in culture supernatants (Figure S14A). Transcriptomic analysis further revealed that compound **2** upregulated genes related to metabolic pathways, including cytoplasmic translation, aerobic respiration, oxidative phosphorylation, mitochondrial translation, and general gene expression. In contrast, downregulated genes were enriched in pathways associated with Wnt signaling, TGF‐β responses, diverse cell differentiation programs, and amoeboid‐type migration (Figure S14B). LPS stimulation alone activated proinflammatory transcriptional programs, including responses to LPS, tumor necrosis factor, and cytokine‐mediated signaling, which were suppressed by compound **2** (Figure S14B). In total, 3438 genes (27.4%) were upregulated and 2963 genes (24.3%) were downregulated upon compound **2** treatment (Figure S14C,D). Functional enrichment of these DEGs is presented in Figure S14E–F.

### 2.8 Compound 2 Reduced Monocyte Antigen‐Presentation and DC Maturation

**FIGURE 7:**
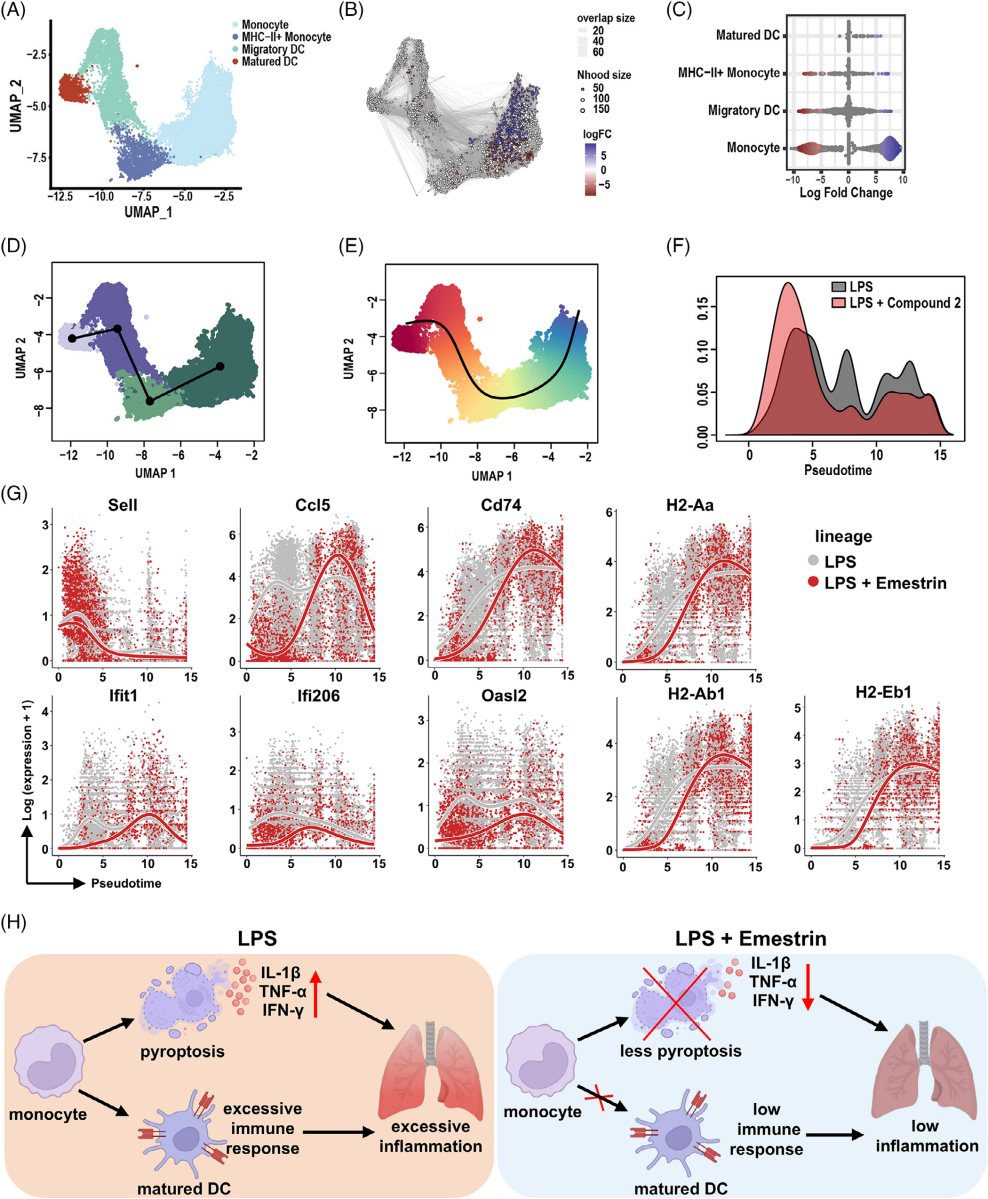
Trajectory analysis of LPS‐induced monocyte to matured DC with compound **2**. (A) UMAP visualization of the monocyte to matured dendritic cell (DC) differentiation trajectory. The plot illustrates distinct cellular clusters and transitions between cell states. (B) Neighborhood graph depicting results from Milo differential abundance testing. Nodes represent cellular neighborhoods colored by log fold change between LPS‐induced conditions with and without compound **2**. Nondifferentially abundant neighborhoods (FDR 10%) are colored white, with node size indicating the number of cells. Graph edges denote shared cells between neighborhoods, and node layout reflects UMAP cell positions. (C) Beeswarm plot showing the distribution of log fold change across neighborhoods containing cells from different cell type clusters comparing LPS with compound **2** to LPS alone. Differentially abundant neighborhoods at FDR 10% are color coded. Cell types identified as differentially abundant are indicated. (D and E) Pseudotime trajectories for monocytes based on Slingshot analysis, representing a single trajectory consisting of four nodes: monocyte, MHC‐II+ monocyte, migratory DC, and matured DC. (F) Distribution of cells along the pseudotime trajectory in LPS‐induced conditions with and without compound **2** treatment. (G) Representation of differential trajectory genes associated with antigen presentation (*Cd74*, *H2‐Aa*, *H2‐Ab1*, and *H2‐Eb1*), leukocyte migration (*Sell* and *Ccl5*), and interferon gamma response (*Ifit1*, *Ifi206*, and *Oasl2*). (H) Graphical depiction of the hypothesized effects of compound **2** treatment in LPS‐induced acute lung injury, illustrating its potential modulation of monocyte to matured DC differentiation trajectory and associated gene expression changes. *Abbreviations*: UMAP: uniform manifold approximation and projection; FDR: false discovery rate; DEGs: differentially expressed genes.

To delineate the impact of compound **2** on monocyte maturation into DCs, we further subdivided the monocyte‐DC compartment. Nodes proximal to MHC‐II monocytes exhibited greater fold changes compared with those further along the DC maturation trajectory (Figure 7A–C).

Based on our previous observations, we hypothesized that compound **2** modulates monocyte antigen presentation and inhibits their differentiation into DCs. Trajectory analysis revealed a gradient of gene expression from infiltrated monocytes to antigen presentation, migration, and DC maturation states (Figure 7D,E). Comparing LPS‐injured samples with or without compound **2** demonstrated that compound **2** partially suppressed early migration and maturation along this trajectory (Figure 7F). A total of 584 genes showed significant differences along the trajectory and were clustered into eight expression modules (Figure S15). Notably, expression of *Sell* (*Cd62l*), an adhesion/homing receptor downregulated during tissue infiltration, was reduced (Figure 7G). Key antigen presentation genes, including *H2‐Ab1*, *Cd74*, *H2‐Eb1*, and *H2‐Aa*, increased across the trajectory but were attenuated by compound **2** treatment (Figure 7G). Genes related to interferon responses, such as *Ifit1*, *Ifi206*, and *Oasl2*, were also suppressed by compound **2** (Figure 7G). Collectively, these findings suggest that compound **2** reduces monocyte pyroptosis and inhibits their differentiation into mature DCs in LPS‐induced ALI (Figure 7H).

## 3 Discussion

Sepsis remains a major global health challenge due to prolonged inflammation, dysregulated immune responses, high susceptibility to infection, and mortality. Pyroptosis, a form of programmed cell death distinct from apoptosis, is tightly linked to hyperinflammation [36]. GSDMD‐mediated pyroptosis plays a critical role in sepsis pathogenesis [7, 14]. Upon activation, the N‐terminal fragment of GSDMD translocates to the plasma membrane, forming pores that compromise integrity, causing cell swelling, rupture, and release of cytoplasmic contents. This process unleashes proinflammatory cytokines, including IL‐1β and IL‐18, further amplifying inflammation [37]. In severe sepsis, uncontrolled pyroptosis exacerbates tissue injury, drives organ dysfunction, and worsens disease outcomes. Targeting GSDMD‐mediated pyroptosis therefore represents a promising therapeutic strategy for severe sepsis [38].

Currently identified small molecules inhibiting GSDMD cleavage target cysteine‐191/192 of GSDMD [18, 19, 20, 21]. Caspase‐3/7‐mediated apoptosis can also suppress pyroptosis by cleaving GSDMD at D87, thereby preventing membrane pore formation by the p30 fragment [25, 26]. This mechanism not only ensures clearance of infected cells but also functions as a counter‐regulatory pathway to dampen pyroptosis and inflammation, potentially improving survival in severe inflammatory states. Notably, the baseline GSDMD cleavage observed in unstimulated cells aligns with established mechanisms where proteinaceous dietary antigens [26] or metabolic stress [39] constitutively activate caspases, triggering nonlytic GSDMD fragmentation independent of canonical inducers like LPS/nigericin.

ETPs are a class of 2,5‐diketopiperazine derivatives with demonstrated antibacterial, immunomodulatory, and cytotoxic properties. However, their precise molecular targets have remained largely elusive. To elucidate this, we employed SPR analysis, which identified procaspase‐3 as a direct binding target of these compounds. Structurally, emestrin‐type ETPs feature key pharmacophores such as a piperazine ring and a phenolic hydroxyl group that are also present in the established procaspase‐3 activator PAC‐1 [27]. Based on this structural insight and functional precedent, we propose that emestrin‐type ETPs act as direct allosteric activators of procaspase‐3. They induce a conformational change that alleviates zymogen auto‐inhibition and facilitates catalytic auto‐activation, thus offering a novel therapeutic strategy for sepsis by shifting cell death from inflammatory pyroptosis to noninflammatory apoptosis. Future structural optimization could focus on improving binding kinetics while maintaining catalytic efficiency.

LPS‐induced sepsis triggers multiorgan dysfunction, with ALI representing a life‐threatening complication driven by Gram‐negative bacterial LPS [40, 41]. This pathogen‐associated molecular pattern stimulates inflammatory mediators (e.g., TNF‐α, IL‐1β) that activate neutrophils and accumulate in bronchoalveolar lavage fluid of ARDS patients [42, 43, 44]. Given the critical role of ALI in sepsis mortality, studying LPS‐induced models remains essential for developing targeted therapies. Notably, macrophage pyroptosis suppression has emerged as a key protective mechanism against sepsis‐related ALI [45, 46, 47]. While established pyroptosis inhibitors like NSA provide valuable benchmarks, our compound's unique caspase‐3/7‐dependent mechanism (versus direct GSDMD targeting) justified initial standalone efficacy assessment. In our study, compound **2** significantly improved the survival rate of mice in the LPS challenge model, protecting against pulmonary injury by inhibiting the pyroptosis of CD45+ immune cells and the release of proinflammatory cytokines.

Leveraging single‐cell RNA sequencing, we characterized lung immune cell dynamics in response to LPS‐induced injury with compound **2** treatment. Analysis revealed marked downregulation of GSDMD expression in monocytes, accompanied by suppression of immune effector processes, type II interferon signaling, and canonical NF‐κB transduction pathways. Trajectory inference further demonstrated that monocytes differentiate into antigen‐presenting DCs upon LPS challenge—a process attenuated by compound **2**. Given DCs’ critical role in bridging innate and adaptive immunity [48, 49] and their capacity to exacerbate inflammation via TLR4‐mediated cytokine production [50], our findings establish that compound **2** mitigates sepsis severity by inhibiting monocyte‐to‐DC maturation, thereby reducing antigen presentation, interferon responses, and DC migratory functions.

In summary, our experiments demonstrated that emestrin‐type ETPs can mitigate GSDMD‐mediated inflammation both in vitro and in vivo. These findings provide new insights and potential avenues for developing treatments for LPS‐induced lung inflammation. However, it is important to acknowledge two key limitations of this study: (1) while compound **2** effectively attenuates GSDMD‐mediated pyroptosis in the lung, liver, and spleen, its effects on brain and intestinal inflammation remain unexamined. Future studies will investigate neuroinflammation and intestinal barrier dysfunction via GSDMD regulation, leveraging existing sepsis pyroptosis evidence [51, 52]; and (2) although compound **2** reduces DC maturation and systemic inflammation, the precise mechanisms of its immunomodulatory effects—particularly caspase‐3/7‐dependent regulation of cellular immunity—require further investigation. Future research should focus on optimizing these compounds for clinical use and exploring their potential in other inflammatory diseases, paving the way for novel treatment strategies in sepsis and beyond.

## 4 Materials and Methods

### 4.1 Cell Culture and Pyroptosis Induction

THP‐1 cells, obtained from ATCC, were used for all cell experiments. The cells were cultured at a density of 1 × 10^6^ cells/mL in RPMI 1640 medium supplemented with 15% FBS, at 37°C in a 5% CO_2_ incubator. Prior to treatment, THP‐1 monocytes were differentiated into adherent macrophages by incubating with 100 nM phorbol 12‐myristate 13‐acetate (PMA) (Sigma; P8139) for 48 h. Following differentiation, cells were starved for 24 h in RPMI 1640 medium. The starved cells were then primed with 1 µg/mL LPS (Sigma; L4391) and 3 mM ATP (Sigma; A2383), and supplemented with either vehicle or the indicated compounds for 3 h. Subsequently, the cells were treated with 10 µM nigericin (Selleck; S6653) for 1 h. Both the cells and culture medium were collected for further analysis.

### 4.2 High‐Content Screening

For HCS, THP‐1 cells were differentiated with PMA and seeded into 96‐well plates at a density of 6 × 10^4^ cells/well for 48 h. Prior to treatment, the cells were starved in RPMI 1640 medium for 24 h. Starved cells were then primed with 1 µg/mL LPS and 3 mM ATP, each supplemented with one of 536 different compounds in separate wells at a final concentration of 1 µM for 3 h. Following this, cells were treated with 10 µM Nigericin and 1 µM YO‐PRO‐1 (Beyotime; C2022) for 1 h. The cells were washed with PBS and fixed with paraformaldehyde (PFA) for 10 min. Fixed cells were then imaged and analyzed using the Opera Phenix HCS system (Revvity). Repeatability metric: *R* = |viability^EXP1^ – viability^EXP2^|; repeatability score: –log_10_ (*R*/viability) (threshold: >1.5, equivalent to <3.2% variability). The viability threshold was set at 0.7 (70%) to prioritize compounds with specific antipyroptotic effects while excluding nonspecific cytotoxicity. This value was empirically validated against control data and aligns with HTS conventions for balancing sensitivity and specificity.

### 4.3 N‑Terminal Sequence Analysis

The specific sequences of p10 fragments were analyzed by Beijing Bio‐Tech Pack Technology Company Ltd. (Beijing, China). Protein samples were separated by SDS‐PAGE on a 4–20% gradient gel and stained with Coomassie Blue Super Fast Staining Solution (Beyotime; P0017F). After destaining, the specific bands were excised and subjected to enzymatic hydrolysis using chymotrypsin and trypsin, respectively. The resulting peptides were analyzed by LC–MS/MS, and raw data were processed using PEAKS Studio software.

### 4.4 Elisa and LDH Assays

Differentiated THP‐1 cells were primed with LPS and ATP and supplemented with the indicated compounds at a concentration of 1 µM for 3 h. Cells were then stimulated with nigericin (10 µM) for 1 h, after which culture supernatants were collected. Levels of lactate LDH, IL‐1β, and IL‐18 were quantified using commercial assay kits (Hycezmbio, China) according to the manufacturer's instructions.

### 4.5 Western Blotting

Western blotting was performed following standard protocols. Total proteins were extracted and resolved on 4–20% FuturePAGE precast gels (ACE Biotechnology, China), then transferred onto PVDF membranes. After blocking with 5% nonfat milk for 1 h at room temperature, membranes were incubated with specific primary antibodies overnight at 4°C. Subsequently, membranes were incubated with appropriate secondary antibodies for 1 h at room temperature, and protein bands were visualized using enhanced chemiluminescence reagents (Hycezmbio). The following primary antibodies were used: GSDMD (Abcam; ab219800), α‐tubulin (Servicebio; GB11200‐100), caspase‐1 (Abcam; ab179515), caspase‐3 (Abmart; T40044), procaspase‐7 (Huabio; ET1612‐28), and caspase‐7 p20 (Huabio; ER60002).

### 4.6 Immunofluorescent Staining

Cells were cultured under various conditions, then fixed with 4% PFA. Following permeabilization, 10% goat serum was applied for 30 min at room temperature to block nonspecific protein binding sites. Cells were then incubated with primary antibody GSDMD (Abcam; ab219800) overnight at 4°C. This antibody can recognize the full‐length GSDMD (55‐kDa), pyroptosis‐associated p30 fragment (30‐kDa), and apoptosis‐derived p10 fragment (13‐kDa). Subsequently, cells were incubated with secondary antibody Alexa Fluor 647 (Invitrogen; A21244) for 1 h at room temperature, washed with PBS, and stained with DAPI for nuclear visualization. Stained cells were visualized using an Olympus FV3000 confocal system. GSDMD plasma membrane positivity was defined by colocalization with cell membranes using immunofluorescence imaging, with quantitative thresholds established through Pearson's correlation analysis (>0.7).

### 4.7 Live Cell Imaging

Differentiated THP‐1 cells were seeded in a 96‐well plate and cultured under various conditions. YO‐PRO‐1 (1 µM) was added to the culture medium to indicate cell death. The plate was loaded into the live cell application system (Sartorius; IncuCyte S3) for 12 h of imaging. Data were analyzed using the IncuCyte S3 system.

### 4.8 Luminex Liquid Suspension Chip Detection

Luminex liquid suspension chip detection was conducted by Wayen Biotechnologies (Shanghai, China). THP‐1‐derived macrophages were seeded in a six‐well plate and treated with the indicated interventions. Culture media were collected for the Bio‐Plex Pro Human Chemokine Panel kit assay. Animal serum was also collected for the Bio‐Plex Pro Mouse Chemokine Panel kit assay. Results were analyzed using GraphPad Prism software.

### 4.9 Protein Activation Curves

Recombinant human procaspase‐3 protein (CUSABIO; Cat# CSB‐EP004548HU) was diluted to 200 ng/µL in activation buffer (20 mM HEPES, pH 7.5, 100 mM NaCl, 1 mM EDTA, 10% glycerol, and 0.1% CHAPS). Various compounds (5 µM) and the positive control PAC‐1 (5 µM) were added to the protein solution and incubated at 37°C for 12 h. Caspase‐3 activation was quantified using the Caspase‐3 Activity Assay Kit (Beyotime; C1116) according to the manufacturer's instructions, with fluorescence readings (Ex/Em = 488/530 nm) taken every 30 min. Parallel samples were analyzed by western blotting under nonreducing conditions using anticaspase‐3 antibody (Abmart; T40044). Band intensities were quantified with ImageJ software and normalized to the vehicle control.

### 4.10 Molecular Docking Analysis

Molecular docking was performed using AutoDock Vina 1.1.2. The human procaspase‐3 protein crystal structure (PDB ID: 3DEK) was obtained from the PDB database. The 3D structure of the target small molecule was constructed using Chem3D, with energy minimization using the MMFF94 force field. Prior to docking, the protein was prepared using PyMol 2.5 software, including dehydrogenation and removal of water and nonligand small molecules. The docking box was defined to surround the protein's active pocket. Small molecules and receptor proteins were converted from PDB to PDBQT format using ADFRsuite 1.02. Docking was performed with the conformation search exhaustiveness set to 32. The conformation with the highest affinity score was identified as the correct conformation and visually analyzed using PyMol 2.5.

### 4.11 Surface Plasmon Resonance

The equilibrium‐binding constant (*K*_d_) of various compounds with recombinant human procaspase‐3 protein was determined using SPR on a Biacore T200 system (Cytiva) at 25°C. Caspase‐3 protein was immobilized on Series S CM5 sensor chips (Cytiva) using amine‐coupling chemistry, achieving an immobilization level of 12,000–14,000 resonance units (RU). Compounds were diluted to 100 µM with running buffer and injected into the chip. In each cycle, a 100 µL sample was passed through the chip for 60 s at a constant flow rate of 30 µL/min. Compound **2** was serially diluted to concentrations of 12.5, 25, 50, 100, and 200 µM in the running buffer and sequentially injected into the chip from low to high concentrations. Each cycle used a 200 µL sample at the indicated concentrations with a contact time of 120 s and a dissociation time of 180 s. Data were analyzed using Biacore Insight Evaluation Software Version 3.0.12 with a steady‐state affinity 1:1 binding model.

### 4.12 Animal Studies

Male mice weighing 25–30 g were used in the study. All mice were purchased from Shulaibao Biotechnology Co., Ltd., Wuhan. Animals were maintained on a 12/12‐h light/dark cycle and provided with water and a standard rodent diet. Moribundity was used as the endpoint for the survival study, in accordance with the Animal Research Advisory Committee Guidelines. Compound **2** was dissolved in 2% DMSO (Sigma–Aldrich; D8418), 30% PEG300 (MedChemExpress; HY‐Y0873), 5% Tween80 (Sigma–Aldrich; P1754), and 63% ddH_2_O. Compound **2** (2 mg/kg body weight) was intraperitoneally injected into mice 24 and 4 h before the LPS (Sigma–Aldrich; L3024) challenge (25 mg/kg body weight). The control group received the same volume of vehicle. For the survival experiment, mice were monitored every 12 h by personnel experienced in recognizing signs of moribundity. For other experiments, various organ tissues and serum samples were collected 12 h after the LPS injection and stored at –80°C for further testing.

### 4.13 Lung Histology

After cardiac perfusion with heparin saline, the left upper lungs of mice were isolated and fixed in 4% PFA for 24 h, then routinely processed in paraffin. Sections, 4 µm thick, were cut for hematoxylin and eosin staining. For IHC staining, 4 µm‐thick lung sections were deparaffinized in xylene and rehydrated through a series of graded EtOH washes. Antigen retrieval was performed using Tris–EDTA buffer (10 mM Tris pH 9.0, 1 mM EDTA) for 30 min at 95°C, followed by 5‐min incubation with proteinase K at room temperature. Lung tissue sections were blocked with 10% BSA in TBS for 1 h at room temperature, then incubated overnight at 4°C with IL‐1β (Servicebio; GB11113‐100) and TNF‐α (Servicebio; GB11188‐100) primary antibodies. Horseradish peroxidase‐conjugated secondary anti‐rabbit immunoglobulins were used as appropriate, and the color reaction was developed with 0.1% 3,3‐diaminobenzidine tetrachloride/0.01% hydrogen peroxide. The positive areas of IL‐1β and TNF‐α were semi‐quantitatively calculated using Image‐Pro Plus 6.0.

### 4.14 Flow Cytometry

Lungs and spleens were isolated from mice and cut into small pieces. Whole blood samples were obtained from the inferior vena cava using a 1 mL syringe rinsed with heparin. Lung samples were digested into cell suspensions using a commercial kit (SeekMate; k01801‐30) following the manufacturer's instructions. Spleen samples were filtered through a 70 µm cell strainer (Miltenyi; 130‐098‐462) to create a cell suspension. PBMCs were isolated from whole blood samples using a commercial kit. Cell suspensions were incubated with the following antibodies: APC/Cyanine7 anti‐mouse CD45 (Biolegend; 103116), FITC anti‐mouse CD3ε (Biolegend; 155604), PE‐Cy7 rat anti‐mouse Ly‐6G (BD Pharmingen; 560601), PE rat anti‐mouse F4/80 (BD Pharmingen; 565410), PerCP‐Cy5.5 rat anti‐mouse CD19 (BD Pharmingen; 551001), GSDMD (Abcam; ab219800), and goat anti‐rabbit IgG Alexa Fluor 647 (Invitrogen; A21244) as the secondary antibody for GSDMD, along with Fixable Viability Stain 700 (BD Pharmingen; 564997). Cells were then washed with PBS and analyzed using a BD flow cytometer and FlowJo software.

### 4.15 Detection of mRNA Profiles

THP‐1 differentiated macrophages were subjected to specified treatments, followed by extraction of total RNA using a commercial kit (TIANGEN; DP419). The isolated RNA was sequenced by BGI Co., Ltd. (Shenzhen, China) using RNA‐seq on a BGISEQ‐500 instrument. Sequencing results were further analyzed using R, version 3.5.1.

### 4.16 Single Cell Sequencing of Mouse Lung Samples

After cardiac perfusion with heparin saline, the left upper lungs of mice from different groups were isolated and stored in tissue preservation solution. The scFAST‐seq library was prepared using the SeekOne Single Cell Whole Transcriptome Kit according to the manufacturer's instructions (SeekGene; Cat# K00801). Sequencing results were processed and analyzed using R, version 3.5.1 (see Supporting Information). The scFAST‐seq data generated in this study have been uploaded to the National Center for Biotechnology Information database. The original datasets are also available from the corresponding author upon reasonable request.

### 4.17 Statistical Analysis

Error bars are presented as mean ± SD. The number of independent experiments (*n*) is described in the legends for all figures. For comparison of two groups, unpaired two‐sided Student's *t*‐tests were used. *p* Values less than 0.05 were considered statistically significant for all experiments.

## Author Contributions

This work was designed and guided by Zhengxi Hu, Yonghui Zhang, and Muwei Li, and the manuscript was revised by Wai Yen Yim and Weiguang Sun. Bingchuan Geng and Shuang Lin contributed to the bioactivity tests, extraction, isolation, identification, and manuscript preparation. Xiaotian Zhang, Hanxiao Zeng, and Yixuan Wang provided assistance for extraction and data analysis. Cao Ma, Zhiwen Zhang, Quan Guo, Jie Gao, Qingyi Tong, Zhengfeng Fan, and Jincheng Hou provided assistance for the bioactive study. All authors have read and approved the final manuscript.

## Funding

This work was supported by the Hubei Provincial Natural Science Foundation of China (No. 2024AFA028), the Fundamental Research Funds for the Central Universities (No. 2025BRA015), the National Natural Science Foundation of China (Nos. 82273811, 22577033, 82470380, 82304340, and 82470423), the National Program for Support of Top‐notch Young Professionals (No. 0106514050), the China Postdoctoral Science Foundation (No. 2021M701330), the National Key Research and Development Program of China (No. 2021YFA0910500), and the Open Fund of Hubei Jiangxia Laboratory.

## Conflicts of Interest

Author Yonghui Zhang is an Editorial board member of MedComm. Author Yonghui Zhang was not involved in the journal's review of or decisions related to this manuscript. The other authors declare no conflicts of interest.

## Ethics Statement

Experimental protocols were approved by the Animal Care and Use Committee at the Wuhan Food and Drug Safety Evaluation Center (IACUC# 202310075).

## Supporting information




**Supporting Figure 1**: High content screening of 536 compounds. (A) YO‐PRO‐1 staining results of HCS. (B) Individual pictures for indicated treatments.
**Supporting Figure 2**: Selected 1H–1H COSY (blue bold lines) and HMBC (red arrows) correlations ofcompounds 1, 3–6, and 10–13.
**Supporting Figure 3**: Selected NOESY/ROESY correlations of compounds 1, 3, 4, 6, and 10–13.
**Supporting Figure 4**: X‐Ray crystallographic structures of 3–6.
**Supporting Figure 5**: CD curves of compounds. (A) Experimental ECD curves of compounds 1, 4–6, and 13. (B) Experimental ECD curves of compounds 3 and 12. (C) Experimental ECD curves of compounds 10–11 and calculated ECD curve of 11.
**Supporting Figure 6**: NMR calculations with DP4+ probability analysis for 1, 3, and 10. (A) Linear correlation plots of experimental vs. calculated 1H NMR (left) and 13C NMR (right) chemical shifts of 1. (B) Linear correlation plots of experimental vs. calculated 1H NMR (left) and 13C NMR (right) chemical shifts of 3. (C) Linear correlation plots of experimental vs. calculated 1H NMR (left) and 13C NMR (right) chemical shifts of 10. (D) DP4+ probability analysis.
**Supporting Figure 7**: NMR calculations with DP4+ probability analysis for 11–13. (A) Linear correlation plots of experimental vs. calculated 1H NMR (left) and 13C NMR (right) chemical shifts of 11. (B) Linear correlation plots of experimental vs. calculated 1H NMR (left) and 13C NMR (right) chemical shifts of 12. (C) Linear correlation plots of experimental vs. calculated 1H NMR (left) and 13C NMR (right) chemical shifts of 13. (D) DP4+ probability analysis.
**Supporting Figure 8**: Mutagenesis analysis of pro‐caspase‐3 in HEK293T cells.(A) Western blotting analysis of HEK293T cells overexpressing wild type or different point mutation pro‐caspase‐3 protein treated with compound 2 (1 µM). (B) A predicted binding site of compound 2 in pro‐caspase‐3 protein is conserved across the indicated species.
**Supporting Figure 9**: Compound 2 protected against LPS‐induced sepsis in mice. (A) Western blotting analysis of GSDMD cleavage and α‐tubulin in mouse different organ tissue samples 12 h post 25 mg/kg LPS injection. (B–D) HE staining of mouse liver and spleen tissue samples 12 h post 25 mg/kg LPS injection. Liver and spleen injury score were analyzed. (mean ± SD; *n* = 5 per group, ***p < 0.001). (E–G) Flow cytometry analysis of GSDMD‐positive neutrophils and monocytes in lung samples from mice model 12 h post 25 mg/kg LPS injection (mean ± SD, *n* = 5 per group, **p < 0.01; ***p < 0.001).
**Supporting Figure 10**: Compound 2 protected against CLP‐induced sepsis in mice. (A) Kaplan‐Meier survival plot of mice subjected to CLP surgery following pretreatment with compound 2 (2 mg/kg) or vehicle (*n* = 10 per group, log rank test, *p* = 0.0152). (B–D) Flow cytometry analysis of GSDMD‐positive neutrophils and monocytes in lung samples from mice model 12 h post CLP surgery (mean ± SD, *n* = 5 per group, ns means not significant, *p < 0.05; ***p < 0.001). (E–F) HE staining of mouse lung tissue samples. Lung injury score was analyzed (mean ± SD; *n* = 5 per group, *p < 0.05).
**Supporting Figure 11**: Flow cytometry analysis of different organ tissue in mouse LPS challenge model.(A) Gating strategy for different immune cells in mouse LPS challenge model. (B–C) Flow cytometry analysis of PBMC, spleen and lung tissue in mouse LPS challenge model.
**Supporting Figure 12**: Preprocessing, dimensional reduction, clustering, and cell annotation of scRNAseq data.(A) Barplot showing number of filtered and non‐filtered cells in each sample. (B) UMAP and clustering of cells. (C) Identity score of each clusters using CIPR with Immgen as reference.
**Supporting Figure 13**: Gene expression changes of lung immune cells treated with compound 2. (A) Scatterplot showing pseudobulk differential expressed genes of immune cell types comparing compound 2 with vehicle. Grey dots, *p* > 0.05; red, *p* < 0.05 and log2FC > 0.6 (up‐regulated), blue, *p* < 0.05 and log2FC < –0.6 (down‐regulated). *p*‐values were obtained by DESeq2 testing. (B) Intersect of up‐regulated differentially expressed genes. (C) Intersect of down‐regulated differentially expressed genes. (D) Enriched gene ontology terms of up‐regulated intersected gene modules. (E) Enriched gene ontology terms of down‐regulated intersected gene modules. (D–E, adjusted *p* < 0.05.) (F–G) Plot of gene‐concept networks using Cnetplot function of clusterProfiler to identify overlapping genes based on the enriched gene ontology terms in d and e respectively.
**Supporting Figure 14**: Inflammatory chemokines release result and RNA‐seq analysis of THP‐1 differentiated macrophages. (A) Inflammatory chemokines of THP‐1 differentiated macrophages culture medium were measured by luminex liquid suspension chip. (B) Enriched gene ontology term of up‐ and downregulated differentially expressed genes across conditions (control, *n* = 3; compound 2, *n* = 3, LPS, *n* = 3, LPS + compound 2, *n* = 3) with human THP‐1 differentiated macrophages. (C–D) Intersect of genes up‐ (C) and down‐regulated (D) in THP‐1 cell line and lung monocyte mice in response to LPS with compound 2 compare to LPS only. (E–F) Enriched Gene ontology biological process terms of the intersected and non‐intersected genes in C and D.
**Supporting Figure 15**: Pseudotime trajectory DEGs (absolute fold change > 1, adjusted *p*‐value < 0.05) between conditions clustered into 8 modules based on expression patterns.
**Supporting Figure 16**: 1H NMR spectrum of compound 1 (Recorded in CDCl3).
**Supporting Figure 17**: 13C NMR and DEPT spectra of compound 1 (Recorded in CDCl3).
**Supporting Figure 18**: HSQC spectrum of compound 1 (Recorded in CDCl3).
**Supporting Figure 19**: HMBC spectrum of compound 1 (Recorded in CDCl3).
**Supporting Figure 20**: 1H–1H COSY spectrum of compound 1 (Recorded in CDCl3).
**Supporting Figure 21**: NOESY spectrum of compound 1 (Recorded in CDCl3).
**Supporting Figure 22**: HRESIMS spectrum of compound 1.
**Supporting Figure 23**: UV spectrum of compound 1.
**Supporting Figure 24**: IR spectrum of compound 1.
**Supporting Figure 25**: 1H NMR spectrum of compound 3 (Recorded in CDCl3).
**Supporting Figure 26**: 13C NMR and DEPT spectra of compound 3 (Recorded in CDCl3).
**Supporting Figure 27**: HSQC spectrum of compound 3 (Recorded in CDCl3).
**Supporting Figure 28**: HMBC spectrum of compound 3 (Recorded in CDCl3).
**Supporting Figure 29**: 1H–1H COSY spectrum of compound 3 (Recorded in CDCl3).
**Supporting Figure 30**: NOESY spectrum of compound 3 (Recorded in CDCl3).
**Supporting Figure 31**: HRESIMS spectrum of compound 3.
**Supporting Figure 32**: UV spectrum of compound 3.
**Supporting Figure 33**: IR spectrum of compound 3.
**Supporting Figure 34**: 1H NMR spectrum of compound 4 (Recorded in CDCl3).
**Supporting Figure 35**: 13C NMR and DEPT spectra of compound 4 (Recorded in CDCl3).
**Supporting Figure 36**: HSQC spectrum of compound 4 (Recorded in CDCl3).
**Supporting Figure 37**: HMBC spectrum of compound 4 (Recorded in CDCl3).
**Supporting Figure 38**: 1H–1H COSY spectrum of compound 4 (Recorded in CDCl3).
**Supporting Figure 39**: NOESY spectrum of compound 4 (Recorded in CDCl3).
**Supporting Figure 40**: HRESIMS spectrum of compound 4.
**Supporting Figure 41**: UV spectrum of compound 4.
**Supporting Figure 42**: IR spectrum of compound 4.
**Supporting Figure 43**: 1H NMR spectrum of compound 5 (Recorded in CDCl3).
**Supporting Figure 44**: 13C NMR and DEPT spectra of compound 5 (Recorded in CDCl3).
**Supporting Figure 45**: HSQC spectrum of compound 5 (Recorded in CDCl3).
**Supporting Figure 46**: HMBC spectrum of compound 5 (Recorded in CDCl3).
**Supporting Figure 47**: 1H–1H COSY spectrum of compound 5 (Recorded in CDCl3).
**Supporting Figure 48**: NOESY spectrum of compound 5 (Recorded in CDCl3).
**Supporting Figure 49**: HRESIMS spectrum of compound 5.
**Supporting Figure 50**: UV spectrum of compound 5.
**Supporting Figure 51**: IR spectrum of compound 5.
**Supporting Figure 52**: 1H NMR spectrum of compound 6 (Recorded in CDCl3).
**Supporting Figure 53**: 13C NMR and DEPT spectra of compound 6 (Recorded in CDCl3).
**Supporting Figure 54**: HSQC spectrum of compound 6 (Recorded in CDCl3).
**Supporting Figure 55**: HMBC spectrum of compound 6 (Recorded in CDCl3).
**Supporting Figure 56**: 1H–1H COSY spectrum of compound 6 (Recorded in CDCl3).
**Supporting Figure 57**: NOESY spectrum of compound 6 (Recorded in CDCl3).
**Supporting Figure 58**: HRESIMS spectrum of compound 6.
**Supporting Figure 59**: UV spectrum of compound 6.
**Supporting Figure 60**: IR spectrum of compound 6.
**Supporting Figure 61**: 1H NMR spectrum of compound 10 (Recorded in CDCl3).
**Supporting Figure 62**: 13C NMR and DEPT spectra of compound 10 (Recorded in CDCl3).
**Supporting Figure 63**: HSQC spectrum of compound 10 (Recorded in CDCl3).
**Supporting Figure 64**: HMBC spectrum of compound 10 (Recorded in CDCl3).
**Supporting Figure 65**: 1H–1H COSY spectrum of compound 10 (Recorded in CDCl3).
**Supporting Figure 66**: NOESY spectrum of compound 10 (Recorded in CDCl3).
**Supporting Figure 67**: HRESIMS spectrum of compound 10.
**Supporting Figure 68**: UV spectrum of compound 10.
**Supporting Figure 69**: IR spectrum of compound 10.
**Supporting Figure 70**: 1H NMR spectrum of compound 11 (Recorded in CD3OD).
**Supporting Figure 71**: 13C NMR and DEPT spectra of compound 11 (Recorded in CD3OD).
**Supporting Figure 72**: HSQC spectrum of compound 11 (Recorded in CD3OD).
**Supporting Figure 73**: HMBC spectrum of compound 11 (Recorded in CD3OD).
**Supporting Figure 74**: 1H–1H COSY spectrum of compound 11 (Recorded in CD3OD).
**Supporting Figure 75**: NOESY spectrum of compound 11 (Recorded in CD3OD).
**Supporting Figure 76**: HRESIMS spectrum of compound 11.
**Supporting Figure 77**: UV spectrum of compound 11.
**Supporting Figure 78**: IR spectrum of compound 11.
**Supporting Figure 79**: 1H NMR spectrum of compound 12 (Recorded in CDCl3).
**Supporting Figure 80**: 13C NMR and DEPT spectra of compound 12 (Recorded in CDCl3).
**Supporting Figure 81**: HSQC spectrum of compound 12 (Recorded in CDCl3).
**Supporting Figure 82**: HMBC spectrum of compound 12 (Recorded in CDCl3).
**Supporting Figure 83**: 1H–1H COSY spectrum of compound 12 (Recorded in CDCl3).
**Supporting Figure 84**: NOESY spectrum of compound 12 (Recorded in CDCl3).
**Supporting Figure 85**: HRESIMS spectrum of compound 12.
**Supporting Figure 86**: UV spectrum of compound 12.
**Supporting Figure 87**: IR spectrum of compound 12.
**Supporting Figure 88**: 1H NMR spectrum of compound 13 (Recorded in CD3OD).
**Supporting Figure 89**: 13C NMR and DEPT spectra of compound 13 (Recorded in CD3OD).
**Supporting Figure 90**: HSQC spectrum of compound 13 (Recorded in CD3OD).
**Supporting Figure 91**: HMBC spectrum of compound 13 (Recorded in CD3OD).
**Supporting Figure 92**: 1H–1H COSY spectrum of compound 13 (Recorded in CD3OD).
**Supporting Figure 93**: NOESY spectrum of compound 13 (Recorded in CD3OD).
**Supporting Figure 94**: HRESIMS spectrum of compound 13.
**Supporting Figure 95**: UV spectrum of compound 13.
**Supporting Figure 96**: IR spectrum of compound 13.
**Supporting Table 1**: 1H NMR spectroscopic data (*δ* in ppm, *J* in Hz) for compounds 1, 3–6, and 11–13.
**Supporting Table 2**: 13C NMR spectroscopic data (*δ* in ppm) for compounds 1, 3–6, 11–13.
**Supporting Table 3**: Gibbs free energiesa and equilibrium populationsb of low‐energy conformers of 11.
**Supporting Table 4**: Cartesian coordinates for the low‐energy reoptimized random research conformers of 11 at B3LYP‐D3(BJ)/6‐31G* level of theory in methanol.
**Supporting Table 5**: Gibbs free energiesa and equilibrium populationsb of low‐energy conformers of 1a.
**Supporting Table 6**: Cartesian coordinates for the low‐energy reoptimized random research conformers of 1a at B3LYP‐D3(BJ)/6‐31G* level of theory in methanol.
**Supporting Table 7**: Gibbs free energiesa and equilibrium populationsb of low‐energy conformers of 1b.
**Supporting Table 8**: Cartesian coordinates for the low‐energy reoptimized random research conformers of 1b at B3LYP‐D3(BJ)/6‐31G* level of theory in methanol.
**Supporting Table 9**: Gibbs free energiesa and equilibrium populationsb of low‐energy conformers of 3a.
**Supporting Table 10**: Cartesian coordinates for the low‐energy reoptimized random research conformers of 3a at B3LYP‐D3(BJ)/6‐31G* level of theory in chloroform.
**Supporting Table 11**: Gibbs free energiesa and equilibrium populationsb of low‐energy conformers of 3b.
**Supporting Table 12**: Cartesian coordinates for the low‐energy reoptimized random research conformers of 3b at B3LYP‐D3(BJ)/6‐31G* level of theory in chloroform.
**Supporting Table 13**: Gibbs free energiesa and equilibrium populationsb of low‐energy conformers of 11a.
**Supporting Table 14**: Cartesian coordinates for the low‐energy reoptimized random research conformers of 11a at B3LYP‐D3(BJ)/6‐31G* level of theory in methanol.
**Supporting Table 15**: Gibbs free energiesa and equilibrium populationsb of low‐energy conformers of 11b.
**Supporting Table 16**: Cartesian coordinates for the low‐energy reoptimized random research conformers of 11b at B3LYP‐D3(BJ)/6‐31G* level of theory in methanol.
**Supporting Table 17**: Gibbs free energiesa and equilibrium populationsb of low‐energy conformers of 12a.
**Supporting Table 18**: Cartesian coordinates for the low‐energy reoptimized random research conformers of 12a at B3LYP‐D3(BJ)/6‐31G* level of theory in chloroform.
**Supporting Table 19**: Gibbs free energiesa and equilibrium populationsb of low‐energy conformers of 12b.
**Supporting Table 20**: Cartesian coordinates for the low‐energy reoptimized random research conformers of 12b at B3LYP‐D3(BJ)/6‐31G* level of theory in chloroform.
**Supporting Table 21**: Gibbs free energiesa and equilibrium populationsb of low‐energy conformers of 13a.
**Supporting Table 22**: Cartesian coordinates for the low‐energy reoptimized random research conformers of 13a at B3LYP‐D3(BJ)/6‐31G* level of theory in methanol.
**Supporting Table 23**: Gibbs free energiesa and equilibrium populationsb of low‐energy conformers of 13b.
**Supporting Table 24**: Cartesian coordinates for the low‐energy reoptimized random research conformers of 13b at B3LYP‐D3(BJ)/6‐31G* level of theory in methanol.

## Data Availability

The bulk RNA‐Seq and scRNA‐Seq data in this study were deposited in the National Genomics Data Center (NGDC) (https://ngdc.cncb.ac.cn), accession ID PRJCA036583 and PRJCA036557. Values for data points in figures are reported in the Supporting Data Values file. Other data and materials are available upon reasonable request.
