# Supplementary material for: Emestrin‐Type Epidithiodiketopiperazines Inhibited Gasdermin D‐Mediated Pyroptosis via Caspase‐3/7 Activation
Source: MedComm (2020). 2026 Jan 13;7(1):e70548. doi: 10.1002/mco2.70548 (PMC12796841; doi:10.1002/mco2.70548)
Supplement: Supplementary file 1 — Supporting Figure 1: High content screening of 536 compounds. (A) YO‐PRO‐1 staining results of HCS. (B) Individual pictures for indicated treatments. Supporting Figure 2: Selected 1H–1H COSY (blue bold lines) and HMBC (red arrows) correlations ofcompounds 1, 3–6, and 10–13. Supporting Figure 3: Selected NOESY/ROESY correlations of compounds 1, 3, 4, 6, and 10–13. Supporting Figure 4: X‐Ray crystallographic structures of 3–6. Supporting Figure 5: CD curves of compounds. (A) Experimental ECD curves of compounds 1, 4–6, and 13. (B) Experimental ECD curves of compounds 3 and 12. (C) Experimental ECD curves of compounds 10–11 and calculated ECD curve of 11. Supporting Figure 6: NMR calculations with DP4+ probability analysis for 1, 3, and 10. (A) Linear correlation plots of experimental vs. calculated 1H NMR (left) and 13C NMR (right) chemical shifts of 1. (B) Linear correlation plots of experimental vs. calculated 1H NMR (left) and 13C NMR (right) chemical shifts of 3. (C) Linear correlation plots of experimental vs. calculated 1H NMR (left) and 13C NMR (right) chemical shifts of 10. (D) DP4+ probability analysis. Supporting Figure 7: NMR calculations with DP4+ probability analysis for 11–13. (A) Linear correlation plots of experimental vs. calculated 1H NMR (left) and 13C NMR (right) chemical shifts of 11. (B) Linear correlation plots of experimental vs. calculated 1H NMR (left) and 13C NMR (right) chemical shifts of 12. (C) Linear correlation plots of experimental vs. calculated 1H NMR (left) and 13C NMR (right) chemical shifts of 13. (D) DP4+ probability analysis. Supporting Figure 8: Mutagenesis analysis of pro‐caspase‐3 in HEK293T cells.(A) Western blotting analysis of HEK293T cells overexpressing wild type or different point mutation pro‐caspase‐3 protein treated with compound 2 (1 µM). (B) A predicted binding site of compound 2 in pro‐caspase‐3 protein is conserved across the indicated species. Supporting Figure 9: Compound 2 protected against LPS‐induced sepsis [file MCO2-7-e70548-s001.pdf]

Supporting Information

Emestrin-Type Epidithiodiketopiperazines Inhibited Gasdermin D-Mediated Pyroptosis via Caspase-3/7 Activation

Bingchuan Geng[1,2,3,4,#], Shuang Lin[1,5,#], Wai Yen Yim[4,#], Weiguang Sun[1,#], Xiaotian Zhang[1], Cao Ma[2], Zhiwen Zhang[2], Quan Guo[2], Jie Gao[1], Hanxiao Zeng[1], Qingyi Tong[1], Yixuan Wang[4], Zhengfeng Fan[4], Jincheng Hou[4], Muwei Li[2,3,***], Yonghui Zhang[1,**], Zhengxi Hu[1,6,7,*]

[1]*Hubei Key Laboratory of Natural Medicinal Chemistry and Resource Evaluation, School of Pharmacy, Tongji Medical College, Huazhong University of Science and Technology, Wuhan, China*
[2]*Fuwai Central China Cardiovascular Hospital, Central China Fuwai Hospital of Zhengzhou University, Zhengzhou, China*
[3]*Henan Provincial Clinical Research Center for Cardiovascular Disease, Zhengzhou, China*
[4]*Department of Cardiovascular Surgery, Union Hospital, Tongji Medical College, Huazhong University of Science and Technology, Wuhan, China*
[5]*Institute of Pharmaceutical Process, Hubei Province Key Laboratory of Occupational Hazard Identification and Control, School of Medicine, Wuhan University of Science and Technology, Wuhan, China*
[6]*Hubei Shizhen Laboratory, Wuhan, China*
[7]*Hubei Jiangxia Laboratory, Wuhan, China*

[*]Corresponding author.

[**]Corresponding author.

[***]Corresponding author.

E-mail addresses: hzx616@126.com (Z. Hu), Tel./fax: +86-027-83692892; zhangyh@mails.tjmu.edu.cn (Y. Zhang); lmwei0207@zzu.edu.cn (M. Li).

[#]These authors have contributed equally to this work.

Contents	Pages
**Materials and Methods**	3
**Supplementary Text**	14
**References**	18
**Figures S1 to S96**	22
**Table S1 to S24**	83

Materials and Methods

General Experimental Procedures

HRESIMS data were acquired with a Bruker MicrOTOF II spectrometer in the positive ion mode. UV spectra were recorded with a PerkinElmer Lambda 35 spectrophotometer. Optical rotations were recorded in MeOH on a Perkin-Elmer 341 polarimeter. IR spectra were performed on a Tenor 27 FT-IR spectrometer with KBr pellets. ECD data were collected from a JASCO-810 CD spectrometer. The 1D and 2D NMR data were measured with a Bruker AM-400 or AM-600 NMR spectrometer, and the ^{1}H and ^{13}C NMR chemical shifts were referenced to the solvent signals for $CDCl_3$ (δ_H 7.26/δ_C 77.2) and CD_3OD (δ_H 3.31/δ_C 49.0). Column chromatography was performed with silica gel (100–200 and 200–300 mesh, Qingdao Marine Chemical Inc., Qingdao, China), Sephadex LH-20 (GE Healthcare Bio-Sciences AB, Sweden), and ODS (50 μm, YMC, Tokyo, Japan). Semipreparative HPLC was performed on an Agilent 1200 liquid chromatograph using an RP-C_{18} column (5 μm, 10 × 250 mm, Welch Ultimate XB-C_{18}). Thin-layer chromatography (TLC) was performed with silica gel 60 F_{254} on glass plates (Yantai Chemical Industry Research Institute), and spots were observed by heating silica gel plates sprayed with 10% H_2SO_4 in EtOH.

Fungal Material

The strain *Aspergillus* sp. TJ403-G07 was isolated from grasshopper, which was collected from Wuhan City of Hubei Province, P. R. China. This strain was identified by morphology and sequence analysis of the ITS region of rDNA (GenBank No. ON026863). The fungal strain was deposited in the culture collection of college of pharmacy, Tongji Medical College, Huazhong University of Science and Technology.

Extraction and Isolation

The strain *Aspergillus* sp. TJ403-G07 was cultivated on potato dextrose agar (PDA) plates at 28 °C

for 5 days. Then the agar plugs were cut into small pieces and inoculated into 400 sterilized erlenmeyer flasks (1 L), each containing 250 g of rice and 250 mL of distilled water. After incubation at 28 °C for 28 days, the growth of cells was stopped by infusing 300 mL of EtOH to each flask for 24 h. The solvent was poured out and concentrated by a rotatory evaporator. The cultures were soaked repeatedly with the recycled EtOH until the solvent extract was almost colorless. The dry extract was suspended in water (2 L) and extracted with EtOAc (1:1, v/v) ten times. The organic solvent was evaporated under reduced pressure to afford the crude extract (500 g), which was then subjected to a silica gel CC progressively eluted with PE–EtOAc (20:1−0:1, v/v) to obtain five major fractions (A–E). Fraction C (PE–EtOAc, 1:1, v/v) was fractionated by RP-C_{18} CC eluted with MeOH–H_2O (20:80–100:0, v/v) to obtain three subfractions (C1–C3).

Fraction C1 was separated into five major fractions (C1-1–C1-5) using silica gel CC (PE–EtOAc, 10:1→0:1). Fraction C1-3 was applied to Sephadex LH-20 (CH_2Cl_2–MeOH, 1:1, v/v) column and semipreparative HPLC (MeOH–H_2O, 71:29, v/v) to provide compound **8** (4.5 mg, t_R = 21 min).

Fraction C2 was subjected to Sephadex LH-20 (CH_2Cl_2–MeOH, 1:1, v/v) column to give ten main subfractions (C2-1–C2-10). Then subfraction C2-1 was applied to semipreparative HPLC (MeOH–H_2O, 74:26, v/v; 2.0 mL/min) to provide compound **13** (2.3 mg, t_R = 16 min). Fraction C2-4 was subjected to RP-HPLC eluted with MeCN–H_2O (85:25, v/v, 2.0 mL/min) to afford compound **5** (2.0 mg, t_R = 13 min). Subfraction C2-5 was fractionated by semipreparative HPLC (MeOH–H_2O, 62:38, v/v; 2.0 mL/min) to obtain compounds **1** (16.7 mg, t_R = 39 min), **4** (3.0 mg, t_R = 59 min), **7** (11.0 mg, t_R = 18 min), **10** (3.8 mg, t_R = 38 min), and **11** (5.0 mg, t_R = 50 min). Subfraction C2-6 was separated by semipreparative HPLC (MeOH–H_2O, 70:30, v/v; 2.0 mL/min) to acquire compounds **3** (3.3 mg, t_R = 22 min) and **6** (3.0 mg, t_R = 40 min). Fraction C2-7 was applied to repeated RP-HPLC eluted with MeOH–H_2O (64:36, v/v, 2.0 mL/min) to obtain **9** (2.4

mg, t_R = 36 min) and **12** (1.6 mg, t_R = 25 min). Recrystallization of fraction C2-10 provided compound **2** (18.0 mg).

*Aspermestrin A (**1**)*: white amorphous powders; $[\alpha]_D^{25}$: –96 (MeOH, *c* 0.3); UV (MeOH) λ_{max} (log ε): 225 (3.83), 255 (3.57), 295 (3.20) nm; IR (KBr) ν_{max}: 3430, 2926, 2854, 1709, 1692, 1606, 1520, 1358, 1291, 1220, 1144, 1028, 899, 763, 714 cm^{-1}; For ^1H and ^{13}C NMR data, see Tables S1 and S2; (+)-HRESIMS *m/z* 561.0763 $[M + Na]^+$ (calcd for $C_{26}H_{22}N_2O_7S_2Na^+$, 561.0761).

*Aspemestrin B (**3**)*: colorless needle crystals; $[\alpha]_D^{25}$: +111 (MeOH, *c* 0.3); UV (MeOH) λ_{max} (log ε): 201 (4.46), 217 (4.31), 258 (3.92), 280 (4.62) nm; IR (KBr) ν_{max}: 3440, 2929, 2851, 1707, 1641, 1606, 1513, 1272, 1126, 1052, 1020, 813, 764, 719, 676 cm^{-1}; For ^1H and ^{13}C NMR data, see Tables S1 and S2; (+)-HRESIMS *m/z* 605.0651 $[M + Na]^+$ (calcd for $C_{27}H_{22}N_2O_9S_2Na^+$, 605.0659).

*Aspermestrin C (**4**)*: colorless needle crystals; $[\alpha]_D^{25}$: –80 (MeOH, *c* 0.3); UV (MeOH) λ_{max} (log ε): 201 (4.97), 256 (4.52) nm; IR (KBr) ν_{max}: 3426, 2934, 2848, 1710, 1692, 1606, 1514, 1356, 1258, 1173, 1102, 1026, 848, 769 cm^{-1}; For ^1H and ^{13}C NMR data, see Tables S1 and S2; (+)-HRESIMS *m/z* 575.0917 $[M + Na]^+$ (calcd for $C_{27}H_{24}N_2O_7S_2Na^+$, 575.0917).

*Aspermestrin D (**5**)*: colorless needle crystals; $[\alpha]_D^{25}$: +55 (MeOH, *c* 0.3); UV (MeOH) λ_{max} (log ε): 216 (4.52), 260 (4.18), 292 (3.93) nm; IR (KBr) ν_{max}: 3428, 2920, 2849, 1706, 1614, 1590, 1511, 1358, 1279, 1214, 1125, 1024, 890, 760, 711 cm^{-1}; For ^1H and ^{13}C NMR data, see Tables S1 and S2; (+)-HRESIMS *m/z* 559.0960 $[M + Na]^+$ (calcd for $C_{27}H_{24}N_2O_6S_2Na^+$, 559.0968).

*Aspermestrin E (**6**)*: colorless needle crystals; $[\alpha]_D^{25}$: +93 (MeOH, *c* 0.3); UV (MeOH) λ_{max} (log ε): 225 (4.54), 260 (4.23), 283 (3.92) nm; IR (KBr) ν_{max}: 3444, 2925, 2852, 1712, 1667, 1608, 1515, 1345, 1273, 1202, 1127, 1023, 897, 812, 763 cm^{-1}; For ^1H and ^{13}C NMR data, see Tables S1 and S2; (+)-HRESIMS *m/z* 605.0672 $[M + Na]^+$ (calcd for $C_{27}H_{22}N_2O_9S_2Na^+$, 605.0659).

*Aspermestrin E (**10**)*: white amorphous powders; $[\alpha]_D^{25}$: –86 (MeOH, *c* 0.3); UV (MeOH) λ_{max}

(log ε): 201 (4.59), 216 (4.52), 260 (4.06), 298 (3.84) nm; IR (KBr) ν_{max}: 3504, 2925, 2850, 1708, 1680, 1613, 1375, 1280, 1214, 1139, 1024, 764, 715 cm^{-1}; For ^{1}H and ^{13}C NMR data, see Tables S1 and S2; (+)-HRESIMS *m/z* 639.0352 [M + Na]$^+$ (calcd for $C_{27}H_{24}N_2O_7S_4Na^+$, 639.0359).

*Aspermestrin G (**11**)*: white amorphous powders; $[\alpha]_D^{25}$: –130 (MeOH, *c* 0.3); UV (MeOH) λ_{max} (log ε): 200 (4.42), 218 (4.15), 263 (3.68), 300 (3.44) nm; IR (KBr) ν_{max}: 3445, 2928, 2853, 1681, 1608, 1382, 1289, 1222, 1141, 1025, 765, 714 cm^{-1}; For ^{1}H and ^{13}C NMR data, see Tables S1 and S2; (+)-HRESIMS *m/z* 625.0195 [M + Na]$^+$ (calcd for $C_{26}H_{22}N_2O_7S_4Na^+$, 625.0202).

*Aspermestrin H (**12**)*: light yellow amorphous powders; $[\alpha]_D^{25}$: +45 (MeOH, *c* 0.3); UV (MeOH) λ_{max} (log ε): 201 (4.68), 217 (4.53), 258 (4.22), 280 (4.01) nm; IR (KBr) ν_{max}: 3445, 2931, 2855, 1694, 1606, 1514, 1273, 1129, 1049, 1019, 816, 764, 707, 681 cm^{-1}; For ^{1}H and ^{13}C NMR data, see Tables S1 and S2; (+)-HRESIMS *m/z* 621.0609 [M + Na]$^+$ (calcd for $C_{27}H_{22}N_2O_{10}S_2Na^+$, 621.0608).

*Aspermestrin I (**13**)*: white amorphous powders; $[\alpha]_D^{25}$: –94 (MeOH, *c* 0.3); UV (MeOH) λ_{max} (log ε): 200 (4.66), 256 (4.18) nm; IR (KBr) ν_{max}: 3419, 2925, 2852, 1710, 1691, 1609, 1515, 1358, 1271, 1169, 1145, 1022, 854, 771, 729 cm^{-1}; For ^{1}H and ^{13}C NMR data, see Tables S1 and S2; (+)-HRESIMS *m/z* 561.0762 [M + Na]$^+$ (calcd for $C_{26}H_{22}N_2O_7S_2Na^+$, 561.0761).

NMR Calculations Details

Conformational analyses were carried out via random searching in the Sybyl-X 2.0 using the MMFF94S force field with an energy cutoff of 5.0 kcal/mol. The results showed four lowest energy conformers. Subsequently, the conformers were re-optimized at the B3LYP-D3(BJ)/6-31G* level in PCM chloroform by the Gaussian 09 program [1]. All conformers used for property calculations in this work were characterized to be stable point on potential energy surface (PES) with no imaginary frequencies. NMR shielding constants were computed using the GIAO method at the

mPW1PW91/6-311+G** level in PCM chloroform by the Gaussian 09 program [1]. Gibbs free energies for conformers were determined by using thermal correction at B3LYP-D3(BJ)/6-31G* level and electronic energies evaluated at the wB97M-V/def2-TZVP level in PCM chloroform using ORCA [2, 3]. Boltzmann weights were computed using relative Gibbs free energies [4]. The unscaled chemical shifts (δ_u) were computed using TMS (Tetramethylsilane) as a reference standard according to $\delta_u = \sigma_0 - \sigma_x$, where σ_x is the Boltzmann averaged shielding tensor (over all significantly populated conformations) and σ_0 is the shielding tensor of the TMS computed at the same level of theory employed for σ_x. The scaled chemical shifts (δ_s) were calculated as $\delta_s = (\delta_u - b)/m$, where m and b are the slope and intercept, respectively, deduced from a linear regression calculation on a plot of δ_u against δ_{exp}. The DP4+ calculations were run by the Excel spreadsheet available for free at sarotti-nmr.weebly.com or as part of the Supporting Information of the original paper [4].

ECD Calculation

Conformational analyses were performed via random searching using the MMFF94S force field with an energy cutoff of 5 kcal/mol in the Sybyl-X 2.0. The results showed five lowest energy conformers of compound **11**. Subsequently, geometry optimizations and frequency analyses were carried out at the B3LYP-D3(BJ)/6-31G* level in CPCM methanol using ORCA5.0.1 [2, 3]. All conformers used for property calculations in this work were characterized to be stable point on potential energy surface (PES) with no imaginary frequencies. The excitation energies, oscillator strengths, and rotational strengths (velocity) of the first 60 excited states were calculated using the TD-DFT methodology at the CAM-B3LYP/def2-TZVP level in CPCM methanol using ORCA5.0.1 [2, 3]. The ECD spectra were simulated via the overlapping Gaussian function (half the bandwidth at 1/*e* peak height, sigma = 0.30 for all) [5]. Gibbs free energies for conformers

were determined by using thermal correction at B3LYP-D3(BJ)/6-31G* level and electronic energies evaluated at the wB97M-V/def2-TZVP level in CPCM methanol using ORCA5.0.1 [1]. To get the final spectra, the simulated spectra of the conformers were averaged according to the Boltzmann distribution theory and their relative Gibbs free energy (ΔG). The absolute configuration of compound **11** was determined by comparing the experiment spectra with the calculated model molecules.

X-Ray Crystallographic Analyses

Colorless crystals of compounds **3**–**6** were obtained from the mixed solvent CH_2Cl_2–MeOH (10:1), by slow volatilization at room temperature. The single-crystal X-ray diffraction data were collected by using graphite-monochromated Cu Kα radiation on a Bruker APEX DUO diffractometer equipped with an APEX II CCD. Cell refinement and data reduction were achieved with Bruker SAINT. The structures were solved by direct methods using SHELXS-97 [6], expanded using Fourier difference techniques, and refined by the program and full-matrix least-squares calculations. All non-hydrogen atoms were refined anisotropically, and the hydrogen atoms were put on the calculated positions. Crystallographic data for the reported structures have been deposited in the Cambridge Crystallographic Data Center with deposition numbers CCDC 2145165 for **3**, CCDC 2145169 for **4** and CCDC 2145200 for **5**, and CCDC 2145168 for **6**. Copies of the data can be obtained free of charge from the CCDC, 12 Union Road, Cambridge CB 1EZ, UK [fax: Int. +44(0) (1223) 336 033); e-mail: deposit@ccdc.cam.ac.uk].

Crystallographic Data for Compound ***3***: $C_{27}H_{22}N_2O_9S_2$•CH_4O, M = 614.63, a = 6.06050(10) Å, b = 14.1461(3) Å, c = 31.5994(8) Å, α = 90°, β = 90°, γ = 90°, V = 2709.09(10) Å^3, T = 100(2) K, space group $P2_12_12_1$, Z = 4, μ(Cu Kα) = 2.342 mm^{-1}, 25295 reflections measured, 5317 independent reflections (R_{int} = 0.0599). The final R_1 values were 0.0407 ($I > 2\sigma(I)$). The final $wR(F^2)$

values were 0.1068 ($I > 2\sigma(I)$). The final R_1 values were 0.0437 (all data). The final $wR(F^2)$ values were 0.1086 (all data). The goodness of fit on F^2 was 1.030. Flack parameter = 0.035(6).

*Crystallographic Data for Compound **4***: $C_{27}H_{24}N_2O_7S_2$, M = 552.60, a = 9.2743(2) Å, b = 14.0039(4) Å, c = 19.2906(5) Å, α = 90°, β = 98.6110(10)°, γ = 90°, V = 2477.15(11) Å^3, T = 100(2) K, space group P1211, Z = 4, μ(Cu Kα) = 2.399 mm^{-1}, 52200 reflections measured, 9674 independent reflections (R_{int} = 0.0565). The final R_1 values were 0.0371 ($I > 2\sigma(I)$). The final $wR(F^2)$ values were 0.0996 ($I > 2\sigma(I)$). The final R_1 values were 0.0380 (all data). The final $wR(F^2)$ values were 0.1005 (all data). The goodness of fit on F^2 was 1.031. Flack parameter = 0.080(6).

*Crystallographic Data for Compound **5***: $C_{27}H_{24}N_2O_6S_2$, M = 536.60, a = 6.277 Å, b = 17.48810(10) Å, c = 22.00790(10) Å, α = 90°, β = 90°, γ = 90°, V = 2415.869(18) Å^3, T = 293(2) K, space group $P2_12_12_1$, Z = 4, μ(Cu Kα) = 2.410 mm^{-1}, 25575 reflections measured, 4838 independent reflections (R_{int} = 0.0375). The final R_1 values were 0.0295 ($I > 2\sigma(I)$). The final $wR(F^2)$ values were 0.0755 ($I > 2\sigma(I)$). The final R_1 values were 0.0300 (all data). The final $wR(F^2)$ values were 0.0758 (all data). The goodness of fit on F^2 was 1.027. Flack parameter = 0.024(6).

*Crystallographic Data for Compound **6***: $C_{27}H_{22}N_2O_9S_2$, M = 582.58, a = 7.8617(2) Å, b = 13.8151(4) Å, c = 22.1011(6) Å, α = 90°, β = 90°, γ = 90°, V = 2400.40(11) Å^3, T = 118(2) K, space group $P2_12_12_1$, Z = 4, μ(Cu Kα) = 2.577 mm^{-1}, 24869 reflections measured, 4728 independent reflections (R_{int} = 0.0575). The final R_1 values were 0.0283 ($I > 2\sigma(I)$). The final $wR(F^2)$ values were 0.0714 ($I > 2\sigma(I)$). The final R_1 values were 0.0313 (all data). The final $wR(F^2)$ values were 0.0733 (all data). The goodness of fit on F^2 was 1.050. Flack parameter = 0.031(6).

Single Cell Full-Length RNA Sequence Transcriptome-Seq (scFAST-seq) library Construction and Sequencing

scFAST-seq library were prepared using SeekOne® Single Cell Whole Trancriptome Kit according

to manufacturer's instructions (SeekGene Catalog No. K00801). Briefly, an appropriate number of cells were mixed with reverse transcription reagents and added to the sample wells of the SeekOne® DD Chip S3 (Chip S3). Then, Barcoded Hydrogel Beads (BHBs) and partitioning oil were dispensed into corresponding wells separately in Chip S3. Subsequently, Cell-containing reverse transcription reagents and BHBs were encapsulated into emulsion droplets using SeekOne® Digital Droplet System. Immediately following transferring emulsion droplets into PCR tubes, fifteen cycles of annealing (ramping from 8 °C to 42 °C) followed by a 5-min heat inactivation at 85 °C were performed to obtain barcoded cDNA. Next, the barcoded cDNA was purified from broken droplet and then two PCR reactions were performed to remove the majority of ribosomal and mitochondrial cDNA. AMPure beads were used to purify cDNA from the post PCR reaction mixture. Finally, one forth volume of cDNA was fragmented, end repaired, A-tailed and ligated into sequencing adaptor. DNA amplified by index PCR contains any part of polyA or non-PolyA RNA as well as Cell Barcode and Unique Molecular Index. The indexed sequencing libraries were purified using AMPure beads and quantified by quantitative PCR (KAPA Biosystems KK4824). The libraries were then sequenced on Illumina NovaSeq 6000 with PE150 read length or DNBSEQ-T7 platform with PE150 read length.

Sequencing Data Quality Control

Fastp (v0.20.1) was used to trim primer sequence and low-quality bases of raw reads and collect the basic statistics (https://doi.org/10.1002/imt2.107). The specific parameters could be summarized as below: 1) A 1 bp sliding window was moved from tail (3') to front. The bases in the window were dropped if its mean quality was below 3, and the window kept moving until the last base. The trailing N bases were also trimmed, similar to the Trimmomatic TRAILING method (--cut_tail --cut_tail_window_size 1--cut_tail_mean_quality 3). 2) The auto adapter was detected

for PE data (detect_adapter_for_pe). 3) The trimmed Reads shorter than 60 bp were discarded (--length_required 60). The cleaned reads after trimmed were used in the following steps.

Processing the Single Cell Full-Length RNA Sequencing Data

We used the SeekSoul Tools pipeline to process the cleaned reads and generate the transcript expression matrix. Firstly, the cell barcodes and UMI sequences were extracted based on the defined pattern about the localization of the barcode, linker and UMI within a read. The barcode was corrected with whitelist. The corrected barcode, together with UMI, were put in the header of their corresponding reads. Secondly, the reads were mapped to the reference genomes using STAR 2.5 [7], and the reads with barcode and UMI information were assigned to transcriptome using featureCounts of package Subread 1.6.4 [8]. Parameters "-s" and "-t" of featureCounts vary with different type of chemistries and regions. For the parameter "-s", "-s 1" was used for product with 3' chemistry and "-s 2" was used for product with 5' chemistry. Another parameter "-t exon" was used for read counting only with exon, and "-t transcript" was used for read counting with exon and intron. We also set the parameter "-fracOverlap" to 0.5. Other parameters remained as default. Finally, similar to the raw_feature_bc_matrix results of CellRanger [9], the raw UMI count matrix was generated according to barcodes and transcripts. A cell-calling algorithm was used to filter the raw UMI count matrix and get the cell only filtered_feature_bc_matrix. The algorithm was similar to that of CellRanger and EmptyDrops [10], which had two key steps: 1) It used a cutoff based on total UMI counts of each barcode to identify cells. This step identified the primary mode of the high RNA content cells. 2) The algorithm then used the RNA profile of each remaining barcode to determine if it is an "empty" or a cell containing partition. This step captured the low RNA content cells which total UMI counts might be similar to the empty well

scRNA-Seq Clustering and Cell Type Annotation

The resulting gene-by-cell matrices were transformed into Seurat objects (Seurat v5.0) [11], and downstream analysis was conducted in R v4.3 unless otherwise stated. High-confidence doublets were removed using the DoubletFinder package with the pK parameter optimized for each sample individually [12]. Subsequently, all Seurat objects were merged and filtered to exclude cells with < 1000 and > 6000 number of expressed genes, or with > 10% of reads mapping to mitochondrial RNA. The remaining cells were normalized and scaled using the built-in function in Seurat. 2000 highly variable genes were then used to construct Principal Components (PCs). PCs covering the highest variance in the dataset were selected based on elbow plots and heatmaps and used as input for graph-based clustering. Clusters were calculated using the FindNeighbours and FindClusters functions with the resolution parameter set to 0.4 and visualized using the Seurat implementation of the dimensional reduction algorithm UMAP. Differential gene expression analysis was performed for the 26 resulting clusters using the Seurat FindAllMarkers function. The main immune cell types were annotated using Cluster Identity PRedictor (CIPR) R package was conducted using The Immunological Genome Project (ImmGen) as reference [13, 14].

Pseudobulk Differential Expression and Gene Ontology Enrichment Analysis

To perform differential gene expression (DGE) tests between conditions, pseudobulk count matrices were created by extracting and aggregating the UMI counts across replicates and cell types using Seurat 'aggregate expression' function and DGE was performed using R package DESeq2 [15]. Function annotations and pathway enrichment were performed using R package ClusterProfiler v4.0 using references from mouse gene ontology biological pathways gene set collections found on MSigDB [16, 17]. For comparison of enriched pathways between multiple cell types/conditions, the compareCluster 'compareCluster' function was utilized. Enriched pathways were visualized using the enrichplot 'dotplot' function.

Neighborhood-Based Differential Abundance Analysis of scRNA-seq Data

In addition to cell ratio comparison, we conducted neighborhood-based differential abundance analysis using R package Milo [18]. Milo graph was built ('buildGraph') on the top 30 PCs and with k = 20. Refined neighborhoods were identified ('makeNhoods') with 20% of the vertices (prop function = 0.2). After identifying the differentially abundant neighborhoods with 'testNhoods', those with spatial FDR < 0.2 were considered significantly different.

Monocyte to Matured Dendritic Cells Trajectory Analysis

Monocytes are able to phagocytose foreign antigen, causing them to differentiate and mature into antigen presenting dendritic cell (DC) and migrate into the lymph nodes to activate T cells [19]. We extracted 21325 cells from the monocyte to DC maturation compartment, and inferred cell-state trajectories between monocytes and activated states within each sample using slingshot 3.8.0 bioconductor release in R v4.3.1 [20]. Slingshot was run on the original UMAP space without re-clustering. The most representative trajectory was selected by following the most connected pathway across the minimum spanning tree inferred by slingshot. To identify interesting gene expression patterns along the slingshot inferred trajectories, we applied the method tradeSeq in R v4.3.1 [21]. A generalized additive model (GAM) was fit with a condition-specific smoother for 2,000 highly variable genes using fitGAM (nknots = 5) on raw counts with and without treatment of compound **2**. We looked for genes that showed differential trajectory between condition with slingshot-inferred pseudo-time using the conditionTest() in tradeSeq. Differential trajectory markers were identified as those with an FDR adjusted two-sided p-value < 0.05 from conditionTest (), log fold-change > |2|. Clustering of differential trajectory genes based on expression pattern were performed using Kmeans algorithm (k = 8) from the ComplexHeatmap package [22] and visualized using the package ClusterGVis

(https://github.com/junjunlab/ClusterGVis).

Supplementary Text

Isolation and Structural Elucidation of Thirteen Emestrin-Type ETPs from an Insect Endophytic Fungus *Aspergillus* sp. TJ403-G07

Aspermestrin A (**1**) was isolated as a white amorphous powder with the molecular formula $C_{26}H_{22}N_2O_7S_2$, as deduced from the high-resolution electrospray ionization mass spectrometry (HRESIMS) analysis displaying a sodium ion peak at *m*/*z* 561.0763 (calcd for $C_{26}H_{22}N_2O_7S_2Na^+$, 561.0761), indicating 17 degrees of unsaturation. The ^{13}C NMR data along with the DEPT data of **1** (Tables S1 and S2) displayed 26 carbon resonances consisting of one *N*-methyl, two sp^3 methylenes, 13 methines (including 11 aromatic/olefinic), and 10 nonprotonated carbons (including three carbonyls and five aromatic/olefinic). These data, compared with those of emestrin J (**7**) [23], revealed that **1** was an emestrin-type derivative, featuring the same epidithiodioxopiperazine skeleton. The only difference between compound **1** and **7** was that the C-4′ methoxy group in **7** was replaced by the hydroxy group in compound **1**, as supported by the disappeared NMR signals of the methoxy group and HRESIMS data. Detailed HSQC, ^{1}H–^{1}H COSY, and HMBC spectroscopic analyses (Figure S2) were further undertaken to confirm the gross structure of compound **1**.

Aspermestrin B (**3**) and aspermestrin E (**6**) shared the same molecular formula $C_{27}H_{22}N_2O_9S_2$, as concluded via their HRESIMS data. Analysis of the ^{1}H and ^{13}C NMR data (Tables S1 and S2) suggested that compound **3** was a structural analogue of the known compound MPC1001C [23]. However, the presence of 2″-OH (δ_H 6.72) indicated that the 2″-OCH_3 in MPC1001C was demethylated in compound **3**, which could be further verified by the HMBC correlations from 2″-OH to C-1″/C-2″/C-3″ (Figure S2). The same planar structure of compounds **6** with **3** was

determined by detailed interpretation of its ^1H–^1H COSY and HMBC experiments (Figure S2).

Aspermestrin C (**4**) was isolated as a colorless crystal with a molecular formula of $C_{27}H_{24}N_2O_7S_2$ deduced by the (+)-HRESIMS data. Detailed analysis of the ^1H and ^{13}C NMR data (Tables S1 and S2) suggested that **4** was a structural analogue of secoemestrin D (**8**) [24], and the main difference was that the OH-3′ was reduced to H-3′ (δ_H 6.95) in compound **4**. The above deduction was confirmed by the ^1H–^1H COSY correlation of H-2′ (δ_H 8.08) with H-3′ and the HMBC correlations (Figure S2) from H-3′ to C-1′ (δ_C 122.4)/C-4′ (δ_C 163.6). In addition, compound **4** only had two sulfur atoms by analysis of its HRESIMS data. The 1D and 2D NMR data (Tables S1 and S2) of aspermestrin I (**13**) highly resembled those of compound **4**, and the main difference was that 4″-OCH_3 in **4** was changed to 4″-OH in compound **13**. The above deduction was verified by the HRESIMS data and the ^{13}C NMR chemical shifts (Tables S1 and S2), from C-2′/6′ (δ_C 132.6)–C-3′/5′ (δ_C 113.7) in **4** to C-2′/6′ (δ_C 133.8)–C-3′/5′ (δ_C 116.1) in **13**. Thus, the planar structures of compounds **4** and **13** were ascertained.

The HRESIMS data of aspermestrin D (**5**) pinpointed that its molecular formula was $C_{27}H_{24}N_2O_6S_2$, suggesting 17 indices of hydrogen deficiency. Interpretation of its 1D and 2D NMR data (Tables S1 and S2) suggested that **5** maintained the similar framework with emestrin K [23]. The evident difference was that a disulfide bond was formed between C-3 and C-11a in compound **5**, which coincided with 30 mass units (C_2H_6) less than emestrin K and the absence of two *S*-methyl signals in **5**. Fortunately, a suitable crystal of compound **5** was obtained and subjected to the single-crystal X-ray crystallographic data analysis [Flack parameter = 0.024(6)] (Figure S4), which enabled us to determine its absolute structure.

Aspermestrin F (**10**) was isolated as a white, amorphous powder. Detailed interpretation of its 1D and 2D NMR data (Tables S1 and S2) proposed that compound **10** maintained the same structural features with emestrin J (**7**) [23], and the extra 64 mass units in the HRESIMS spectrum of **10** indicated two more sulfur atoms than **7**. The above conclusion was further supported by the deshielded chemical shifts of C-1 (δ_C 168.4), C-3 (δ_C 78.7), C-4 (δ_C 164.1), and C-11a (δ_C 74.5) in compound **10**. Aspermestrin G (**11**) had a molecular formula of $C_{26}H_{22}N_2O_7S_4$, as designated by the HRESIMS and ^{13}C NMR data. The 1D and 2D NMR data (Tables S1 and S2) of compound **11** were very similar to those of **10**, and the only difference was that the resonance signal of 4′-OCH_3 was absent in **11**. Accordingly, the planar constructions of compounds **10** and **11** were corroborated.

Aspermestrin H (**12**) gave the molecular formula $C_{27}H_{22}N_2O_{10}S_2$, based upon the ^{13}C NMR data as well as the HRESIMS analysis. Comparison of the 1D NMR data (Tables S1 and S2) of compounds **12** and **3** revealed that both substances shared similar structural features, and the major difference was that the methylene at C-7″ (δ_C 36.6) in **3** was replaced by a hydroxylated methine (δ_C 75.7) in **12** (Figure 2A). This conclusion was further supported by the key HMBC correlations of H-7″ (δ_H 5.42) with C-3 (δ_C 82.8)/C-4 (δ_C 166.1)/C-5″ (δ_C 128.8)/C-6″ (δ_C 125.0) (Figure S2).

The relative configurations of all new compounds **1**, **3**–**6**, and **10**–**13** were assigned by analysis of the NOESY/ROESY data, coupling constants, and quantum chemical NMR calculation with DP4+ probability analysis. Their absolute configurations were established via the calculated and experimental ECD data analyses and single-crystal X-ray diffraction (Cu Kα). Suitable crystals of compounds **3**–**6** were obtained from CH_2Cl_2–MeOH (10:1), and then subjected to the single-crystal X-ray diffraction experiments using Cu Kα irradiation (Figure S4), which unveiled the

absolute configurations of compounds **3**–**6** to be 3*S*,5a*S*,6*S*,11*R*,11a*S*,$\Delta^{7,8}$*Z*,$\Delta^{10,10a}$*E* (**3**), 3*R*,5a*S*,6*S*,11a*R*,$\Delta^{7,8}$*Z*,$\Delta^{10,10a}$*Z* (**4**), 3*R*,5a*S*,6*S*,11a*R*,$\Delta^{7,8}$*Z*,$\Delta^{10,10a}$*Z* (**5**), and 3*R*,5a*S*,6*S*,11*R*,11a*R*,7″*S*,$\Delta^{7,8}$*Z*,$\Delta^{10,10a}$*E* (**6**), respectively.

The relative configuration of the dihydrooxepine moiety in compound **1** was determined to be *anti*-axial configuration by the large coupling constant ($J_{H\text{-}5a/H\text{-}6}$ = 8.9 Hz). To determine the relative configuration of disulfide bridge, the theoretical NMR calculations with DP4+ probability analyses were employed. As shown in Figure S6, the calculated ^{13}C NMR results for **1a** (R^2 = 0.9975) showed a better match with the experimental data than those of **1b** (R^2 = 0.9952). Furthermore, **1a** was assigned with the DP4+ probability of 100% (Figure S6). The above messages suggested that **1a** was the correct relative configuration for compound **1**. Meanwhile, the identical chiral centers and ECD curves (Figure S5) of **1** and **4** displayed that the absolute configuration of **1** was 3*R*,5a*S*,6*S*,11a*R*,$\Delta^{7,8}$*Z*,$\Delta^{10,10a}$*Z*.

The relative configurations of compounds **10** and **11** were confirmed by analysis of the coupling constants and ^{13}C NMR-DP4+ probability analysis, which were identical to those of **1** (Figure S6–S7). Finally, the absolute configurations of compounds **10** and **11** were further confirmed to be 3*R*,5a*S*,6*S*,11a*R*,$\Delta^{7,8}$*Z*,$\Delta^{10,10a}$*Z* by comparing their experimental ECD curve with calculated ECD curve of **11** (Figure S5).

Compounds **12** and **3** were shown to have identical relative configurations at C-5a, C-6 and C-11, as deduced from their similar NOESY/ROESY data analysis. The key ROESY correlation of H-7″ with *N*-CH_3 (δ_H 3.22)/H-4″ (δ_H 7.20), of 7″-OH (δ_H 4.97) with H-6″ (δ_H 8.45) can be obviously observed, but no cross-peaks of 7″-OH with *N*-CH_3/H-4″ could be observed, which implied that 7″-OH of **12** was *β*-oriented (Figure S3). Similarly, the relative configuration of the sulfur bridge of compound **12** was determined as *β*-orientation by the calculated ^{13}C NMR-DP4+

probability analysis (Figure S7). The absolute configuration of **12** was deduced as 3*S*,5a*S*,6*S*,11*R*,11a*S*,7″*S*, which was established by comparing the overlaid experimental ECD curves of compounds **12** and **3** (Figure S5).

The relative configuration of the dihydrooxepine moiety in compound **13** was partially established to be the same as its analogues via the identical coupling constants and ROESY cross-peaks. Meanwhile, the relative configuration of disulfide bridge was determined to be identical with **4** according to the theoretical NMR calculations and DP4+ probability analyses. Finally, the ECD spectra of compounds **13** and **4** showed similar Cotton effects (Figure S5), which suggested that the absolute configuration of **13** was also assigned as 3*R*,5a*S*,6*S*,11a*R*,$\Delta^{7,8}$*Z*,$\Delta^{10,10a}$*Z*.

Four known congeners were identified as emestrin (**2**) [25], emestrin J (**7**) [23], secoemestrin D (**8**) [24], and dethiosecoemestrin (**9**) [26] by comparison of their NMR and HRESIMS data with the literature.

In conclusion, nine new emestrin-type ETPs, namely aspermestrins A–I (**1**, **3**–**6**, and **10**–**13**), along with four known congeners (**2** and **7**–**9**), were obtained from an insect endophytic fungus *Aspergillus* sp. TJ403-G07. All the structures for nine new compounds were fully characterized on the basis of the extensive spectroscopic analyses, including NMR spectra, quantum chemical NMR calculation, ECD calculations, and single-crystal X-ray diffraction analysis. Structurally, compounds **3** and **12** are the first examples of emestrin-type ETPs with a *β*-oriented sulfur bridge, and the chemical shifts of C-11a in **3** (δ_C 83.2) and **12** (δ_C 82.5) are much larger than that of C-11a in **1**–**2**, **4**–**11**, and **13** (δ_C 73.1–75.9).

References

1. D.B. Pu, B.W. Du, W. Chen, et al., “Premnafulvol A: A diterpenoid with a 6/5/7/3-fused

tetracyclic core and its biosynthetically related analogues from *Premna fulva*," *Organic Letters* 20, no. 19 (2018): 6314–6317.

2. F. Neese, "The ORCA program system," *WIREs Computational Molecular Science* 2, no. 1 (2012): 73–78.

3. F. Neese, "Software update: the ORCA program system, version 4.0," *WIREs Computational Molecular Science* 8, no. 1 (2018): e1327.

4. Z. Liu, Y. Yashiroda, P. Sun, et al., "Argenteolides A and B, glycosylated polyketide–peptide hybrid macrolides from an actinomycete *Streptomyces argenteolus*," *Organic Letters* 25, no. 4 (2023): 571–575.

5. P.J. Stephens, N. Harada, "ECD cotton effect approximated by the Gaussian curve and other methods," *Chirality* 22, no. 2 (2010): 229–233.

6. S. Lin, H. Zeng, C. Wang, et al., "Discovery of novel natural cardiomyocyte protectants from a toxigenic fungus *Stachybotrys chartarum*," *Bioorganic Chemistry* 148 (2024): 107461.

7. A. Dobin, C.A. Davis, F. Schlesinger, et al., "STAR: ultrafast universal RNA-seq aligner," *Bioinformatics* 29, no. 1 (2013): 15–21.

8. Y. Liao, G.K. Smyth, W. Shi, "The Subread aligner: fast, accurate and scalable read mapping by seed-and-vote," *Nucleic Acids Research* 41, no. 10 (2013): e108.

9. G.X. Zheng, J.M. Terry, P. Belgrader, et al., "Massively parallel digital transcriptional profiling of single cells," *Nature Communications* 8 (2017): 14049.

10. A.T.L. Lun, S. Riesenfeld, T. Andrews, et al., "EmptyDrops: distinguishing cells from empty droplets in droplet-based single-cell RNA sequencing data," *Genome Biology* 20, no. 1 (2019): 63.

11. A. Butler, P. Hoffman, P. Smibert, et al., "Integrating single-cell transcriptomic data across different conditions, technologies, and species," *Nature Biotechnology* 36, no. 5 (2018): 411–420.

12. C.S. McGinnis, L.M. Murrow, Z.J. Gartner, "DoubletFinder: Doublet detection in single-cell rna sequencing data using artificial nearest neighbors," *Cell Systems* 8, no. 4 (2019): 329–337.e324.

13. H.A. Ekiz, C.J. Conley, W.Z. Stephens, et al., "CIPR: a web-based R/shiny app and R package to annotate cell clusters in single cell RNA sequencing experiments," *BMC Bioinformatics* 21, no. 1 (2020): 191.

14. T. Shay, J. Kang, "Immunological Genome Project and systems immunology," *Trends in Immunology* 34, no. 12 (2013): 602–609.

15. M.I. Love, W. Huber, S. Anders, "Moderated estimation of fold change and dispersion for RNA-seq data with DESeq2," *Genome Biology* 15, no. 12 (2014): 550.

16. T. Wu, E. Hu, S. Xu, et al., "ClusterProfiler 4.0: A universal enrichment tool for interpreting omics data," *Innovation (Camb)* 2, no. 3 (2021): 100141.

17. A. Subramanian, P. Tamayo, V.K. Mootha, et al., "Gene set enrichment analysis: a knowledge-based approach for interpreting genome-wide expression profiles," *Proceedings of the National Academy of Sciences of the United States of America* 102, no. 43 (2005): 15545–15550.

18. E. Dann, N.C. Henderson, S.A. Teichmann, et al., "Differential abundance testing on single-cell data using k-nearest neighbor graphs," *Nature Biotechnology* 40, no. 2 (2022): 245–253.

19. C.V. Jakubzick, G.J. Randolph, P.M. Henson, "Monocyte differentiation and antigen-presenting functions," *Nature Reviews: Immunology* 17, no. 6 (2017): 349–362.

20. K. Street, D. Risso, R.B. Fletcher, et al., "Slingshot: cell lineage and pseudotime inference for single-cell transcriptomics," *BMC Genomics* 19, no. 1 (2018): 477.

21. K. Van den Berge, H. Roux de Bézieux, K. Street, et al., "Trajectory-based differential expression analysis for single-cell sequencing data," *Nature Communications* 11, no. 1 (2020): 1201.

22. Z. Gu, R. Eils, M. Schlesner, “Complex heatmaps reveal patterns and correlations in multidimensional genomic data,” *Bioinformatics* 32, no. 18 (2016): 2847–2849.

23. Y. Li, Q. Yue, N.M. Krausert, et al., “Emestrins: Anti-cryptococcus epipolythiodioxopiperazines from *Podospora australis*,” *Journal of Natural Products* 79, no. 9 (2016): 2357–2363.

24. Y.M. Xu, P. Espinosa-Artiles, M.X. Liu, et al., “Secoemestrin D, a cytotoxic epitetrathiodioxopiperizine, and emericellenes A–E, five sesterterpenoids from *Emericella* sp. AST0036, a fungal endophyte of *Astragalus lentiginosus*,” *Journal of Natural Products* 76, no. 12 (2013): 2330–2336.

25. M. Oberhofer, J. Wackerlig, M. Zehl, et al., “Endophytic *Akanthomyces* sp. LN303 from edelweiss produces emestrin and two new 2-hydroxy-4 pyridone alkaloids,” *ACS Omega* 6, no. 3 (2021): 2184–2191.

26. F.Y. Lv, A. Mándi, X.M. Li, et al., “Emestrin-type thiodiketopiperazines from *Aspergillus nidulans* SD-531, a fungus obtained from the deep-sea sediment of cold seep in the South China Sea,” *Deep Sea Research Part I: Oceanographic Research Papers* 195 (2023): 104004.

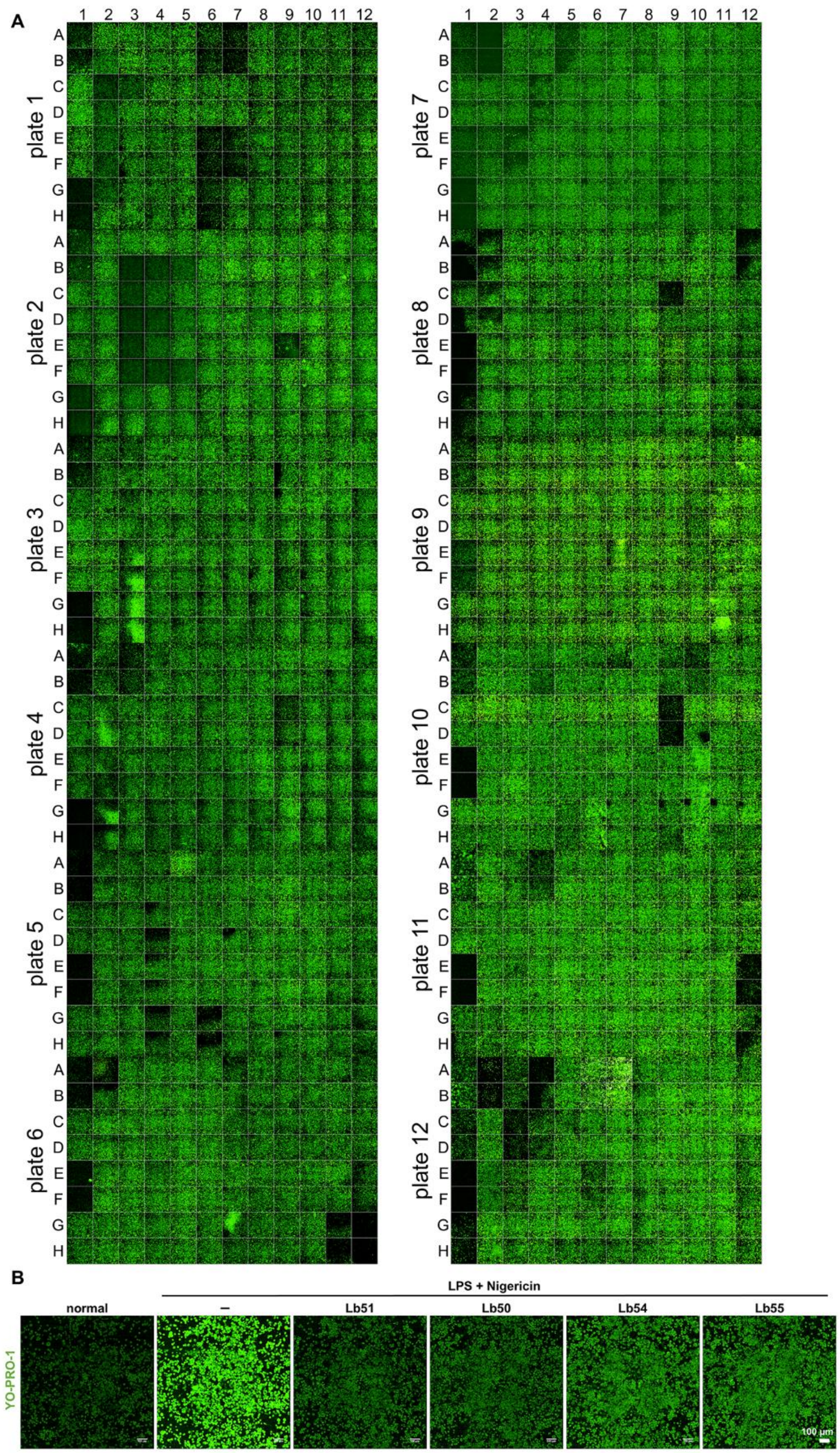

Figure S1. High content screening of 536 compounds.
(A) YO-PRO-1 staining results of HCS. (B) Individual pictures for indicated treatments.

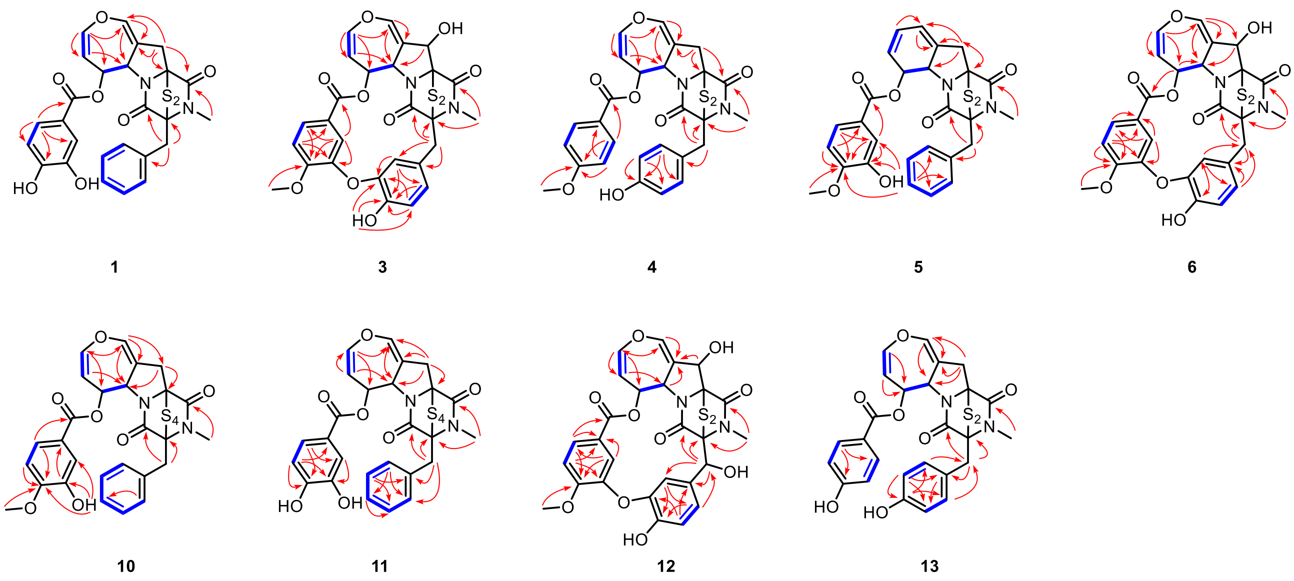

Figure S2. Selected ^{1}H–^{1}H COSY (blue bold lines) and HMBC (red arrows) correlations of compounds 1, 3–6, and 10–13.

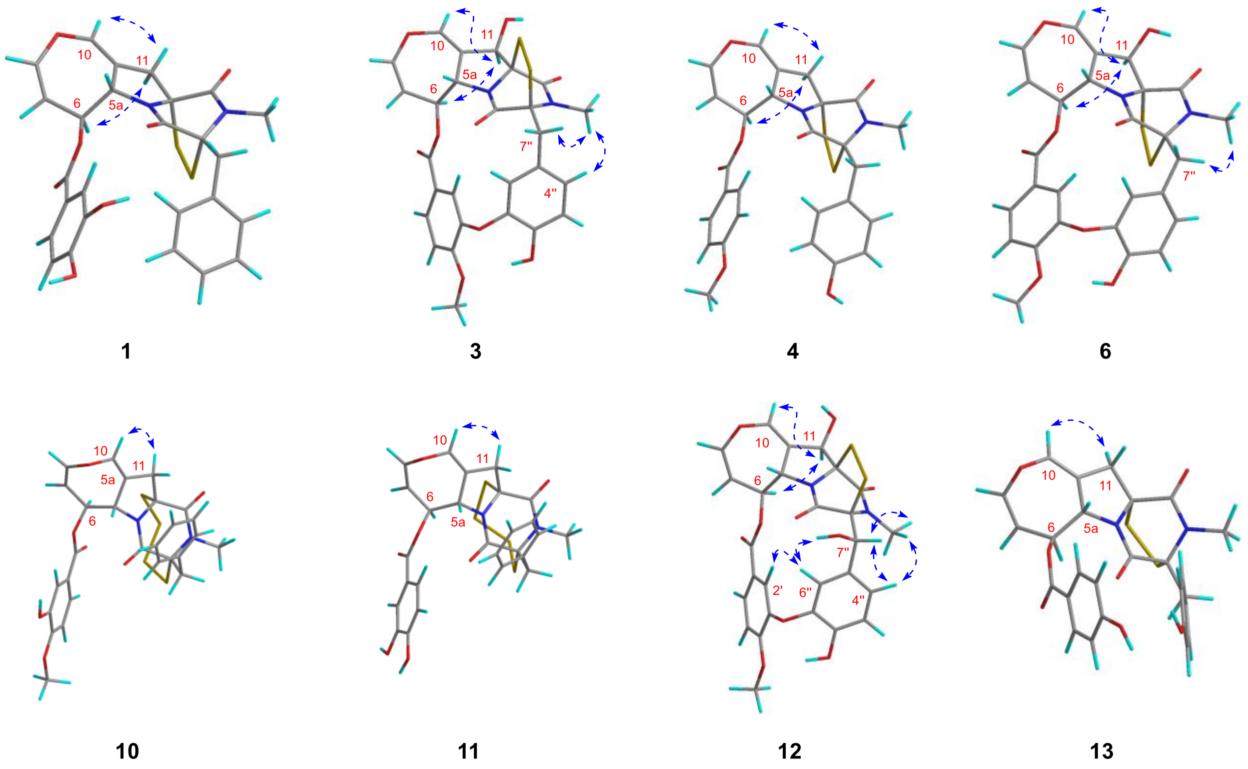

Figure S3. Selected NOESY/ROESY correlations of compounds 1, 3, 4, 6, and 10–13.

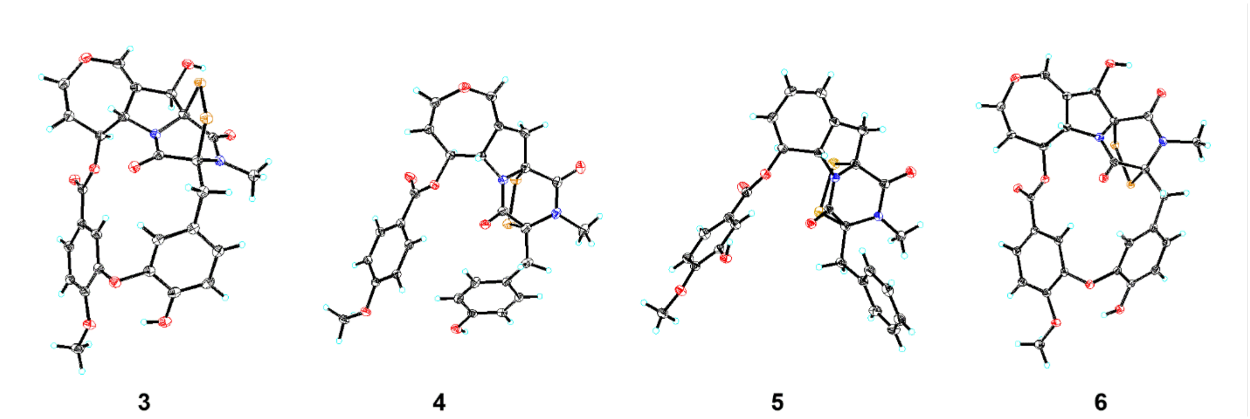

Figure S4. X-Ray crystallographic structures of 3–6.

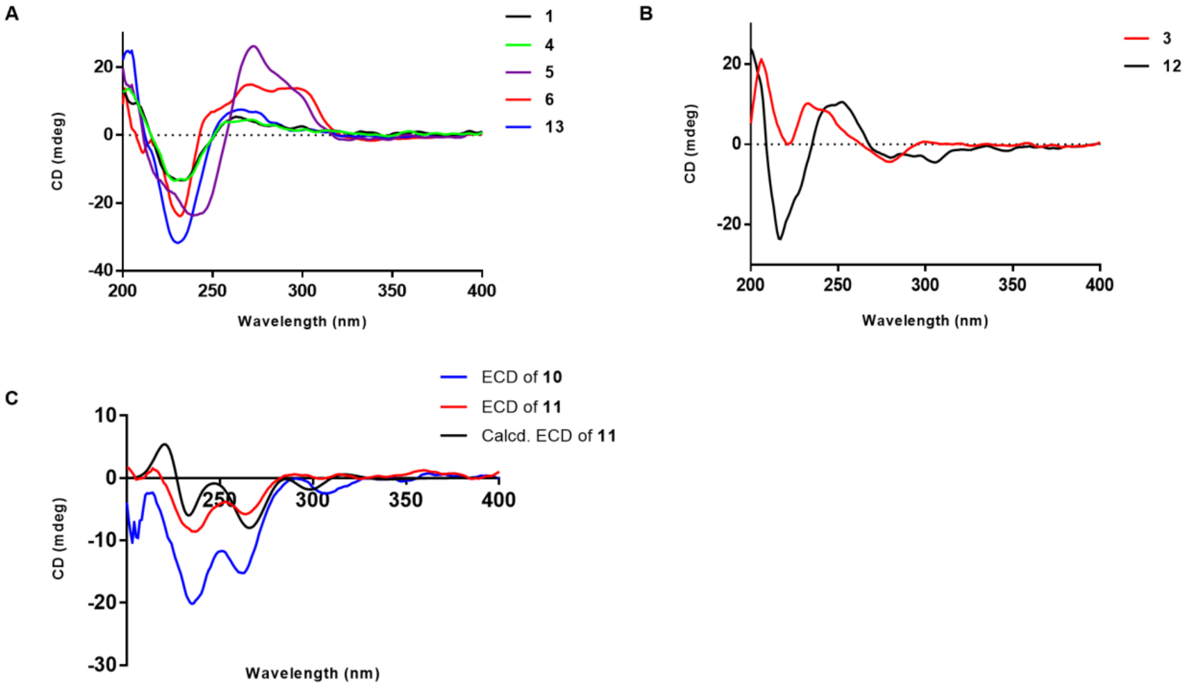

Figure S5. CD curves of compounds.

(**A**) Experimental ECD curves of compounds **1**, **4–6**, and **13**. (**B**) Experimental ECD curves of compounds **3** and **12**. (**C**) Experimental ECD curves of compounds **10–11** and calculated ECD curve of **11**.

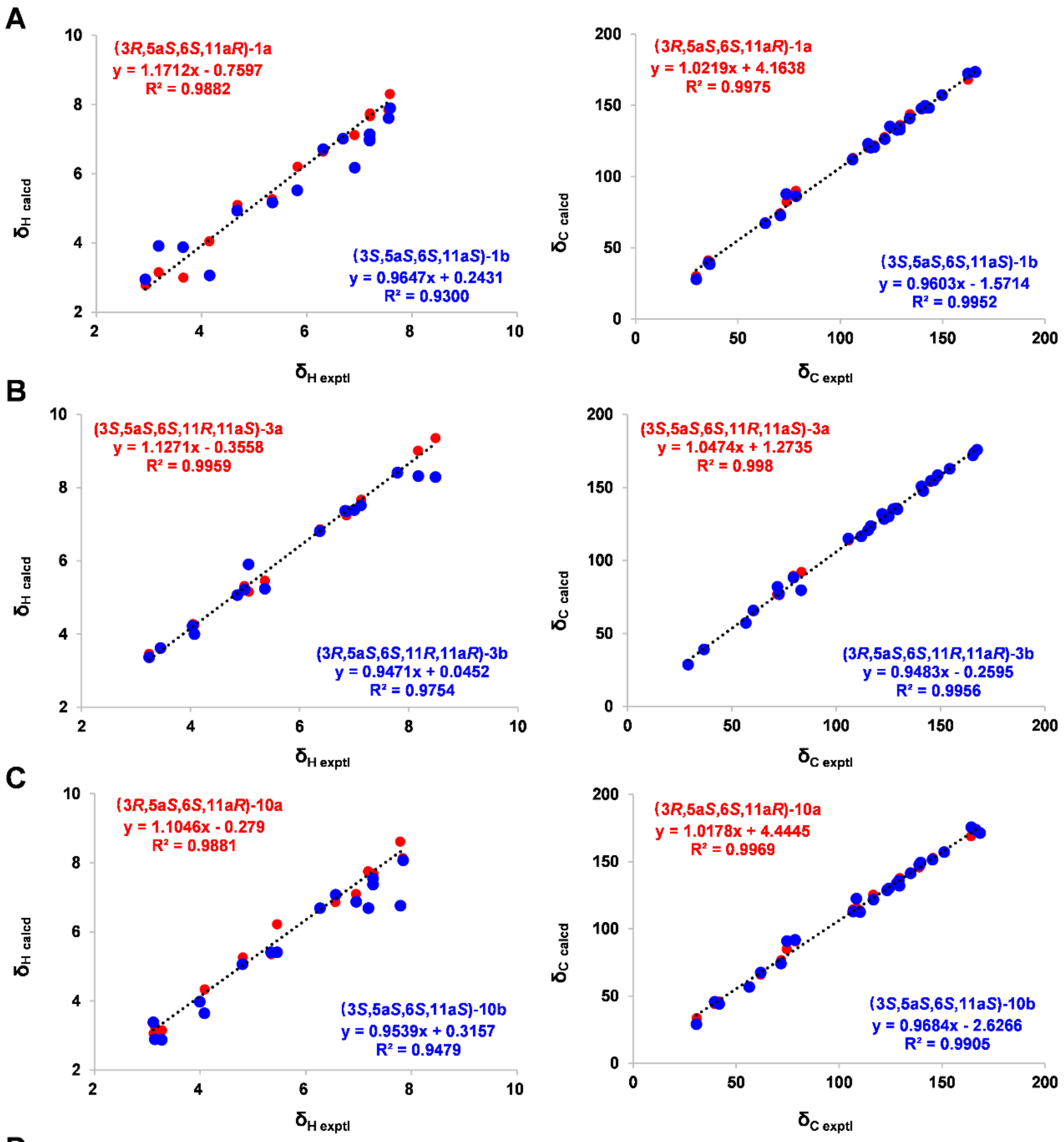

D

Default parameters	1a	1b	3a	3b	10a	10b
sDP4+ (H data)	100.00%	0.00%	100.00%	0.00%	100.00%	0.00%
sDP4+ (C data)	100.00%	0.00%	100.00%	0.00%	100.00%	0.00%
sDP4+ (all data)	100.00%	0.00%	100.00%	0.00%	100.00%	0.00%
DP4+ (H data)	100.00%	0.00%	100.00%	0.00%	100.00%	0.00%
DP4+ (C data)	100.00%	0.00%	100.00%	0.00%	100.00%	0.00%
DP4+ (all data)	100.00%	0.00%	100.00%	0.00%	100.00%	0.00%

Figure S6. NMR calculations with DP4+ probability analysis for 1, 3, and 10.
(**A**) Linear correlation plots of experimental *vs.* calculated ^{1}H NMR (left) and ^{13}C NMR (right) chemical shifts of **1**. (**B**) Linear correlation plots of experimental *vs.* calculated ^{1}H NMR (left) and ^{13}C NMR (right) chemical shifts of **3**. (**C**) Linear correlation plots of experimental *vs.* calculated ^{1}H NMR (left) and ^{13}C NMR (right) chemical shifts of **10**. (**D**) DP4+ probability analysis.

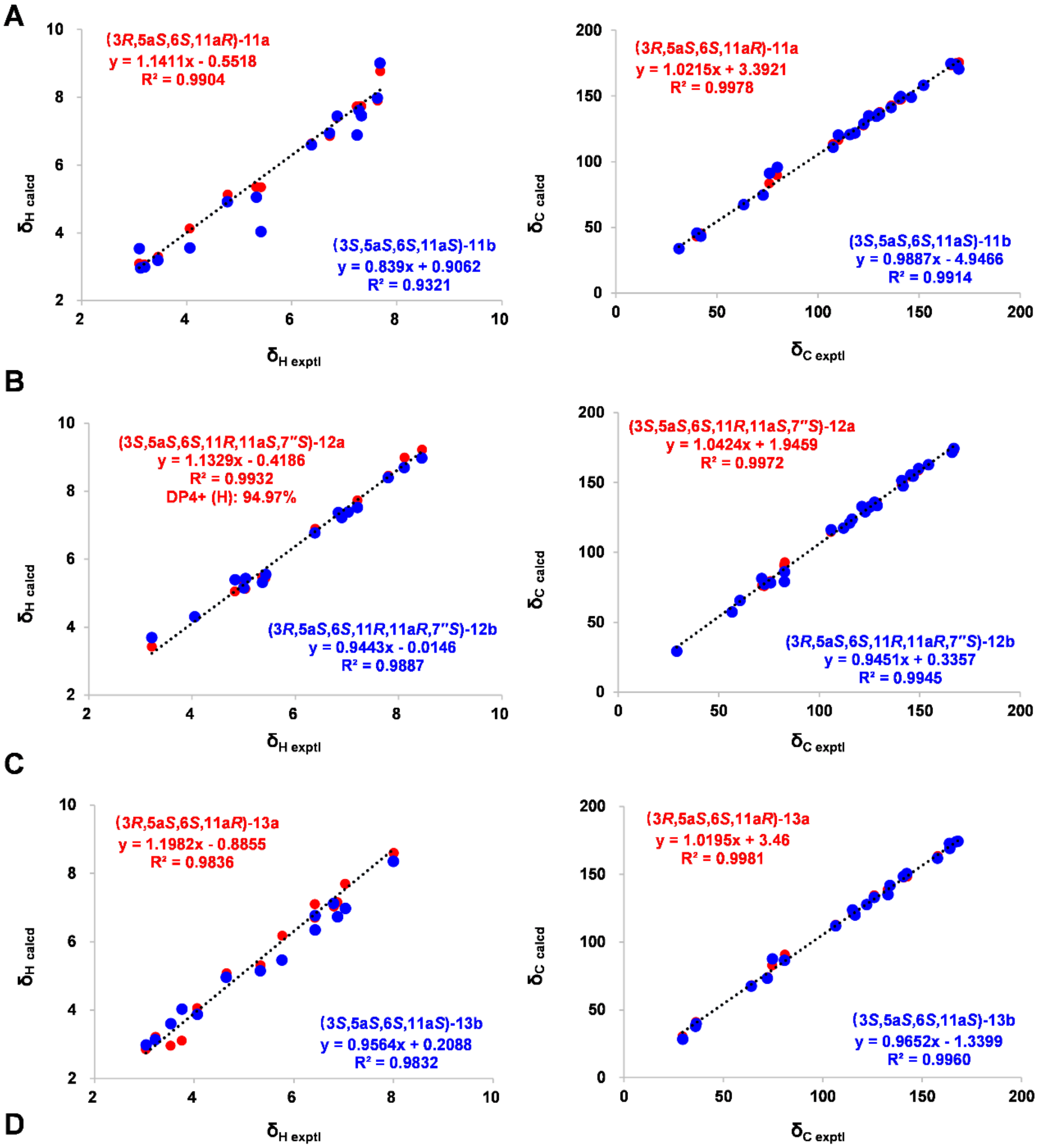

D

Default parameters	11a	11b	12a	12b	13a	13b
sDP4+ (H data)	100.00%	0.00%	94.97%	5.03%	79.02%	20.98%
sDP4+ (C data)	100.00%	0.00%	100.00%	0.00%	100.00%	0.00%
sDP4+ (all data)	100.00%	0.00%	100.00%	0.00%	100.00%	0.00%
DP4+ (H data)	100.00%	0.00%	82.82%	17.18%	87.21%	12.79%
DP4+ (C data)	100.00%	0.00%	100.00%	0.00%	100.00%	0.00%
DP4+ (all data)	100.00%	0.00%	100.00%	0.00%	100.00%	0.00%

Figure S7. NMR calculations with DP4+ probability analysis for 11–13.
(**A**) Linear correlation plots of experimental *vs.* calculated ^{1}H NMR (left) and ^{13}C NMR (right) chemical shifts of **11**. (**B**) Linear correlation plots of experimental *vs.* calculated ^{1}H NMR (left) and ^{13}C NMR (right) chemical shifts of **12**. (**C**) Linear correlation plots of experimental *vs.* calculated ^{1}H NMR (left) and ^{13}C NMR (right) chemical shifts of **13**. (**D**) DP4+ probability analysis.

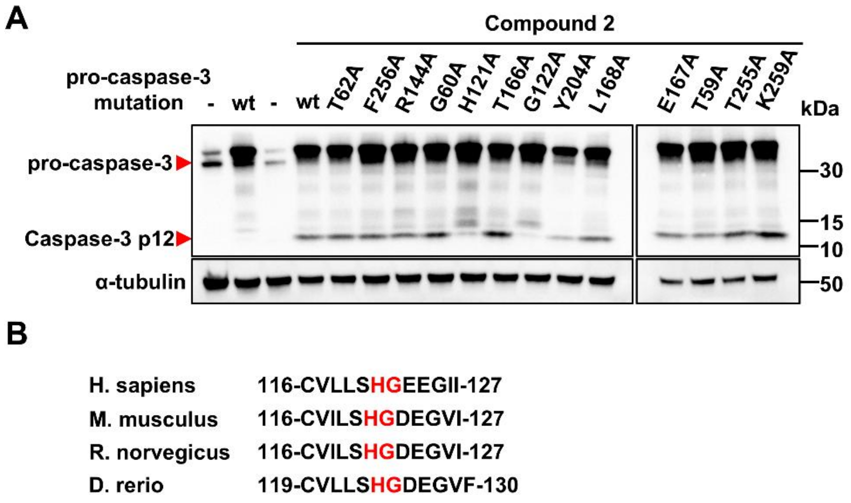

Figure S8. Mutagenesis analysis of pro-caspase-3 in HEK293T cells.

(**A**) Western blotting analysis of HEK293T cells overexpressing wild type or different point mutation pro-caspase-3 protein treated with compound **2** (1 μM). (**B**) A predicted binding site of compound **2** in pro-caspase-3 protein is conserved across the indicated species.

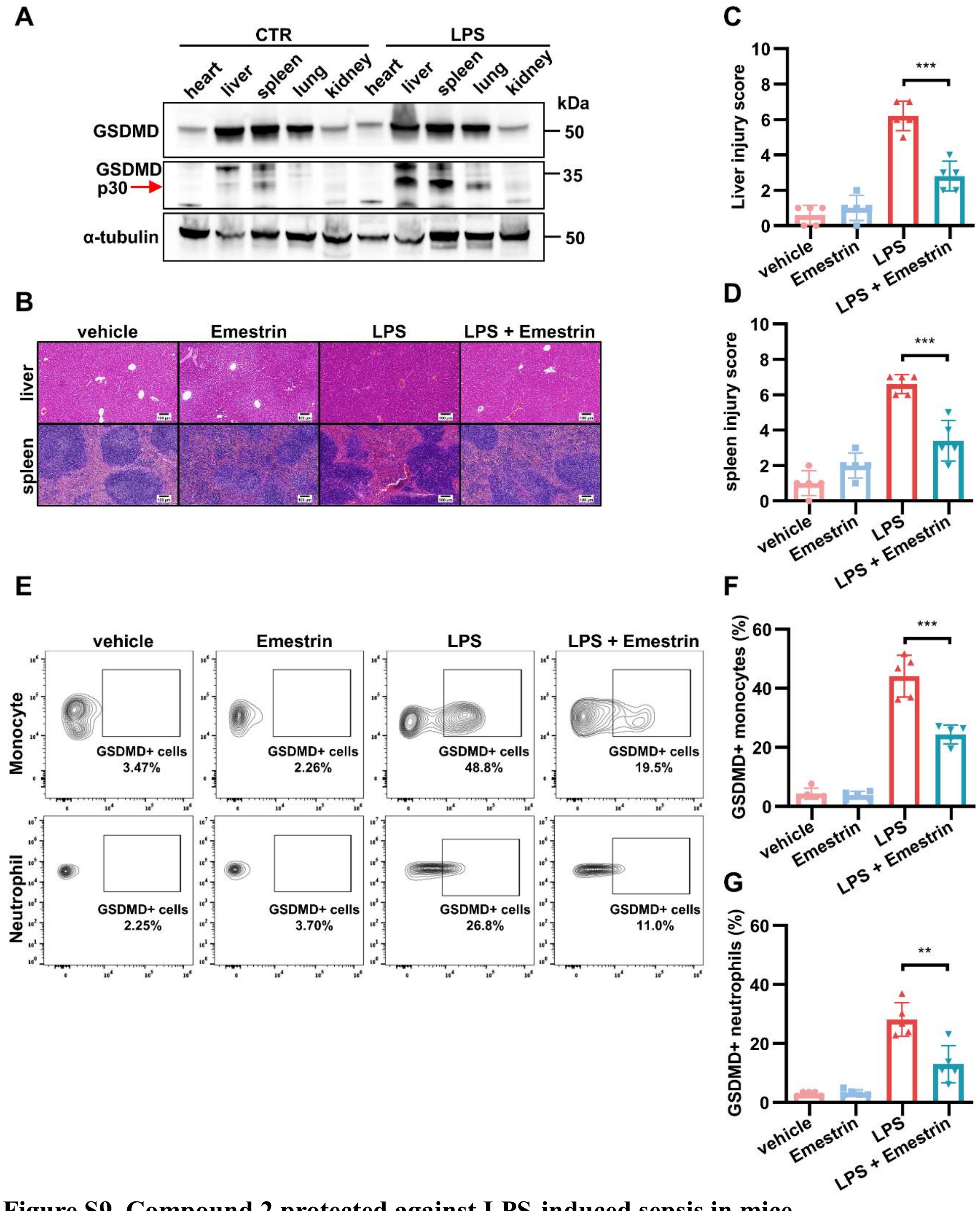

Figure S9. Compound 2 protected against LPS-induced sepsis in mice.

(**A**) Western blotting analysis of GSDMD cleavage and α-tubulin in mouse different organ tissue samples 12 h post 25 mg/kg LPS injection. (**B**–**D**) HE staining of mouse liver and spleen tissue samples 12 h post 25 mg/kg LPS injection. Liver and spleen injury score were analyzed. (mean ± SD; n = 5 per group, ***p < 0.001). (**E**–**G**) Flow cytometry analysis of GSDMD-positive neutrophils and monocytes in lung samples from mice model 12 h post 25 mg/kg LPS injection (mean ± SD, n = 5 per group, **p < 0.01; ***p < 0.001).

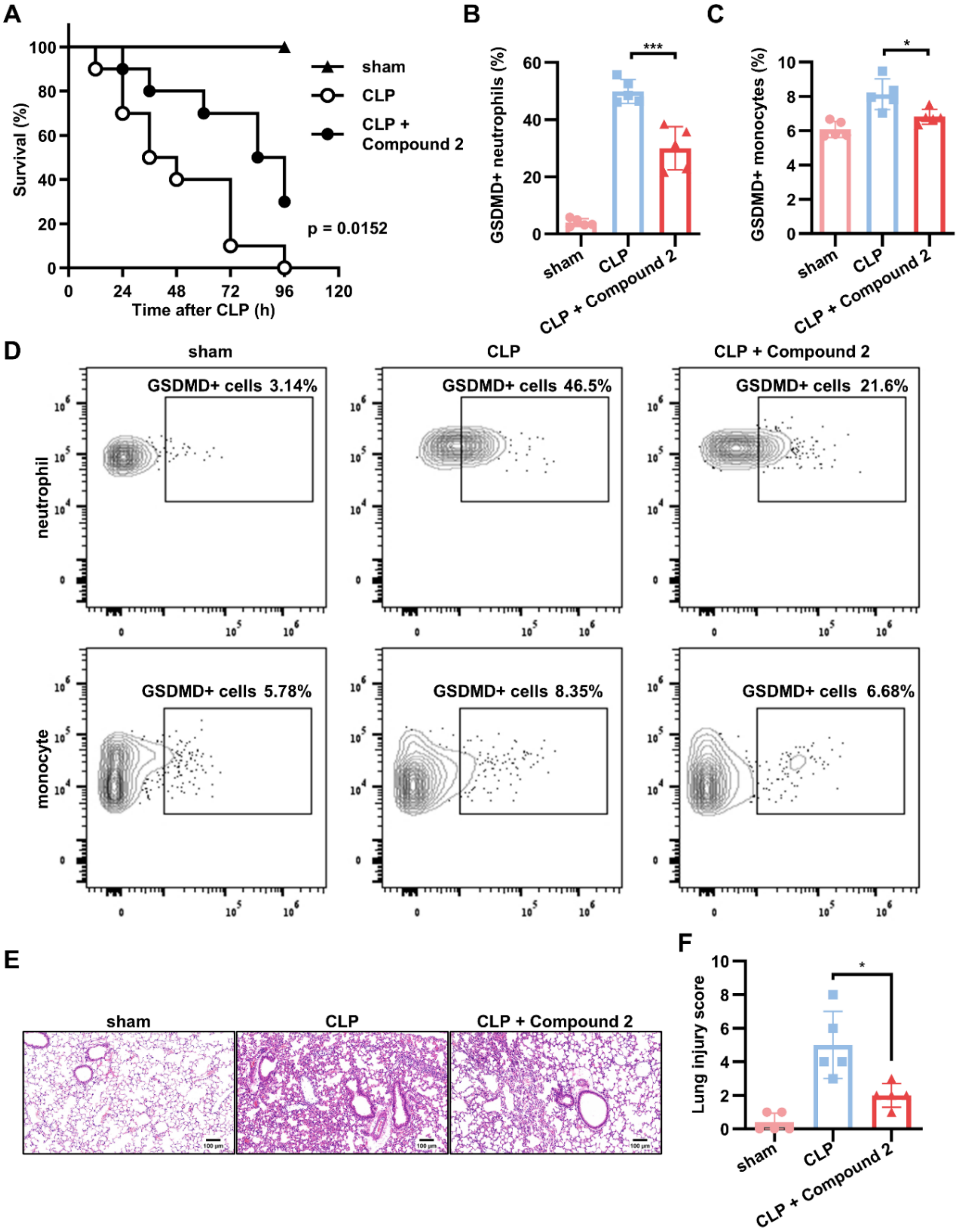

Figure S10. Compound 2 protected against CLP-induced sepsis in mice.
(**A**) Kaplan-Meier survival plot of mice subjected to CLP surgery following pretreatment with compound **2** (2 mg/kg) or vehicle (n = 10 per group, log rank test, p = 0.0152). (**B–D**) Flow cytometry analysis of GSDMD-positive neutrophils and monocytes in lung samples from mice model 12 h post CLP surgery (mean ± SD, n = 5 per group, ns means not significant, *p < 0.05; ***p < 0.001). (**E–F**) HE staining of mouse lung tissue samples. Lung injury score was analyzed (mean ± SD; n = 5 per group, *p < 0.05).

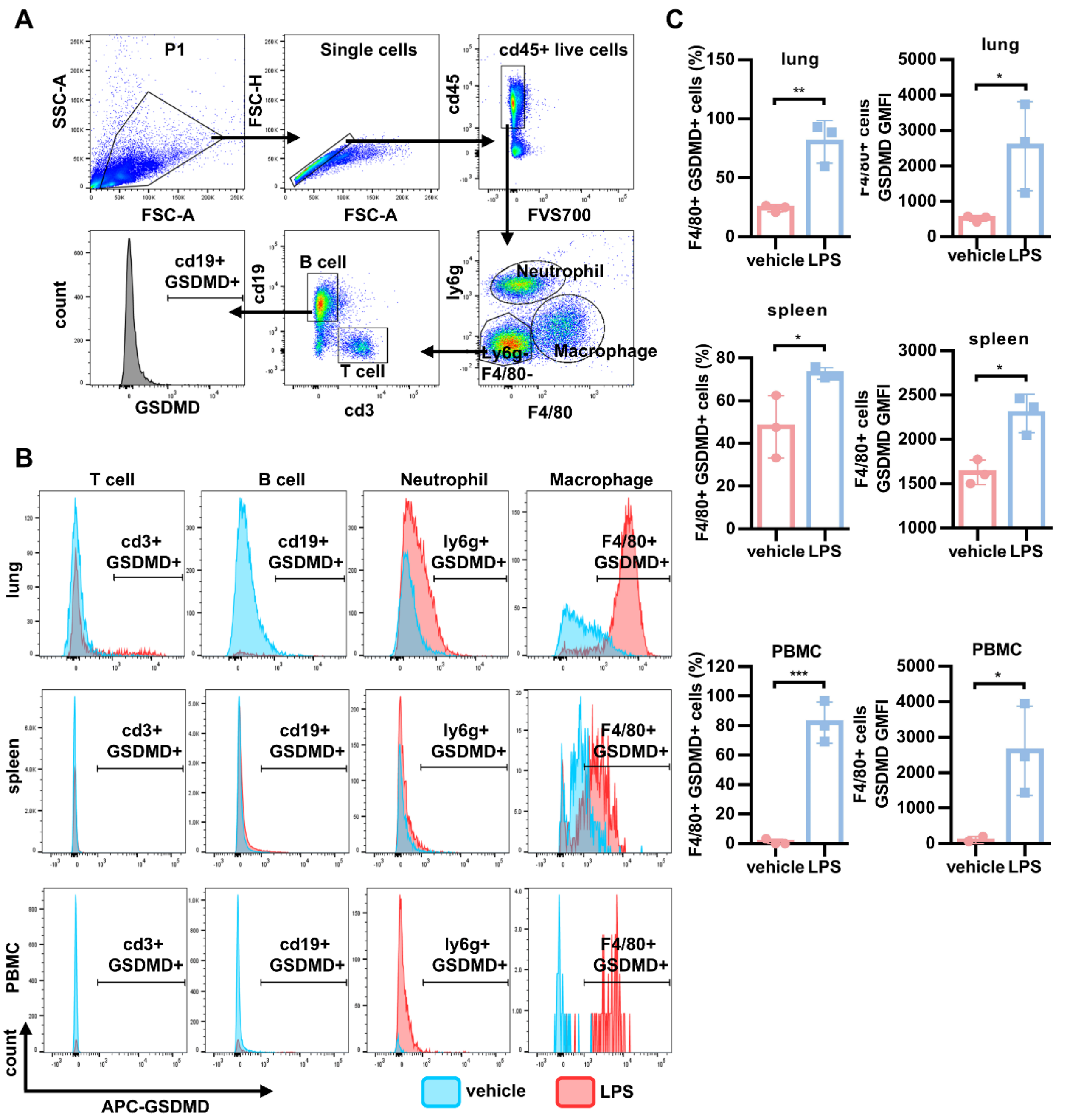

Figure S11. Flow cytometry analysis of different organ tissue in mouse LPS challenge model. (**A**) Gating strategy for different immune cells in mouse LPS challenge model. (**B**–**C**) Flow cytometry analysis of PBMC, spleen and lung tissue in mouse LPS challenge model.

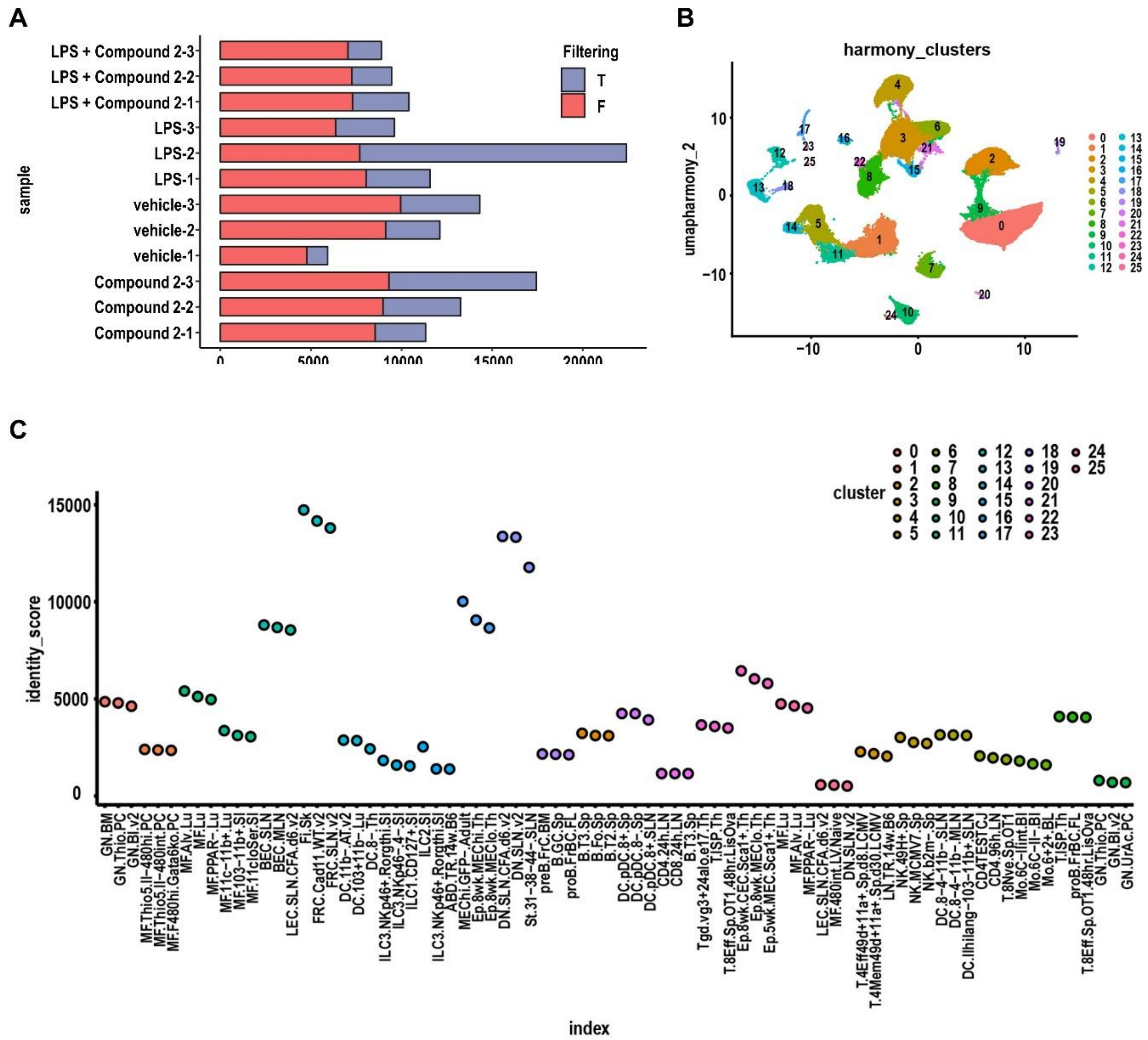

Figure S12. Preprocessing, dimensional reduction, clustering, and cell annotation of scRNAseq data.

(**A**) Barplot showing number of filtered and non-filtered cells in each sample. (**B**) UMAP and clustering of cells. (**C**) Identity score of each clusters using CIPR with Immgen as reference.

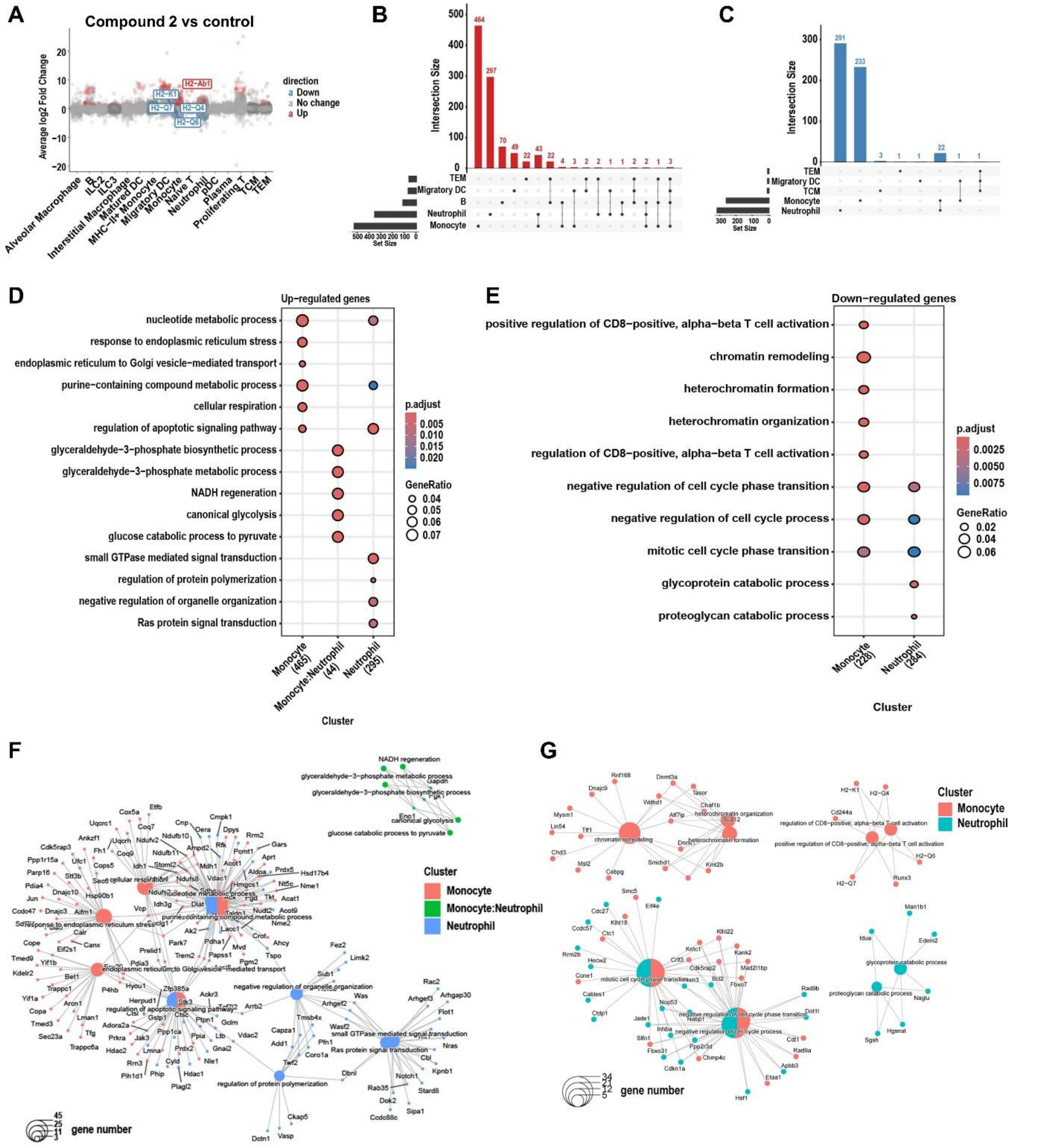

Figure S13. Gene expression changes of lung immune cells treated with compound 2.
(**A**) Scatterplot showing pseudobulk differential expressed genes of immune cell types comparing compound **2** with vehicle. Grey dots, $p > 0.05$; red, $p < 0.05$ and $\log_2 FC > 0.6$ (up-regulated), blue, $p < 0.05$ and $\log_2 FC < -0.6$ (down-regulated). p-values were obtained by DESeq2 testing. (**B**) Intersect of up-regulated differentially expressed genes. (**C**) Intersect of down-regulated differentially expressed genes. (**D**) Enriched gene ontology terms of up-regulated intersected gene modules. (**E**) Enriched gene ontology terms of down-regulated intersected gene modules. (**D**–**E**, adjusted $p < 0.05$.) (**F**–**G**) Plot of gene-concept networks using Cnetplot function of clusterProfiler to identify overlapping genes based on the enriched gene ontology terms in d and e respectively.

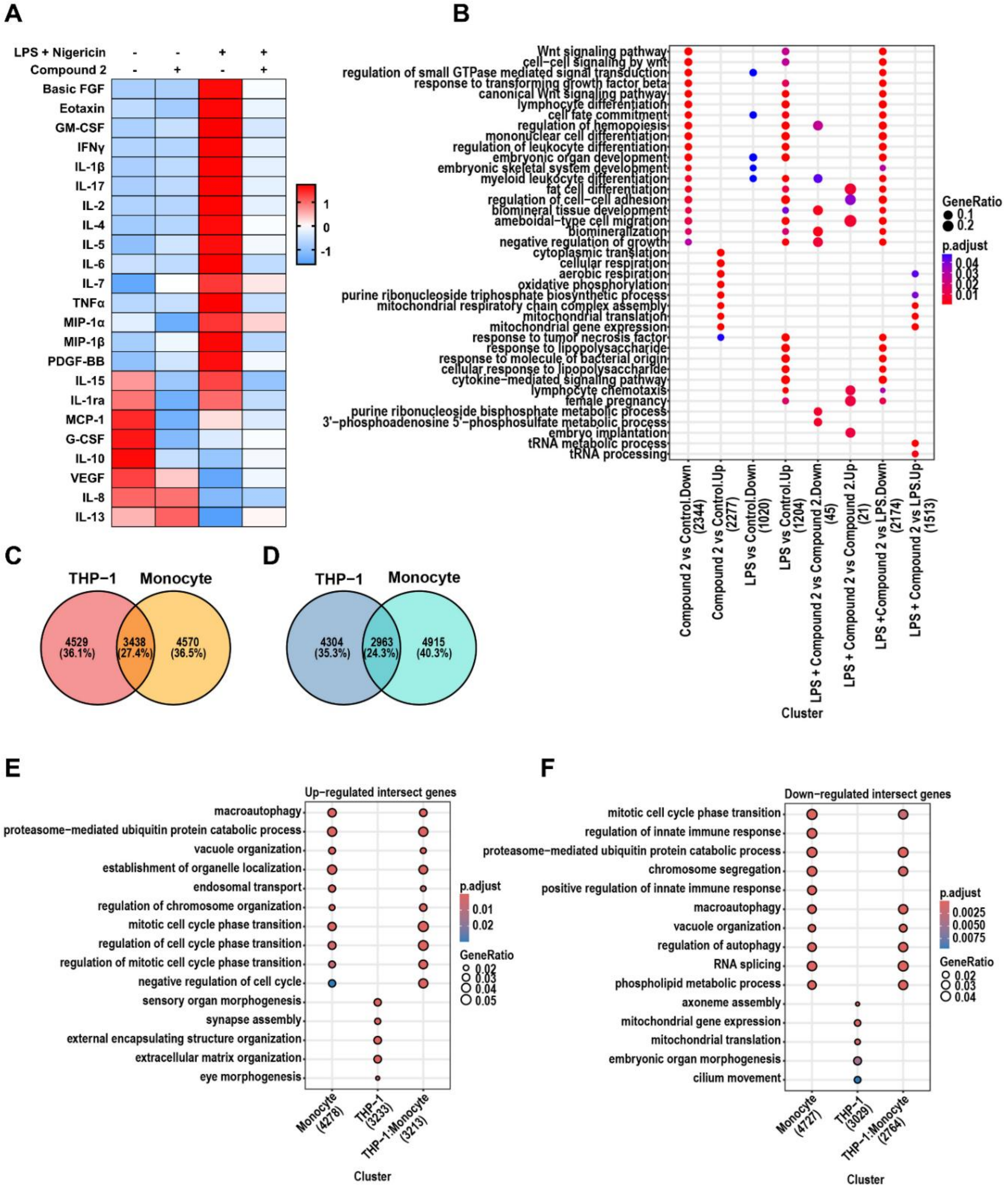

Figure S14. Inflammatory chemokines release result and RNA-seq analysis of THP-1 differentiated macrophages.

(**A**) Inflammatory chemokines of THP-1 differentiated macrophages culture medium were measured by luminex liquid suspension chip. (**B**) Enriched gene ontology term of up- and down-regulated differentially expressed genes across conditions (control, $n = 3$; compound **2**, $n = 3$, LPS, $n = 3$, LPS + compound **2**, $n = 3$) with human THP-1 differentiated macrophages. (**C**–**D**) Intersect of genes up- (**C**) and down-regulated (**D**) in THP-1 cell line and lung monocyte mice in response to LPS with compound **2** compare to LPS only. (**E**–**F**) Enriched Gene ontology biological process terms of the intersected and non-intersected genes in **C** and **D**.

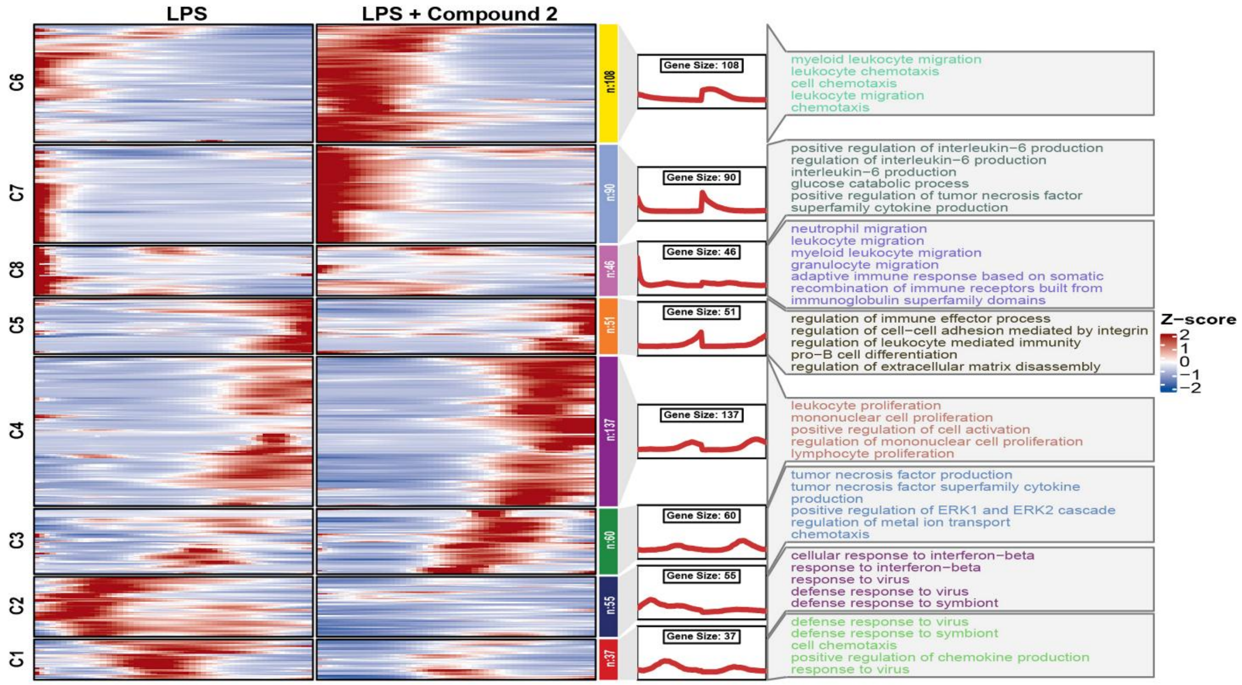

Figure S15. Pseudotime trajectory DEGs (absolute fold change > 1, adjusted *p*-value < 0.05) between conditions clustered into 8 modules based on expression patterns.

Figure S16. ^{1}H **NMR spectrum of compound 1 (Recorded in** $CDCl_3$**)**

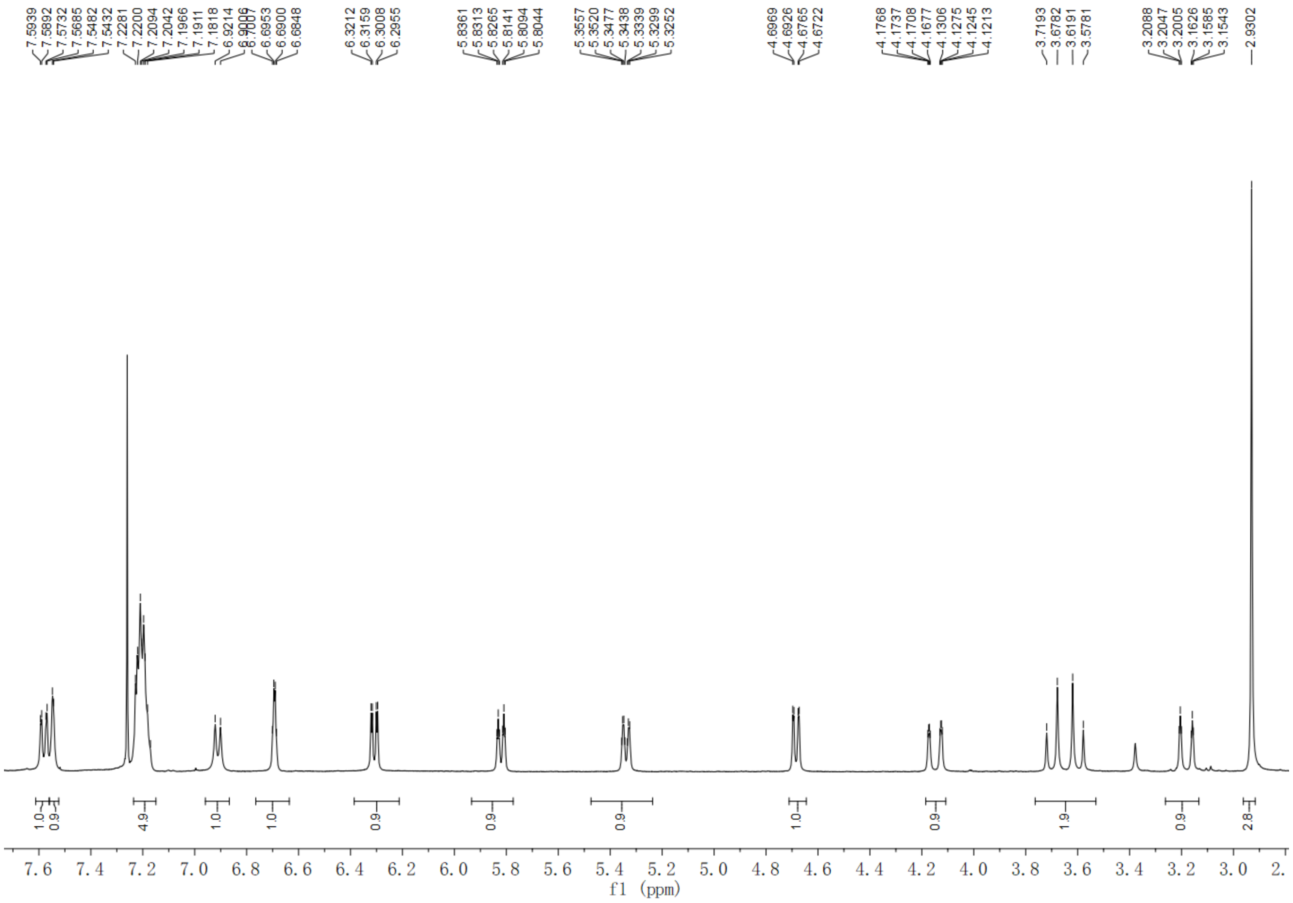

Figure S17. ^{13}C NMR and DEPT spectra of compound 1 (Recorded in $CDCl_3$)

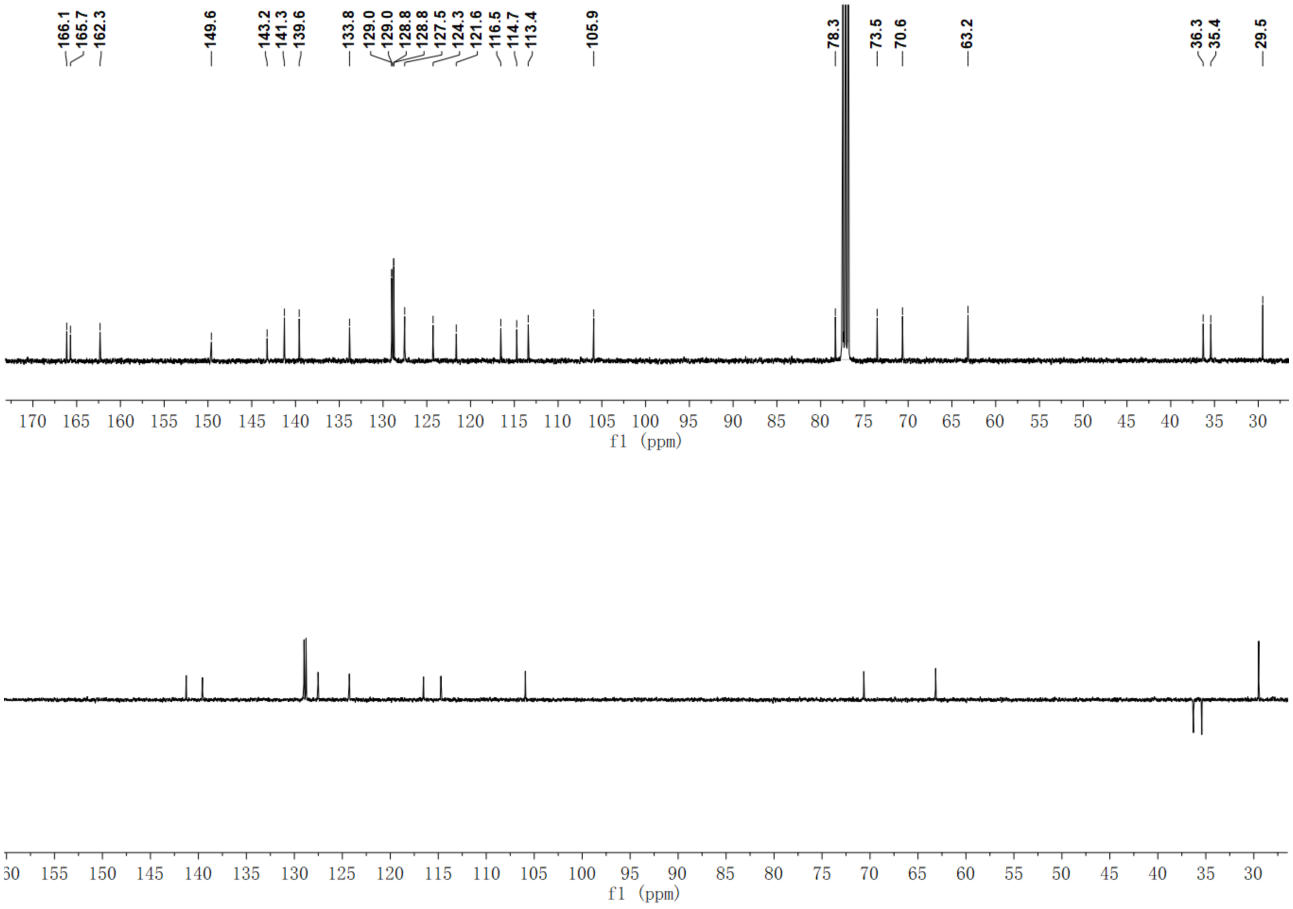

Figure S18. HSQC spectrum of compound 1 (Recorded in $CDCl_3$)

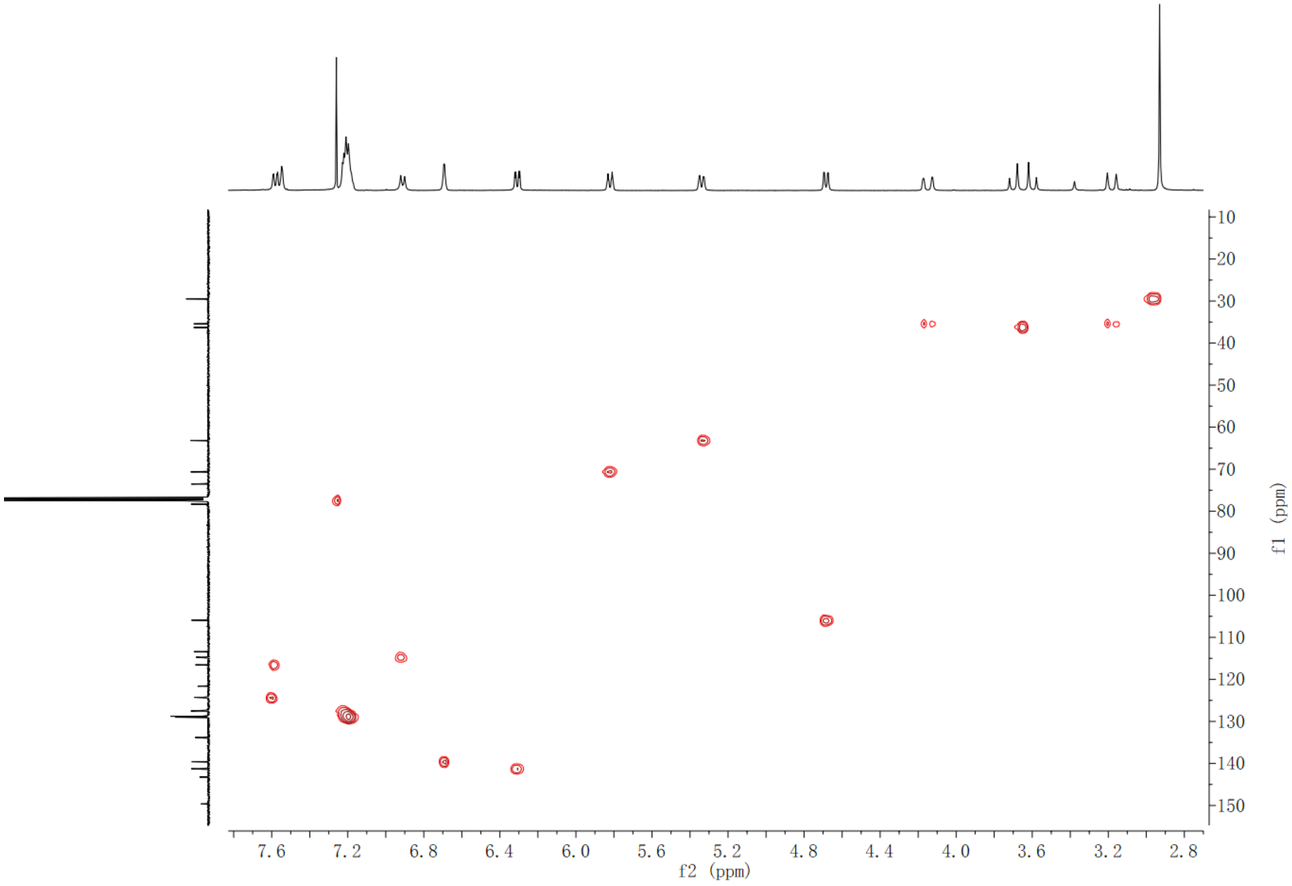

Figure S19. HMBC spectrum of compound 1 (Recorded in $CDCl_3$)

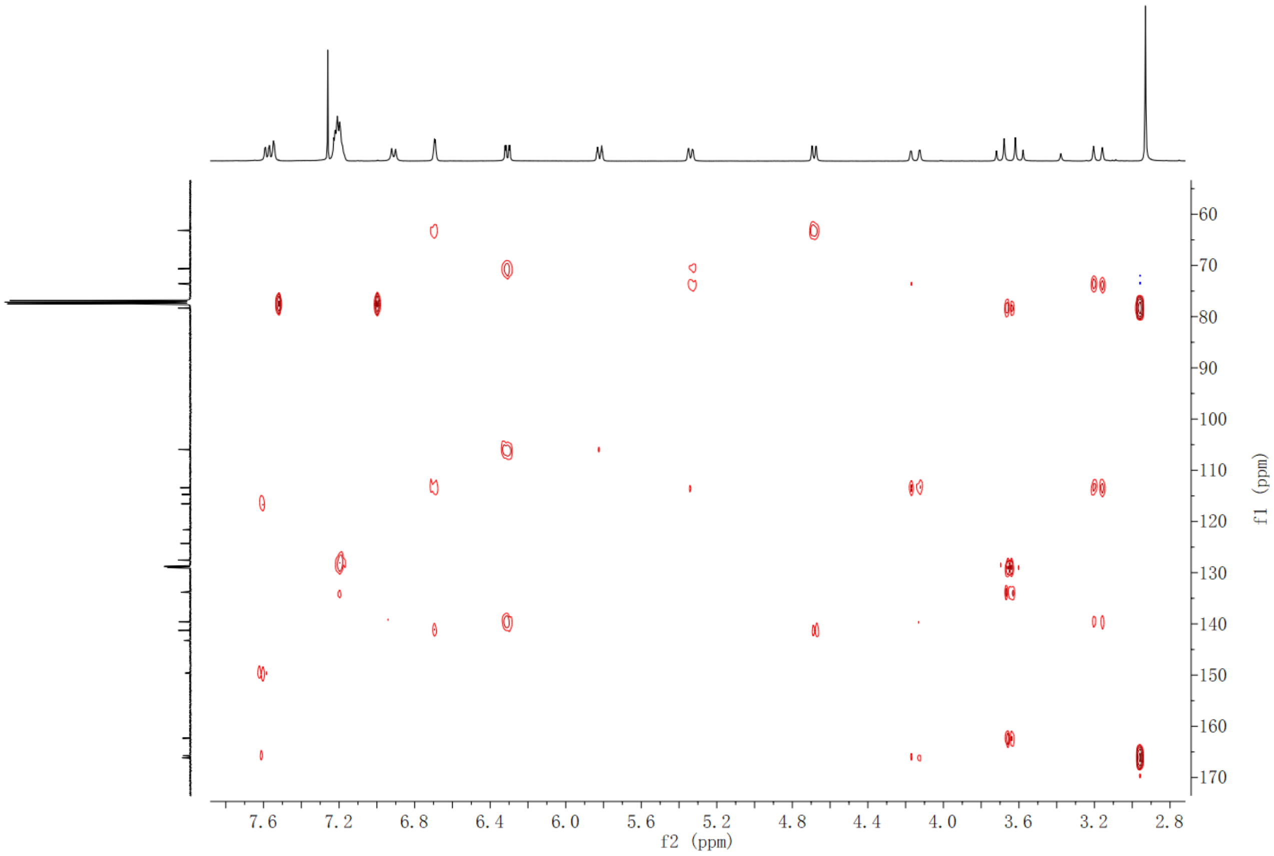

Figure S20. ^{1}H–^{1}H **COSY spectrum of compound 1 (Recorded in** $CDCl_3$**)**

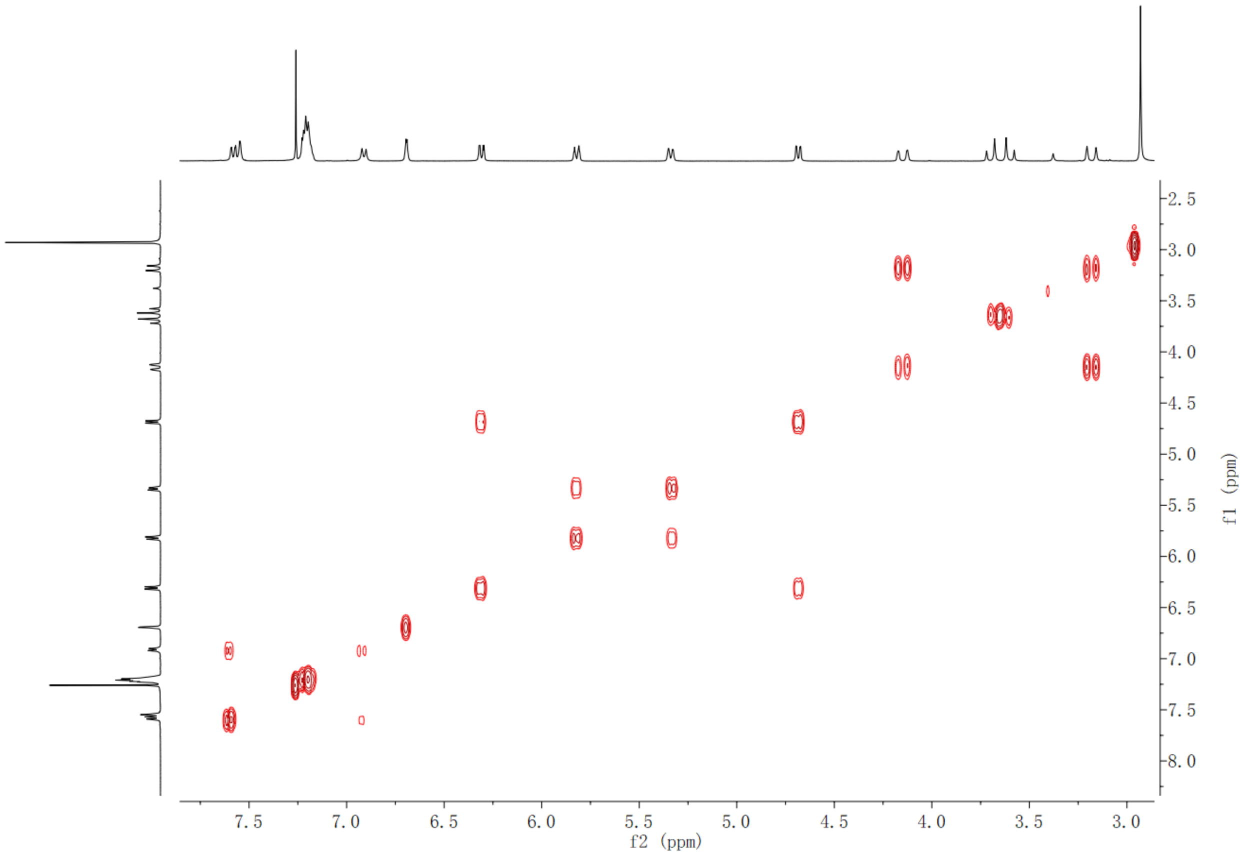

Figure S21. NOESY spectrum of compound 1 (Recorded in $CDCl_3$)

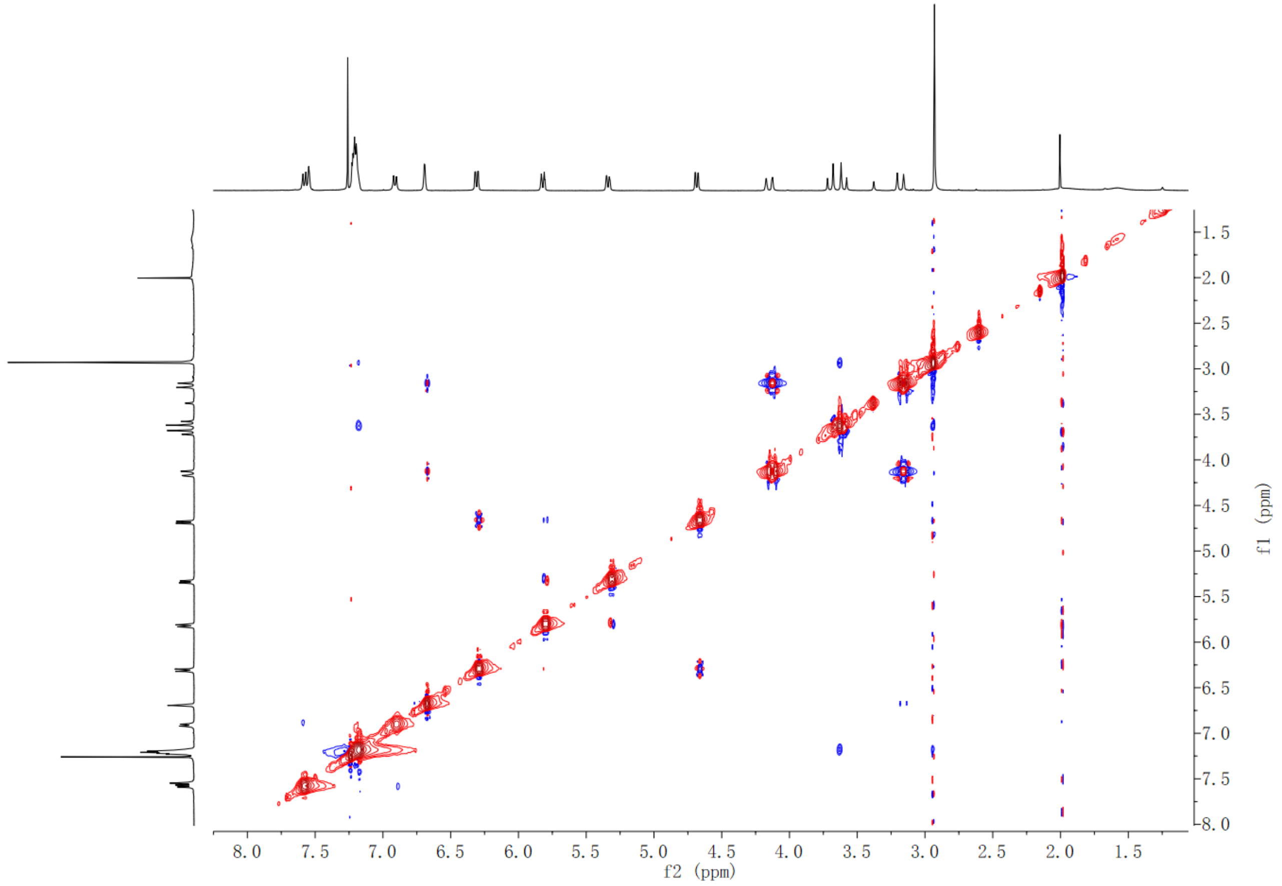

Figure S22. HRESIMS spectrum of compound 1

Mass Spectrum SmartFormula Report

Analysis Info		Acquisition D	2021/6/25 15:13:25
Analysis Name	H:\MS\LS 5-8-4.d		
Method	Tune_pos_Mid.m	Operator	BDAL@DE
Sample Name		Instrument	micrOTOF II 8213750.10426
Comment			

Acquisition Paramet

Source Type	ESI	Ion Polarity	Positive	Set Nebulizer	0.3 Bar
Focus	Not active			Set Dry Heater	200 °C
Scan Begin	100 m/z	Set Capillary	4500 V	Set Dry Gas	4.0 l/min
Scan End	3300 m/z	Set End Plate Offset	-500 V	Set Divert Valve	Waste

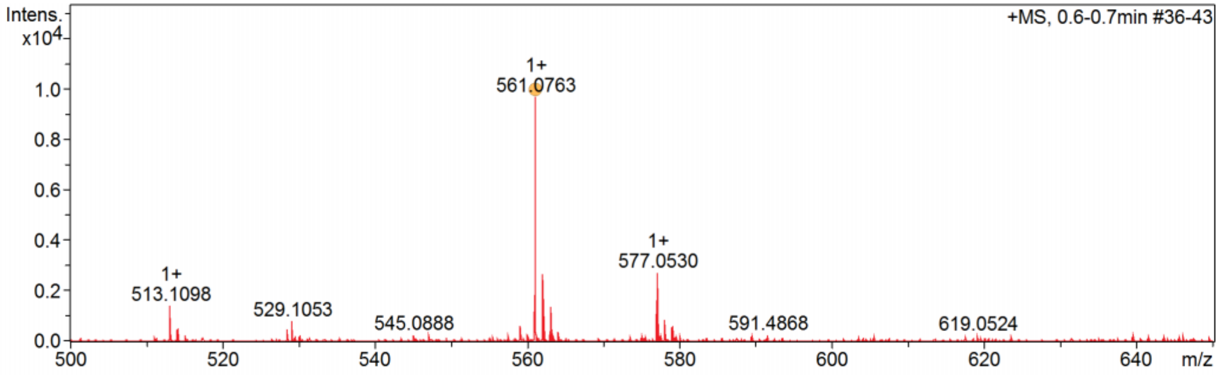

Meas. m/z	#	Ion Formula	m/z	err [ppm]	Mean err [ppm]	rdb	N-Rule	e¡¥ Conf	mSigma	Std I
561.076292	1	C26H22N2NaO7S2	561.076064	-0.4	984.8	16.5	ok	even	15.9	28.4

Figure S23. UV spectrum of compound 1

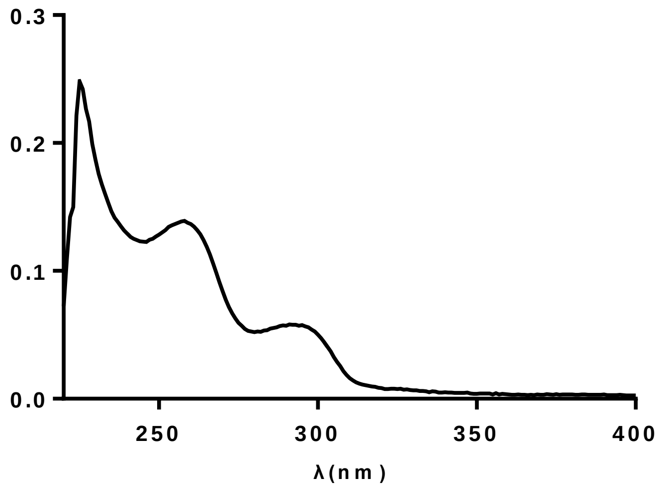

Figure S24. IR spectrum of compound 1

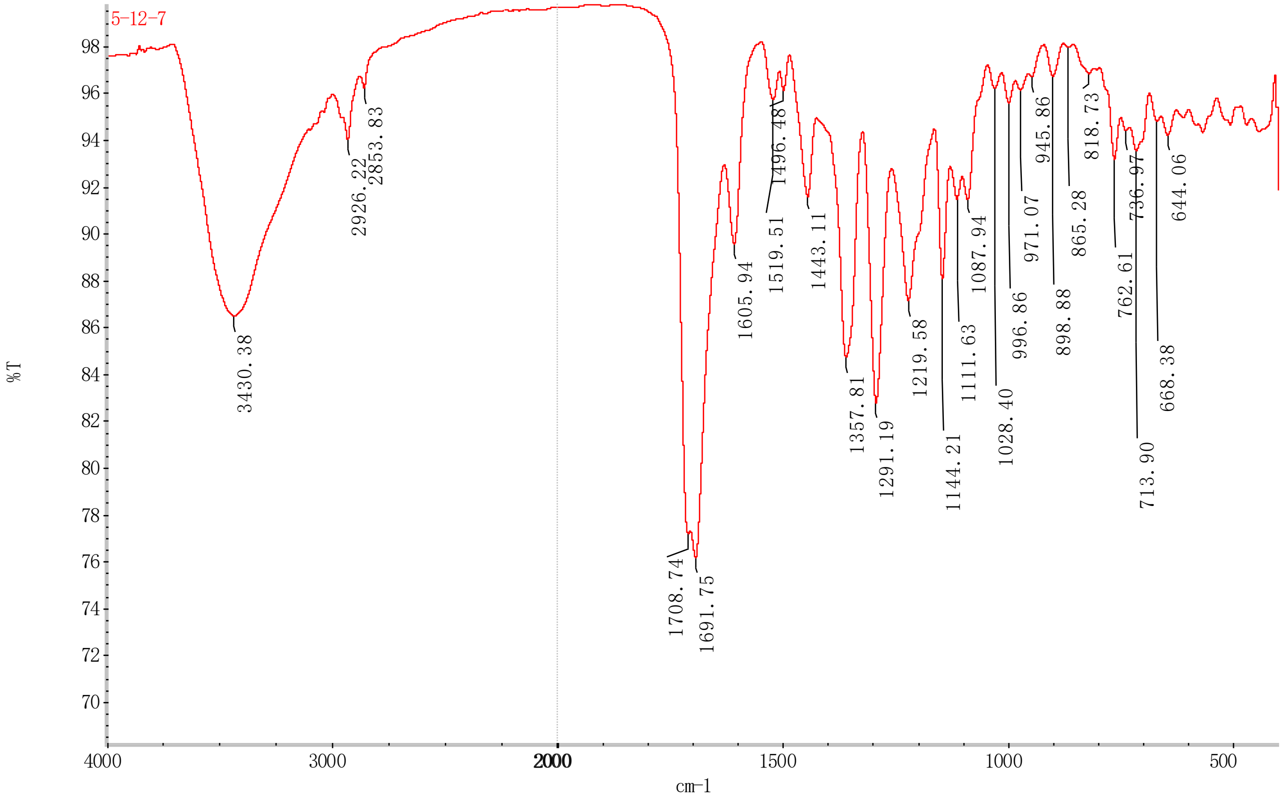

Figure S25. ¹H NMR spectrum of compound 3 (Recorded in $CDCl_3$)

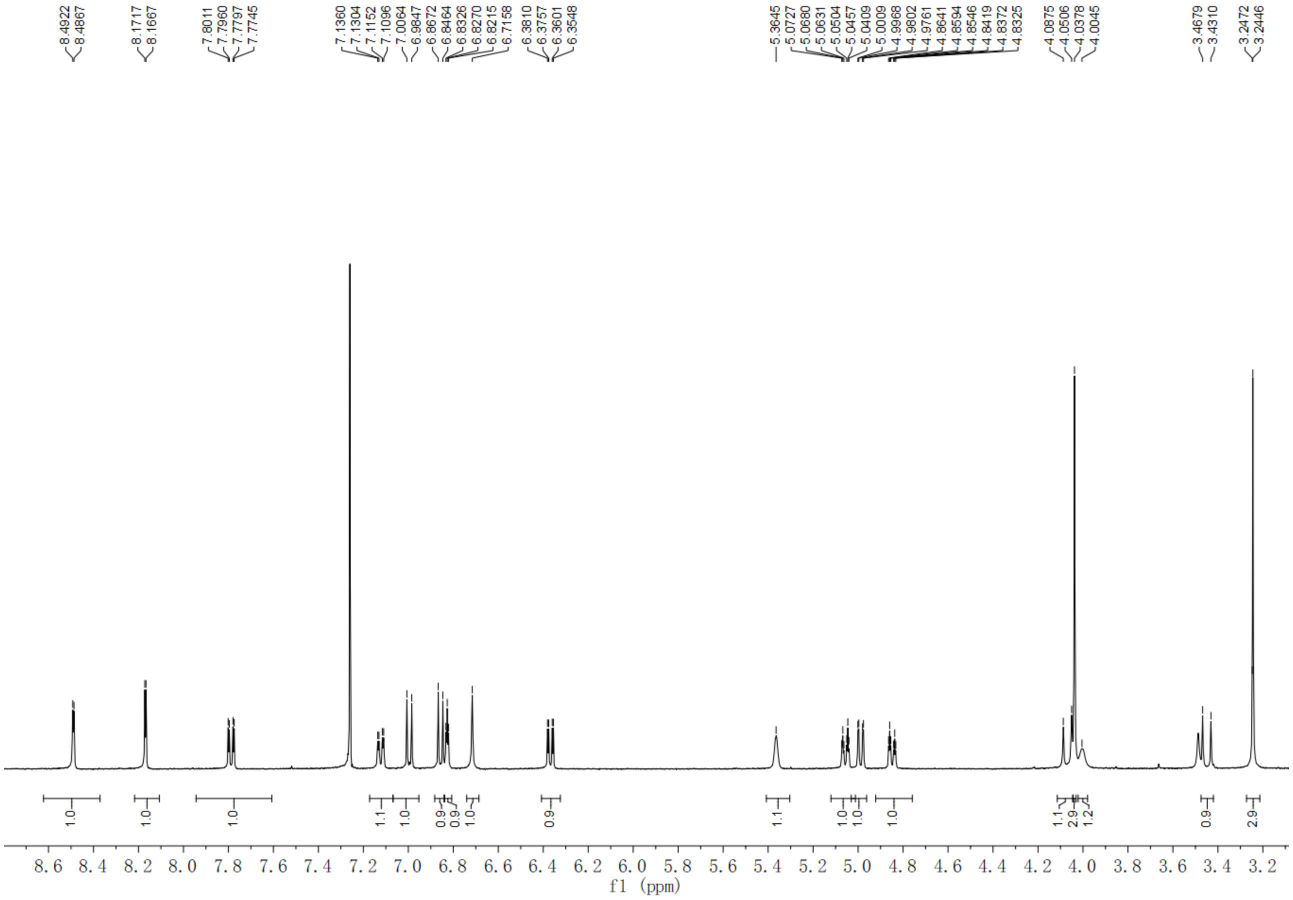

Figure S26. ^{13}C NMR and DEPT spectra of compound 3 (Recorded in $CDCl_3$)

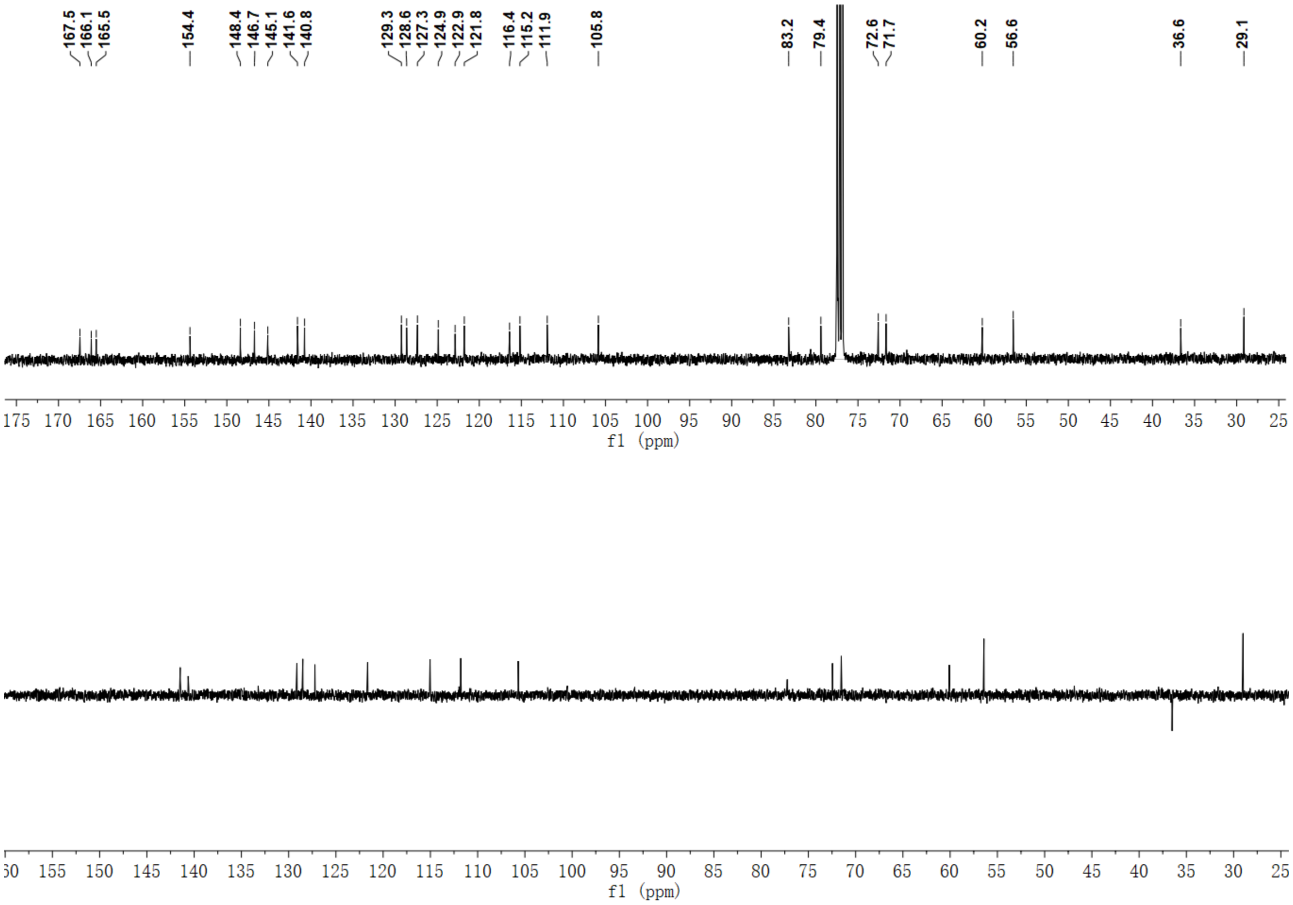

Figure S27. HSQC spectrum of compound 3 (Recorded in CDCl$_3$)

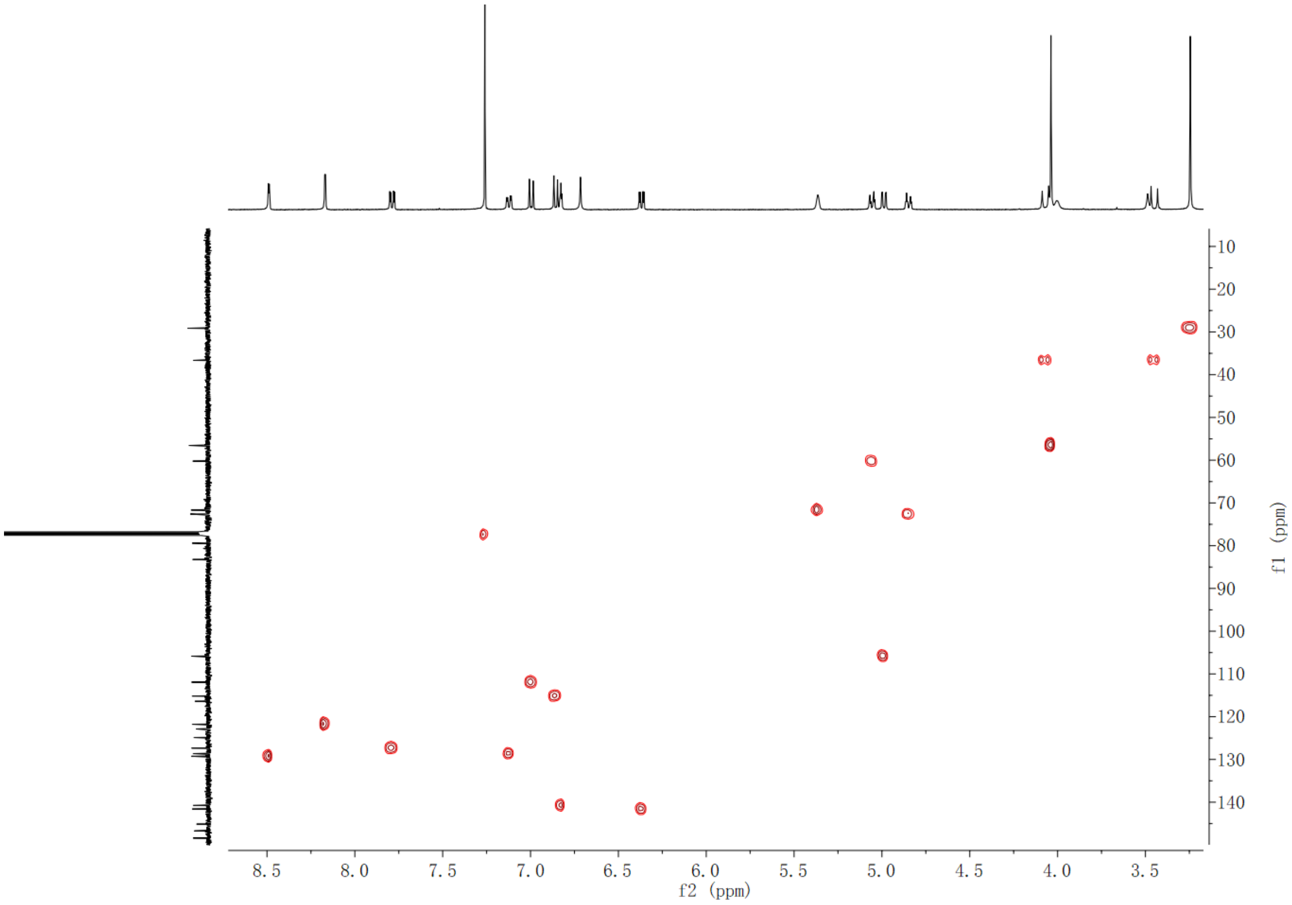

Figure S28. HMBC spectrum of compound 3 (Recorded in CDCl₃)

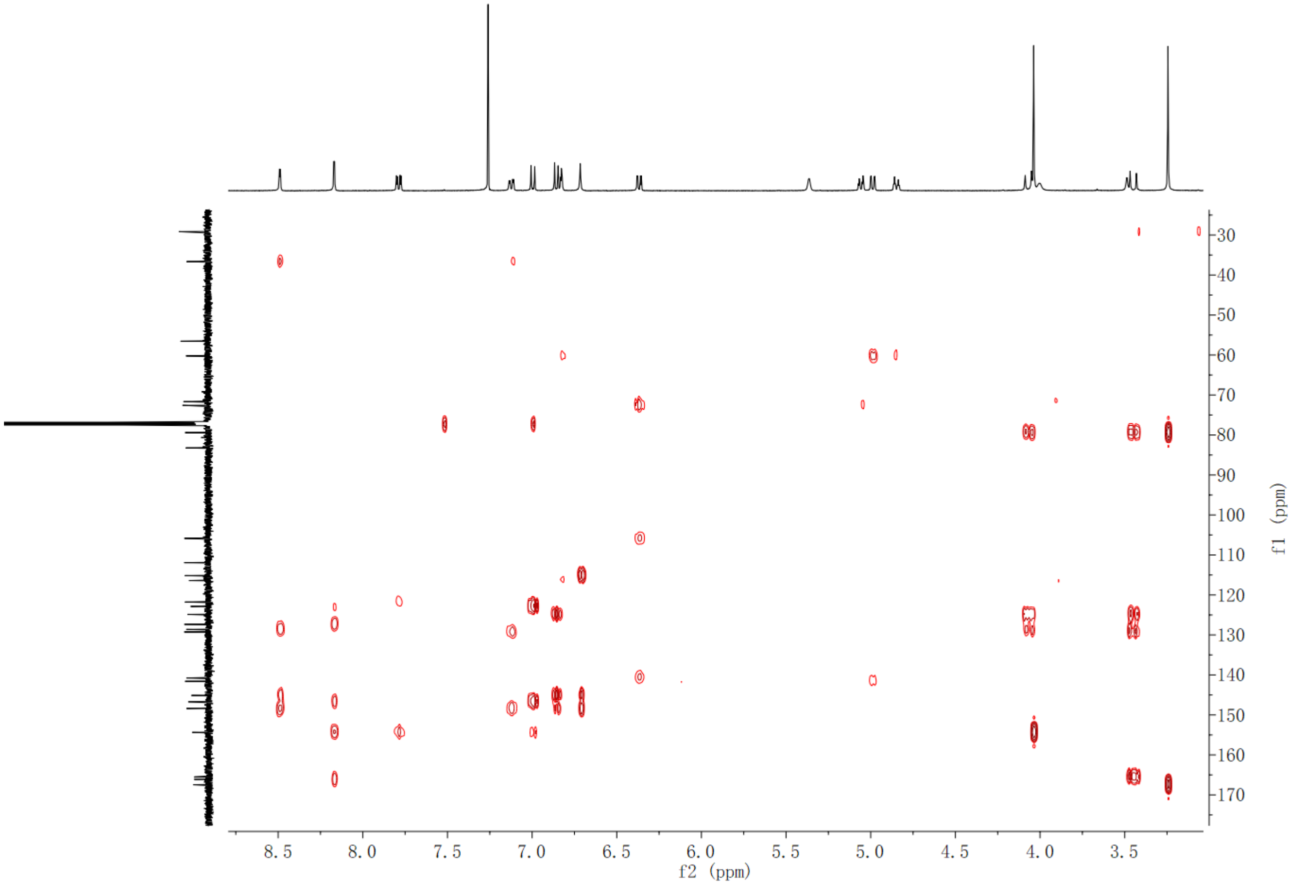

Figure S29. ^1H–^1H COSY spectrum of compound 3 (Recorded in $CDCl_3$)

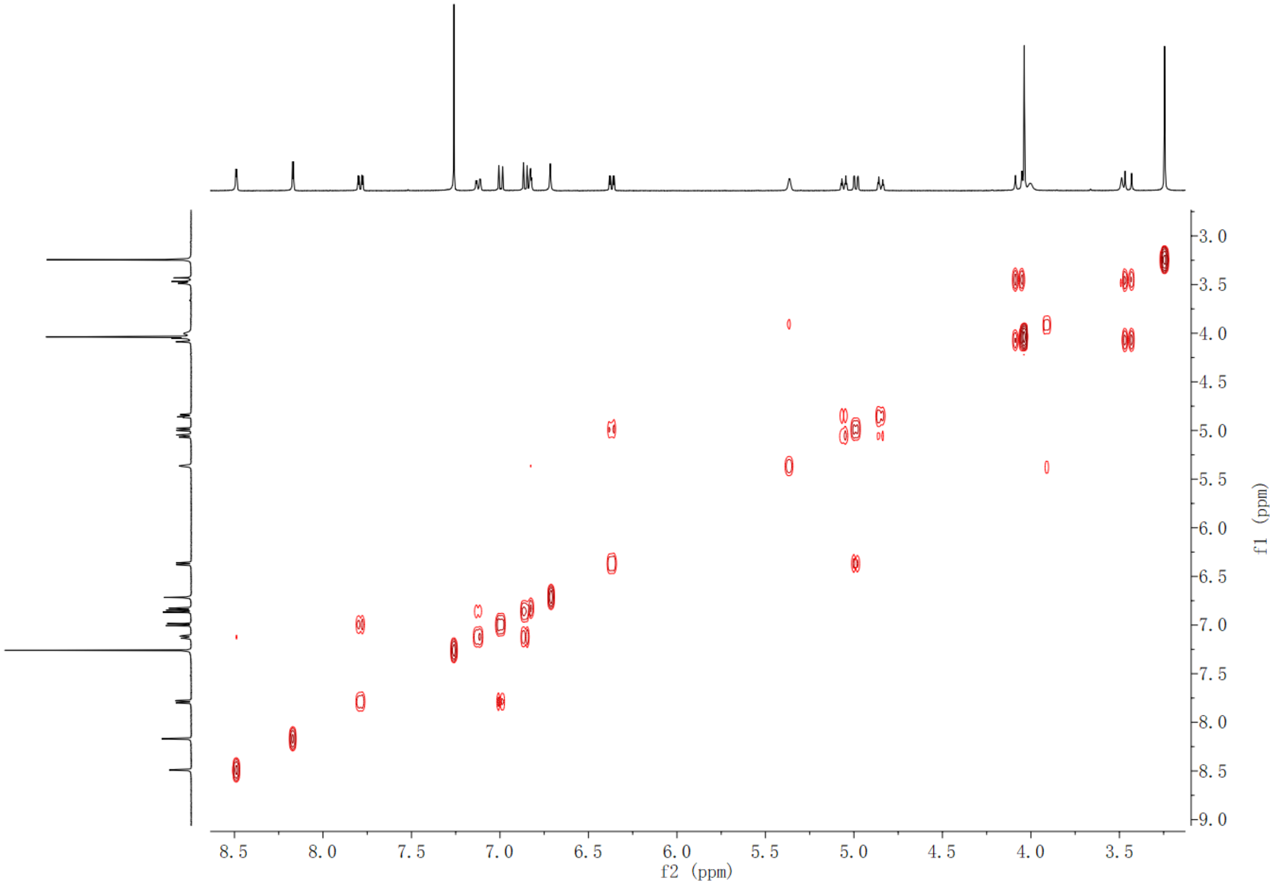

Figure S30. NOESY spectrum of compound 3 (Recorded in CDCl₃)

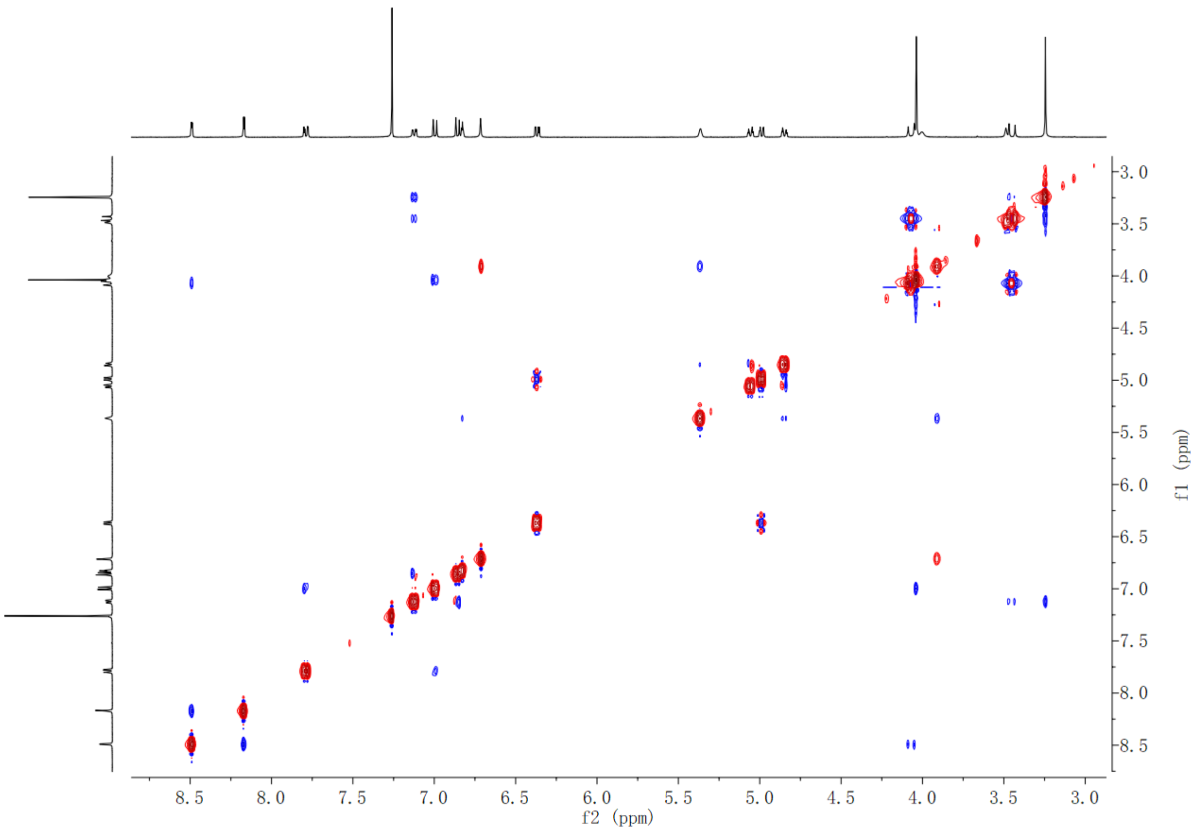

Figure S31. HRESIMS spectrum of compound 3

Mass Spectrum SmartFormula Report

Analysis Info

Acquisition D 2021/6/22 11:33:38

Analysis Name	H:\MS\3. LS5-12-3.d		
Method	Tune_pos_Mid.m	Operator	BDAL@DE
Sample Name		Instrument	micrOTOF II 8213750.1042 6

Comment

Acquisition Paramet

Source Type	ESI	Ion Polarity	Positive	Set Nebulizer	0.3 Bar
Focus	Not active			Set Dry Heater	200 °C
Scan Begin	50 m/z	Set Capillary	4500 V	Set Dry Gas	4.0 l/min
Scan End	3000 m/z	Set End Plate Offset	-500 V	Set Divert Valve	Waste

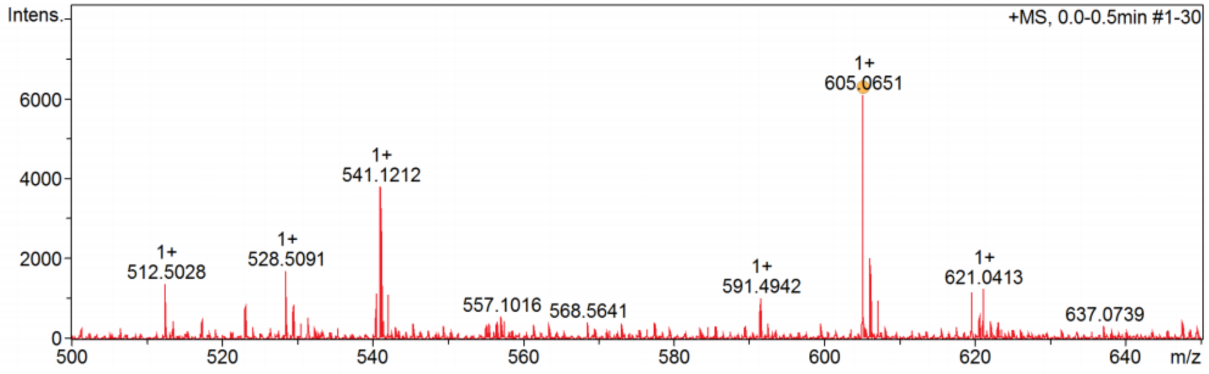

Meas. m/z	#	Ion Formula	m/z	err [ppm]	Mean err [ppm]	rdb	N-Rule	e¡¥ Conf	mSigma	Std I
605.065081	1	C27H22N2NaO9S2	605.065893	1.3	911.3	17.5	ok	even	7.1	10.1

Figure S32. UV spectrum of compound 3

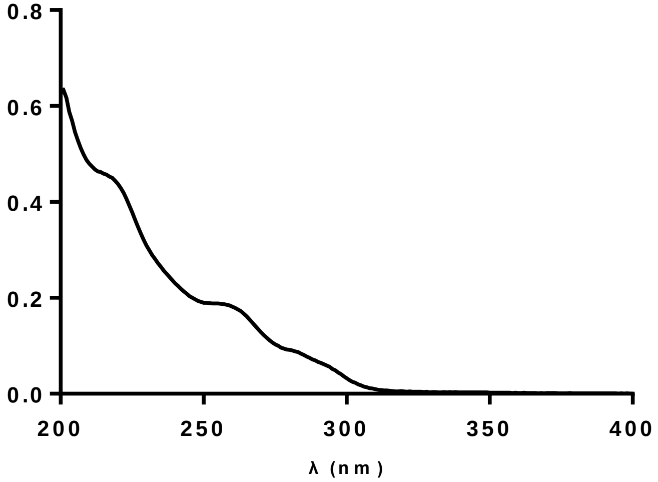

Figure S33. IR spectrum of compound 3

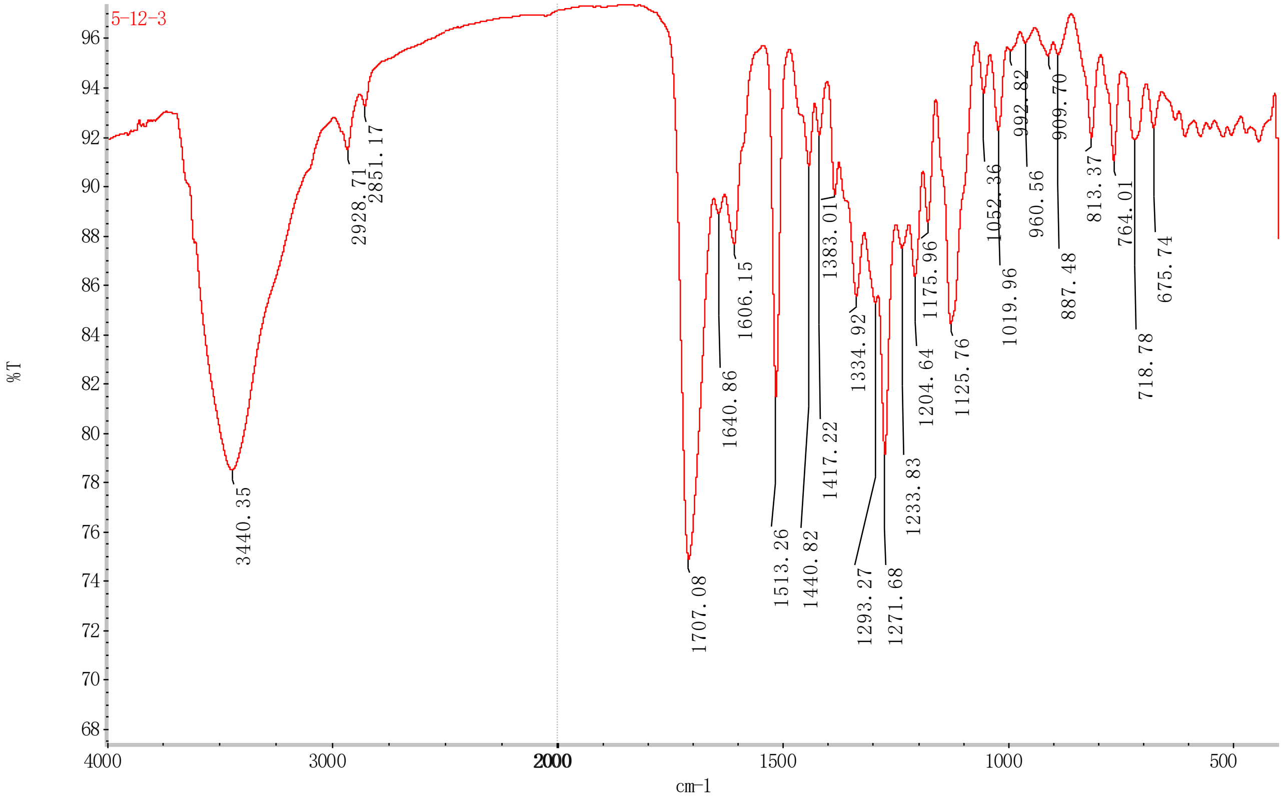

Figure S34. ^{1}H NMR spectrum of compound 4 (Recorded in CDCl₃)

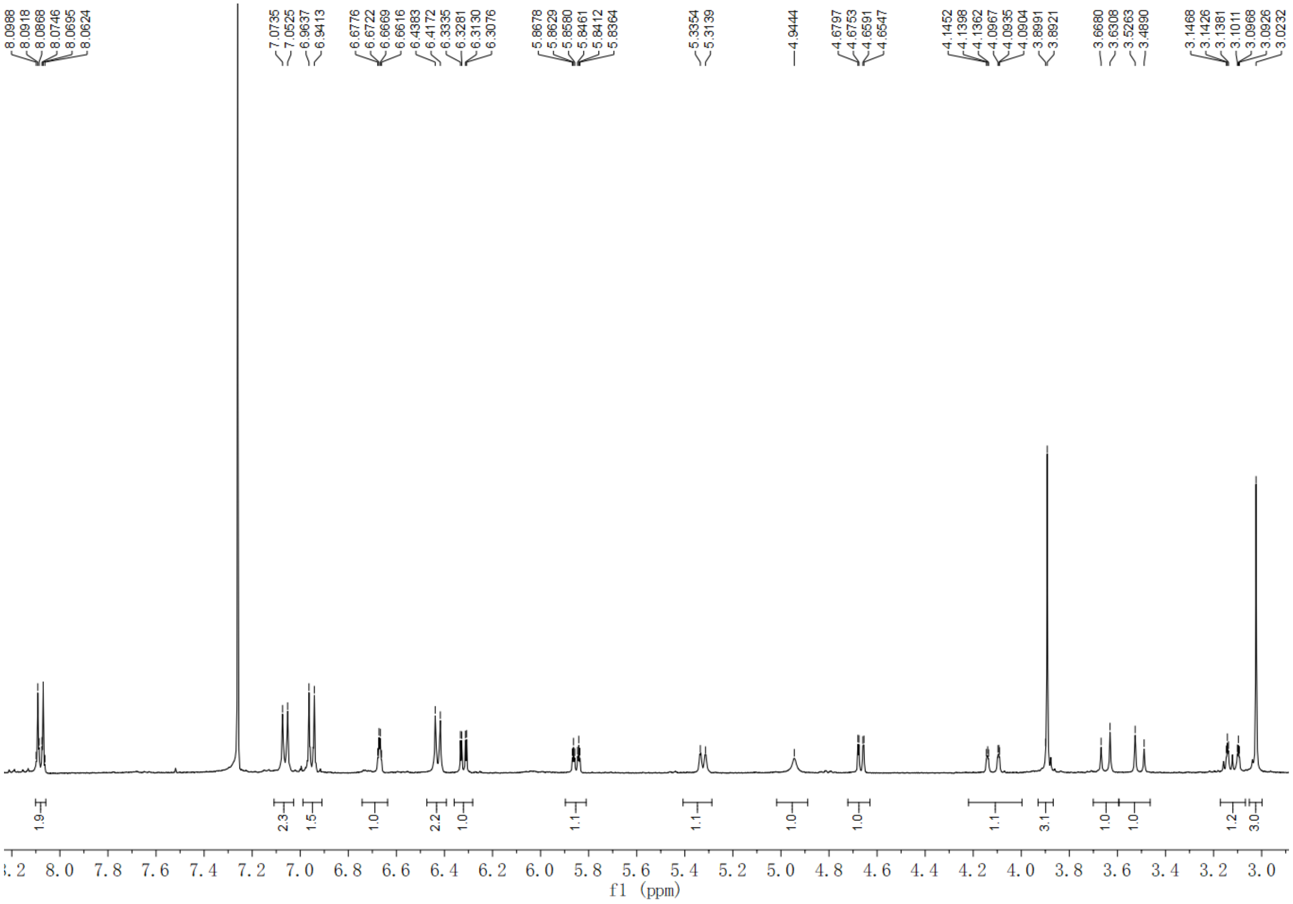

Figure S35. ^{13}C NMR and DEPT spectra of compound 4 (Recorded in $CDCl_3$)

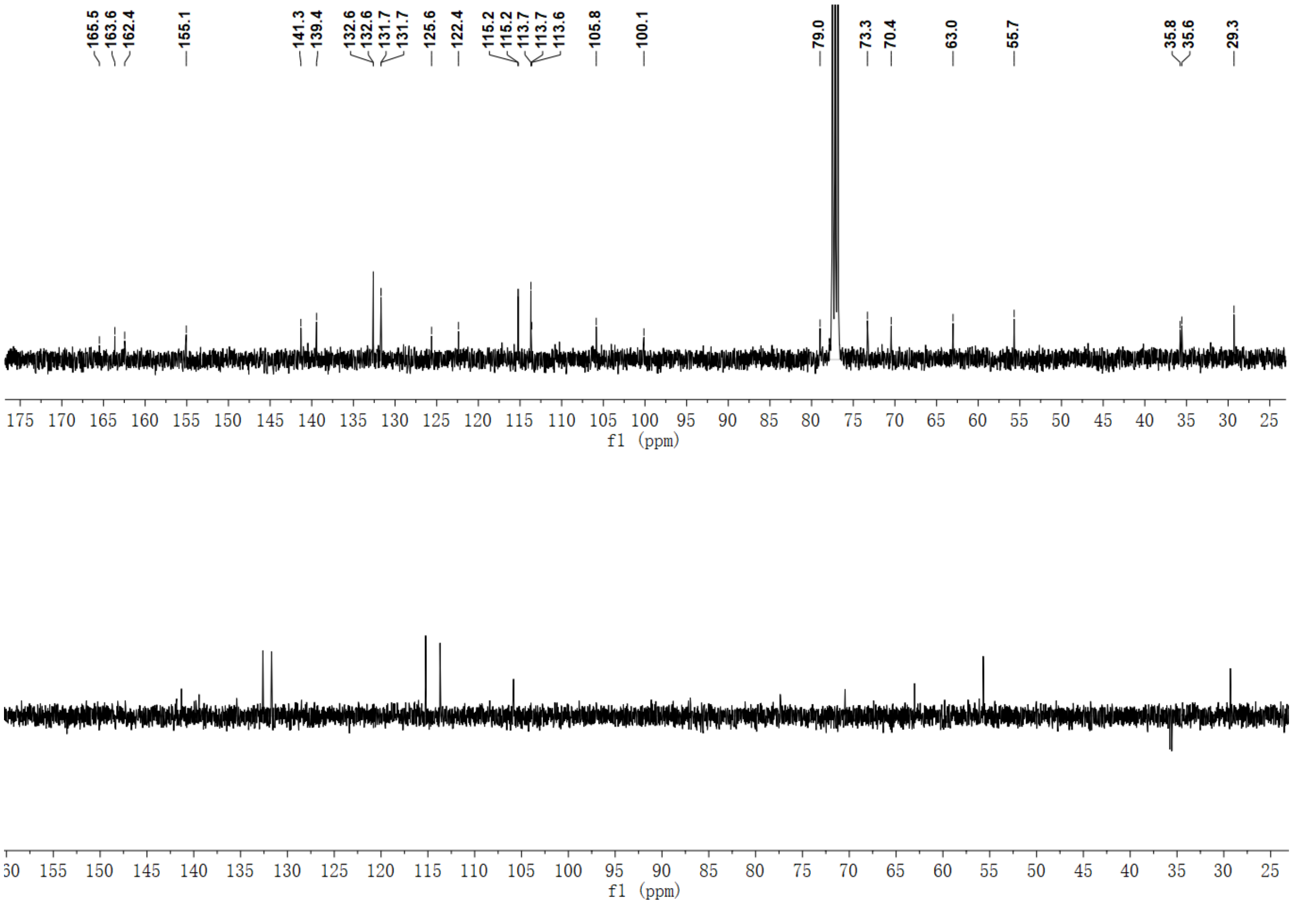

Figure S36. HSQC spectrum of compound 4 (Recorded in CDCl3)

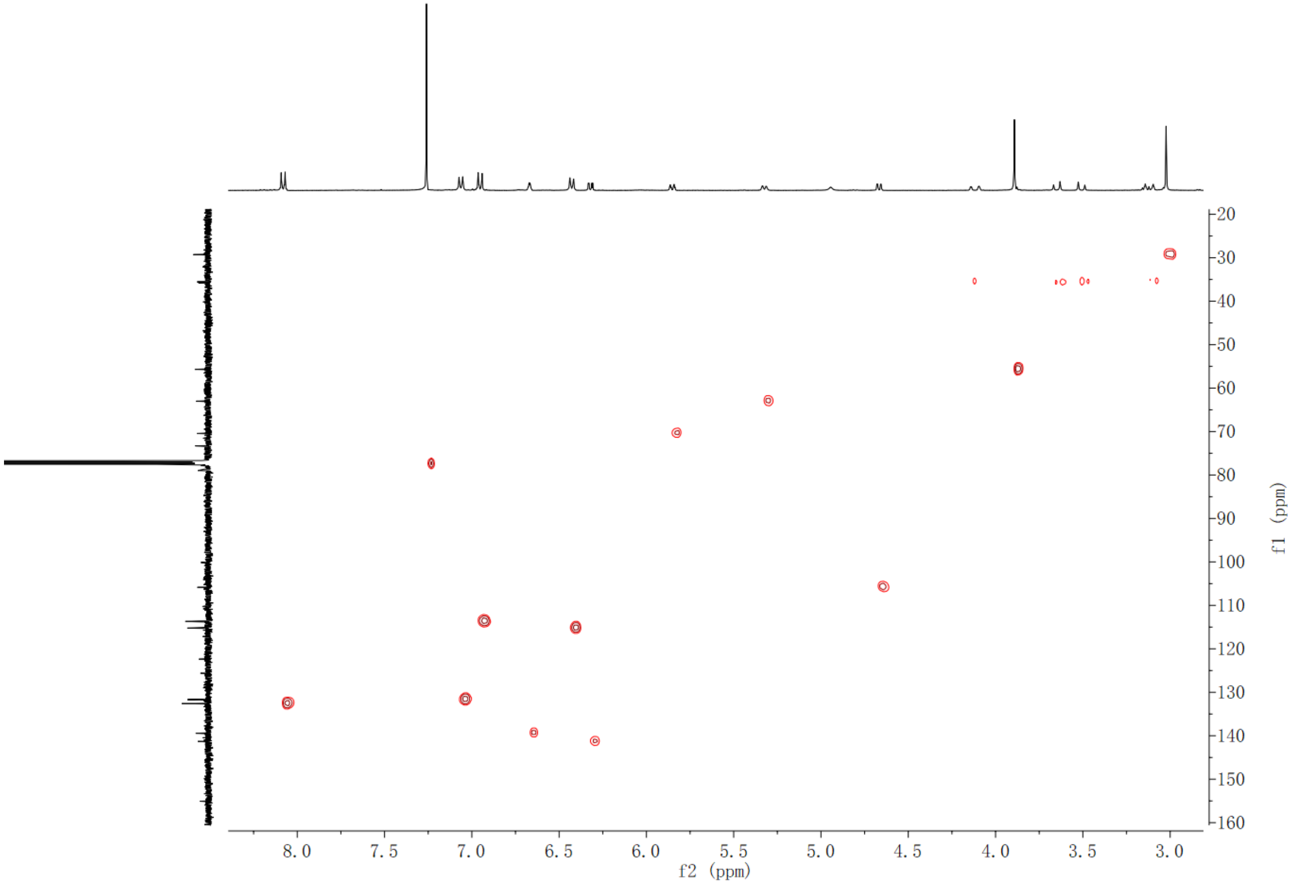

Figure S37. HMBC spectrum of compound 4 (Recorded in $CDCl_3$)

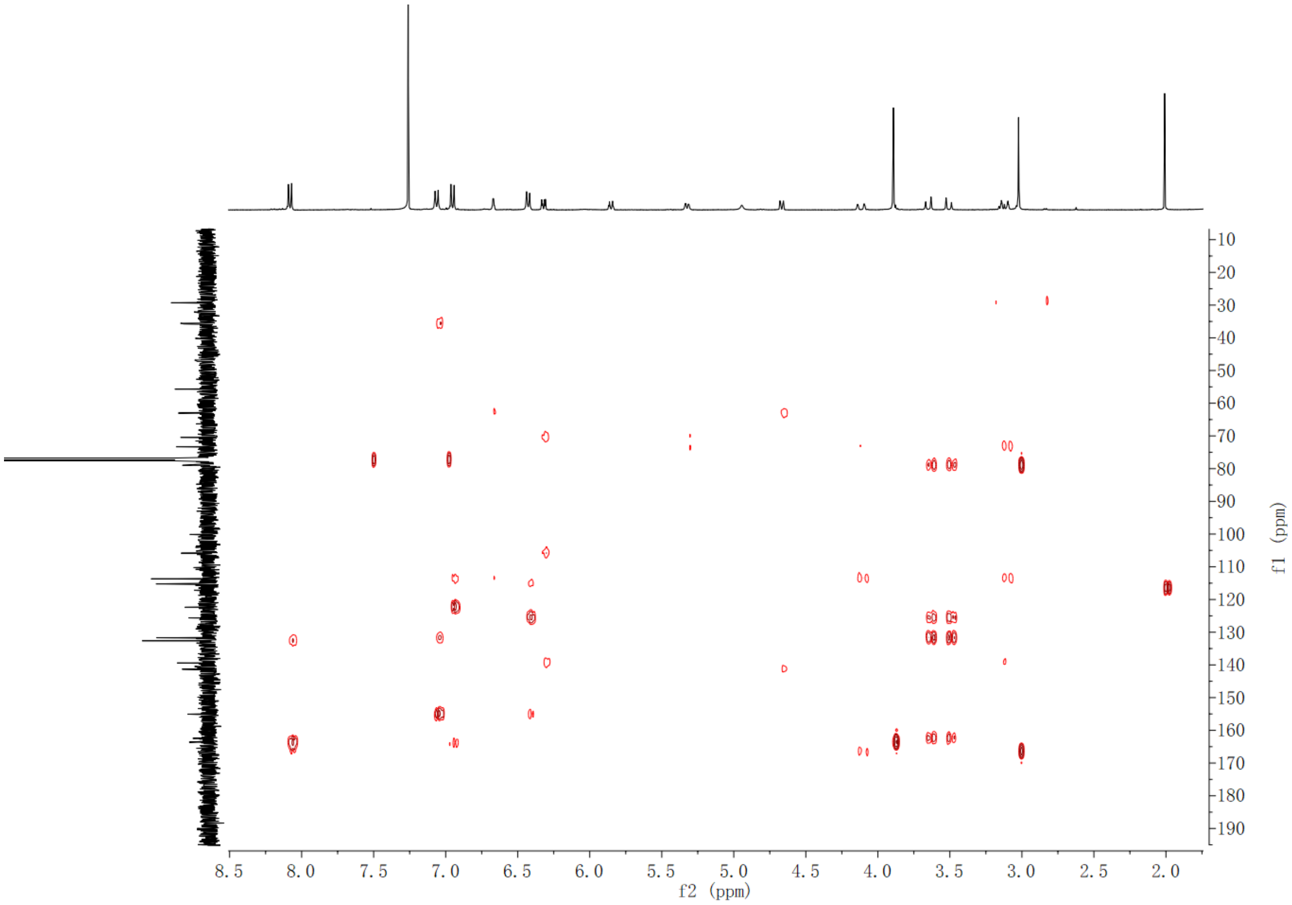

Figure S38. ^1H–^1H **COSY spectrum of compound 4 (Recorded in** $CDCl_3$**)**

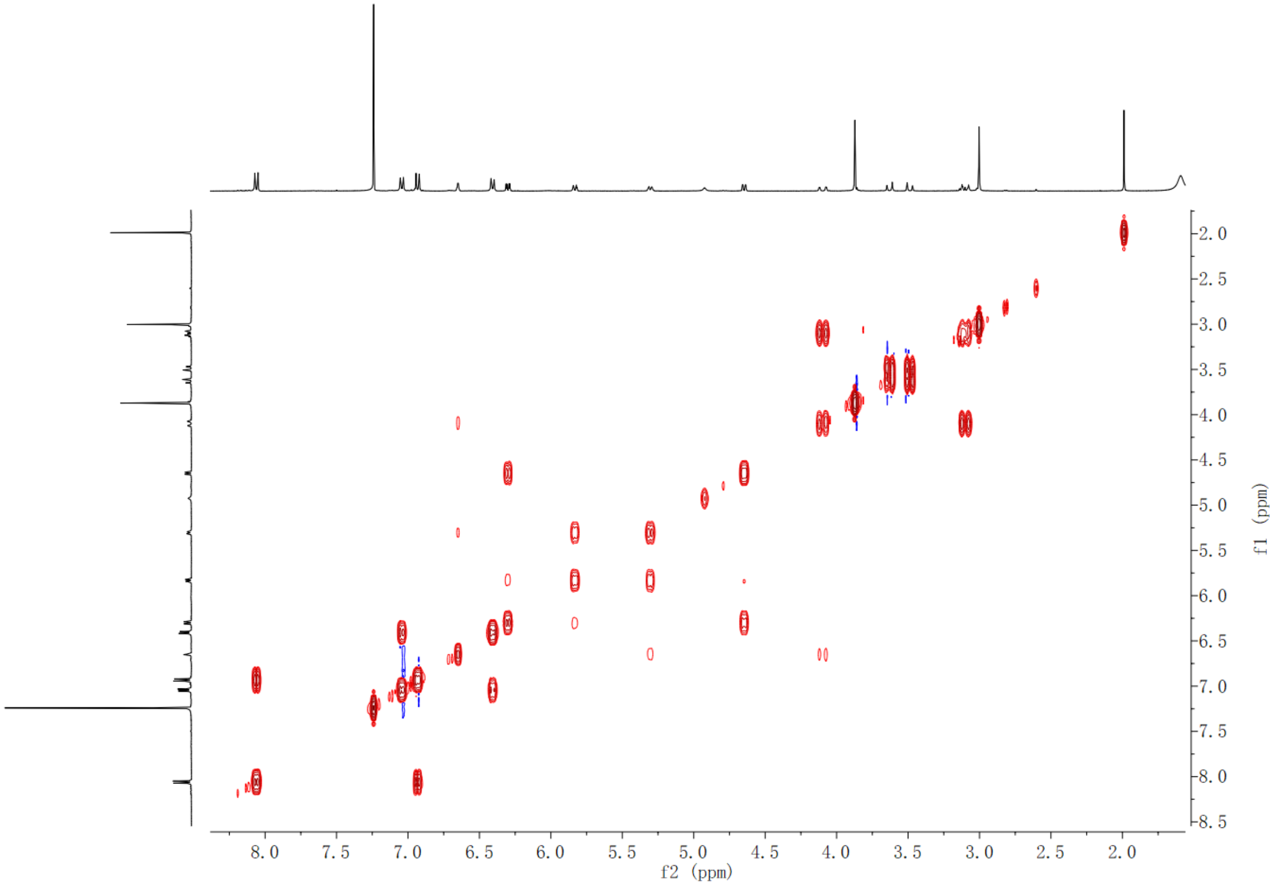

Figure S39. NOESY spectrum of compound 4 (Recorded in $CDCl_3$)

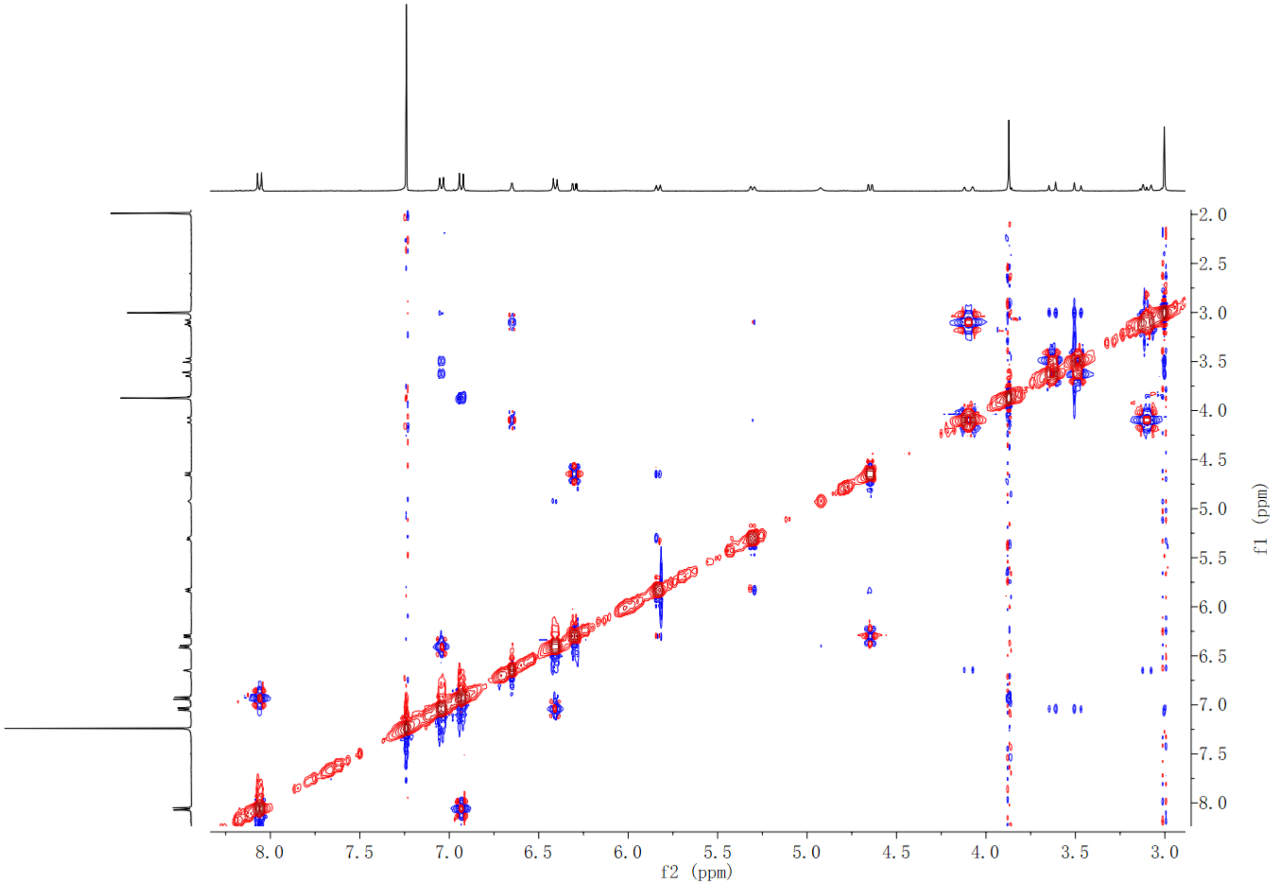

Figure S40. HRESIMS spectrum of compound 4

Mass Spectrum SmartFormula Report

Analysis Info		Acquisition D	2021/5/19 16:37:34
Analysis Name	H:\MS\4. LS 5-8-10.d		
Method	Tune_pos_Mid.m	Operator	BDAL@DE
Sample Name		Instrument	micrOTOF II 8213750.10426
Comment			

Acquisition Paramet

Source Type	ESI	Ion Polarity	Positive	Set Nebulizer	0.3 Bar
Focus	Not active			Set Dry Heater	200 °C
Scan Begin	100 m/z	Set Capillary	4500 V	Set Dry Gas	4.0 l/min
Scan End	1500 m/z	Set End Plate Offset	-500 V	Set Divert Valve	Waste

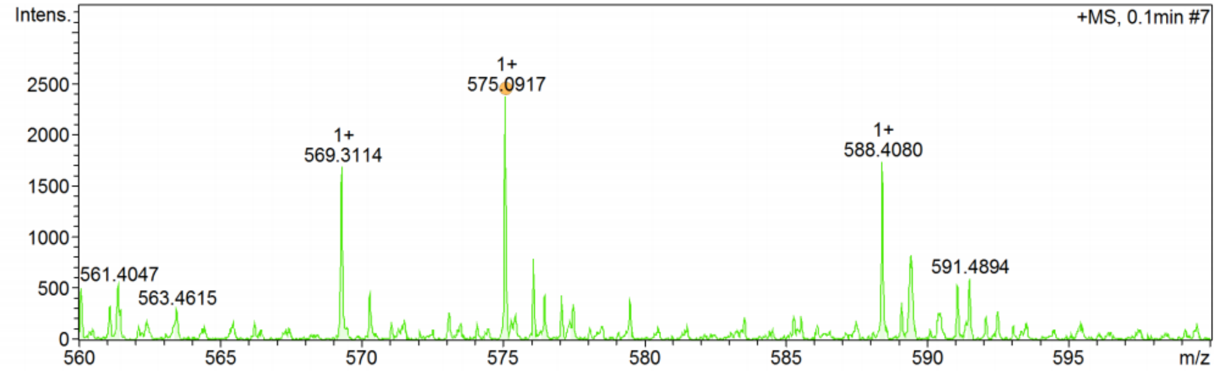

Meas. m/z	#	Ion Formula	m/z	err [ppm]	Mean err [ppm]	rdb	N-Rule	e¡¥ Conf	mSigma	Std I
575.091716	1	C27H24N2NaO7S2	575.091714	-0.0	1702.6	16.5	ok	even	22.7	33.6

Figure S41. UV spectrum of compound 4

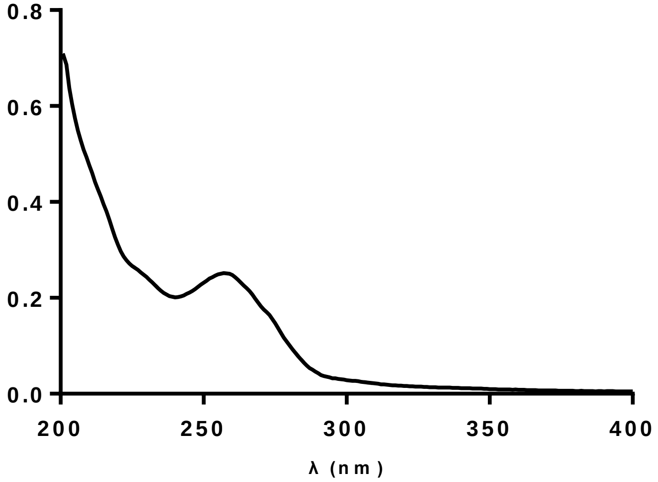

Figure S42. IR spectrum of compound 4

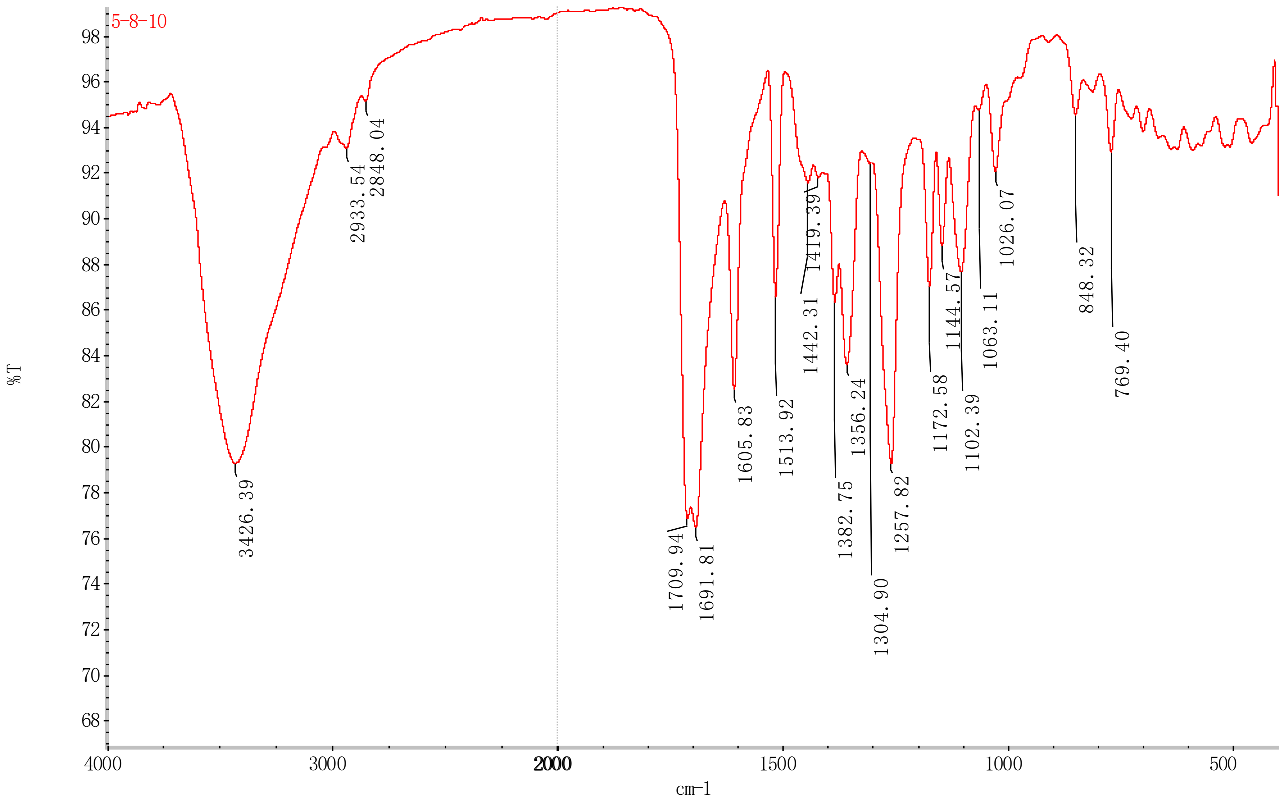

Figure S43. ¹H NMR spectrum of compound 5 (Recorded in CDCl₃)

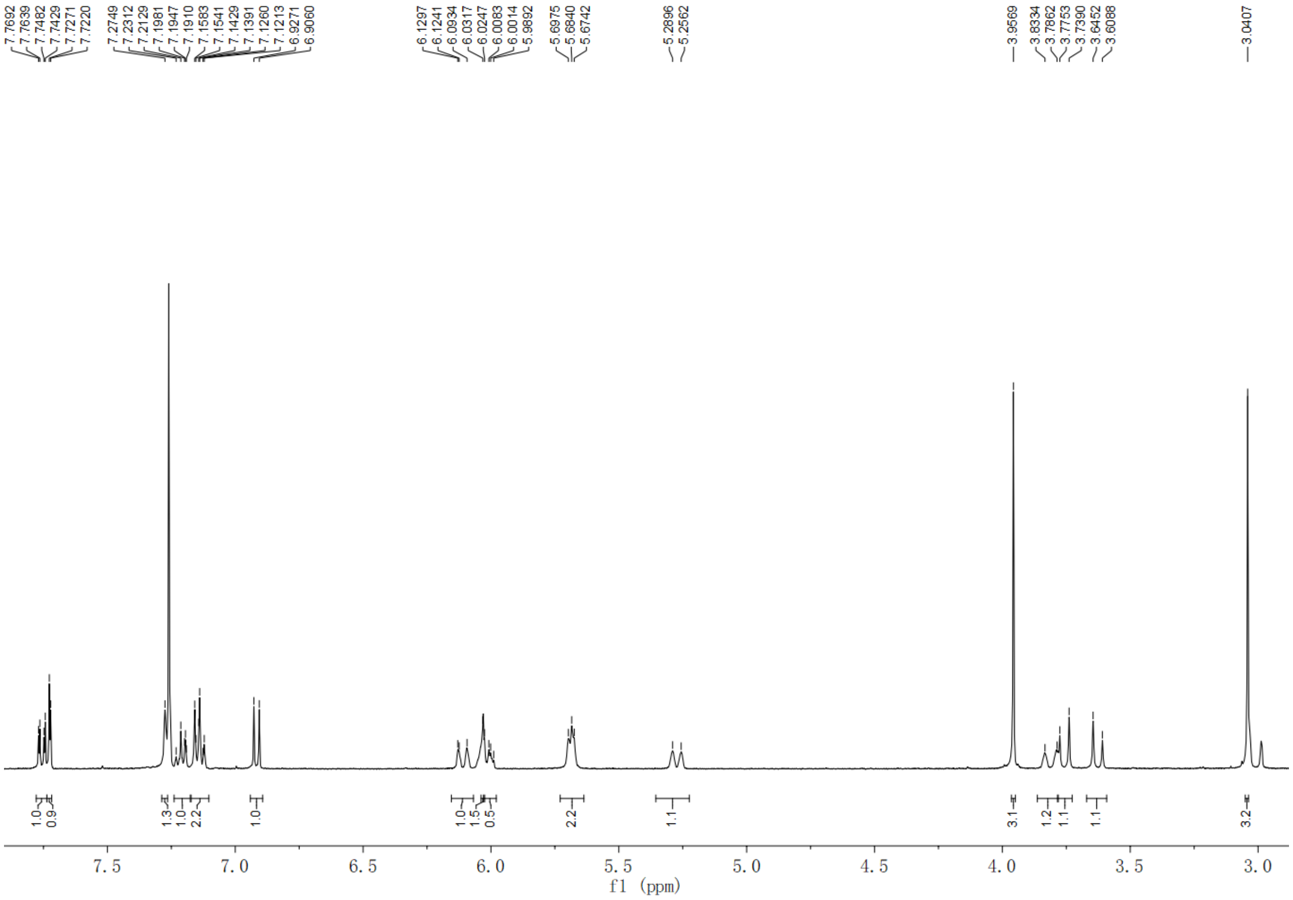

Figure S44. ^{13}C NMR and DEPT spectra of compound 5 (Recorded in $CDCl_3$)

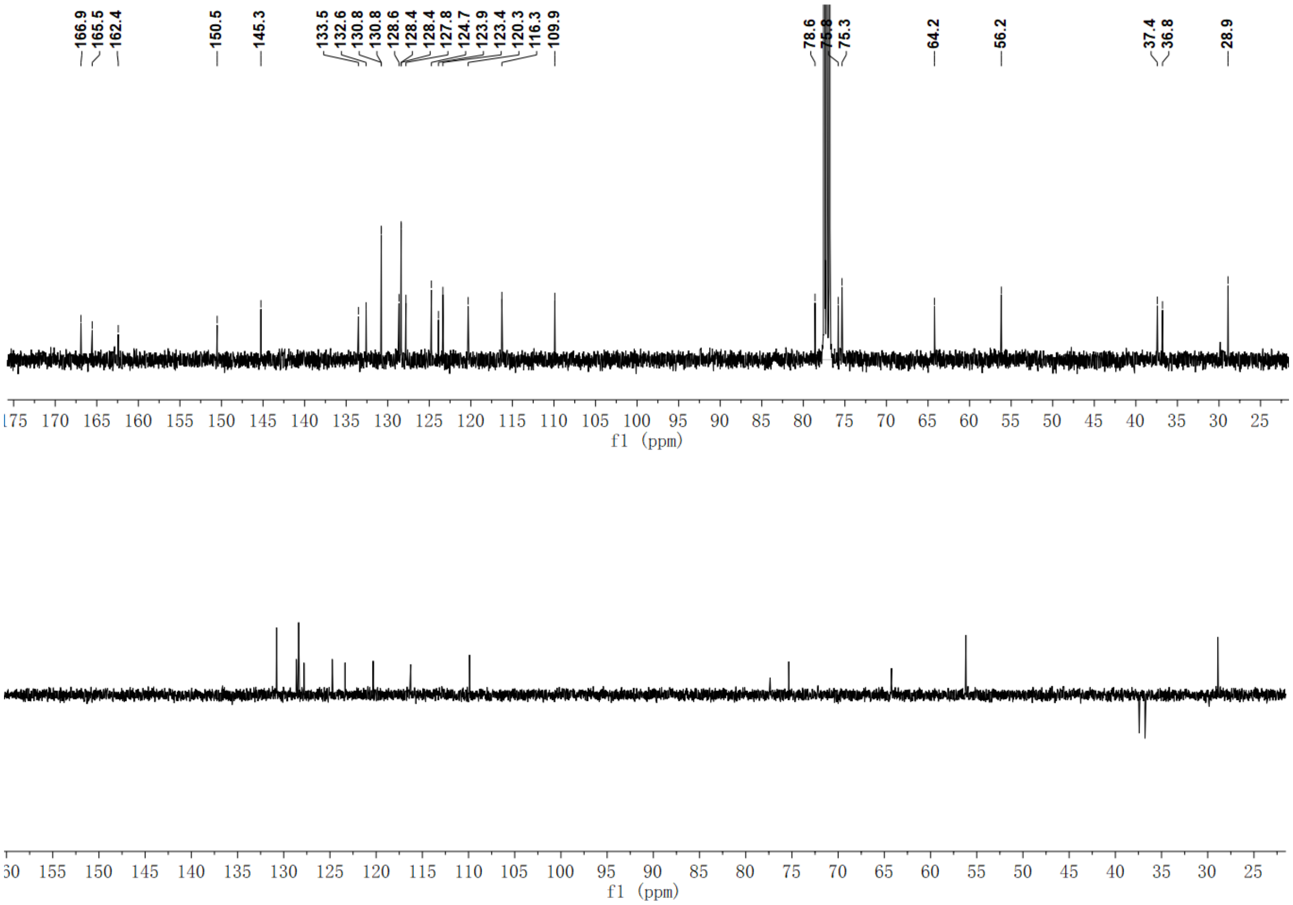

Figure S45. HSQC spectrum of compound 5 (Recorded in $CDCl_3$)

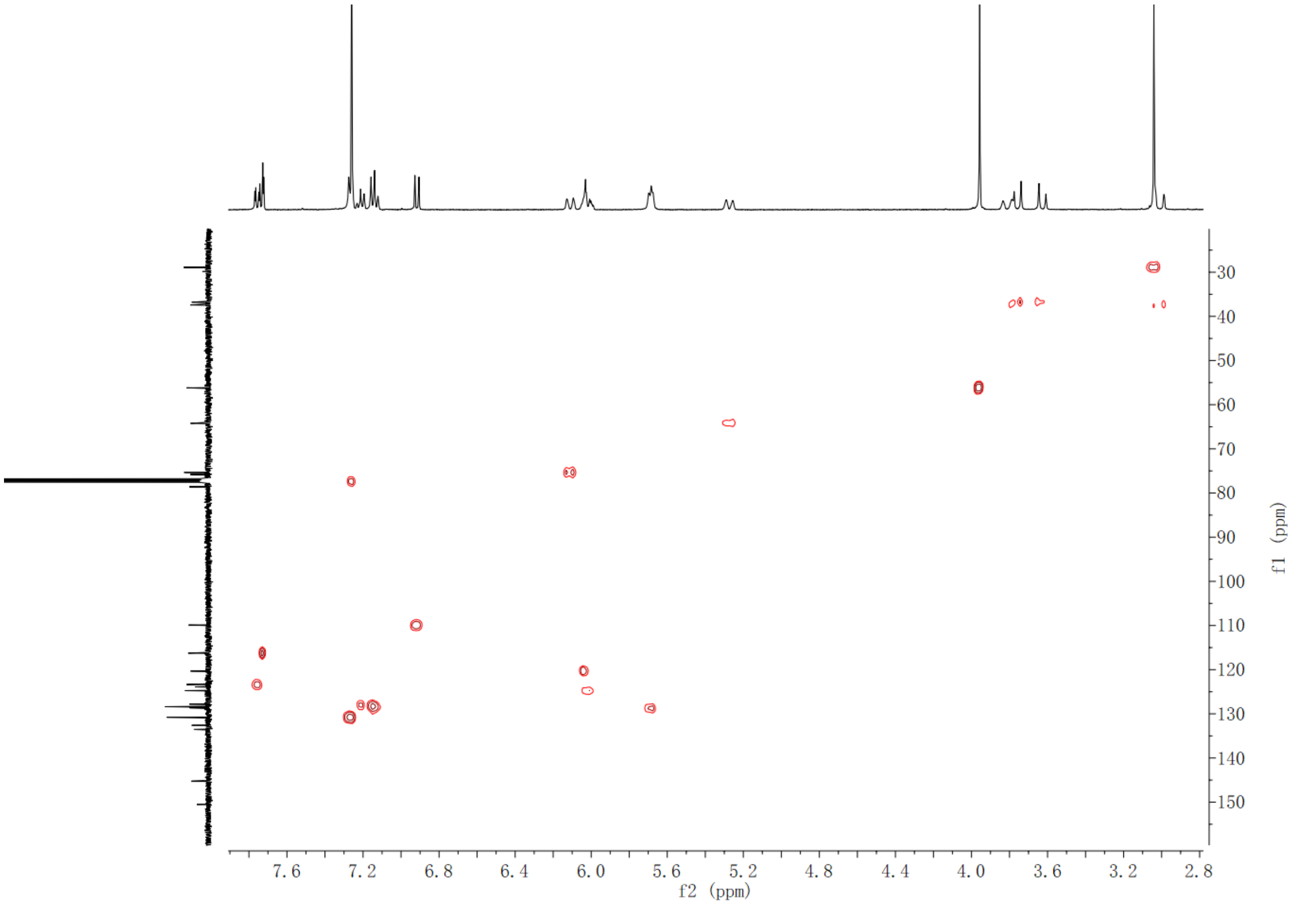

Figure S46. HMBC spectrum of compound 5 (Recorded in CDCl₃)

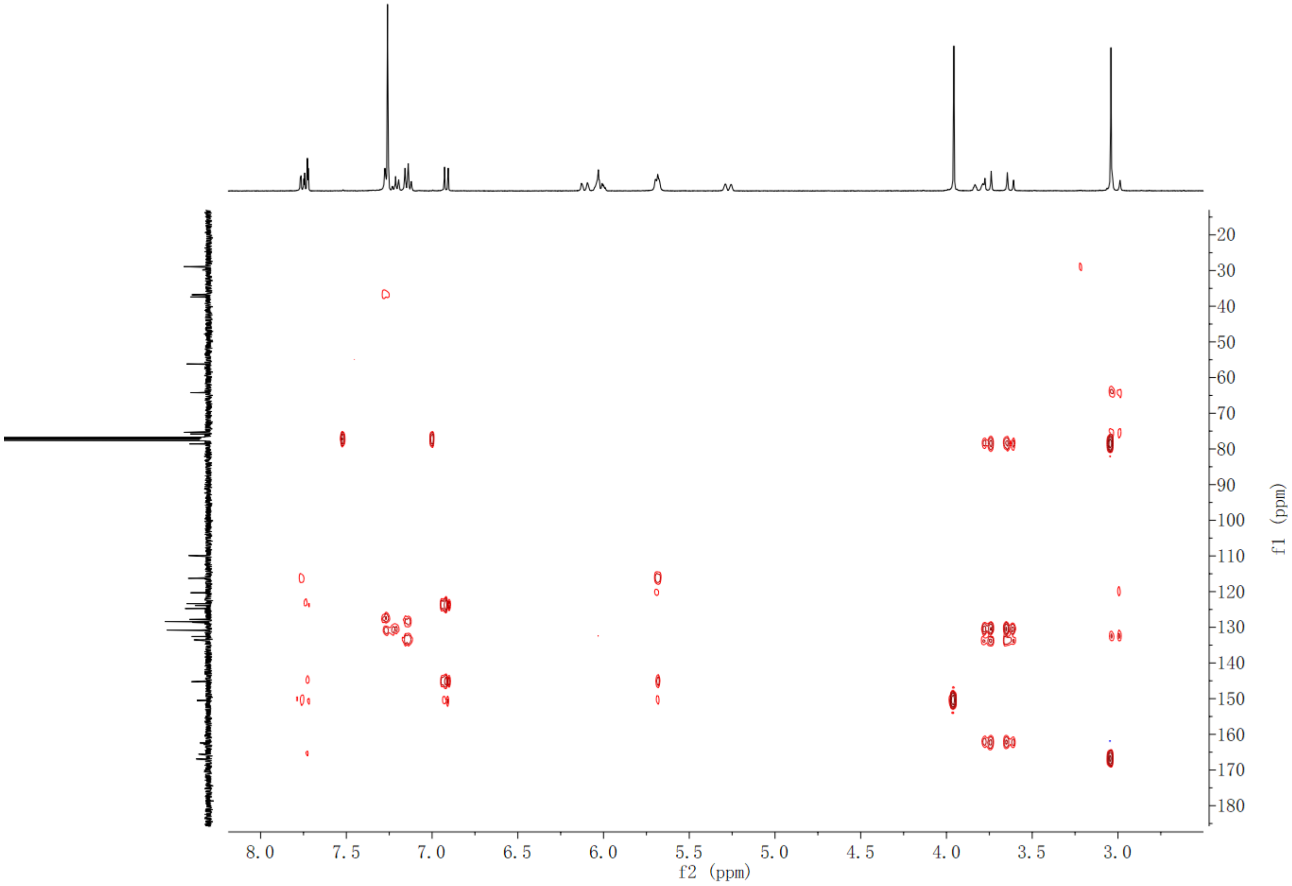

Figure S47. ^{1}H–^{1}H **COSY spectrum of compound 5 (Recorded in** $CDCl_3$**)**

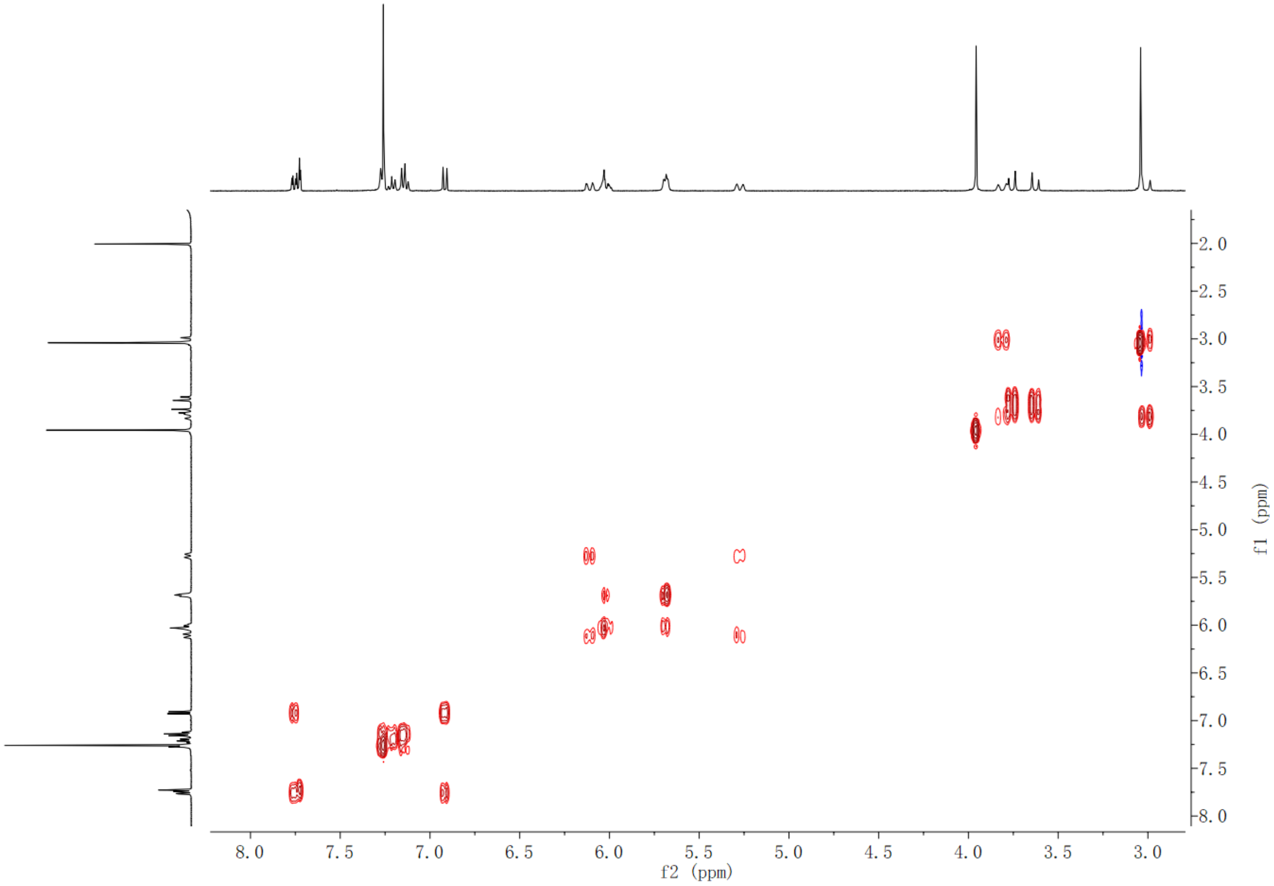

Figure S48. NOESY spectrum of compound 5 (Recorded in CDCl₃)

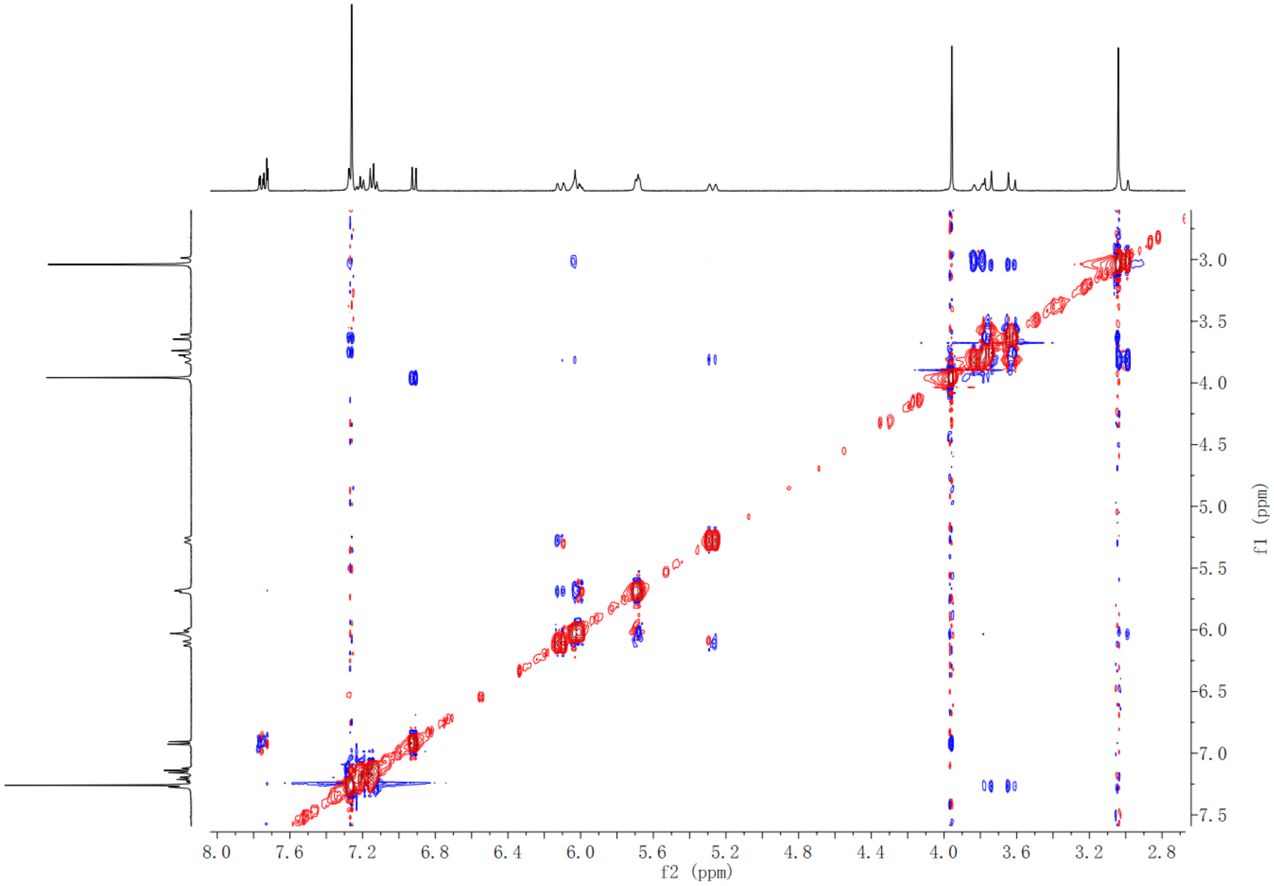

Figure S49. HRESIMS spectrum of compound 5

Mass Spectrum SmartFormula Report

Analysis Info

Analysis Name H:\MS\5. LS-5-5-4.d

Method Tune_pos_Mid.m

Sample Name

Comment

Acquisition D 2021/5/27 9:29:50

Operator BDAL@DE

Instrument micrOTOF II 8213750.10426

Acquisition Paramet

Source Type	ESI	Ion Polarity	Positive	Set Nebulizer	0.3 Bar
Focus	Not active			Set Dry Heater	200 °C
Scan Begin	100 m/z	Set Capillary	4500 V	Set Dry Gas	4.0 l/min
Scan End	1600 m/z	Set End Plate Offset	-500 V	Set Divert Valve	Waste

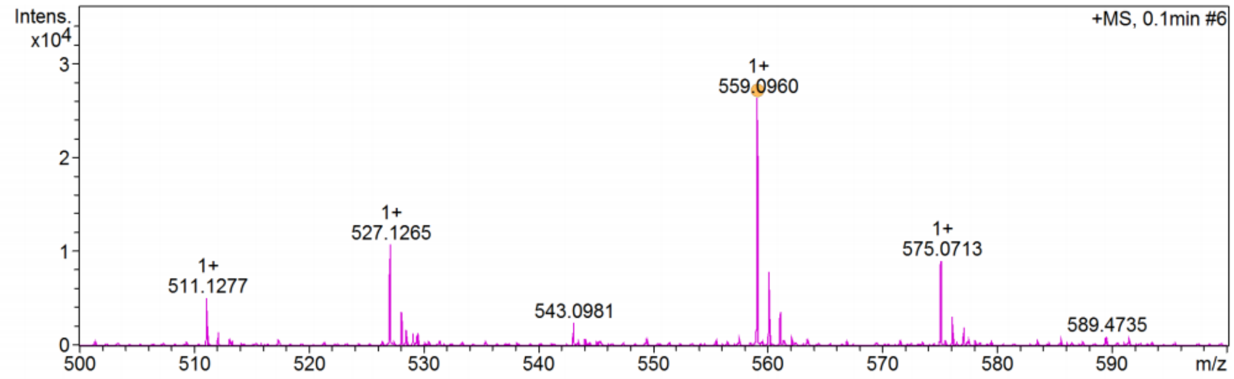

Meas. m/z	#	Ion Formula	m/z	err [ppm]	Mean err [ppm]	rdb	N-Rule	e¡¥ Conf	mSigma	Std I
559.095951	1	C27H24N2NaO6S2	559.096799	1.5	987.8	16.5	ok	even	11.7	23.2

Figure S50. UV spectrum of compound 5

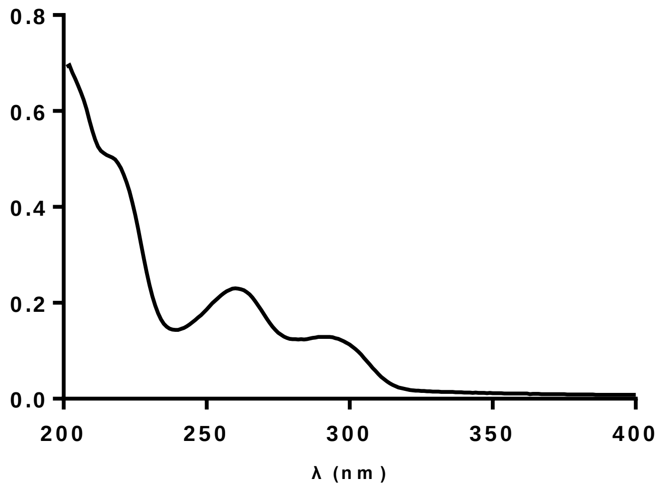

Figure S51. IR spectrum of compound 5

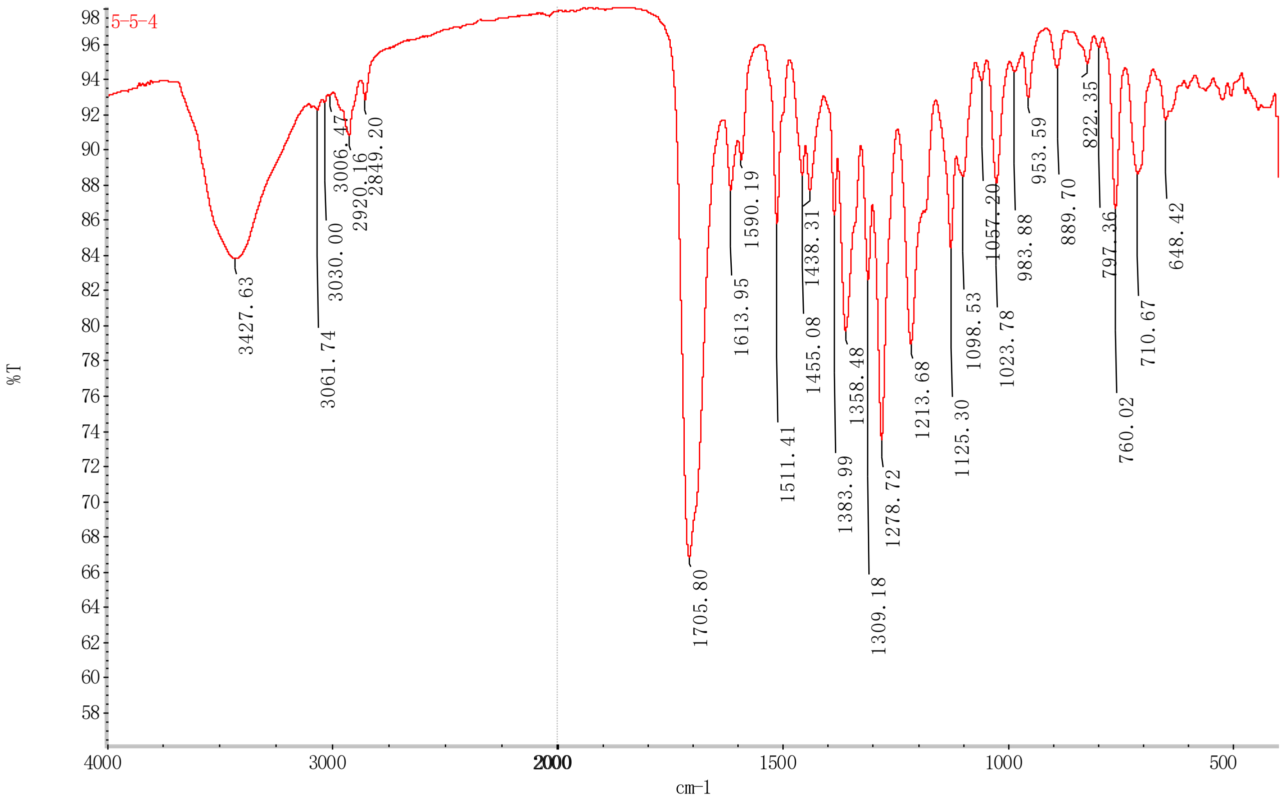

Figure S52. ^{1}H NMR spectrum of compound 6 (Recorded in $CDCl_3$)

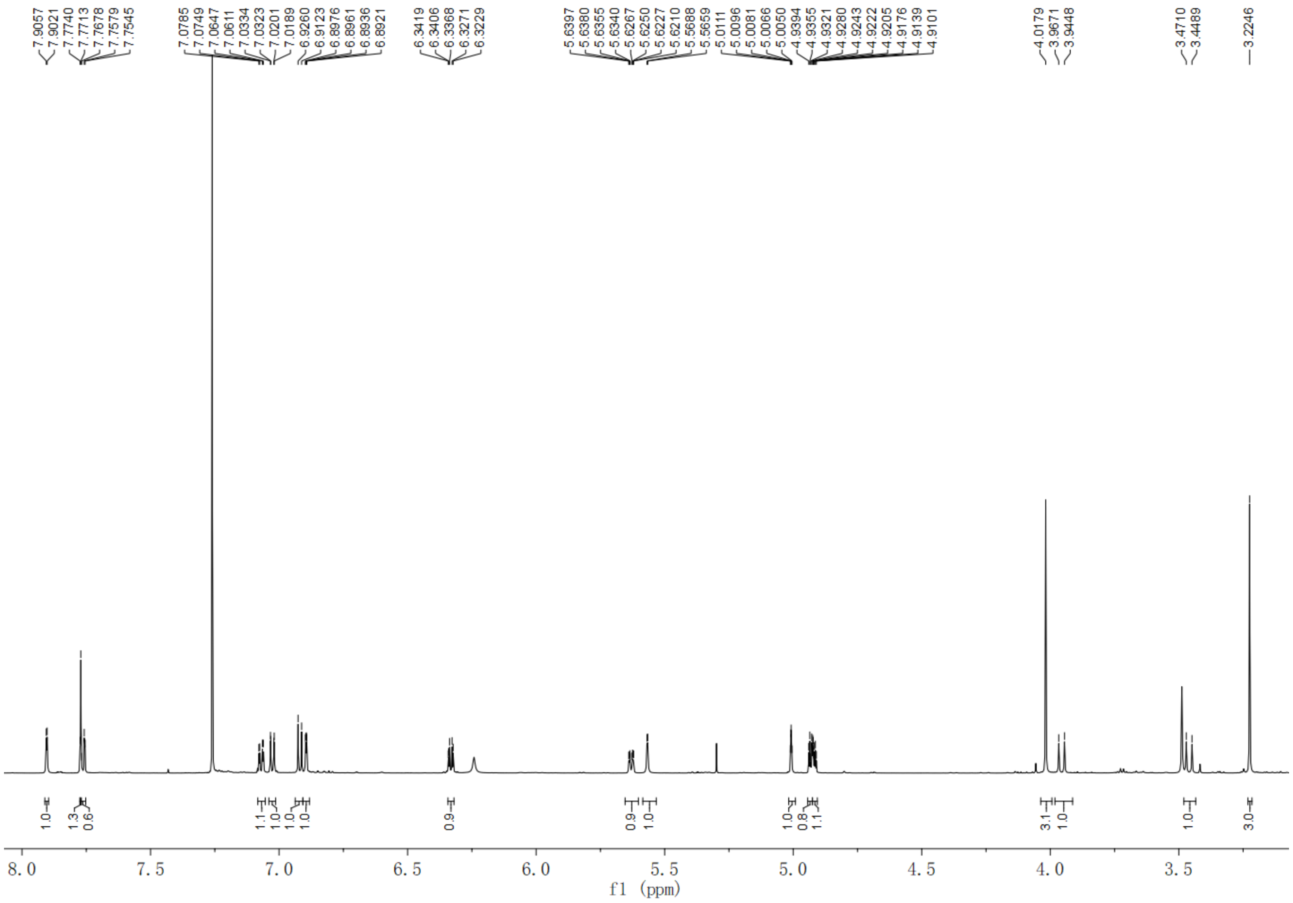

Figure S53. ^{13}**C NMR and DEPT spectra of compound 6 (Recorded in CDCl**$_3$**)**

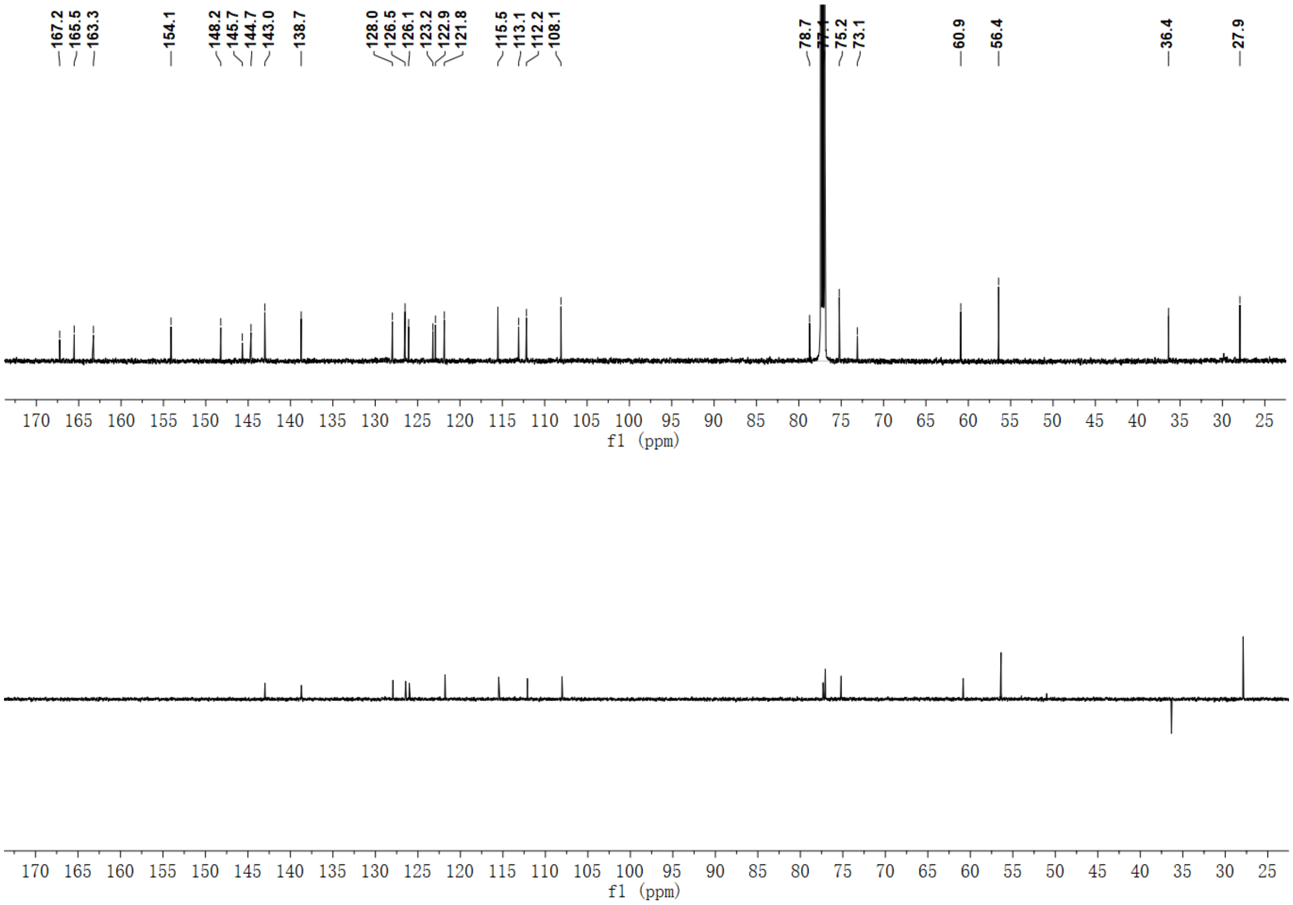

Figure S54. HSQC spectrum of compound 6 (Recorded in $CDCl_3$)

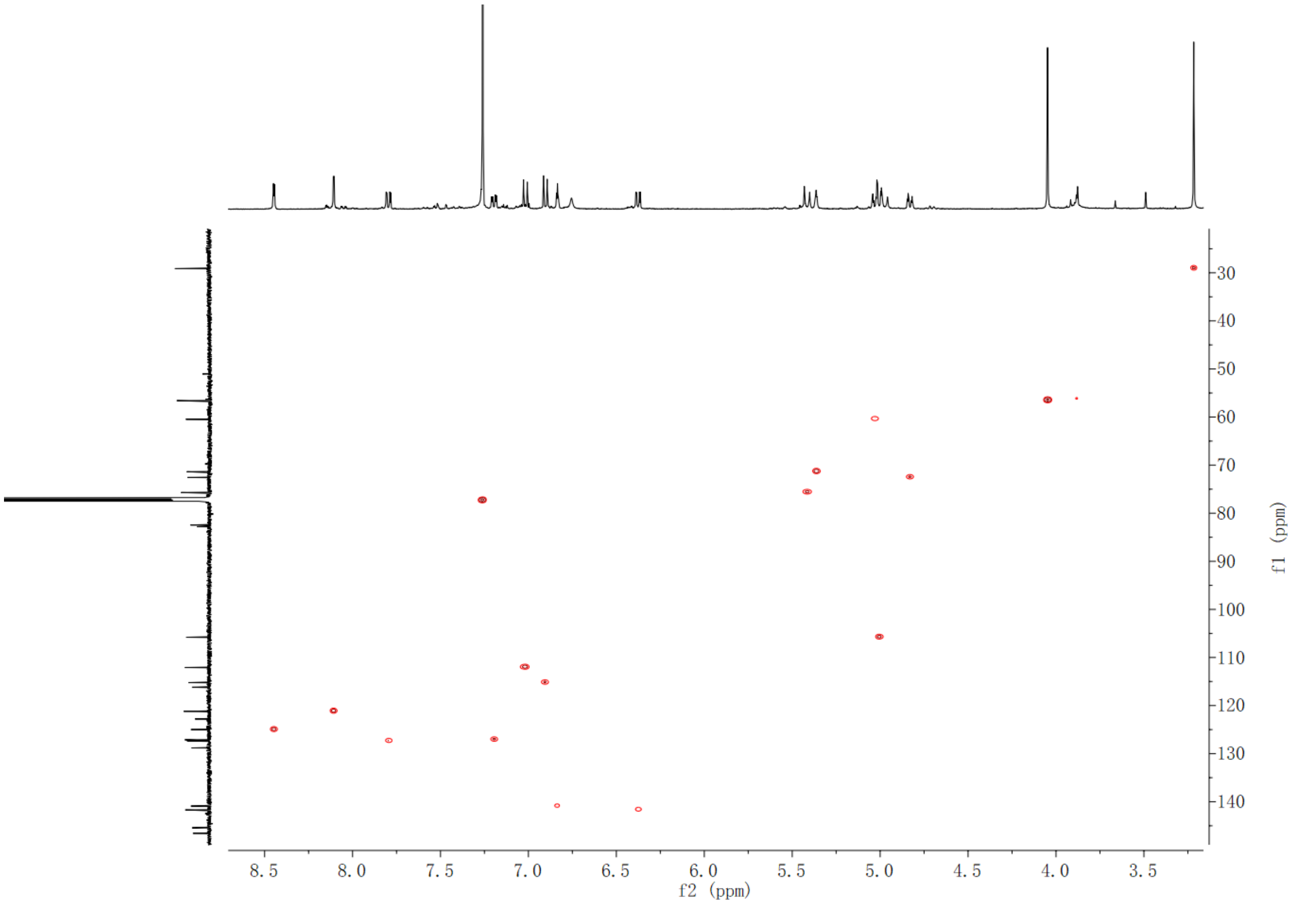

Figure S55. HMBC spectrum of compound 6 (Recorded in $CDCl_3$)

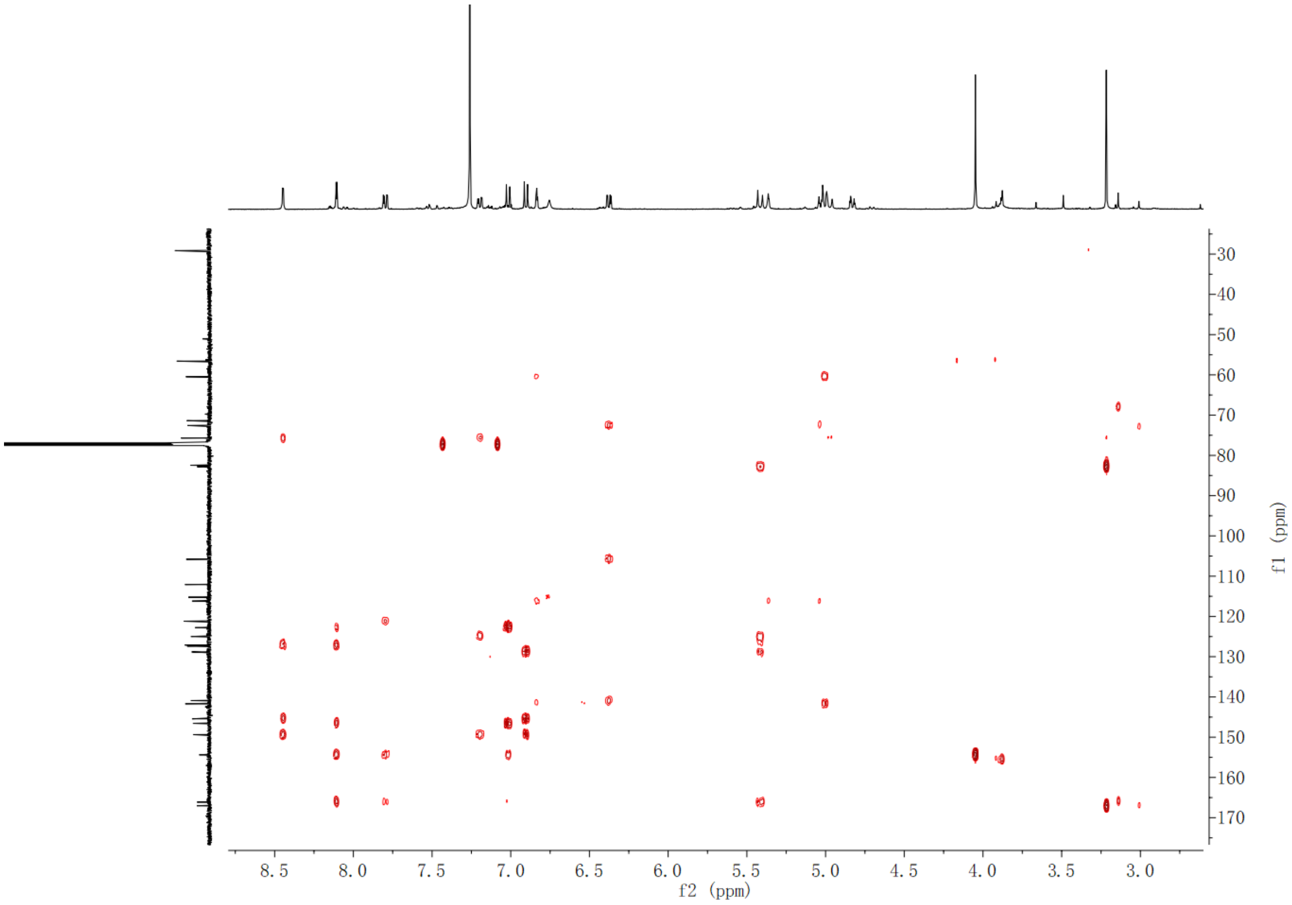

Figure S56. ^1H–^1H **COSY spectrum of compound 6 (Recorded in** $CDCl_3$**)**

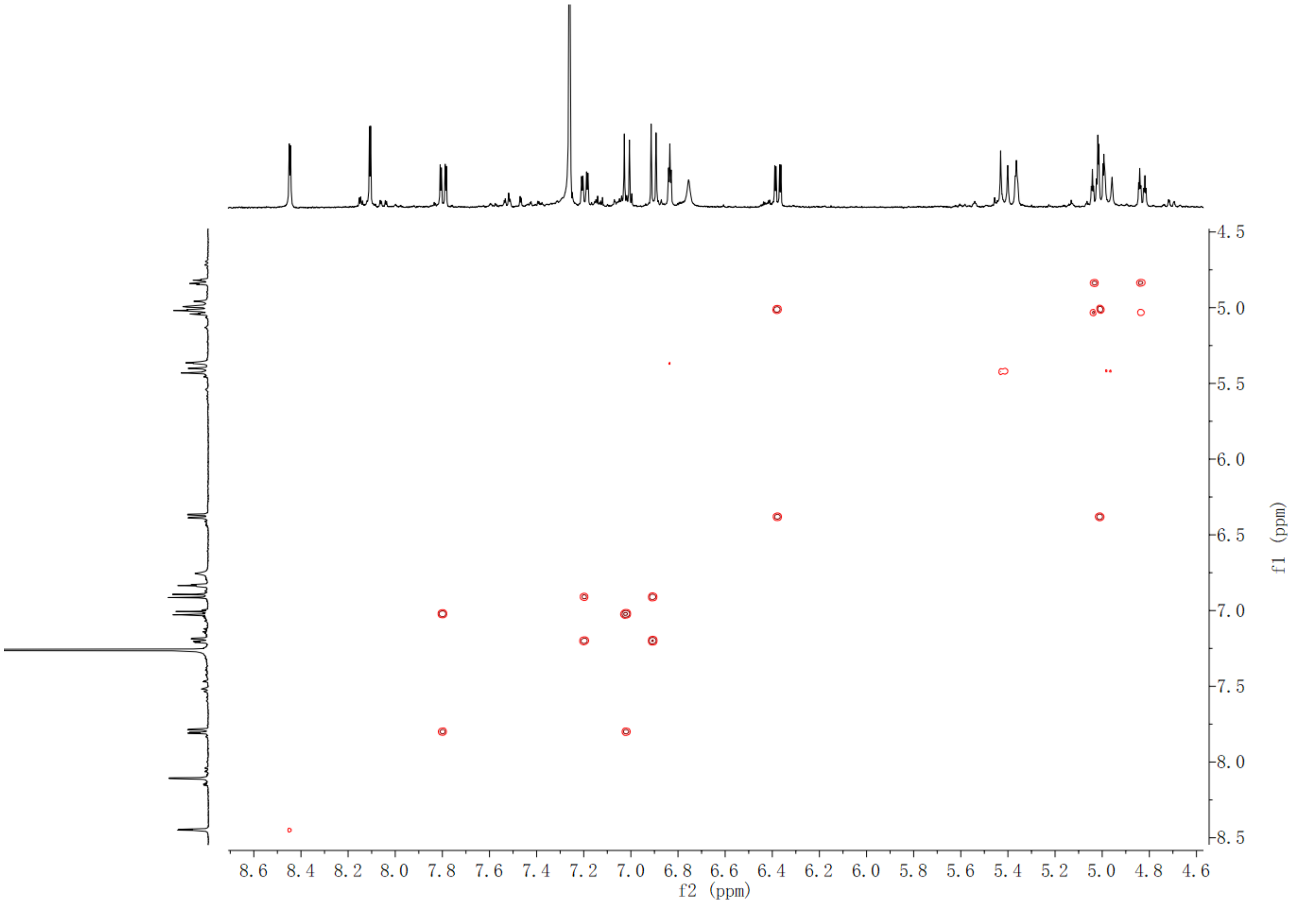

Figure S57. NOESY spectrum of compound 6 (Recorded in CDCl₃)

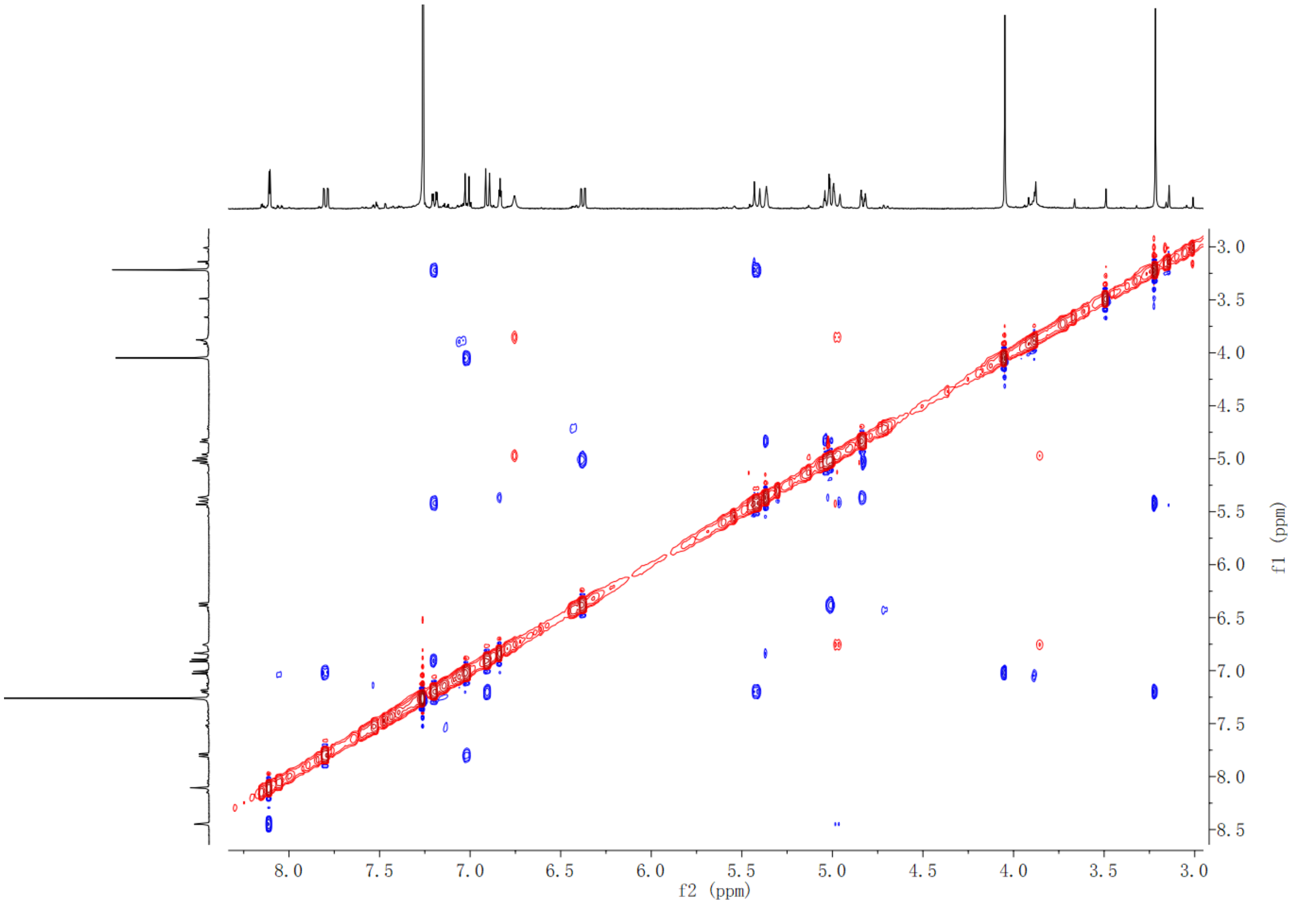

Figure S58. HRESIMS spectrum of compound 6

Mass Spectrum SmartFormula Report

Analysis Info

Acquisition D 2021/9/23 11:41:43

Analysis Name H:\MS\6. LS5-12-6.d
Method Tune_pos_Mid.m
Sample Name

Operator BDAL@DE
Instrument micrOTOF II 8213750.10426

Comment

Acquisition Paramet

Source Type	ESI	Ion Polarity	Positive	Set Nebulizer	0.3 Bar
Focus	Not active			Set Dry Heater	200 °C
Scan Begin	100 m/z	Set Capillary	4500 V	Set Dry Gas	4.0 l/min
Scan End	2400 m/z	Set End Plate Offset	-500 V	Set Divert Valve	Waste

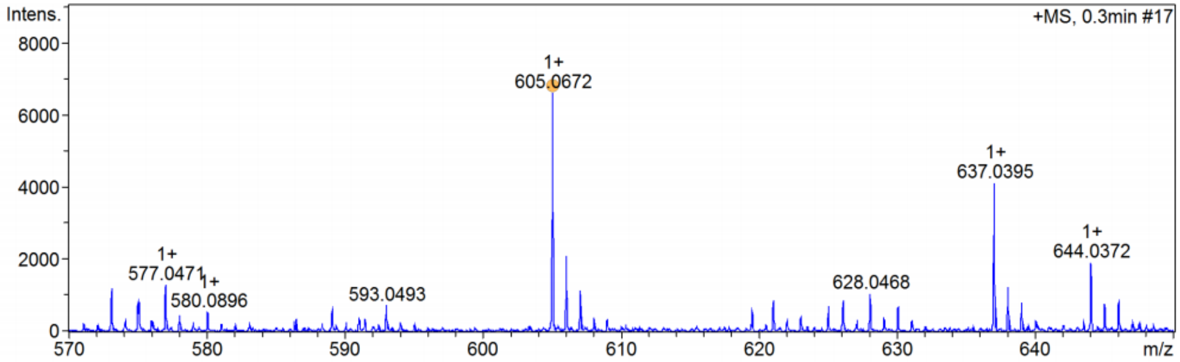

Meas. m/z	#	Ion Formula	m/z	err [ppm]	Mean err [ppm]	rdb	N-Rule	e¡¥ Conf	mSigma	Std I
605.067232	1	C27H22N2NaO9S2	605.065893	-2.2	909.4	17.5	ok	even	12.6	20.3

Figure S59. UV spectrum of compound 6

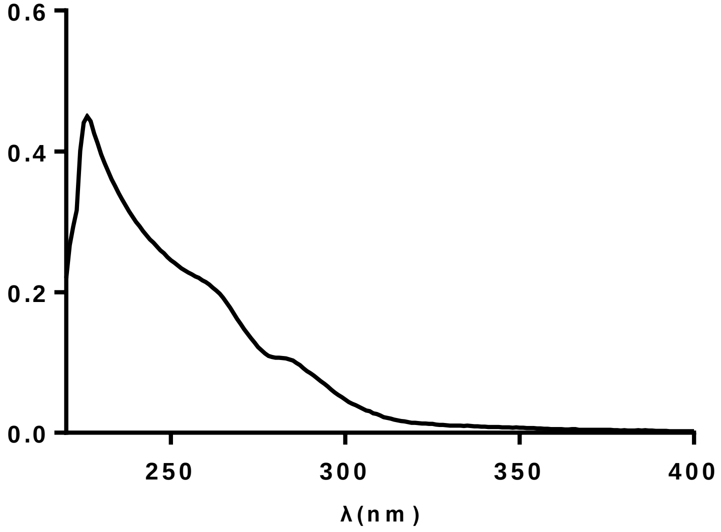

Figure S60. IR spectrum of compound 6

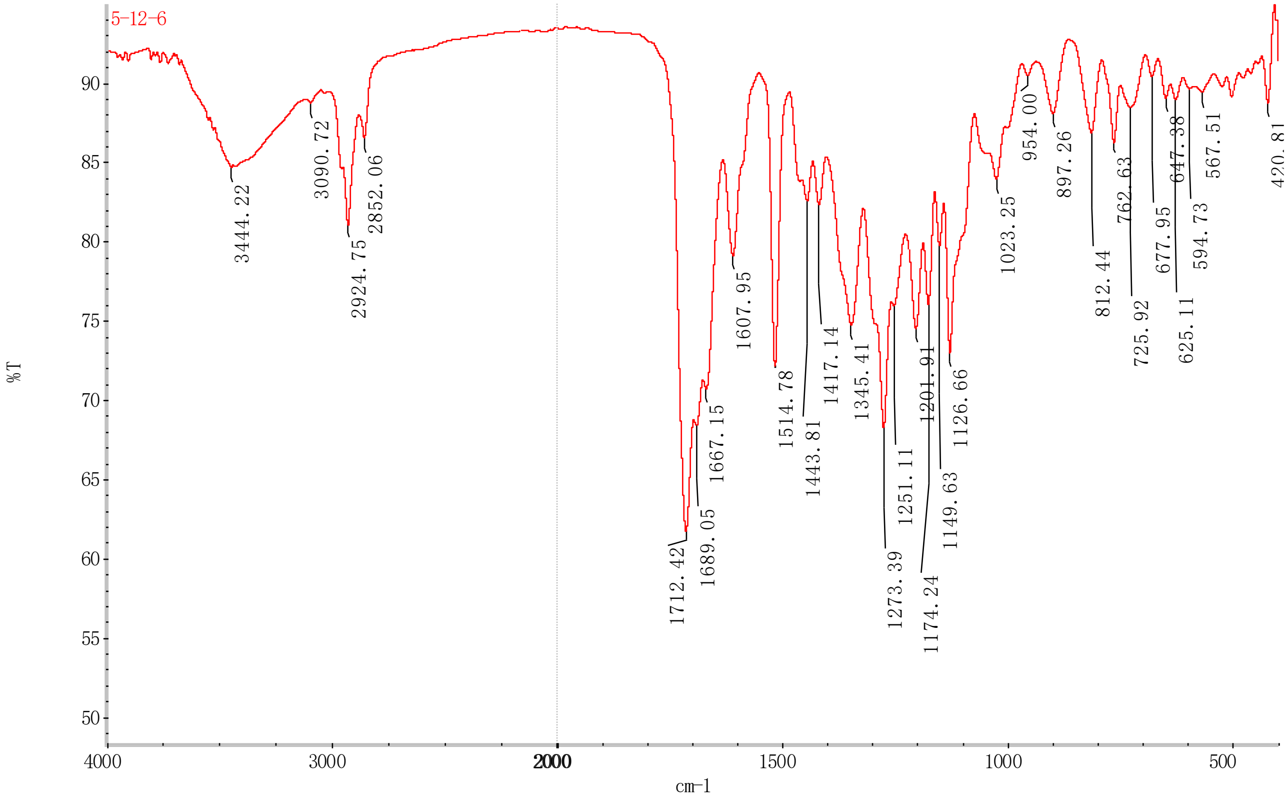

Figure S61. ^{1}H NMR spectrum of compound 10 (Recorded in $CDCl_3$)

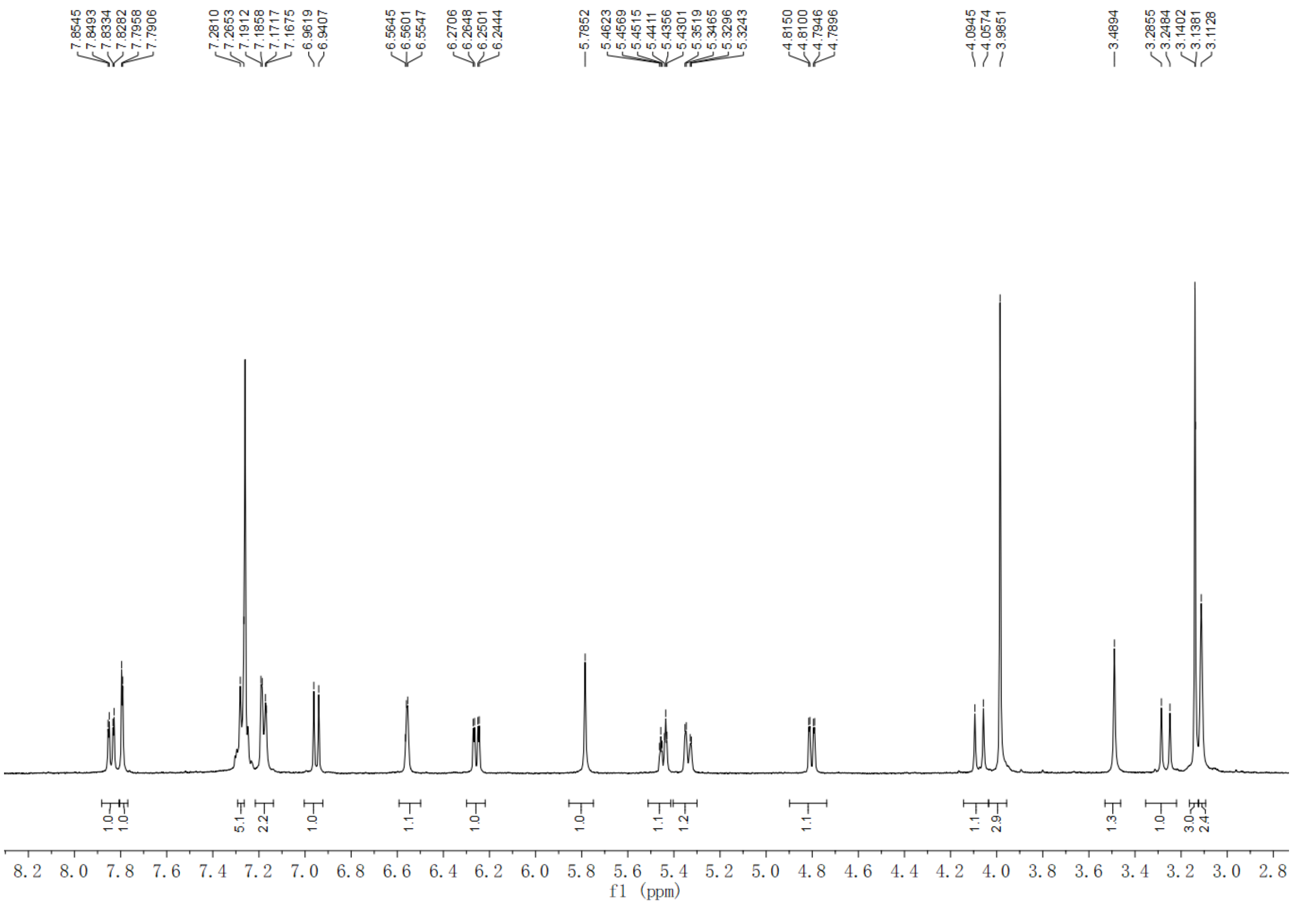

Figure S62. ^{13}C NMR and DEPT spectra of compound 10 (Recorded in $CDCl_3$)

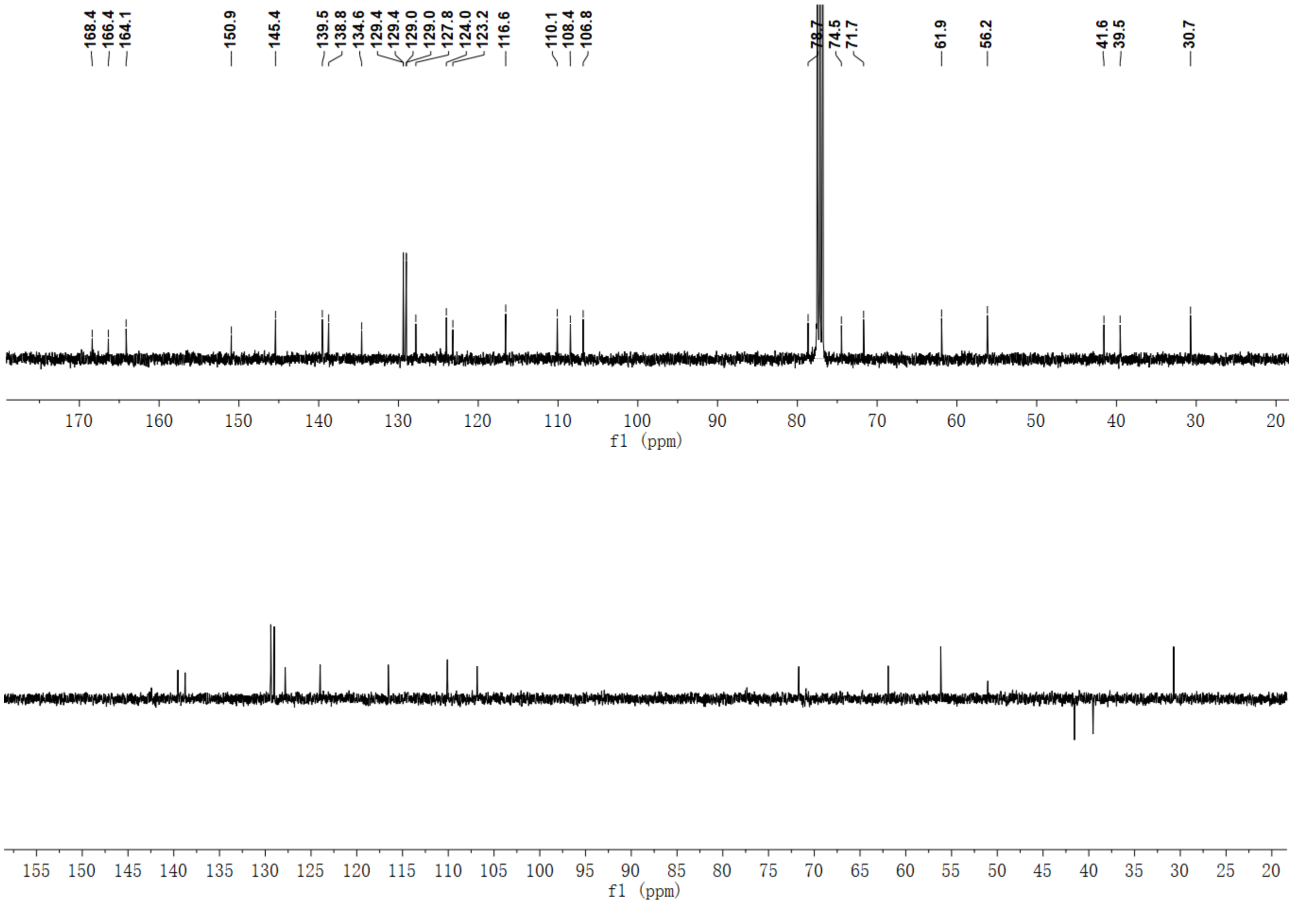

Figure S63. HSQC spectrum of compound 10 (Recorded in CDCl₃)

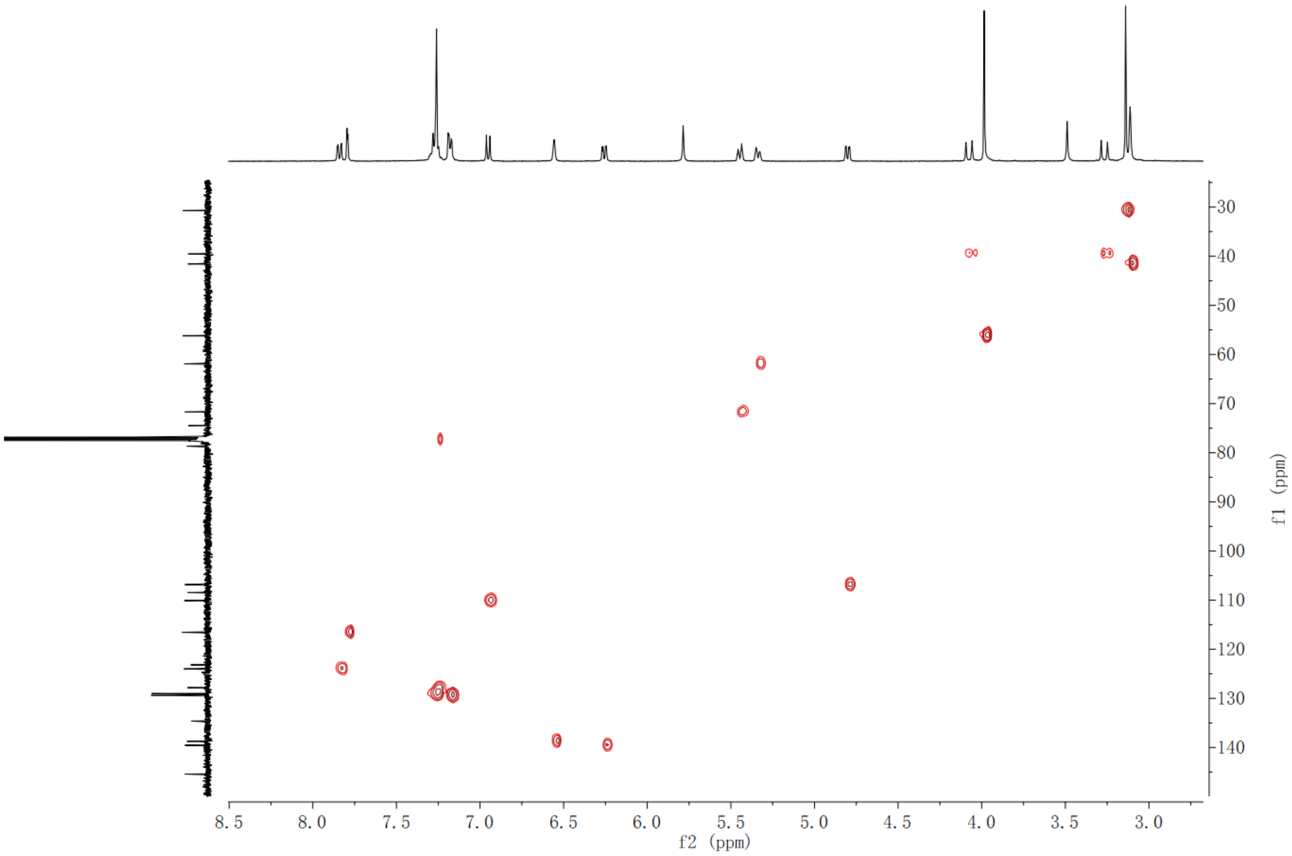

Figure S64. HMBC spectrum of compound 10 (Recorded in CDCl$_3$)

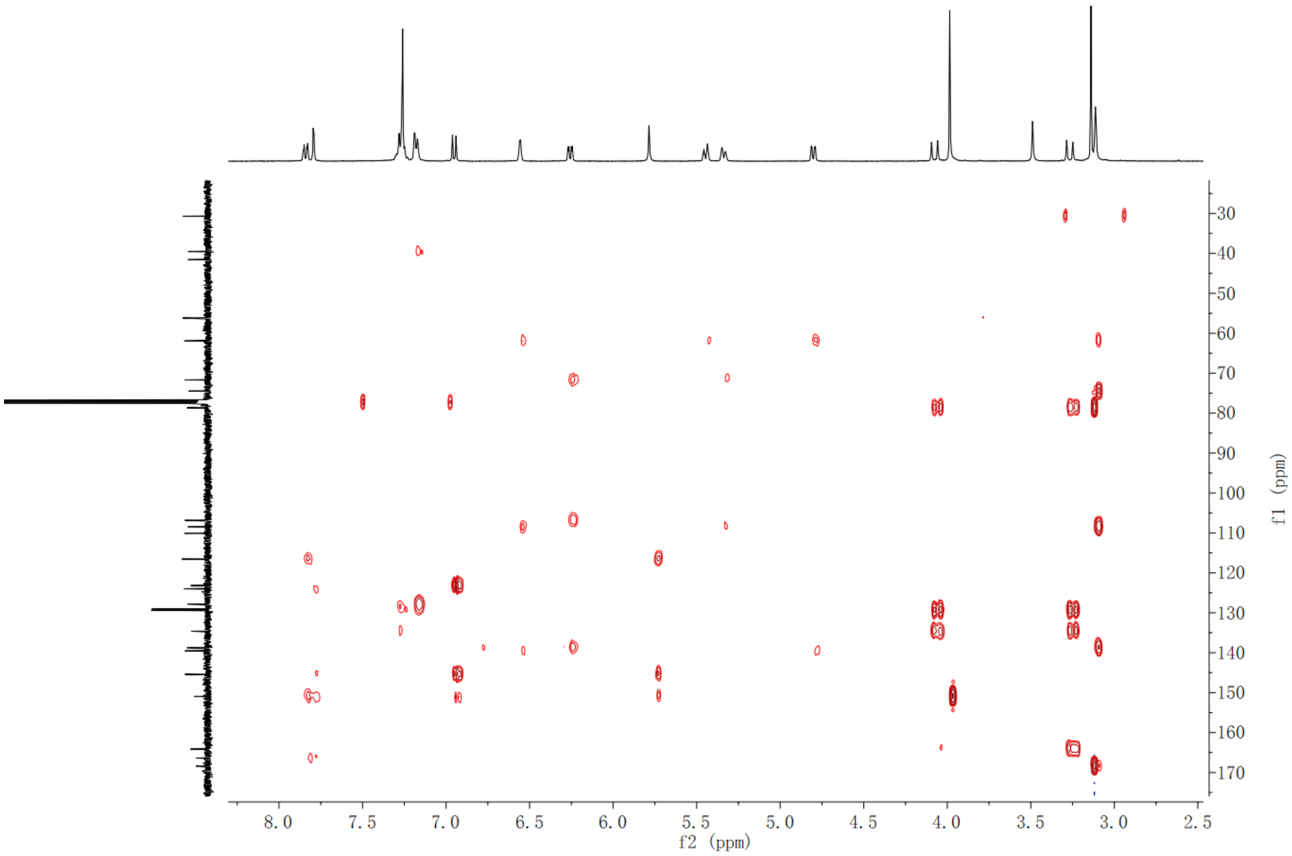

Figure S65. ^1H–^1H COSY spectrum of compound 10 (Recorded in $CDCl_3$)

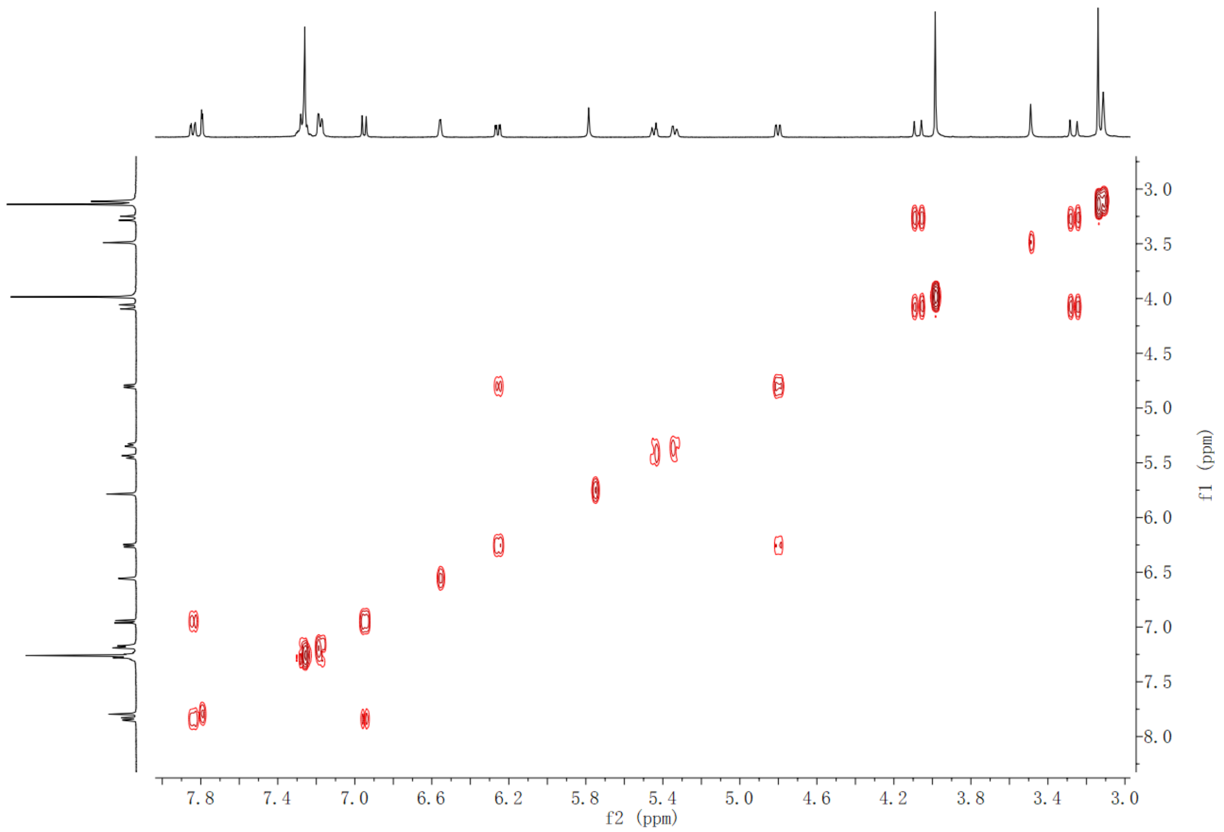

Figure S66. NOESY spectrum of compound 10 (Recorded in $CDCl_3$)

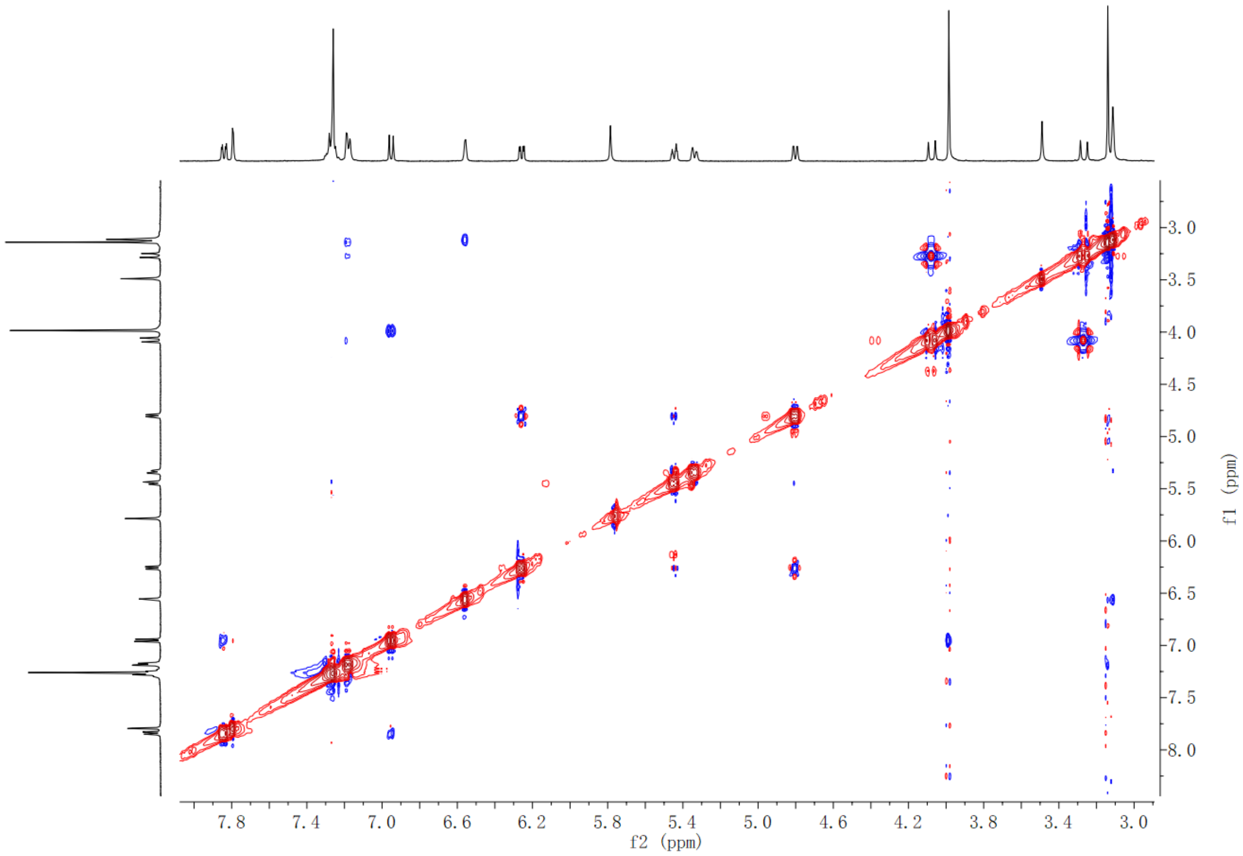

Figure S67. HRESIMS spectrum of compound 10

Mass Spectrum SmartFormula Report

Analysis Info		Acquisition D	2021/11/22 17:39:44
Analysis Name	H:\MS\5\LS5-6-7.d		
Method	Tune_pos_Standard.m	Operator	BDAL@DE
Sample Name		Instrument	micrOTOF II 8213750.10426
Comment			

Acquisition Paramet

Source Type	ESI	Ion Polarity	Positive	Set Nebulizer	0.3 Bar
Focus	Not active			Set Dry Heater	200 °C
Scan Begin	50 m/z	Set Capillary	4500 V	Set Dry Gas	4.0 l/min
Scan End	3150 m/z	Set End Plate Offset	-500 V	Set Divert Valve	Waste

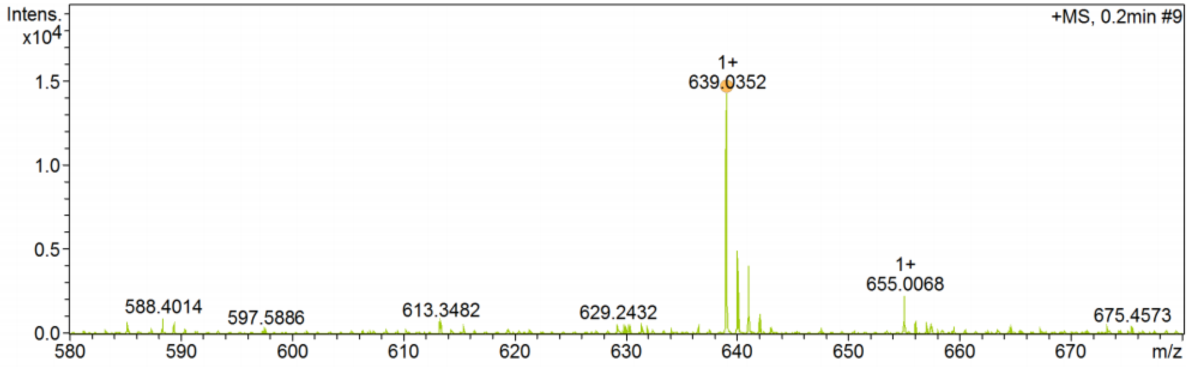

Meas. m/z	#	Ion Formula	m/z	err [ppm]	Mean err [ppm]	rdb	N-Rule	e¡¥ Conf	mSigma	Std I	Std Mean m/z
639.035178	1	C27H24N2NaO7S4	639.035856	1.1	3.8	16.5	ok	even	17.6	26.7	n.a.

Figure S68. UV spectrum of compound 10

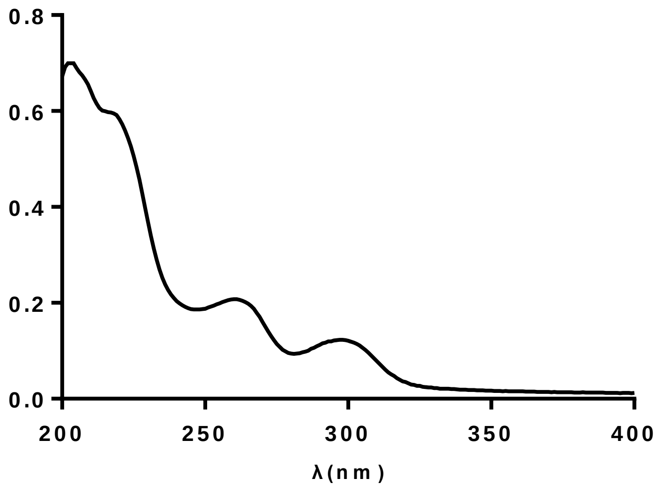

Figure S69. IR spectrum of compound 10

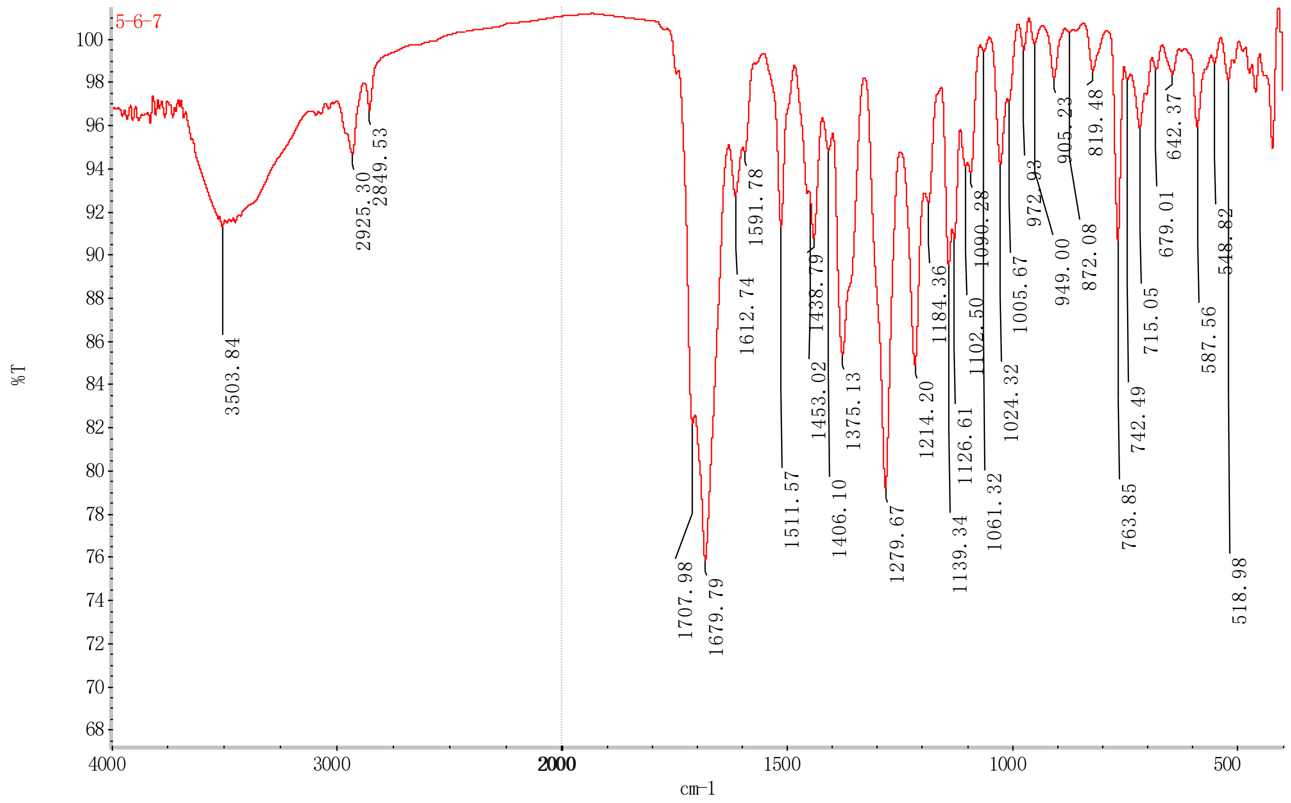

Figure S70. ^{1}H NMR spectrum of compound 11 (Recorded in CD_3OD)

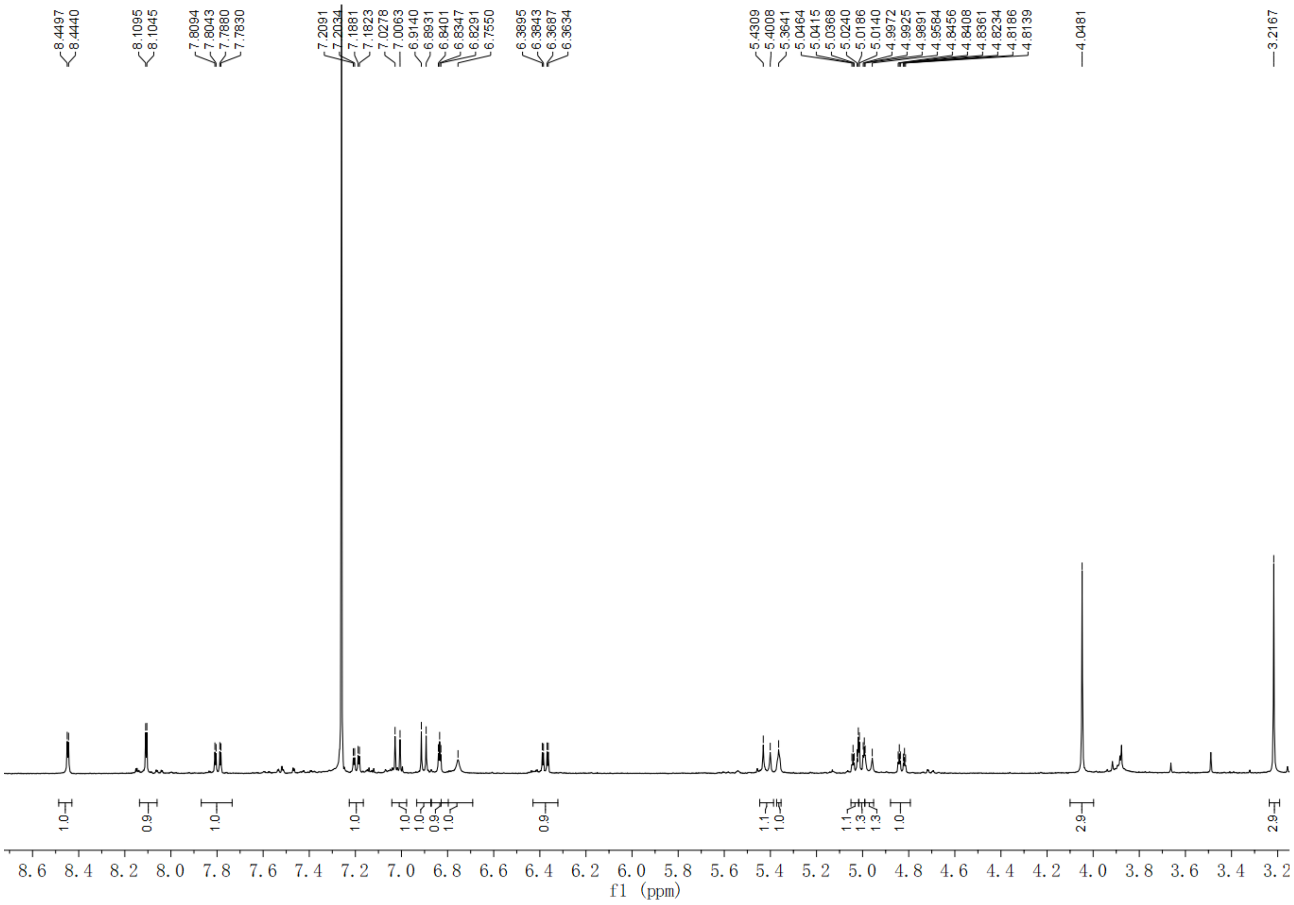

Figure S71. ^{13}C NMR and DEPT spectra of compound 11 (Recorded in CD_3OD)

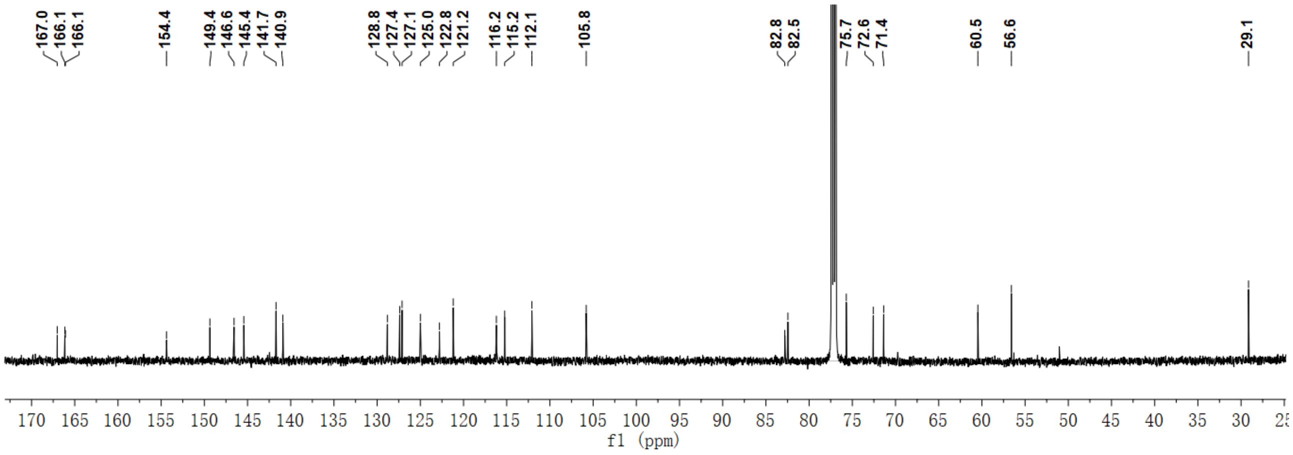

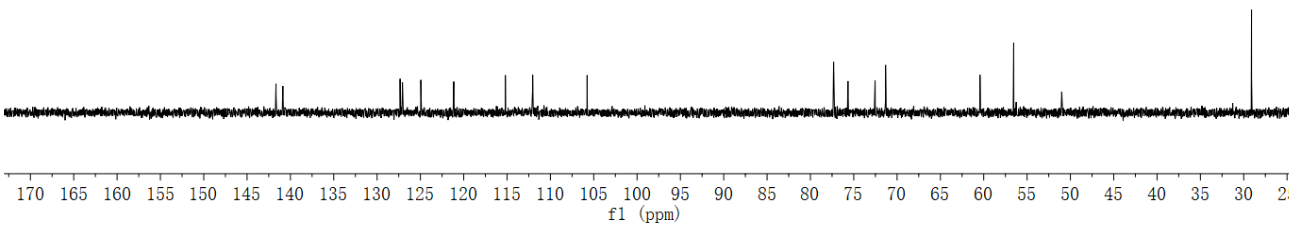

Figure S72. HSQC spectrum of compound 11 (Recorded in CD_3OD)

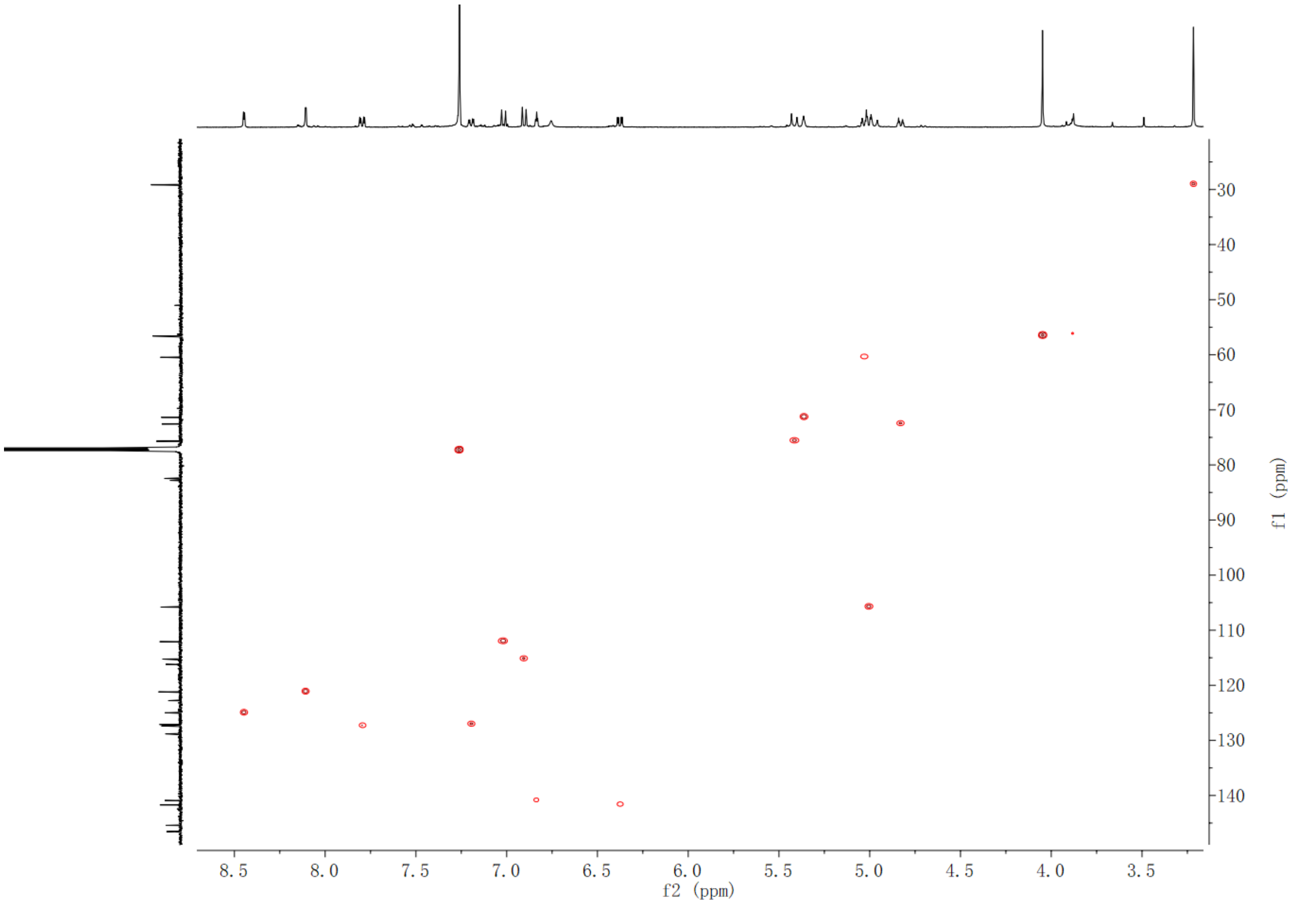

Figure S73. HMBC spectrum of compound 11 (Recorded in CD_3OD)

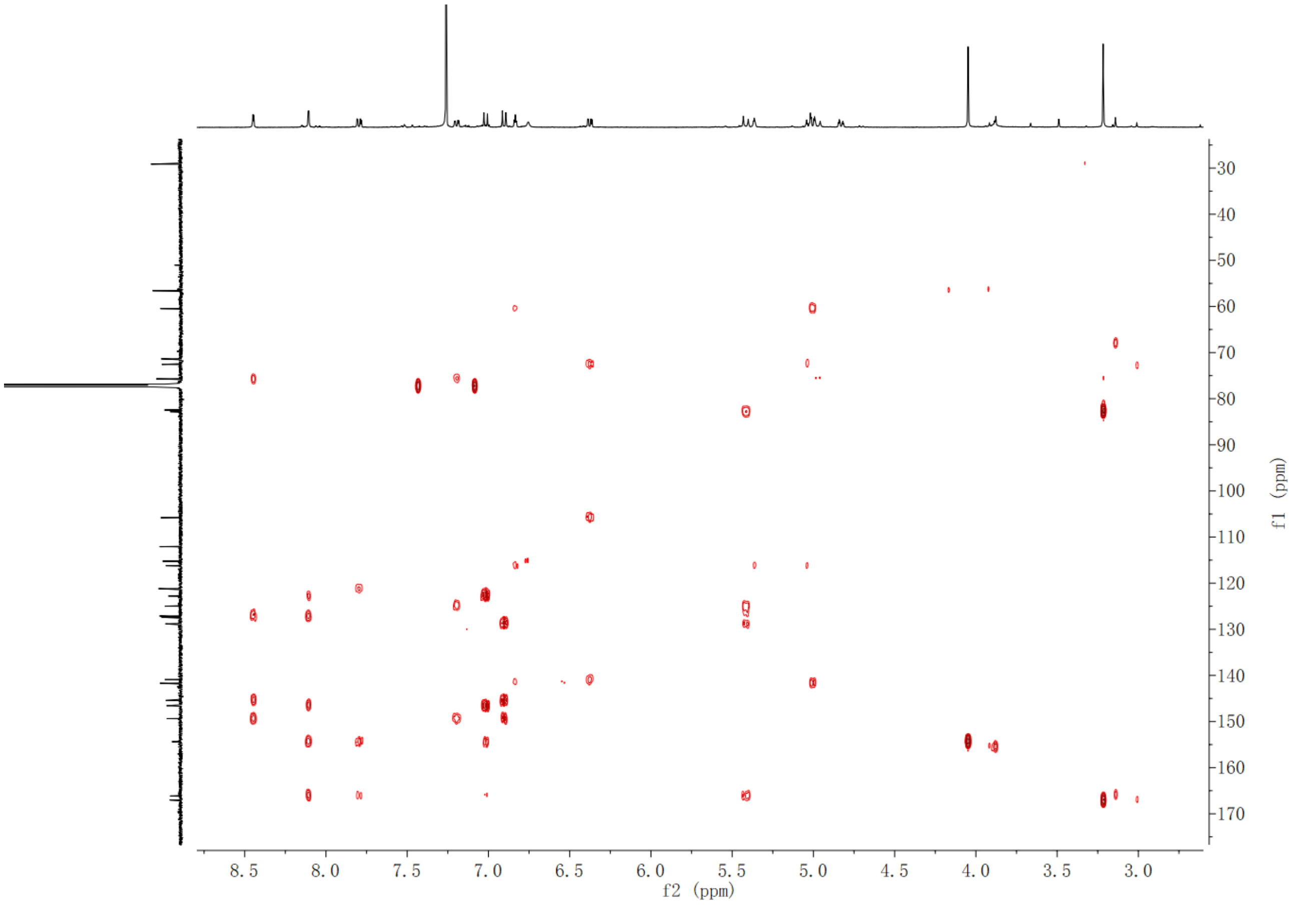

Figure S74. ^1H–^1H **COSY spectrum of compound 11 (Recorded in** CD_3OD**)**

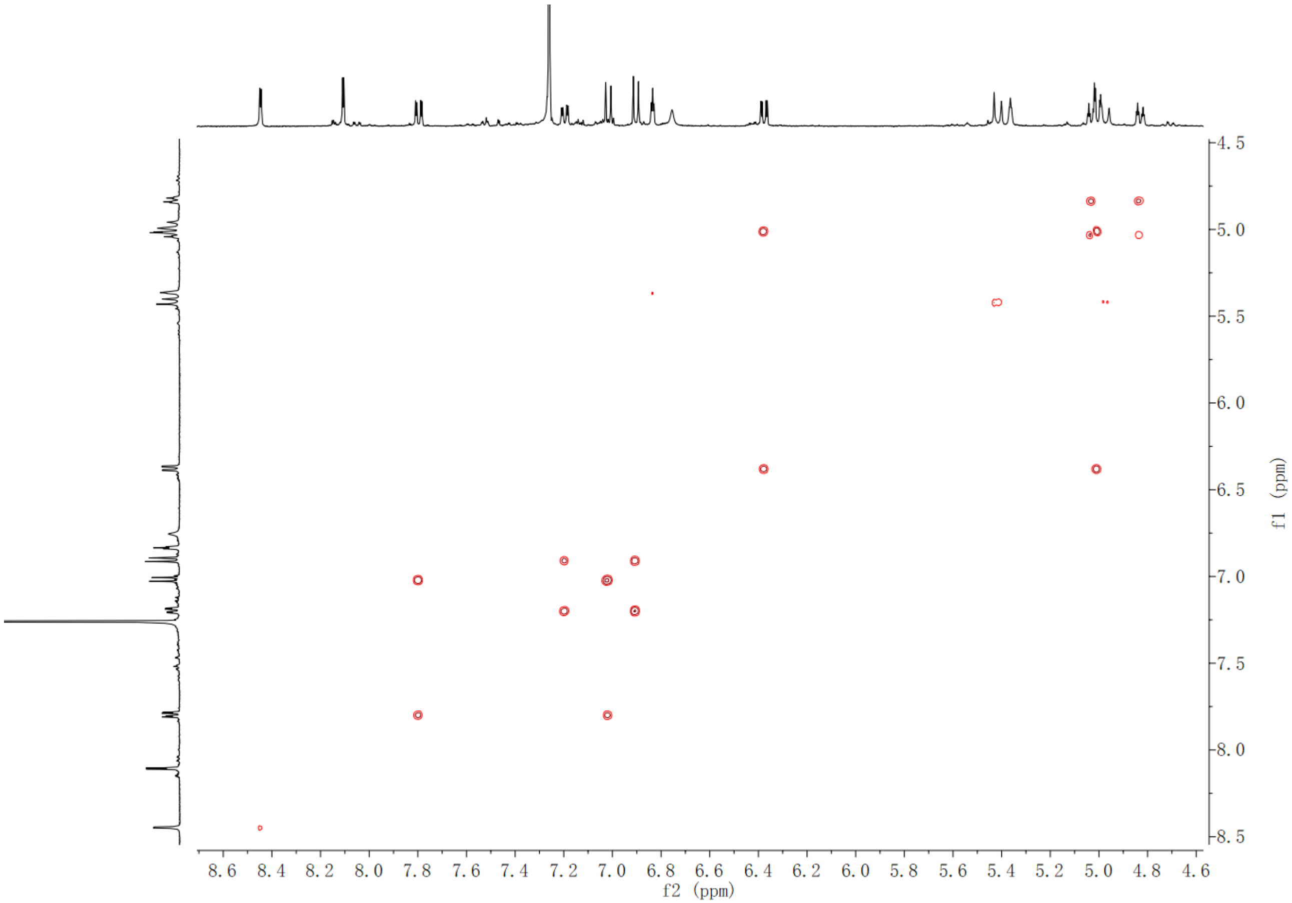

Figure S75. NOESY spectrum of compound 11 (Recorded in CD_3OD)

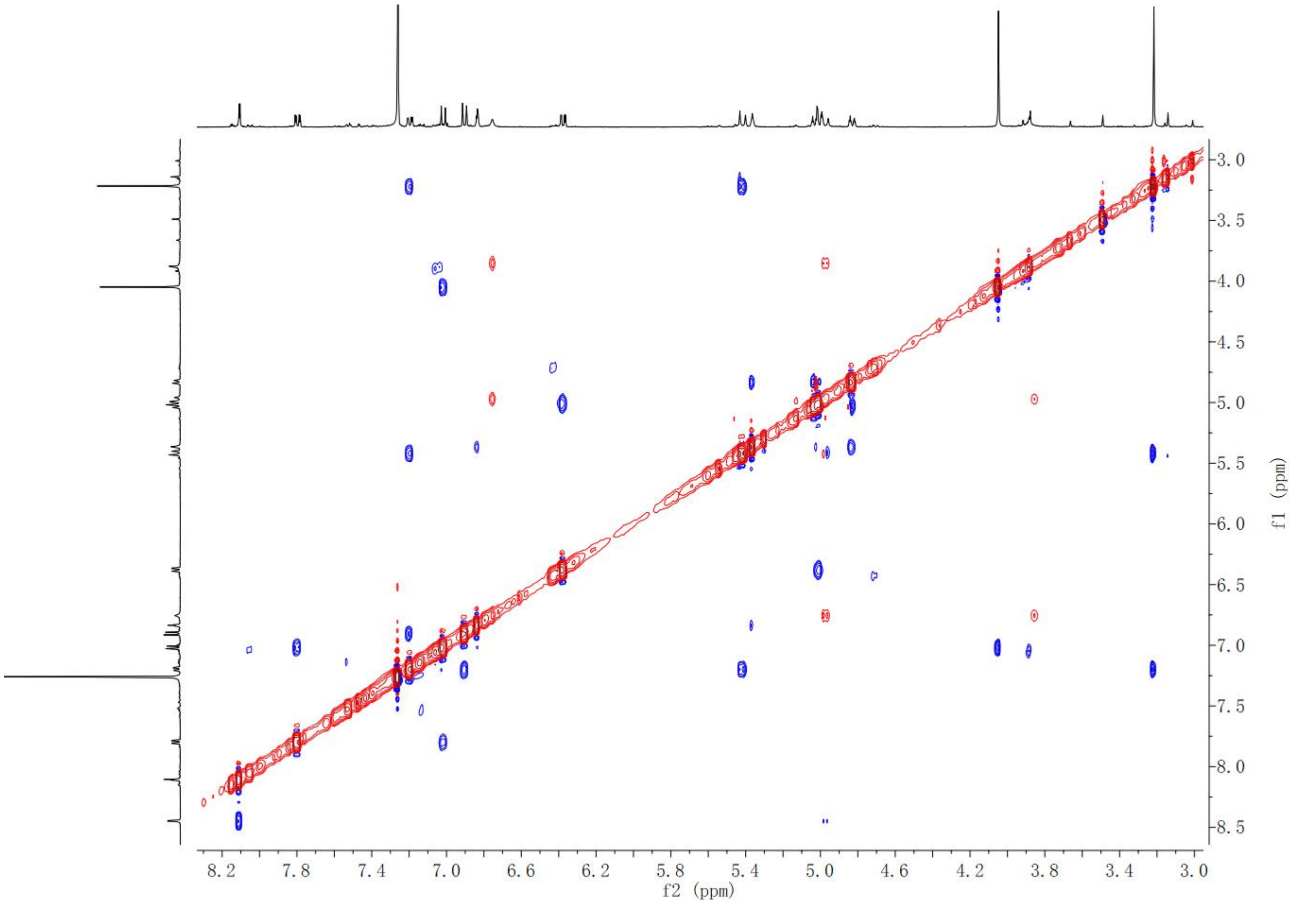

Figure S76. HRESIMS spectrum of compound 11

Mass Spectrum SmartFormula Report

Analysis Info		Acquisition D	2021/9/2 17:32:52
Analysis Name	H:\MS\11. LS5-8-8-9.d		
Method	Tune_pos_Mid.m	Operator	BDAL@DE
Sample Name		Instrument	micrOTOF II 8213750.10426
Comment			

Acquisition Paramet

Source Type	ESI	Ion Polarity	Positive	Set Nebulizer	0.3 Bar
Focus	Not active			Set Dry Heater	200 °C
Scan Begin	50 m/z	Set Capillary	4500 V	Set Dry Gas	4.0 l/min
Scan End	3300 m/z	Set End Plate Offset	-500 V	Set Divert Valve	Waste

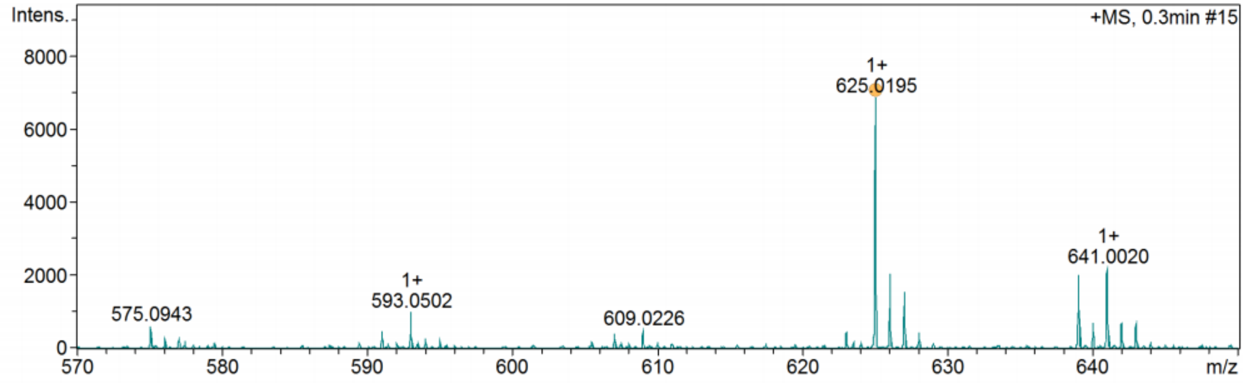

Meas. m/z	#	Ion Formula	m/z	err [ppm]	Mean err [ppm]	rdb	N-Rule	e;¥ Conf	mSigma	Std I
625.019481	1	C26H22N2NaO7S4	625.020206	1.2	877.8	16.5	ok	even	18.3	33.0

Figure S77. UV spectrum of compound 11

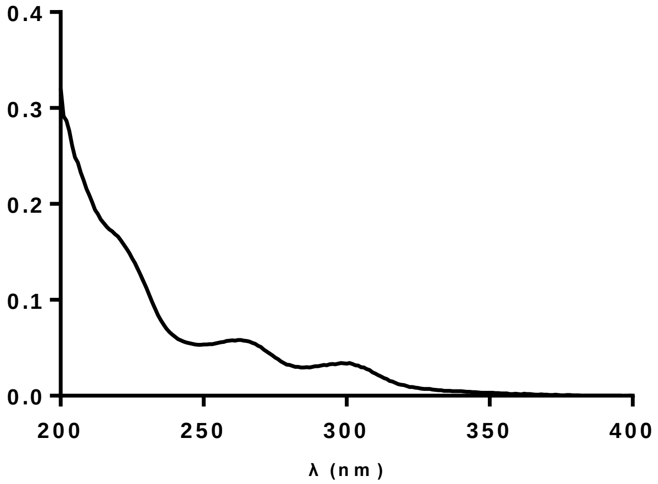

Figure S78. IR spectrum of compound 11

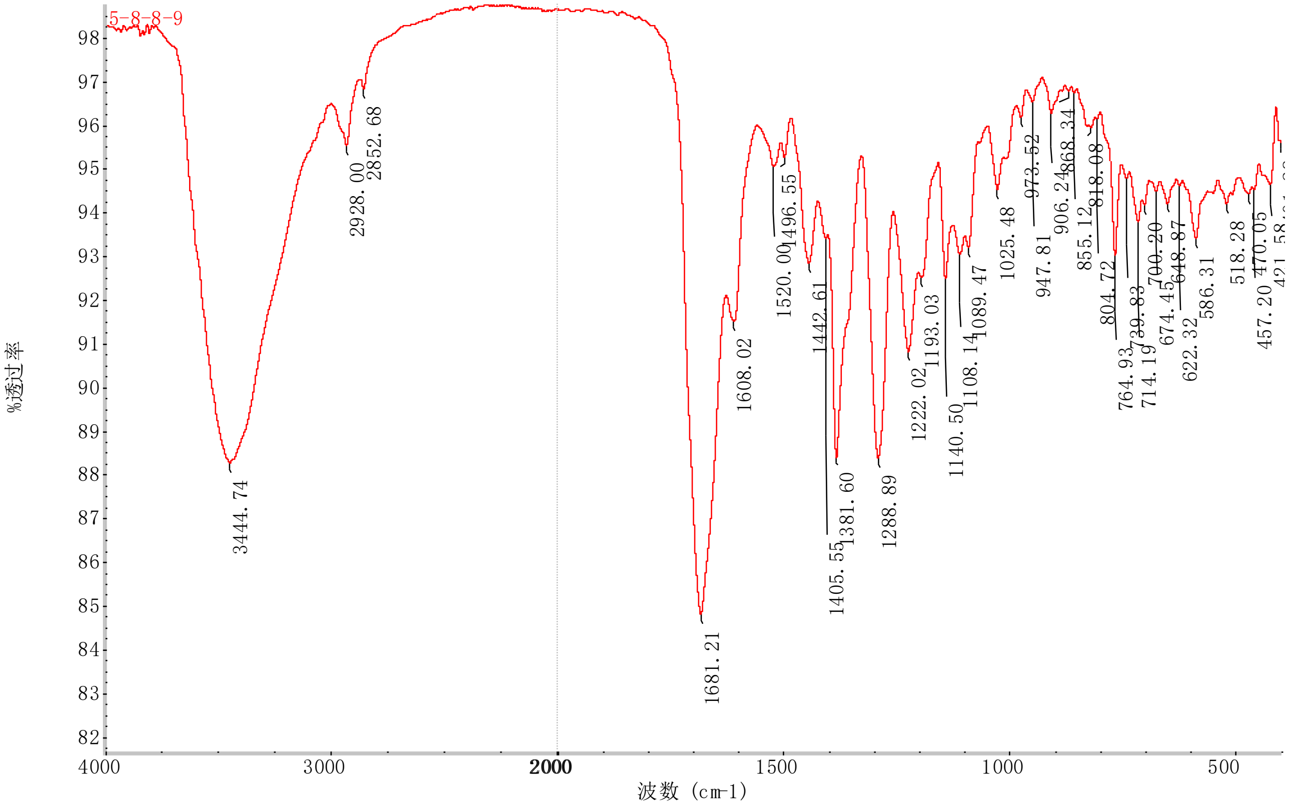

Figure S79. ^{1}H NMR spectrum of compound 12 (Recorded in $CDCl_3$)

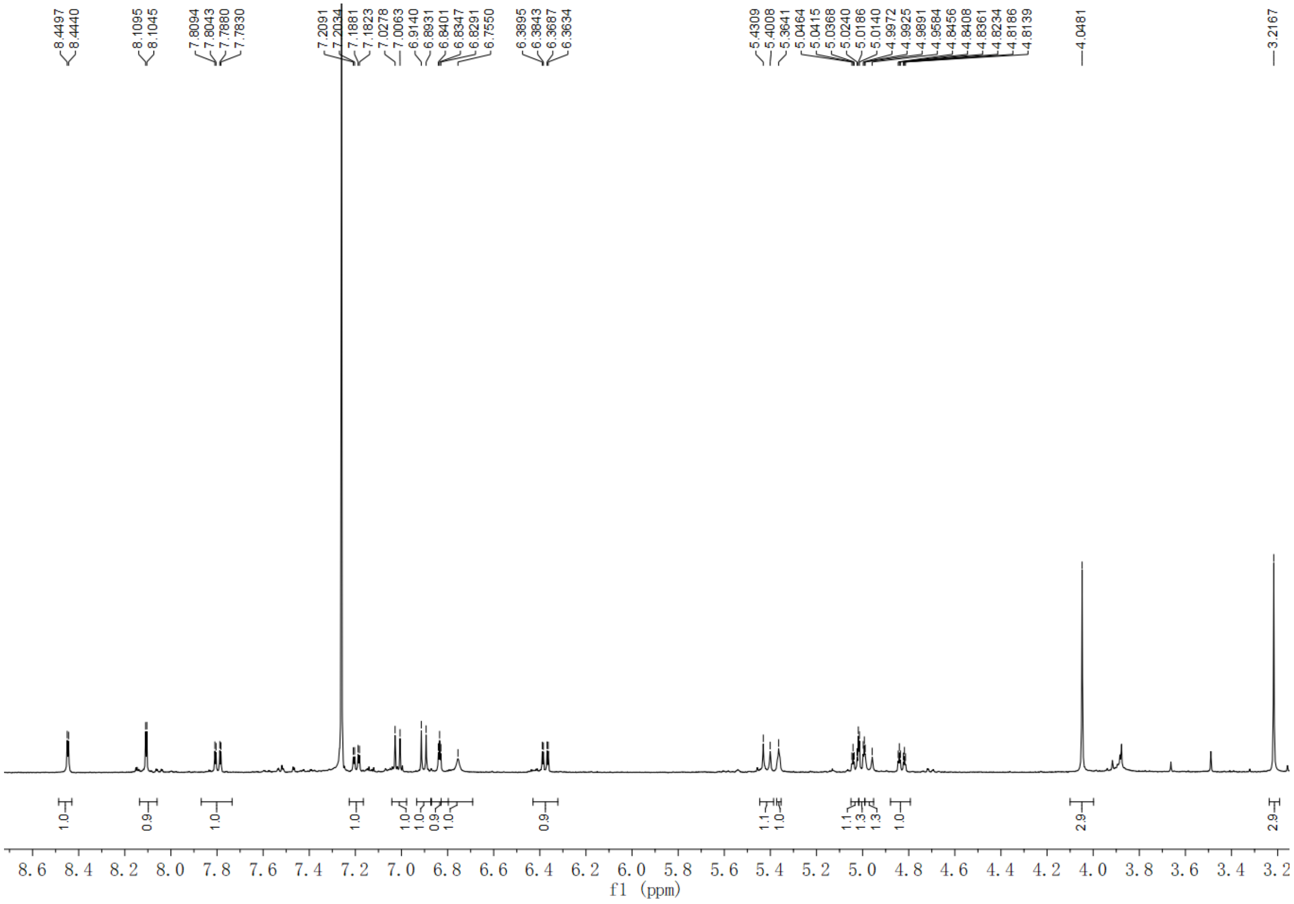

Figure S80. ^{13}C NMR and DEPT spectra of compound 12 (Recorded in $CDCl_3$)

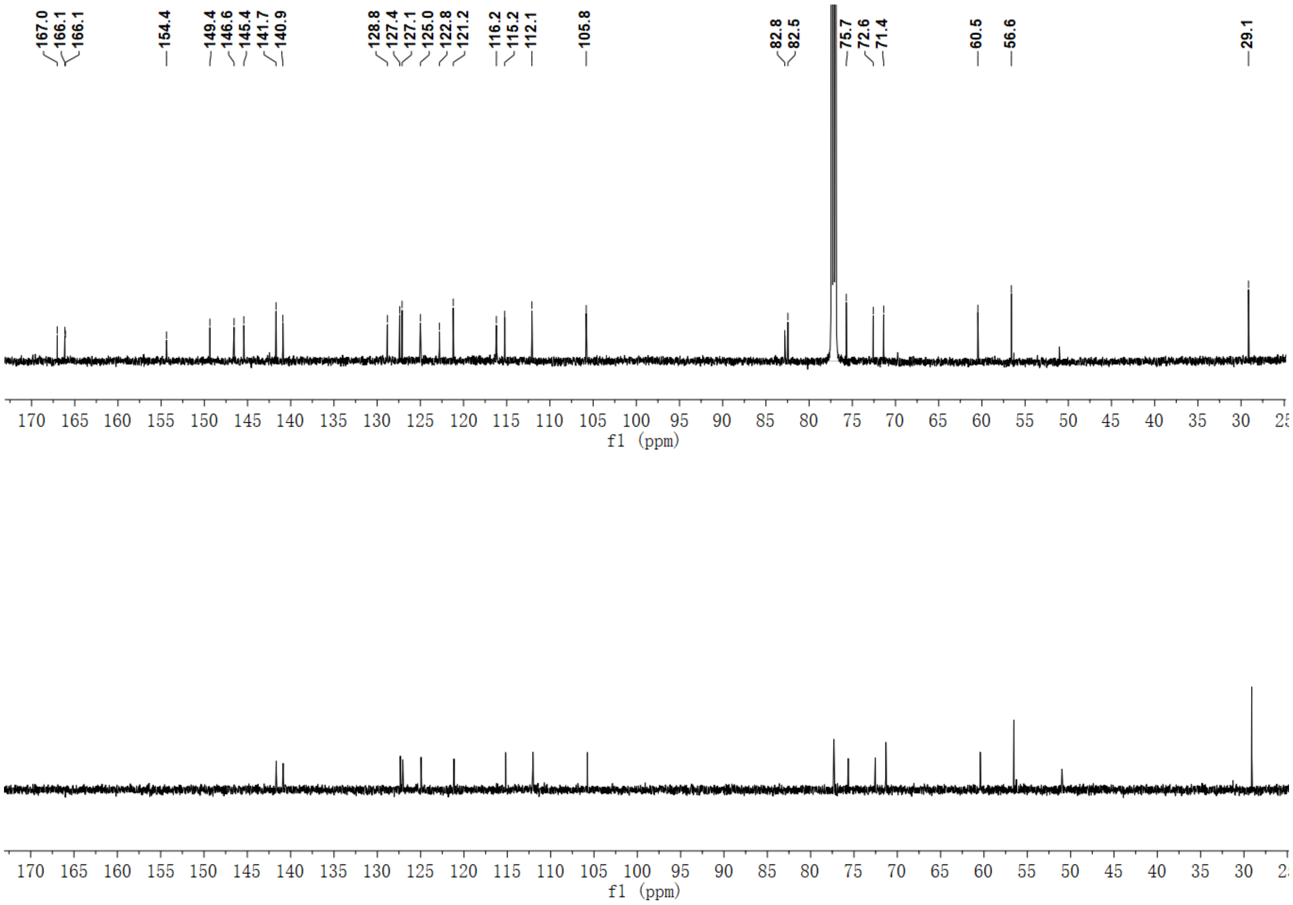

Figure S81. HSQC spectrum of compound 12 (Recorded in CDCl3)

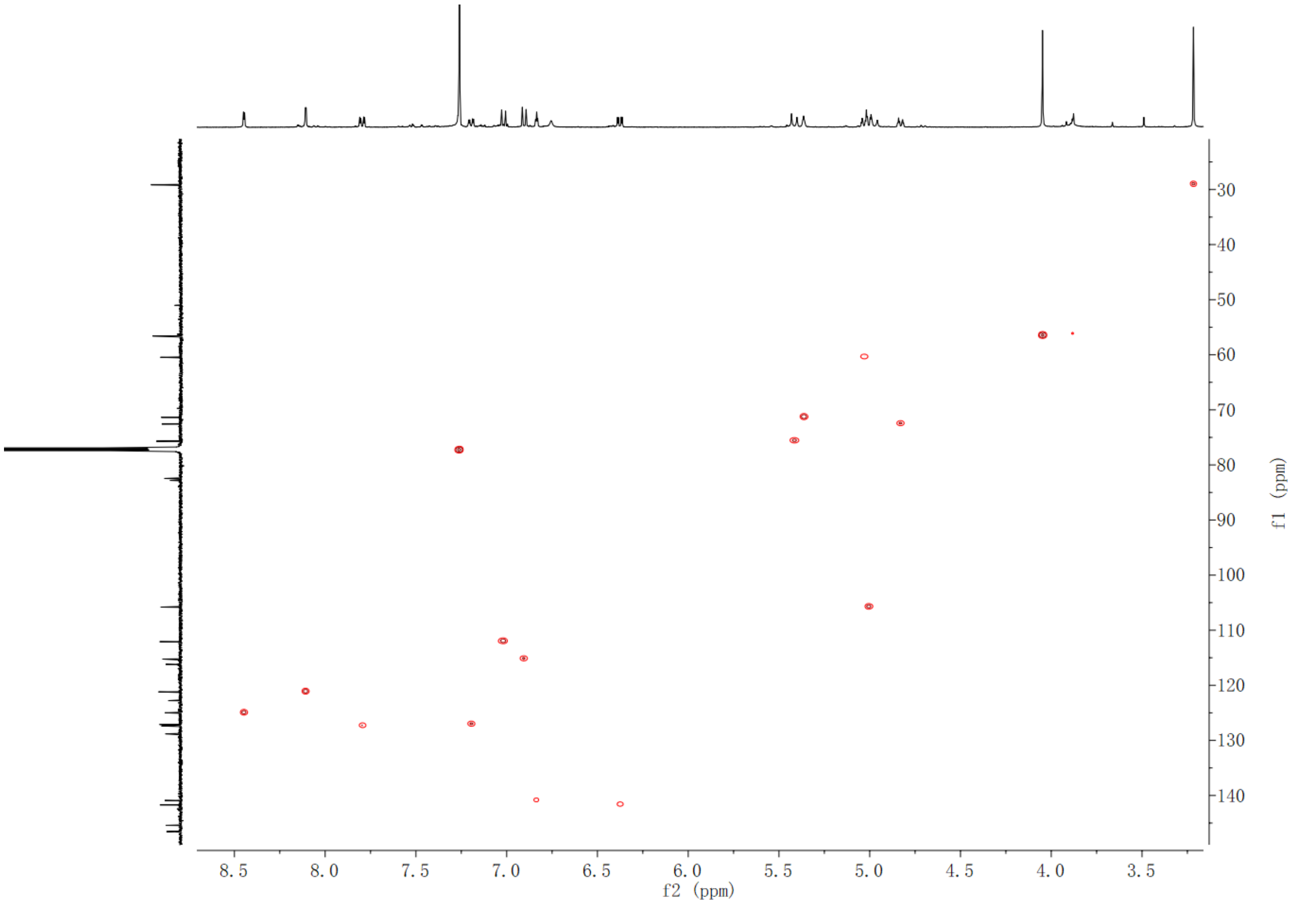

Figure S82. HMBC spectrum of compound 12 (Recorded in $CDCl_3$)

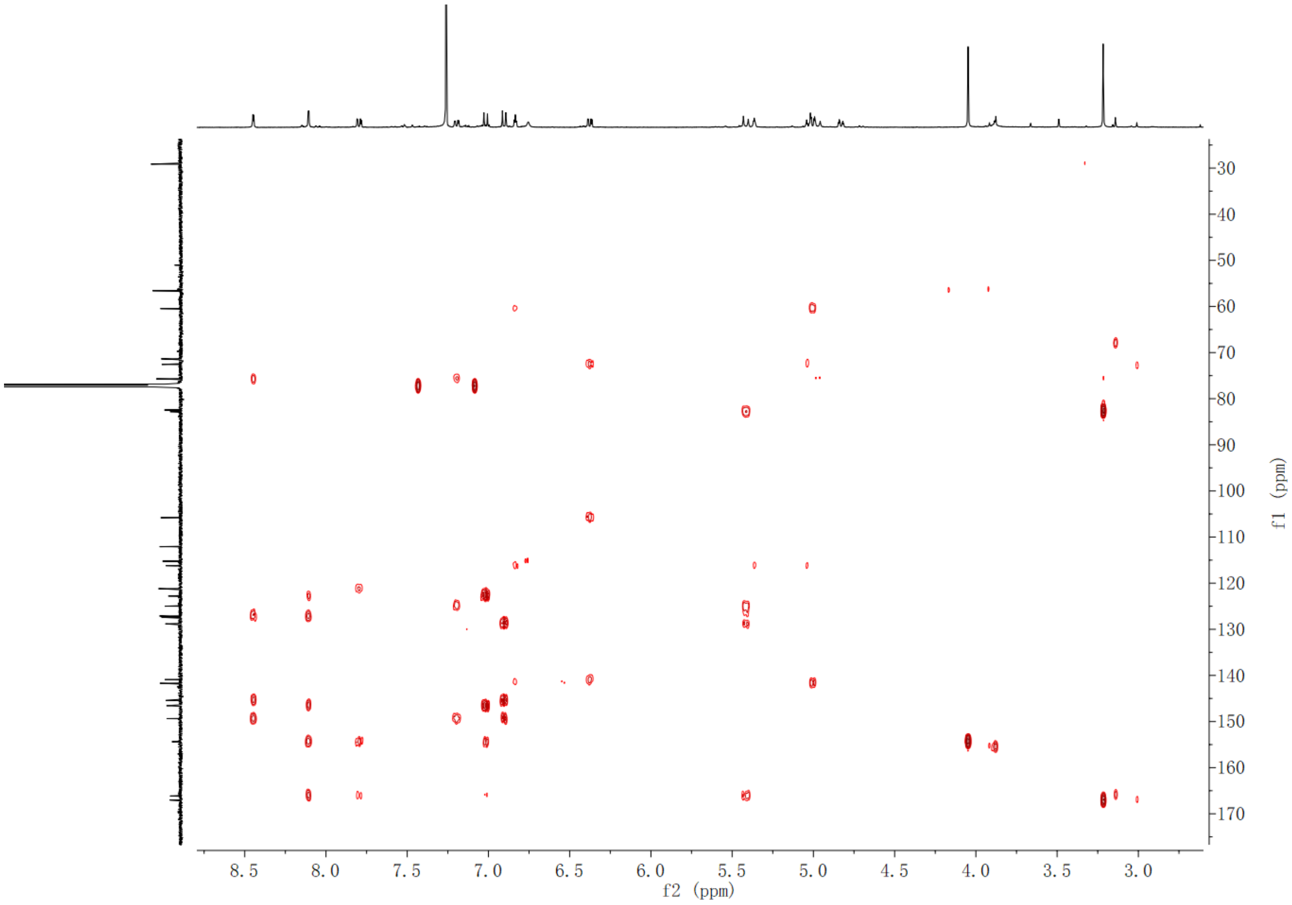

Figure S83. ^{1}H–^{1}H COSY spectrum of compound 12 (Recorded in CDCl$_3$)

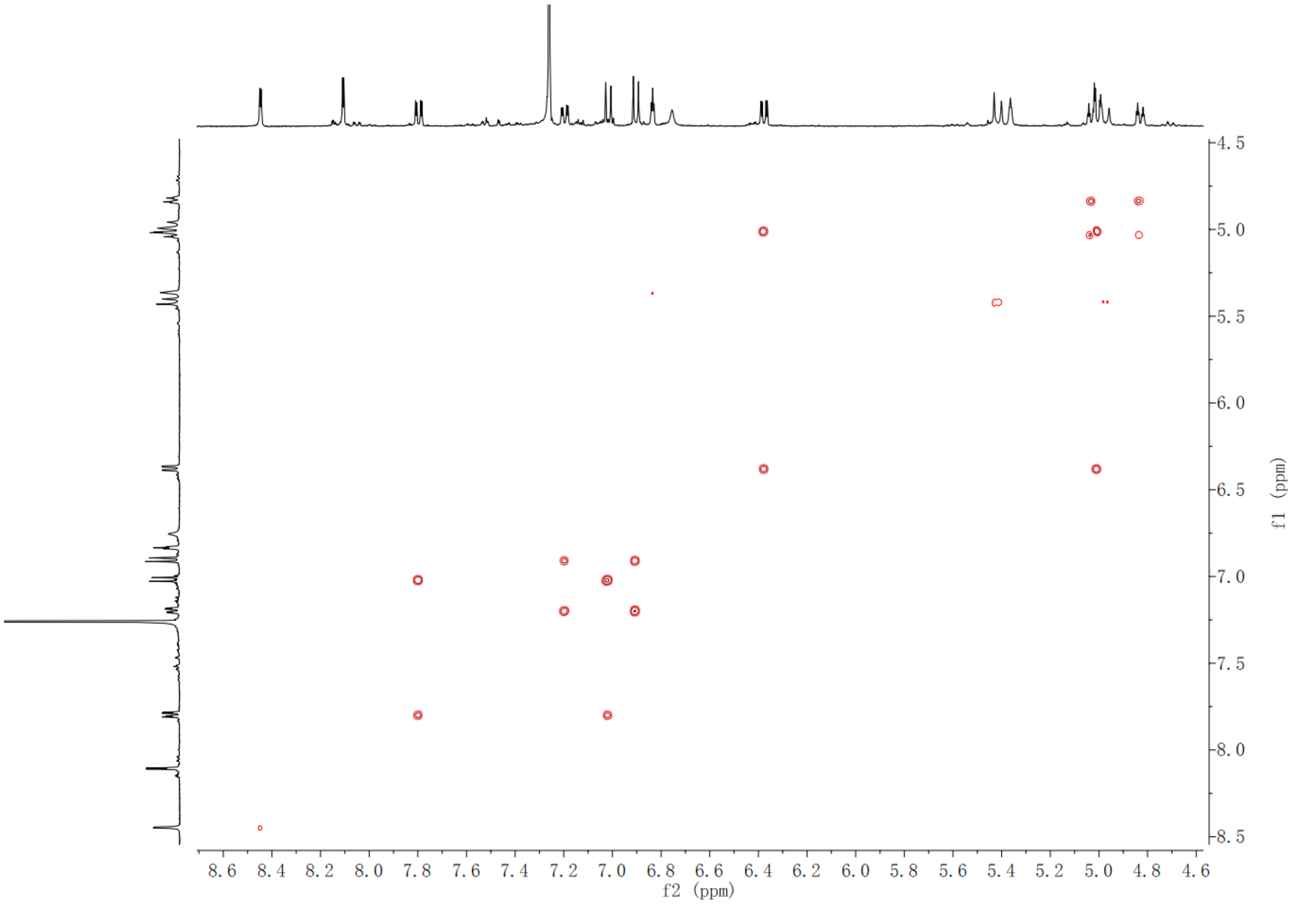

Figure S84. NOESY spectrum of compound 12 (Recorded in $CDCl_3$)

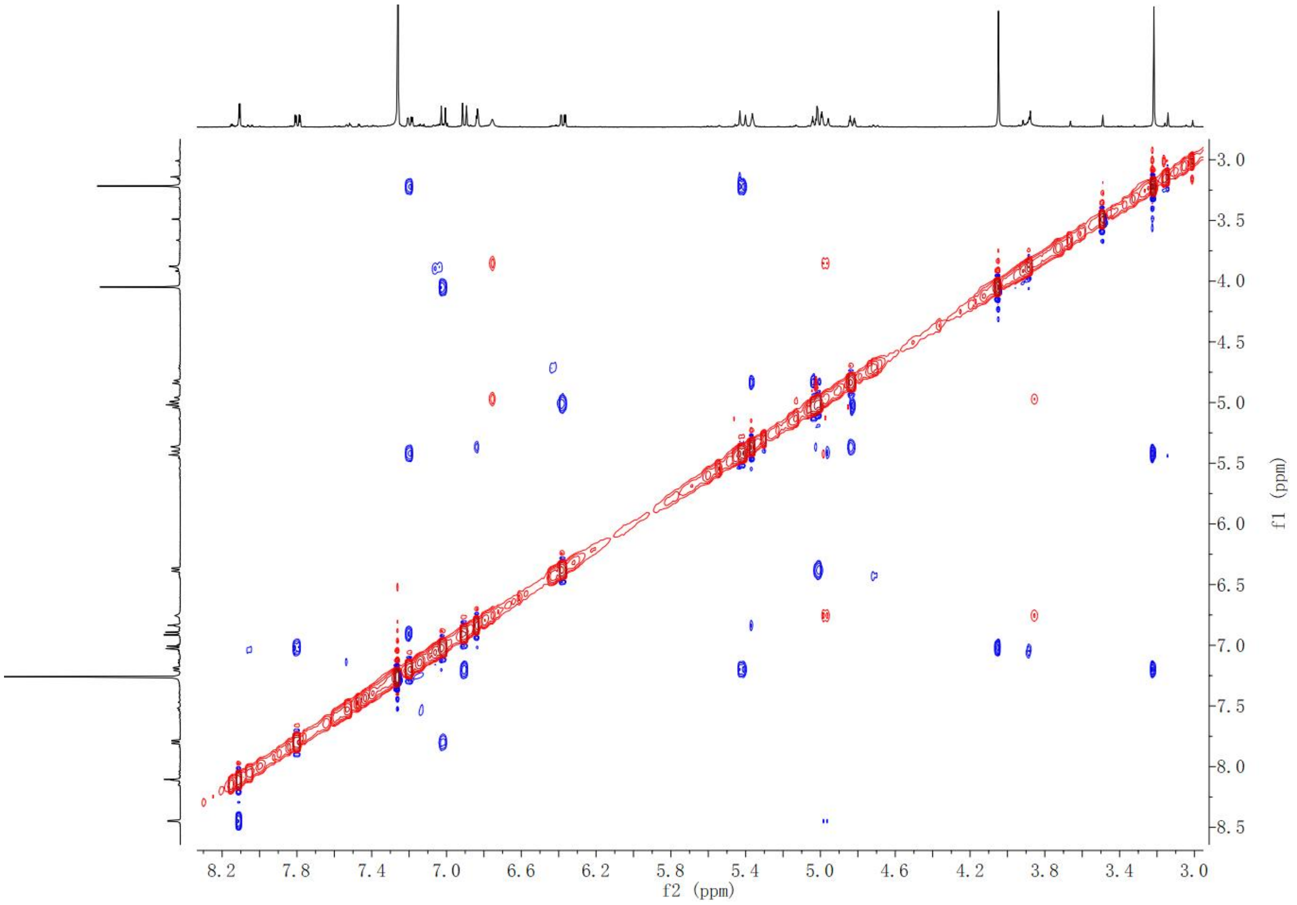

Figure S85. HRESIMS spectrum of compound 12

Mass Spectrum SmartFormula Report

Analysis Info		Acquisition D	2021/10/26 17:43:54
Analysis Name	H:\MS\5\LS5-16-1-1026.d		
Method	Tune_pos_Mid.m	Operator	BDAL@DE
Sample Name		Instrument	micrOTOF II 8213750.1042 6
Comment			

Acquisition Paramet					
Source Type	ESI	Ion Polarity	Positive	Set Nebulizer	0.3 Bar
Focus	Not active			Set Dry Heater	200 °C
Scan Begin	50 m/z	Set Capillary	4500 V	Set Dry Gas	4.0 l/min
Scan End	3300 m/z	Set End Plate Offset	-500 V	Set Divert Valve	Waste

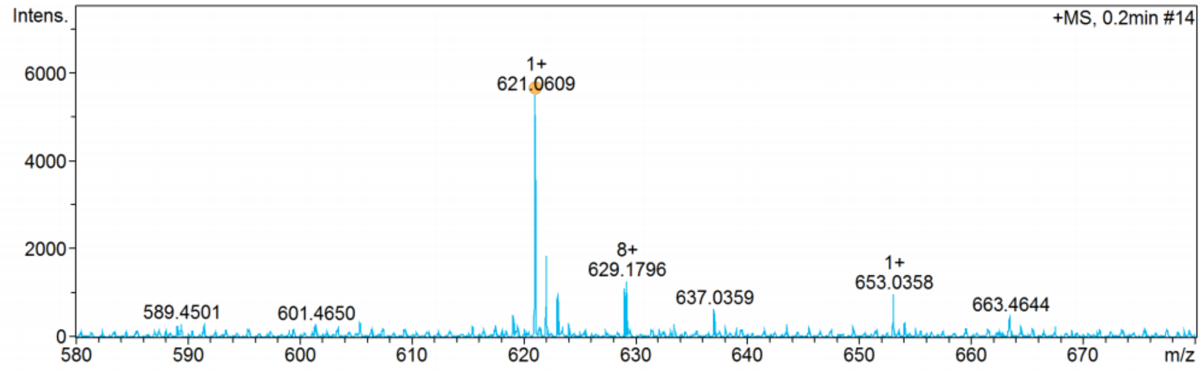

Meas. m/z	#	Ion Formula	m/z	err [ppm]	Mean err [ppm]	rdb	N-Rule	e¡¥ Conf	mSigma	Std I
621.060871	1	C27H22N2NaO10S2	621.060808	-0.1	886.6	17.5	ok	even	13.4	22.2

Figure S86. UV spectrum of compound 12

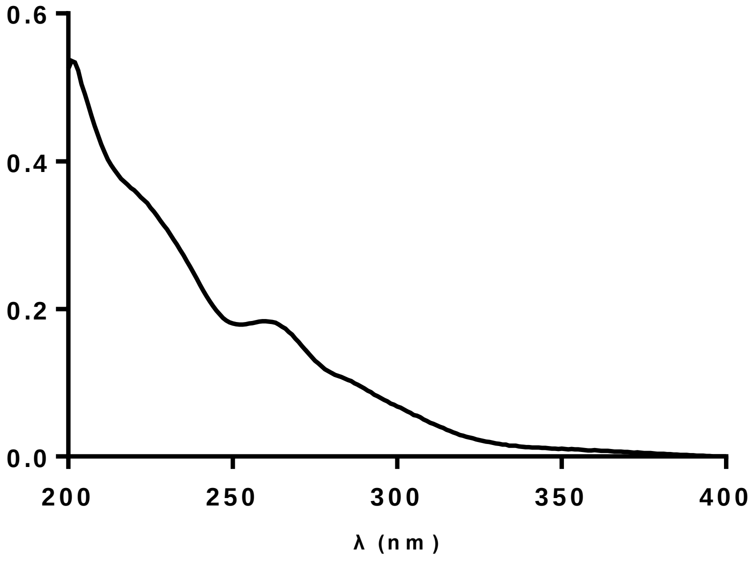

Figure S87. IR spectrum of compound 12

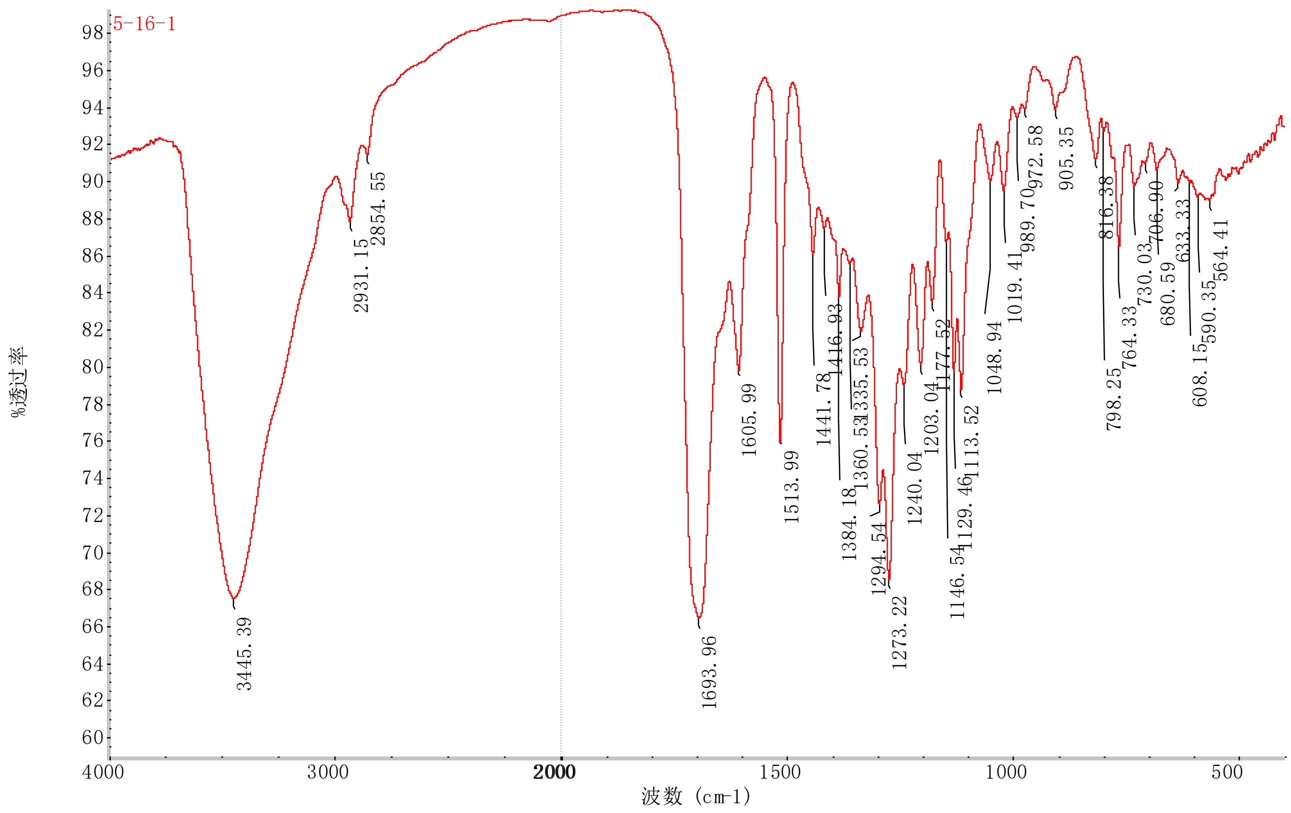

Figure S88. ^{1}H NMR spectrum of compound 13 (Recorded in CD_3OD)

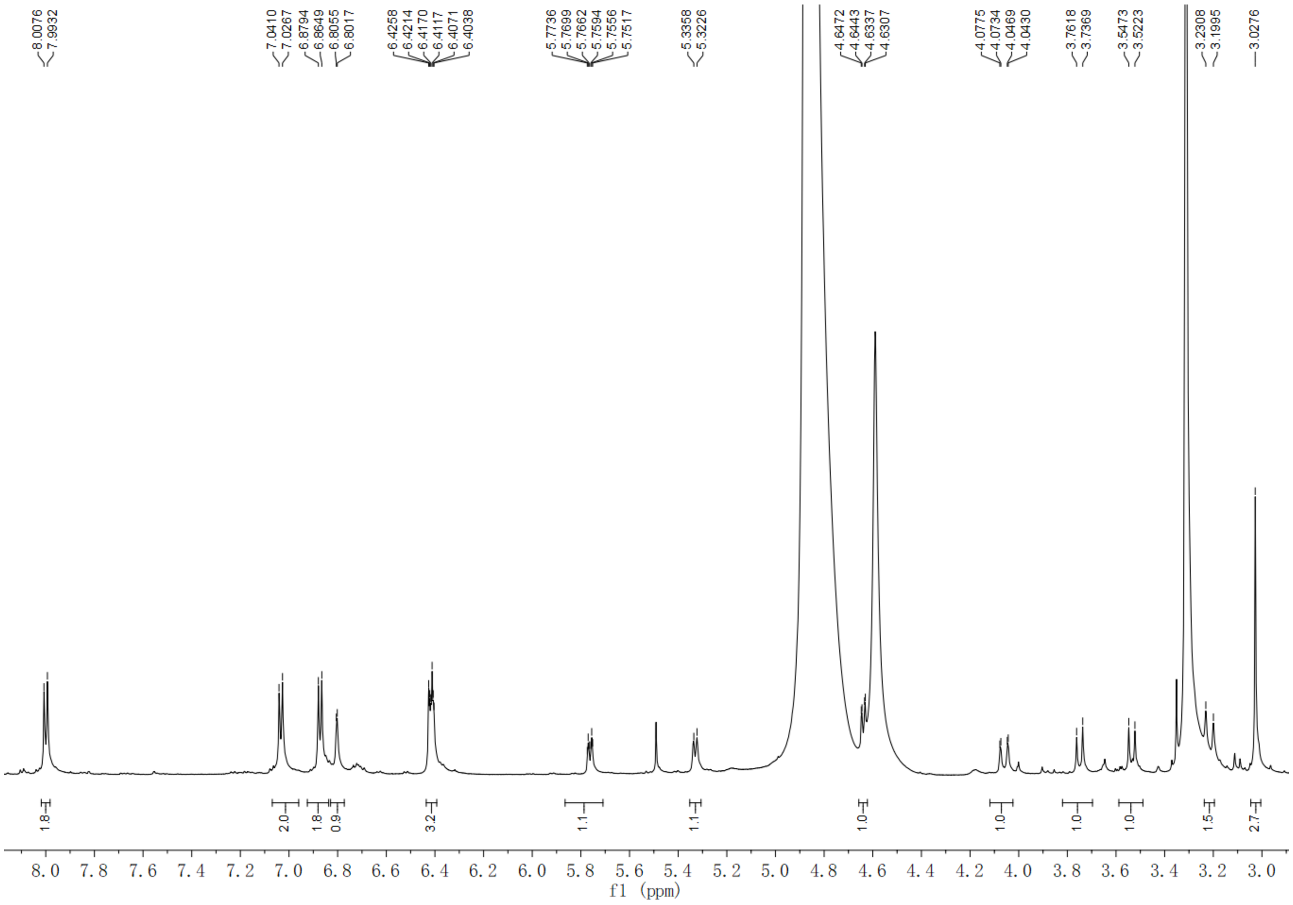

Figure S89. ^{13}C **NMR and DEPT spectra of compound 13 (Recorded in** CD_3OD**)**

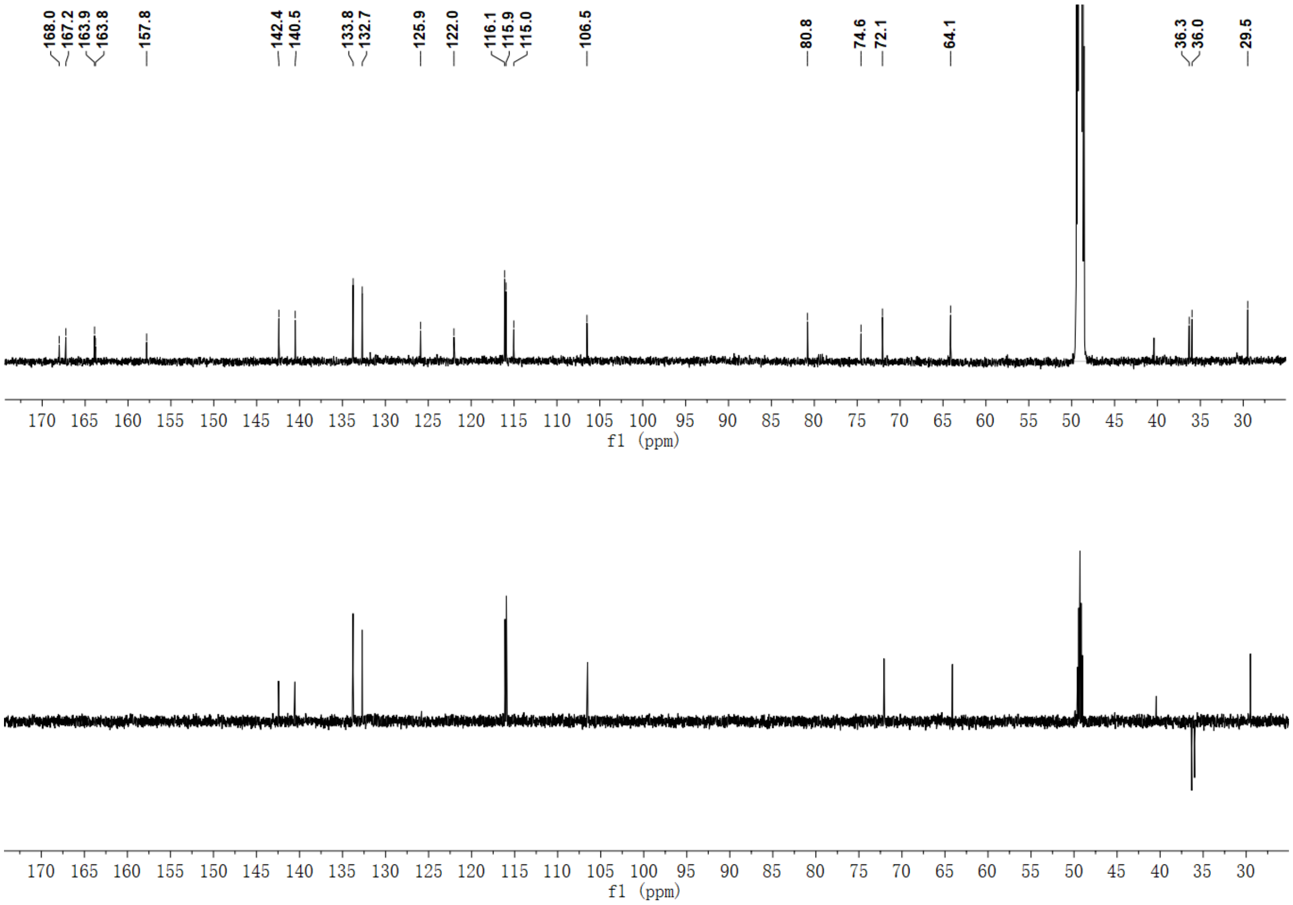

Figure S90. HSQC spectrum of compound 13 (Recorded in CD_3OD)

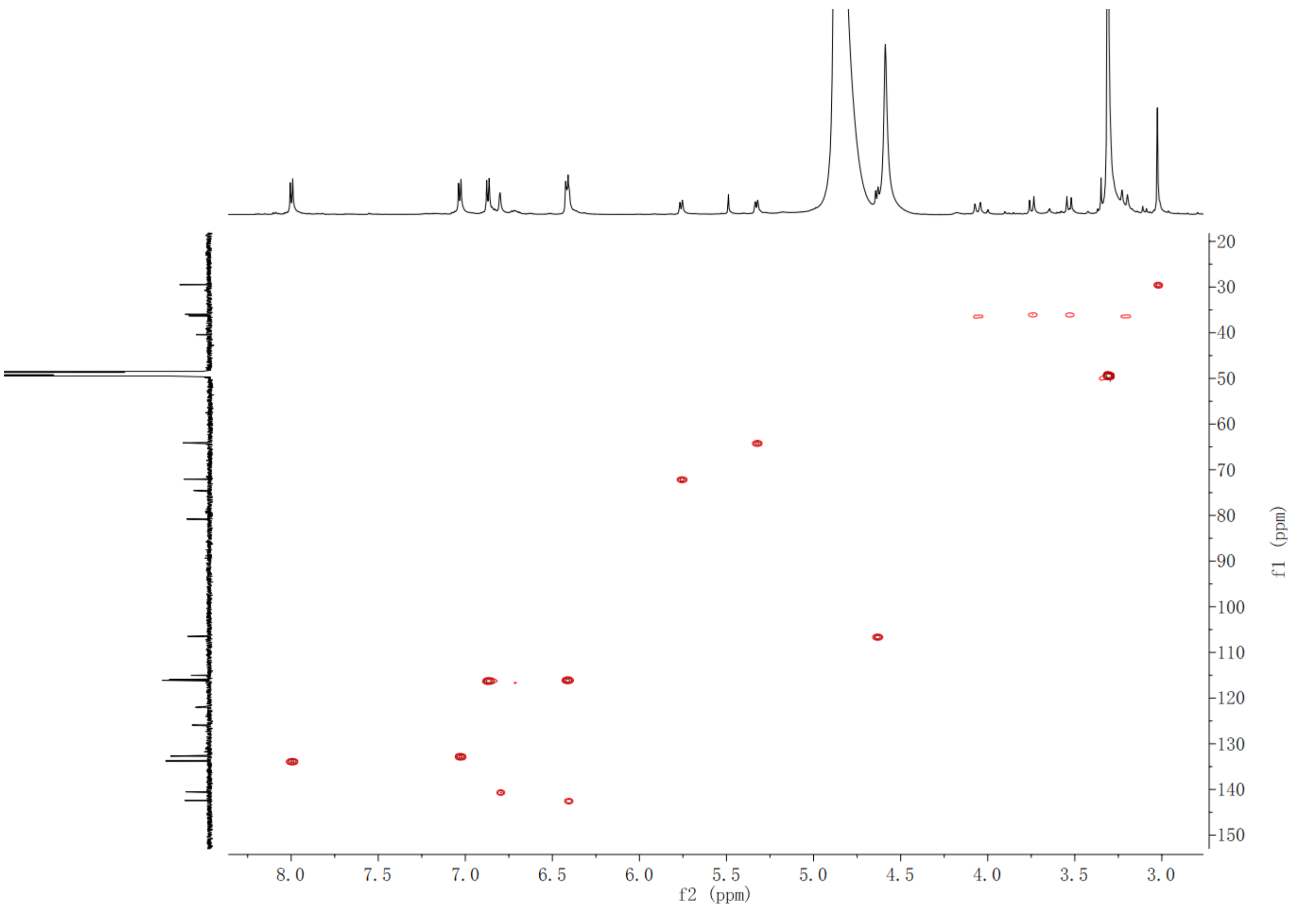

Figure S91. HMBC spectrum of compound 13 (Recorded in CD_3OD)

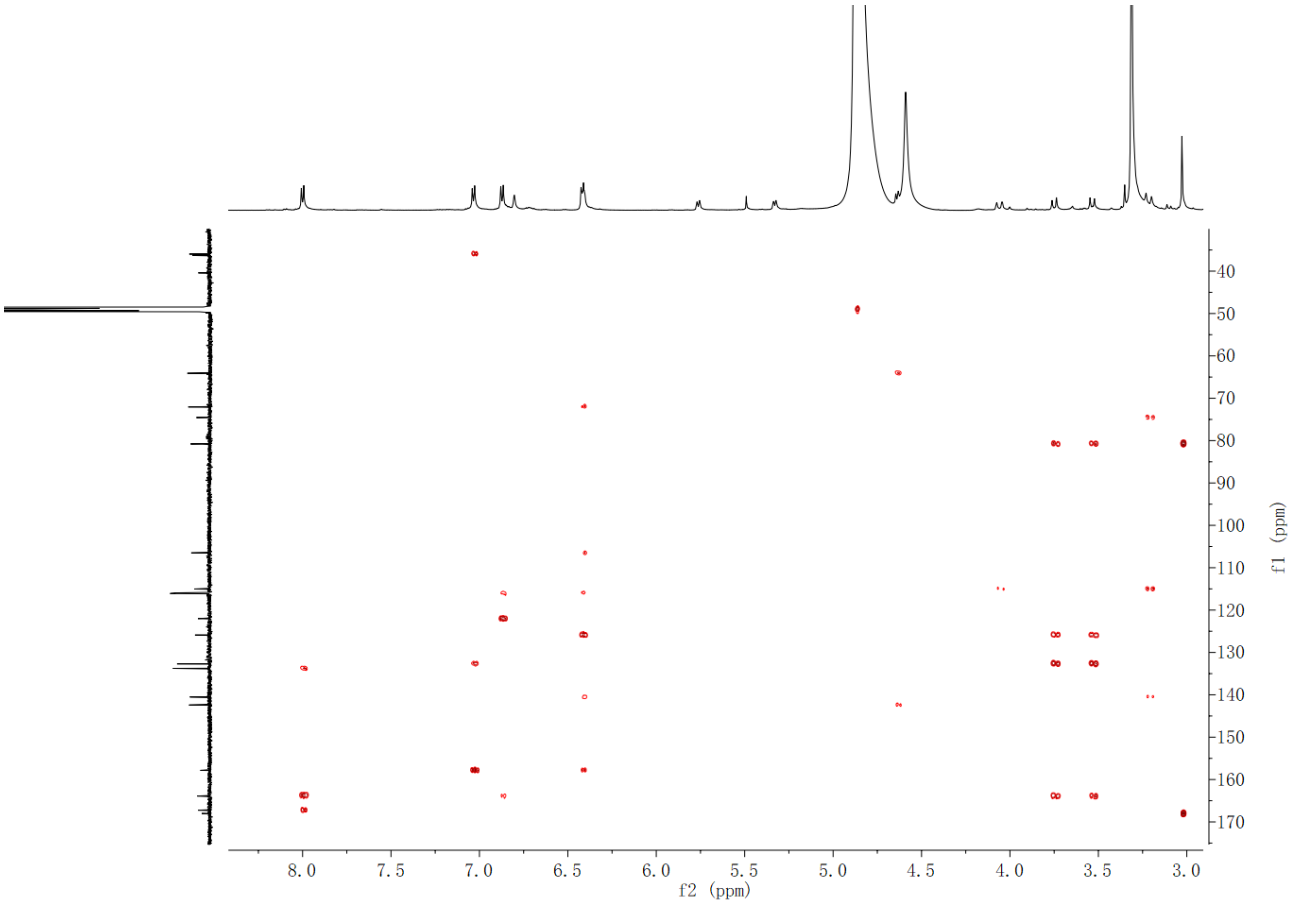

Figure S92. ^{1}H–^{1}H COSY spectrum of compound 13 (Recorded in CD_3OD)

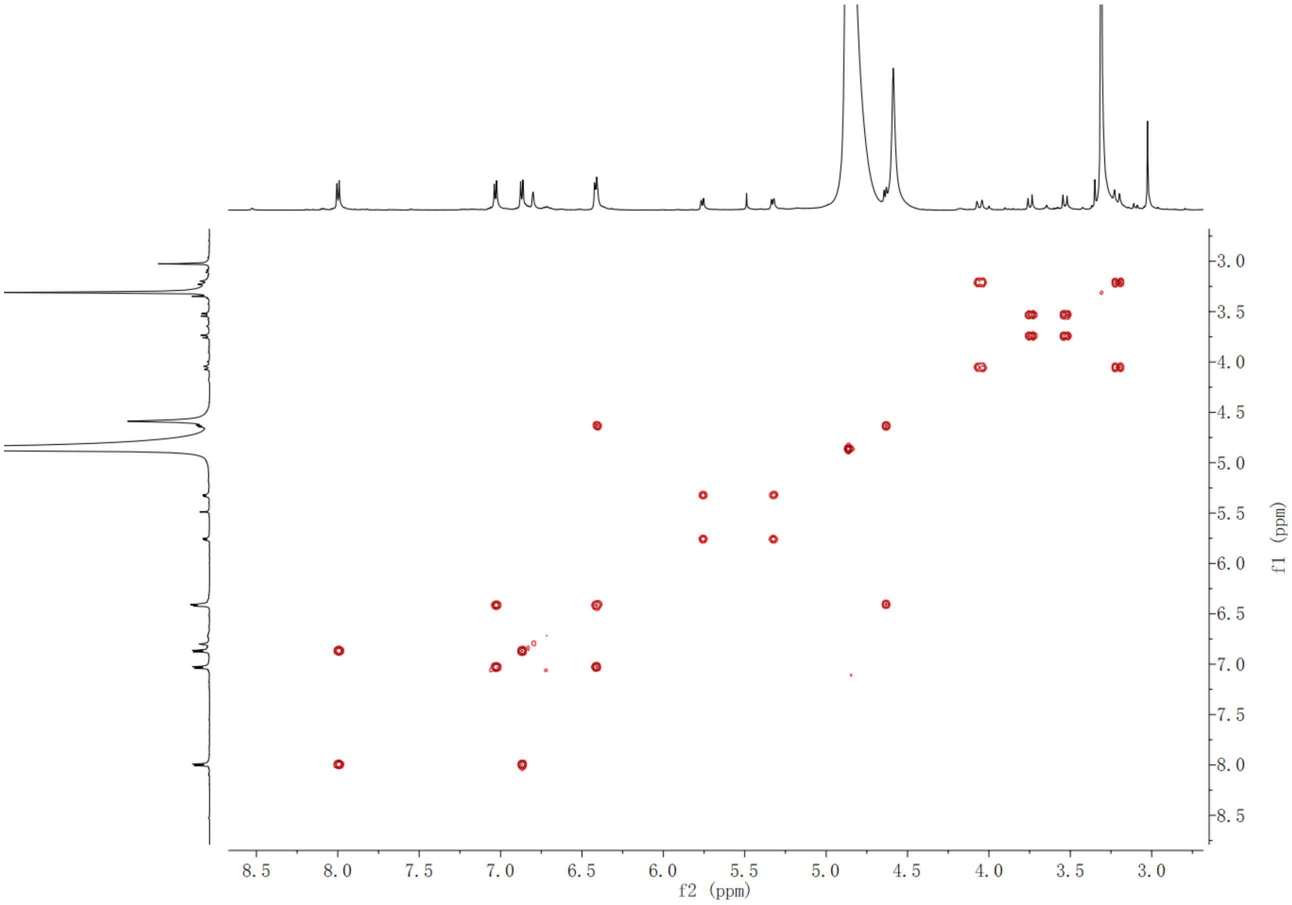

Figure S93. NOESY spectrum of compound 13 (Recorded in CD_3OD)

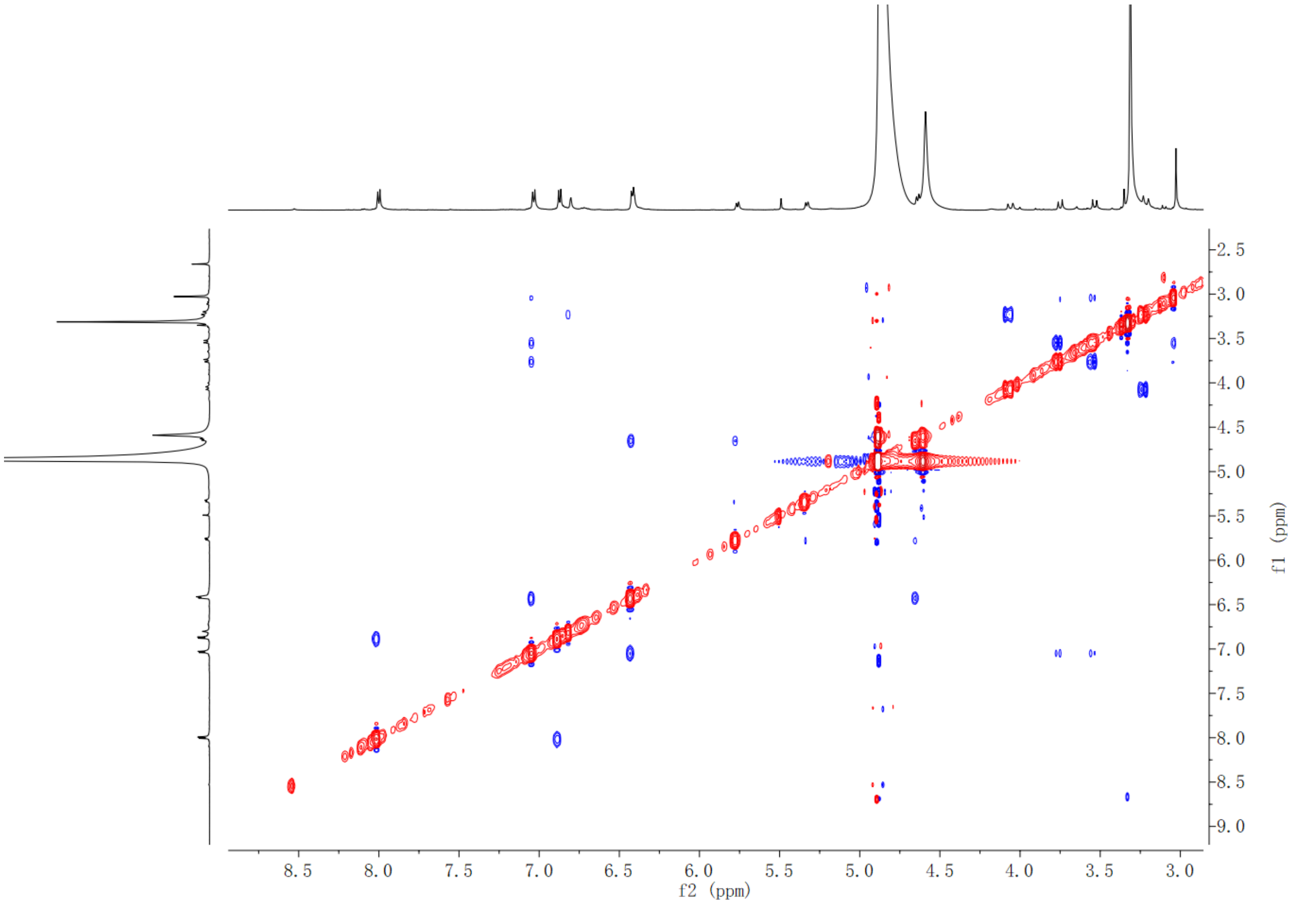

Figure S94. HRESIMS spectrum of compound 13

Mass Spectrum SmartFormula Report

Analysis Info

Acquisition D 2021/9/29 11:26:38

Analysis Name H:\MS\13. LS5-12-1.d

Method Tune_pos_Standard.m

Sample Name

Operator BDAL@DE

Instrument micrOTOF II 8213750.10426

Comment

Acquisition Paramet

Source Type	ESI	Ion Polarity	Positive	Set Nebulizer	0.3 Bar
Focus	Not active			Set Dry Heater	200 °C
Scan Begin	50 m/z	Set Capillary	4500 V	Set Dry Gas	4.0 l/min
Scan End	3050 m/z	Set End Plate Offset	-500 V	Set Divert Valve	Waste

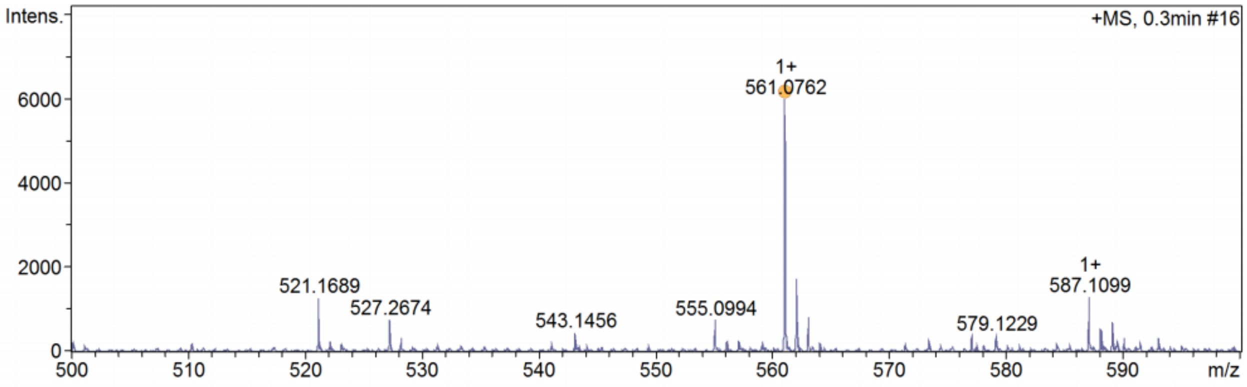

Meas. m/z	#	Ion Formula	m/z	err [ppm]	Mean err [ppm]	rdb	N-Rule	e¡¥ Conf	mSigma	Std I	Std Mean m/z
561.076240	1	C26H22N2NaO7S2	561.076064	-0.3	1747.5	16.5	ok	even	19.9	44.5	n.a.

Figure S95. UV spectrum of compound 13

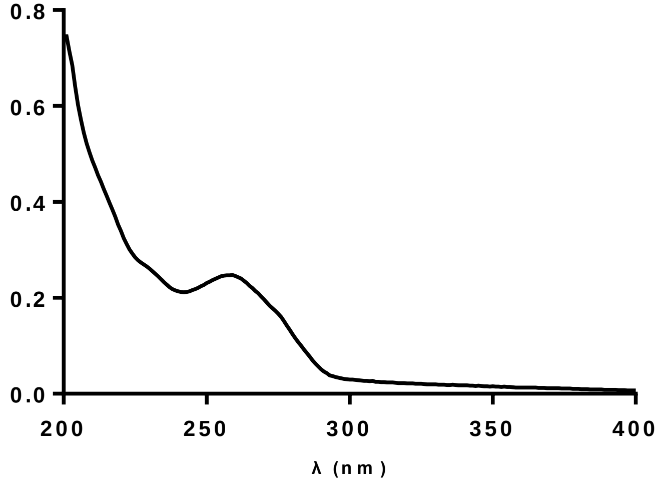

Figure S96. IR spectrum of compound 13

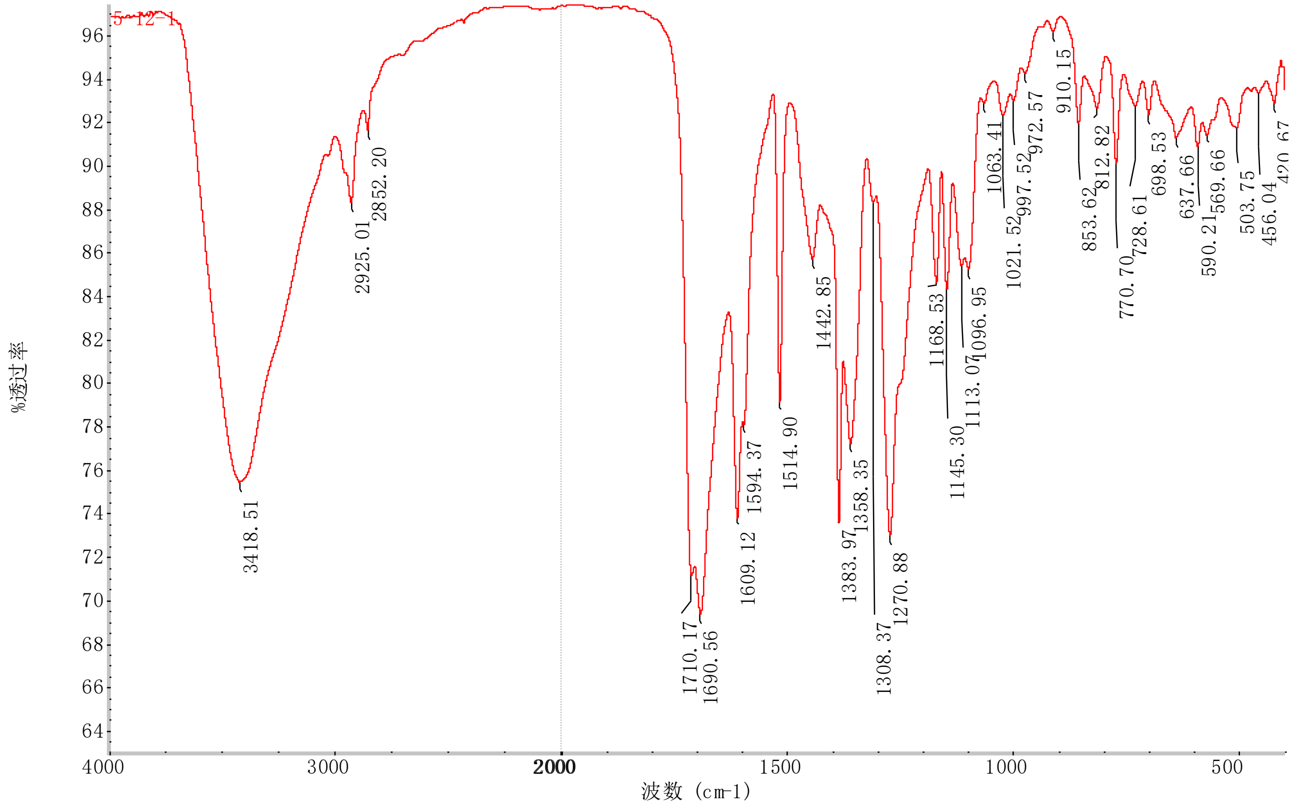

Table S1. ^{1}H NMR spectroscopic data (δ in ppm, J in Hz) for compounds 1, 3–6, and 11–13.

No.	**1**[a,c]	**3**[a,c]	**4**[a,c]	**5**[a,c]	**6**[a,d]	**10**[a,c]	**11**[b,d]	**12**[a,d]	**13**[b,d]
5a	5.34, d (8.9)	5.06, d (8.9)	5.32, d (8.7)	5.27, d (13.1)	5.63, d (7.8)	5.34, d (8.6)	5.32, d (8.4)	5.03, d (8.9)	5.33, d (8.6)
6	5.82, dd (8.9, 1.8)	4.85, dd (8.9, 1.9)	5.85, dd (8.7, 1.9)	6.12, d (13.1)	4.92, dd (7.8, 2.5)	5.45, dd (8.6, 2.2)	5.41, dd (8.4, 2.1)	4.83, dd (8.9, 1.9)	5.76, dd (8.6, 2.3)
7	4.68, dd (8.2, 1.8)	4.99, dd (8.3, 1.9)	4.67, dd (8.2, 1.9)	5.69, m	4.93, dd (8.4, 2.5)	4.80, dd (8.2, 2.2)	4.77, dd (8.1, 2.1)	4.97, dd (8.3, 1.9)	4.64, dd (8.2, 2.3)
8	6.31, dd (8.2, 2.1)	6.37, dd (8.3, 2.2)	6.32, dd (8.2, 2.1)	6.01, m	6.33, dd (8.4, 2.4)	6.26, dd (8.2, 2.1)	6.37, dd, (8.2, 2.3)	6.38, dd (8.3, 2.1)	6.42, dd (8.2, 2.3)
10	6.69, d (2.1)	6.83, d (2.2)	6.67, d (2.1)	6.03, m	6.89, d (2.4,)	6.56, d (2.1)	6.72, d (2.3)	6.83, t (2.1)	6.80, d (2.3)
11	3.18, d (18.5); 4.15, d (18.5)	5.36, s	3.12, d (18.4); 4.12, d (18.4)	2.99, m; 3.81, d (18.9)	5.01, s	3.11, overlap	3.12, overlap; 3.19, d (18.5)	5.36, s	3.22, d (18.5); 4.06, d (18.5)
2′	7.55, d (2.0)	8.17, d (2.0)	8.08, overlap	7.72, d (2.0)	7.77, d (2.1)	7.79, d (2.1)	7.63, d (2.0)	8.11, d (2.0)	8.00, overlap
3′			6.95, overlap						6.87, overlap
5′	6.91, d (8.3)	7.00, d (8.7)	6.95, overlap	6.92, d (8.4)	7.03, d (8.2)	6.95, d (8.4)	6.86, d (8.3)	7.02, d (8.6)	6.87, overlap
6′	7.58, d (8.3, 2.0)	7.79, dd (8.7, 2.0)	8.08, overlap	7.76, dd (8.4, 2.0)	7.76, dd (8.2, 2.1)	7.84, dd (8.4, 2.1)	7.68, dd (8.3, 2.0)	7.80, dd (8.6, 2.0)	8.00, overlap
2″	7.20, overlap		7.06, overlap	7.27, overlap		7.18, dd (7.6, 1.9)	7.24, overlap		7.03, overlap
3″	7.20, overlap	6.86, d (8.3)	6.43, overlap	7.15, overlap	6.92, d (8.3)	7.27, overlap	7.32, overlap	6.90, d (8.4)	6.42, overlap
4″	7.20, overlap	7.12, dd (8.3, 2.2)		7.21, m	7.07, dd (8.3, 2.1)	7.27, overlap	7.28, overlap	7.20, dd (8.4, 2.3)	
5″	7.20, overlap		6.43, overlap	7.15, overlap		7.27, overlap	7.32, overlap		6.42, overlap
6″	7.20, overlap	8.49, d (2.2)	7.06, overlap	7.27, overlap	7.90, d (2.1)	7.18, dd (7.6, 1.9)	7.24, overlap	8.45, d (2.3)	7.03, overlap
7″	3.65, m	4.07, d (14.8); 3.45, d (14.8)	3.51, d (14.8); 3.65, d (14.8)	3.63, d (14.6); 3.76, d (14.6)	3.46, d (13.3); 3.96, d (13.3)	3.27, d (14.8); 4.08, d (14.8)	3.45, d (15.0); 4.05, d (15.0)	5.42, d (12.1)	3.53, d (15.0); 3.75, d (15.0)
N-CH_3	2.93, s	3.24, s	3.02, s	3.04, s	3.22, s	3.14, s	3.09, s	3.22, s	3.03, s
4′-OCH_3		4.04, s	3.89, s	3.96, s	4.02, s	3.99, s		4.05, s	
2″-OH		6.72, s			6.24, s			6.75, s	
3′-OH				5.69, s		5.79, s			
7″-OH								4.97, d (12.1)	

[a]In $CDCl_3$. [b]In CD_3OD. [c]Recorded at 400 MHz. [d]Recorded at 600 MHz.

Table S2. ^{13}C NMR spectroscopic data (δ in ppm) for compounds 1, 3–6, 11–13.

No.	**1**^{a,c}	**3**^{a,c}	**4**^{a,c}	**5**^{a,c}	**6**^{a,d}	**10**^{a,c}	**11**^{b,d}	**12**^{a,d}	**13**^{b,d}
1	166.1, C	167.5, C	165.5, C	166.9, C	167.2, C	168.4, C	169.7, C	167.0, C	168.0, C
3	78.3 C	79.4, C	79.0, C	78.6, C	78.7, C	78.7, C	79.8, C	82.8, C	80.8, C
4	162.3, C	165.5, C	162.4, C	162.4, C	163.3, C	164.1, C	165.8, C	166.1, C	163.8, C
5a	63.2, CH	60.2, CH	63.0, CH	64.2, CH	60.9, CH	61.9, CH	63.2, CH	60.5, CH	64.1, CH
6	70.6, CH	72.6, CH	70.4, CH	75.3, CH	75.2, CH	71.7, CH	72.7, CH	72.6, CH	72.1, CH
7	105.9, CH	105.8, CH	105.8, CH	128.6, CH	108.1, CH	106.8, CH	107.4, CH	105.8, CH	106.5, CH
8	141.3, CH	141.6, CH	141.3, CH	124.7, CH	138.7, CH	139.5, CH	140.9, CH	141.7, CH	142.4, CH
10	139.6, CH	140.8, CH	139.4, CH	120.3, CH	143.0, CH	138.8, CH	140.2, CH	140.9, CH	140.5, CH
10a	113.4, C	116.4, C	113.6, C	132.6, C	113.1, C	108.4, C	110.0, C	116.2, C	115.0, C
11	35.4, CH_2	71.7, CH	35.6, CH_2	36.8, CH_2	77.1, CH	39.5, CH_2	42.1, CH_2	71.4, CH	36.3, CH_2
11a	73.5, C	83.2, C	73.3, C	75.8, C	73.1, C	74.5, C	75.9, C	82.5, C	74.6, C
1′	121.6, C	122.9, C	122.4, C	123.9, C	123.2, C	123.2, C	122.5, C	122.8, C	122.0, C
2′	116.5, CH	121.8, CH	132.6, CH	116.3, CH	121.8, CH	116.6, CH	118.2, CH	121.2, CH	133.8, CH
3′	143.2, C	146.7, C	113.7, CH	145.3, C	145.7, C	145.4, C	146.1, C	146.6, C	116.1, CH
4′	149.6, C	154.4, C	163.6, C	150.3, C	154.1, C	150.9, C	152.1, C	154.4, C	163.9, C
5′	114.7, CH	111.9, CH	113.7, CH	109.9, CH	112.2, CH	110.1, CH	115.8, CH	112.1, CH	116.1, CH
6′	124.3, CH	127.3, CH	132.6, CH	123.4, CH	126.5, CH	124.0, CH	125.0, CH	127.4, CH	133.8, CH
7′	165.7, C	166.1, C	163.6, C	165.6, C	165.5, C	166.4, C	168.2, C	166.1, C	167.2, C
1″	133.8, C	145.1, C	125.6, C	133.5, C	144.7, C	134.6, C	136.2, C	145.4, C	125.9, C
2″	128.8, CH	148.4, C	131.7, CH	130.8, CH	148.2, C	129.4, CH	130.4, CH	149.4, C	132.7, CH
3″	129.0, CH	115.2, CH	115.2, CH	128.4, CH	115.5, CH	129.0, CH	129.9, CH	115.2, CH	115.9, CH
4″	127.5, CH	128.6, CH	155.1, C	127.8, CH	128.0, CH	127.8, CH	128.7, CH	127.1, CH	157.8, C
5″	129.0, CH	124.9, C	115.2, CH	128.4, CH	122.9, C	129.0, CH	129.9, CH	128.8, C	115.9, CH
6″	128.8, CH	129.3, CH	131.7, CH	130.8, CH	126.1, CH	129.4, CH	130.4, CH	125.0, CH	132.7, CH
7″	36.3, CH_2	36.6, CH_2	35.8, CH_2	37.4, CH_2	36.4, CH_2	41.6, CH_2	39.9, CH_2	75.7, CH	36.0, CH_2
N-CH_3	29.5, CH_3	29.1, CH_3	29.3, CH_3	28.9, CH_3	27.9, CH_3	30.7, CH_3	31.2, CH_3	29.1, CH_3	29.5, CH_3
4′-OCH_3		56.6, CH_3	55.7, CH_3	56.2, CH_3	56.4, CH_3	56.2, CH_3		56.6, CH_3	

[a]In $CDCl_3$. [b]In CD_3OD. [c]Recorded at 100 MHz. [d]Recorded at 150 MHz.

Table S3. Gibbs free energies[a] and equilibrium populations[b] of low-energy conformers of 11.

Conformers	ΔG (a.u.)	P (%)/100	G (a.u.)
1	0.0	76.94	-3232.645641
2	0.00231	6.66	-3232.643331
3	0.00146	16.4	-3232.644182

[a]wB97M-V/def2-TZVP, in a.u.

[b]From ΔG values at 298.15 K.

Table S4. Cartesian coordinates for the low-energy reoptimized random research conformers of 11 at B3LYP-D3(BJ)/6-31G* level of theory in methanol.

1		Standard Orientation (A.U.)			
Center number	Atomic number	Atomic Type	X	Y	Z
0	7	0	-6.725683	0.730355	0.831492
1	6	0	-8.567628	-0.977224	1.383224
2	6	0	-7.893416	-3.797225	1.177333
3	7	0	-5.246574	-4.370288	1.096365
4	6	0	-3.38386	-2.58042	1.102961
5	6	0	-4.136079	0.096161	0.437931
6	6	0	-2.687736	2.11687	1.913773
7	6	0	-4.440915	4.363245	1.786908
8	6	0	-7.085179	3.464414	1.229921
9	6	0	-3.600389	6.726844	1.996344
10	8	0	-4.908288	8.947835	1.923328
11	6	0	-7.254806	9.202284	0.867104
12	6	0	-8.631196	7.577542	-0.478242
13	6	0	-8.122587	4.871036	-1.110121
14	8	0	-1.163408	-3.081605	1.485451
15	8	0	-10.693443	-0.36764	2.031393
16	6	0	-13.258331	2.170962	-4.918017
17	6	0	-14.417584	0.604155	-3.135914
18	6	0	-16.444593	-0.902861	-3.842519
19	6	0	-17.3294	-0.85899	-6.315413
20	6	0	-16.156827	0.71614	-8.103638
21	6	0	-14.139847	2.215484	-7.415933
22	6	0	-9.266021	-5.202242	3.341556
23	6	0	-8.676539	-4.097595	5.914208
24	6	0	-6.256517	-4.227594	6.968541
25	6	0	-5.7575	-3.160157	9.320748
26	6	0	-7.670551	-1.956673	10.661579
27	6	0	-10.087128	-1.823858	9.632388
28	6	0	-10.57943	-2.876437	7.276737
29	8	0	-19.313318	-2.342117	-6.979203
30	6	0	-4.440362	-7.022542	1.060331
31	8	0	-10.443386	3.645064	-1.815982
32	6	0	-11.061211	3.767784	-4.30797
33	8	0	-9.901693	5.044344	-5.824847
34	8	0	-17.170544	0.616574	-10.495277
35	1	0	-8.422657	3.707817	2.792557
36	16	0	-3.145295	0.381588	-3.079707
37	16	0	-6.363558	-0.502856	-5.05049
38	16	0	-6.824682	-4.471167	-4.594807
39	16	0	-9.584909	-4.836687	-1.894217
40	1	0	-0.797305	2.468039	1.161301
41	1	0	-2.471897	1.420909	3.860547
42	1	0	-1.620061	7.109596	2.373962

43	1	0	-7.914573	11.115816	1.189514
44	1	0	-10.416725	8.313734	-1.170407
45	1	0	-6.780728	4.689089	-2.676356
46	1	0	-13.709978	0.544204	-1.214353
47	1	0	-17.368014	-2.142009	-2.493755
48	1	0	-13.202202	3.436606	-8.777647
49	1	0	-11.298769	-5.065263	2.996747
50	1	0	-8.755804	-7.204575	3.249429
51	1	0	-4.731096	-5.162596	5.9662
52	1	0	-3.864832	-3.282098	10.106174
53	1	0	-7.281479	-1.128318	12.49883
54	1	0	-11.594933	-0.884637	10.6609
55	1	0	-12.454702	-2.720998	6.457745
56	1	0	-19.658617	-2.074338	-8.754208
57	1	0	-2.62835	-7.131245	0.083819
58	1	0	-4.202804	-7.795029	2.9678
59	1	0	-5.85131	-8.133242	0.04021
60	1	0	-16.245517	1.731647	-11.597321

2		Standard Orientation (A.U.)			
Center number	Atomic number	Atomic Type	X	Y	Z
0	7	0	-6.83441	1.151878	0.885257
1	6	0	-8.783163	-0.466704	1.322638
2	6	0	-8.194225	-3.311279	1.301998
3	7	0	-5.591189	-3.953306	1.63868
4	6	0	-3.659376	-2.238543	1.68929
5	6	0	-4.232365	0.431337	0.835576
6	6	0	-2.9227	2.458119	2.435454
7	6	0	-4.566357	4.74968	2.005963
8	6	0	-7.143577	3.909805	1.135231
9	6	0	-3.69129	7.095851	2.261648
10	8	0	-4.913554	9.34458	1.943869
11	6	0	-7.06839	9.627525	0.542766
12	6	0	-8.301355	7.992626	-0.924292
13	6	0	-7.83028	5.243185	-1.368144
14	8	0	-1.503461	-2.789872	2.298359
15	8	0	-10.938458	0.259142	1.721606
16	6	0	-12.583537	2.519807	-5.630599
17	6	0	-14.034847	1.130415	-3.91647
18	6	0	-16.034904	-0.330717	-4.778688
19	6	0	-16.603368	-0.416983	-7.341701
20	6	0	-15.138608	0.980357	-9.060995
21	6	0	-13.14717	2.433886	-8.217721
22	6	0	-9.946068	-4.674516	3.199463
23	6	0	-9.172706	-4.336244	5.934577
24	6	0	-8.563583	-6.46104	7.378433
25	6	0	-7.839454	-6.231691	9.895427
26	6	0	-7.705315	-3.857977	11.013825

27	6	0	-8.33685	-1.729396	9.605534
28	6	0	-9.076218	-1.962016	7.093339
29	8	0	-18.563571	-1.853479	-8.159227
30	6	0	-4.90263	-6.621423	1.940711
31	8	0	-10.099866	4.067871	-2.293323
32	6	0	-10.403259	4.061668	-4.846151
33	8	0	-9.009047	5.195247	-6.276206
34	8	0	-15.853929	0.764268	-11.550604
35	1	0	-8.663971	4.259616	2.498001
36	16	0	-2.774758	0.61939	-2.507334
37	16	0	-5.701144	-0.302955	-4.875062
38	16	0	-6.312327	-4.230361	-4.254745
39	16	0	-9.451337	-4.366481	-2.000869
40	1	0	-0.9386	2.7249	1.929955
41	1	0	-2.985947	1.823689	4.413841
42	1	0	-1.770438	7.442452	2.894887
43	1	0	-7.689778	11.575253	0.687076
44	1	0	-9.930205	8.763149	-1.90529
45	1	0	-6.319025	4.935587	-2.749042
46	1	0	-13.574972	1.170659	-1.920773
47	1	0	-17.181309	-1.432153	-3.482787
48	1	0	-11.988715	3.519679	-9.522787
49	1	0	-11.852858	-3.935303	2.883001
50	1	0	-9.976127	-6.690373	2.74592
51	1	0	-8.675649	-8.327778	6.524397
52	1	0	-7.380003	-7.912301	10.980505
53	1	0	-7.1323	-3.669255	12.975285
54	1	0	-8.276902	0.130253	10.473069
55	1	0	-9.636596	-0.289428	6.048859
56	1	0	-18.671116	-1.692455	-9.976993
57	1	0	-2.984567	-6.877626	1.230642
58	1	0	-4.957614	-7.18629	3.929749
59	1	0	-6.212472	-7.777652	0.838682
60	1	0	-14.739961	1.755708	-12.594521

3		Standard Orientation (A.U.)			
Center number	Atomic number	Atomic Type	X	Y	Z
0	7	0	-6.643299	0.873486	0.873787
1	6	0	-8.393172	-0.955176	1.327935
2	6	0	-7.542651	-3.722685	1.074912
3	7	0	-4.864478	-4.133488	1.053876
4	6	0	-3.11531	-2.235525	1.16402
5	6	0	-4.010384	0.406804	0.539102
6	6	0	-2.725856	2.47501	2.099263
7	6	0	-4.617255	4.608519	1.997824
8	6	0	-7.179997	3.569342	1.320372
9	6	0	-3.952604	7.010854	2.345035
10	8	0	-5.418252	9.133636	2.313113

11	6	0	-7.705387	9.283488	1.116528
12	6	0	-8.901673	7.626955	-0.356767
13	6	0	-8.216381	4.968693	-1.023609
14	8	0	-0.880796	-2.610978	1.603945
15	8	0	-10.569939	-0.491419	1.926059
16	6	0	-13.059951	2.056192	-5.05299
17	6	0	-14.17664	0.384223	-3.340942
18	6	0	-16.086033	-1.225596	-4.142258
19	6	0	-16.895894	-1.180162	-6.640656
20	6	0	-15.765474	0.500517	-8.358374
21	6	0	-13.865265	2.102932	-7.576322
22	6	0	-8.883771	-5.261126	3.167748
23	6	0	-8.41908	-4.191296	5.780682
24	6	0	-6.022349	-4.217583	6.894018
25	6	0	-5.639799	-3.18402	9.282723
26	6	0	-7.647442	-2.118159	10.601636
27	6	0	-10.041657	-2.089072	9.513585
28	6	0	-10.418219	-3.107579	7.121974
29	8	0	-18.765909	-2.762985	-7.397645
30	6	0	-3.8993	-6.731372	0.982394
31	8	0	-10.440013	3.629119	-1.831922
32	6	0	-10.983794	3.768108	-4.339857
33	8	0	-9.854503	5.142524	-5.792778
34	8	0	-16.696947	0.393054	-10.782915
35	1	0	-8.584921	3.695319	2.837109
36	16	0	-2.94241	0.832644	-2.942907
37	16	0	-6.042824	-0.196667	-5.027672
38	16	0	-6.277328	-4.195714	-4.676533
39	16	0	-9.084975	-4.784625	-2.066071
40	1	0	-0.848078	2.966002	1.394703
41	1	0	-2.500447	1.740956	4.030602
42	1	0	-2.021075	7.515228	2.819954
43	1	0	-8.49525	11.145311	1.448968
44	1	0	-10.6754	8.290151	-1.146049
45	1	0	-6.816334	4.896268	-2.547185
46	1	0	-13.52525	0.32308	-1.399755
47	1	0	-16.973552	-2.547035	-2.848697
48	1	0	-12.961659	3.407367	-8.882573
49	1	0	-10.911989	-5.230342	2.77494
50	1	0	-8.256533	-7.227723	3.038037
51	1	0	-4.423713	-5.043221	5.910212
52	1	0	-3.76321	-3.223822	10.113682
53	1	0	-7.349077	-1.316298	12.467295
54	1	0	-11.622902	-1.257655	10.524252
55	1	0	-12.278405	-3.032993	6.258529
56	1	0	-19.070078	-2.478269	-9.177545
57	1	0	-5.19838	-7.895254	-0.1236
58	1	0	-2.045838	-6.703865	0.081085
59	1	0	-3.693265	-7.542706	2.877126

60	1	0	-15.806957	1.585091	-11.83203

Table S5. Gibbs free energies[a] and equilibrium populations[b] of low-energy conformers of 1a.

Conformers	ΔG(a.u.)	P(%)/100	G(a.u.)
**1a**000001.log	0.0	33.95	-2436.223491
**1a**000002.log	0.00032	24.23	-2436.223173
**1a**000003.log	0.0	34.1	-2436.223495
**1a**000004.log	0.0014	7.72	-2436.222092

[a]wB97M-V/def2-TZVP, in a.u.

[b]From ΔG values at 298.15 K.

Table S6. Cartesian coordinates for the low-energy reoptimized random research conformers of 1a at B3LYP-D3(BJ)/6-31G* level of theory in methanol.

**1a**000001_en_		Standard Orientation (A.U.)			
Center number	Atomic number	Atomic Type	X	Y	Z
0	7	0	-2.824057	-1.83508	-0.640561
1	6	0	-0.304371	-1.561967	-1.117615
2	6	0	1.318129	-2.42782	1.145176
3	7	0	0.581205	-4.897052	1.993872
4	6	0	-1.973114	-5.41536	2.18377
5	6	0	-3.631188	-3.141053	1.578127
6	6	0	-6.41237	-3.759607	1.091572
7	6	0	-7.189844	-1.867562	-0.919745
8	6	0	-4.88345	-0.549191	-1.960506
9	6	0	-9.592014	-1.333964	-1.463558
10	8	0	-10.514242	0.41191	-3.129488
11	6	0	-9.263524	2.585262	-3.771839
12	6	0	-7.044278	3.50644	-2.988964
13	6	0	-5.099471	2.308981	-1.310092
14	8	0	-2.850548	-7.422523	2.898827
15	8	0	0.569667	-0.677693	-3.058727
16	6	0	0.839562	5.780867	-0.291913
17	6	0	2.284749	4.873899	-2.31258
18	6	0	4.817126	5.562939	-2.535068
19	6	0	5.912909	7.167102	-0.757701
20	6	0	4.446976	8.085603	1.266512
21	6	0	1.938173	7.401521	1.500984
22	6	0	4.153179	-2.046839	0.645234
23	6	0	5.497587	-4.047073	-0.914592
24	6	0	7.757618	-5.061925	0.022229
25	6	0	9.105817	-6.872611	-1.336315
26	6	0	8.200663	-7.71205	-3.664767
27	6	0	5.957879	-6.707739	-4.619374
28	6	0	4.619584	-4.8835	-3.266825
29	8	0	8.379771	7.8387	-0.966146
30	6	0	2.310795	-6.455677	3.490557
31	8	0	-2.696999	3.489257	-1.742606
32	6	0	-1.819288	5.040062	0.122161
33	8	0	-3.099356	5.70428	1.910533
34	1	0	-4.60982	-0.759209	-3.994676
35	8	0	5.72457	9.650799	2.907579
36	16	0	-3.213153	-1.08661	4.52165
37	16	0	0.486892	0.144071	3.647729
38	1	0	-7.550754	-3.652289	2.810955
39	1	0	-6.499861	-5.719208	0.426212
40	1	0	-11.144426	-2.368518	-0.612155
41	1	0	-10.438626	3.642892	-5.074287
42	1	0	-6.523484	5.33502	-3.756695

43	1	0	-5.543187	2.518725	0.69191
44	1	0	1.4441	3.590331	-3.661985
45	1	0	5.975711	4.869886	-4.077926
46	1	0	0.789659	8.070763	3.065469
47	1	0	4.315367	-0.203744	-0.282105
48	1	0	5.107734	-1.894429	2.470794
49	1	0	8.465256	-4.428017	1.844853
50	1	0	10.853842	-7.628957	-0.569947
51	1	0	9.235864	-9.13078	-4.727425
52	1	0	5.243588	-7.336947	-6.43861
53	1	0	2.912462	-4.079769	-4.055341
54	1	0	8.767162	8.955277	0.443151
55	1	0	3.077945	-5.335661	5.054836
56	1	0	1.208826	-8.023079	4.240358
57	1	0	3.848155	-7.173464	2.325455
58	1	0	4.594724	10.186451	4.244945

**1a**000002_en_		Standard Orientation (A.U.)			
Center number	Atomic number	Atomic Type	X	Y	Z
0	7	0	3.085076	1.40109	-0.651989
1	6	0	0.511293	1.429773	-0.938743
2	6	0	-0.799547	2.401185	1.475716
3	7	0	0.222141	4.819898	2.186815
4	6	0	2.824426	5.09673	2.147017
5	6	0	4.200983	2.65678	1.457208
6	6	0	6.97508	2.975534	0.719725
7	6	0	7.392811	0.910099	-1.226883
8	6	0	4.879411	-0.113501	-2.112695
9	6	0	9.682349	0.054896	-1.844382
10	8	0	10.322028	-1.835871	-3.481498
11	6	0	8.767708	-3.81439	-4.082823
12	6	0	6.464843	-4.421779	-3.239365
13	6	0	4.74771	-2.98867	-1.502179
14	8	0	3.940863	7.028802	2.720018
15	8	0	-0.610637	0.741542	-2.818963
16	6	0	-1.717657	-5.123472	-0.21773
17	6	0	-2.965159	-4.148294	-2.333631
18	6	0	-5.589972	-3.94607	-2.346801
19	6	0	-6.979818	-4.715384	-0.249499
20	6	0	-5.725337	-5.754434	1.857624
21	6	0	-3.12188	-5.964729	1.873982
22	6	0	-3.674302	2.249168	1.260848
23	6	0	-4.953811	3.652539	-0.889042
24	6	0	-7.350217	2.805255	-1.630222
25	6	0	-8.690793	4.040672	-3.529675
26	6	0	-7.649592	6.145656	-4.73084
27	6	0	-5.258728	6.990281	-4.017878
28	6	0	-3.920218	5.755193	-2.110346

29	8	0	-9.533446	-4.432239	-0.21703
30	6	0	-1.252367	6.58365	3.719567
31	8	0	2.195854	-3.824945	-1.896598
32	6	0	1.065156	-5.097266	0.043006
33	8	0	2.230223	-5.998184	1.804119
34	1	0	4.502717	0.154715	-4.124386
35	8	0	-7.292232	-6.461183	3.812674
36	16	0	3.86395	0.727654	4.495152
37	16	0	0.008506	-0.157577	3.964267
38	1	0	8.243297	2.853636	2.344248
39	1	0	7.184831	4.876556	-0.077556
40	1	0	11.38406	0.912597	-1.087185
41	1	0	9.750818	-5.025222	-5.410642
42	1	0	5.679554	-6.161066	-3.989585
43	1	0	5.200652	-3.28428	0.488298
44	1	0	-1.880473	-3.460516	-3.920081
45	1	0	-6.582976	-3.138355	-3.946893
46	1	0	-2.119514	-6.700012	3.507355
47	1	0	-4.131966	0.242411	1.058905
48	1	0	-4.481767	2.847802	3.070939
49	1	0	-8.162462	1.148171	-0.726382
50	1	0	-10.543533	3.347863	-4.080008
51	1	0	-8.686785	7.108014	-6.218047
52	1	0	-4.416583	8.61186	-4.954242
53	1	0	-2.043057	6.414287	-1.616633
54	1	0	-10.139156	-5.088464	1.391266
55	1	0	-0.053639	8.217645	4.077223
56	1	0	-2.951683	7.173785	2.71079
57	1	0	-1.77044	5.701336	5.520933
58	1	0	-6.287189	-7.120822	5.193673

**1a**000003_en_		Standard Orientation (A.U.)			
Center number	Atomic number	Atomic Type	X	Y	Z
0	7	0	-2.822776	-1.836165	-0.640111
1	6	0	-0.303345	-1.561699	-1.117758
2	6	0	1.320128	-2.42634	1.144817
3	7	0	0.584641	-4.896336	1.993404
4	6	0	-1.969311	-5.415928	2.183937
5	6	0	-3.628691	-3.142297	1.578942
6	6	0	-6.409782	-3.762321	1.093225
7	6	0	-7.188522	-1.871456	-0.91884
8	6	0	-4.883119	-0.551829	-1.959962
9	6	0	-9.591091	-1.339533	-1.462604
10	8	0	-10.51463	0.405558	-3.128614
11	6	0	-9.264976	2.579195	-3.772084
12	6	0	-7.046386	3.502077	-2.989293
13	6	0	-5.10096	2.306342	-1.309881
14	8	0	-2.845511	-7.423622	2.898993

15	8	0	0.569709	-0.677365	-3.059316
16	6	0	0.835927	5.781822	-0.291864
17	6	0	2.281424	4.875813	-2.312714
18	6	0	4.813269	5.566778	-2.535723
19	6	0	5.908133	7.171879	-0.758676
20	6	0	4.44191	8.089326	1.265856
21	6	0	1.933681	7.403312	1.500804
22	6	0	4.154898	-2.04403	0.643535
23	6	0	5.499598	-4.044932	-0.915067
24	6	0	7.758854	-5.060372	0.022962
25	6	0	9.1069	-6.871894	-1.334592
26	6	0	8.202408	-7.7115	-3.663271
27	6	0	5.960482	-6.706487	-4.619136
28	6	0	4.622325	-4.881418	-3.26755
29	8	0	8.374392	7.84552	-0.967648
30	6	0	2.31565	-6.454919	3.488529
31	8	0	-2.69924	3.48798	-1.742219
32	6	0	-1.822307	5.039104	0.122709
33	8	0	-3.102444	5.70212	1.911455
34	1	0	-4.609488	-0.761784	-3.994135
35	8	0	5.718617	9.655602	2.906551
36	16	0	-3.211231	-1.087366	4.521945
37	16	0	0.488761	0.144273	3.647689
38	1	0	-7.547839	-3.654353	2.812784
39	1	0	-6.496684	-5.722302	0.428978
40	1	0	-11.142778	-2.37497	-0.610955
41	1	0	-10.440511	3.635522	-5.075197
42	1	0	-6.526445	5.330481	-3.758049
43	1	0	-5.545089	2.515953	0.692035
44	1	0	1.44155	3.591671	-3.662071
45	1	0	5.972057	4.874489	-4.078771
46	1	0	0.784967	8.071741	3.065505
47	1	0	4.315476	-0.201715	-0.285596
48	1	0	5.110171	-1.889329	2.468525
49	1	0	8.465908	-4.42636	1.845778
50	1	0	10.854312	-7.628751	-0.567325
51	1	0	9.237493	-9.13091	-4.725139
52	1	0	5.246775	-7.335768	-6.43858
53	1	0	2.915802	-4.077094	-4.056827
54	1	0	8.761274	8.961918	0.44197
55	1	0	3.090103	-5.332561	5.047423
56	1	0	1.21281	-8.01815	4.245778
57	1	0	3.847936	-7.179001	2.320556
58	1	0	4.588934	10.189745	4.244669

**1a**000004_en_		Standard Orientation (A.U.)			
Center number	Atomic number	Atomic Type	X	Y	Z
0	7	0	-3.08135	1.395036	0.730282

1	6	0	-0.503851	1.339352	0.976354
2	6	0	0.796439	2.375031	-1.417893
3	7	0	-0.144646	4.869759	-1.967984
4	6	0	-2.735123	5.232381	-1.863792
5	6	0	-4.186051	2.804933	-1.288547
6	6	0	-6.93367	3.172135	-0.481196
7	6	0	-7.396649	0.98612	1.317061
8	6	0	-4.904632	-0.149905	2.124571
9	6	0	-9.706893	0.148633	1.879795
10	8	0	-10.398863	-1.829245	3.386444
11	6	0	-8.892244	-3.874911	3.870846
12	6	0	-6.598233	-4.480864	3.003144
13	6	0	-4.836656	-2.99032	1.361851
14	8	0	-3.79194	7.231641	-2.304161
15	8	0	0.628817	0.552625	2.81188
16	6	0	1.618692	-5.125238	0.040508
17	6	0	2.800786	-4.33408	2.267399
18	6	0	5.421125	-4.099847	2.342956
19	6	0	6.844859	-4.65625	0.205772
20	6	0	5.676537	-5.523925	-2.021198
21	6	0	3.066861	-5.761182	-2.089467
22	6	0	3.666745	2.09389	-1.280752
23	6	0	5.074346	3.361612	0.871762
24	6	0	4.123951	5.376838	2.289568
25	6	0	5.580048	6.488493	4.187217
26	6	0	8.010011	5.6074	4.693563
27	6	0	8.973377	3.591632	3.290453
28	6	0	7.514262	2.478768	1.402784
29	8	0	9.424815	-4.373938	0.057547
30	6	0	1.365762	6.663352	-3.428735
31	8	0	-2.307021	-3.897744	1.737968
32	6	0	-1.166755	-5.081746	-0.256087
33	8	0	-2.320195	-5.880796	-2.068302
34	1	0	-4.508465	0.001593	4.145091
35	8	0	7.090427	-6.083724	-4.101118
36	16	0	-3.972537	1.040303	-4.430378
37	16	0	-0.144294	-0.007308	-4.019228
38	1	0	-8.230781	3.219361	-2.086537
39	1	0	-7.057299	5.01507	0.458541
40	1	0	-11.384894	1.099083	1.182948
41	1	0	-9.910134	-5.143953	5.115552
42	1	0	-5.858992	-6.281726	3.647489
43	1	0	-5.274496	-3.169031	-0.64651
44	1	0	1.678135	-3.797301	3.884506
45	1	0	6.349364	-3.404308	4.038681
46	1	0	2.140837	-6.36566	-3.812779
47	1	0	4.037406	0.06197	-1.1665
48	1	0	4.454432	2.720602	-3.089898
49	1	0	2.224713	6.066288	1.946456

50	1	0	4.800796	8.045034	5.275566
51	1	0	9.140797	6.476064	6.16997
52	1	0	10.864734	2.885563	3.66982
53	1	0	8.26607	0.897206	0.326432
54	1	0	10.07407	-3.856927	1.690456
55	1	0	1.811346	5.871812	-5.290722
56	1	0	0.225512	8.360466	-3.662584
57	1	0	3.107881	7.127518	-2.42674
58	1	0	8.853656	-5.814574	-3.661281

Table S7. Gibbs free energies[a] and equilibrium populations[b] of low-energy conformers of 1b.

Conformers	ΔG(a.u.)	P(%)/100	G(a.u.)
**1b**000001.log	0.00317	3.36	-2436.225814
**1b**000002.log	0.0	96.64	-2436.228985

[a]wB97M-V/def2-TZVP, in a.u.
[b]From ΔG values at 298.15 K.

Table S8. Cartesian coordinates for the low-energy reoptimized random research conformers of 1b at B3LYP-D3(BJ)/6-31G* level of theory in methanol.

**1b**000001_en_		Standard Orientation (A.U.)			
Center number	Atomic number	Atomic Type	X	Y	Z
0	7	0	-3.422668	0.101398	-0.12227
1	6	0	-1.312884	-0.818312	-1.304747
2	6	0	-1.280009	-3.73436	-1.429822
3	7	0	-2.331189	-4.946614	0.77023
4	6	0	-4.530326	-3.969059	1.77904
5	6	0	-5.442532	-1.660985	0.344397
6	6	0	-7.495349	-0.11462	1.621327
7	6	0	-7.008527	2.521353	0.64556
8	6	0	-4.302019	2.75264	-0.246471
9	6	0	-8.756478	4.331459	0.665787
10	8	0	-8.44768	6.821004	0.066065
11	6	0	-6.238105	8.078048	0.537823
12	6	0	-3.958902	7.244012	1.242051
13	6	0	-2.852513	4.638578	1.451895
14	8	0	-5.68851	-4.882917	3.547841
15	8	0	0.323949	0.460892	-2.290608
16	6	0	4.014396	3.85804	0.531233
17	6	0	4.470177	5.675234	-1.336728
18	6	0	6.839935	5.840784	-2.467098
19	6	0	8.747304	4.154021	-1.783821
20	6	0	8.27574	2.316288	0.079718
21	6	0	5.950403	2.20425	1.259752
22	6	0	1.350249	-4.664588	-2.168244
23	6	0	3.434912	-4.438967	-0.197591
24	6	0	5.878093	-5.133053	-0.950697
25	6	0	7.883309	-5.138116	0.758386
26	6	0	7.475504	-4.438435	3.275921
27	6	0	5.061313	-3.691677	4.028178
28	6	0	3.062392	-3.674959	2.306257
29	8	0	11.042699	4.253662	-2.924624
30	6	0	-1.73969	-7.593196	1.310868
31	8	0	-0.285555	4.952973	0.615417
32	6	0	1.518793	3.488812	1.712481
33	8	0	1.068882	2.037047	3.444601
34	1	0	-4.158595	3.417521	-2.201311
35	8	0	10.261357	0.714022	0.59712
36	16	0	-6.813401	-3.015908	-2.713189
37	16	0	-3.393788	-4.368692	-4.32599
38	1	0	-7.203071	-0.259634	3.668912
39	1	0	-9.396005	-0.820136	1.228653
40	1	0	-10.705012	3.952588	1.183354
41	1	0	-6.579827	10.087283	0.326155
42	1	0	-2.581288	8.727519	1.573509

43	1	0	-2.814735	3.906452	3.383537
44	1	0	2.962648	6.931189	-1.917787
45	1	0	7.234413	7.230446	-3.921851
46	1	0	5.575218	0.805904	2.701979
47	1	0	1.206846	-6.642851	-2.760609
48	1	0	1.899431	-3.570146	-3.831264
49	1	0	6.207656	-5.699596	-2.898067
50	1	0	9.750688	-5.720916	0.133111
51	1	0	9.017176	-4.48874	4.631987
52	1	0	4.717448	-3.093922	5.960754
53	1	0	1.225945	-3.036465	2.938175
54	1	0	12.037245	2.868339	-2.2303
55	1	0	-2.89262	-8.147651	2.922266
56	1	0	-2.223278	-8.773555	-0.321223
57	1	0	0.255723	-7.812884	1.777751
58	1	0	9.583766	-0.843212	1.30417

**1b**000002_en_		Standard Orientation (A.U.)			
Center number	Atomic number	Atomic Type	X	Y	Z
0	7	0	-3.595329	0.204013	-0.261028
1	6	0	-1.943814	-1.273368	-1.596843
2	6	0	-2.109449	-4.076957	-0.803079
3	7	0	-2.728975	-4.428104	1.83766
4	6	0	-4.653392	-2.999055	2.860953
5	6	0	-5.662068	-1.07817	0.985966
6	6	0	-7.295531	1.034442	2.065198
7	6	0	-6.691727	3.255749	0.373851
8	6	0	-4.157323	2.852695	-0.868121
9	6	0	-8.106051	5.327452	0.178784
10	8	0	-7.511114	7.501224	-1.086368
11	6	0	-5.087983	8.404877	-1.251129
12	6	0	-2.874749	7.374707	-0.593108
13	6	0	-2.255533	4.741543	0.270271
14	8	0	-5.50117	-3.246952	4.989379
15	8	0	-0.582282	-0.549839	-3.30089
16	6	0	4.58437	3.473264	0.006266
17	6	0	4.719892	2.075431	-2.236446
18	6	0	7.016364	1.058845	-3.014185
19	6	0	9.179307	1.419966	-1.561516
20	6	0	9.039793	2.841657	0.683194
21	6	0	6.768763	3.863188	1.464409
22	6	0	0.268196	-5.467671	-1.659703
23	6	0	2.725964	-4.682035	-0.38611
24	6	0	4.962852	-5.795858	-1.266032
25	6	0	7.291007	-5.187951	-0.202205
26	6	0	7.424423	-3.456136	1.784062
27	6	0	5.216452	-2.33873	2.672741
28	6	0	2.887211	-2.93345	1.58759

29	8	0	11.408373	0.368002	-2.275288
30	6	0	-2.037542	-6.774798	3.123719
31	8	0	0.225592	4.090455	-0.619415
32	6	0	2.178264	4.46364	1.010127
33	8	0	1.922076	5.479247	3.060743
34	1	0	-4.195233	3.098002	-2.920559
35	8	0	11.290182	3.037393	1.984047
36	16	0	-7.688336	-2.947462	-1.321189
37	16	0	-4.820442	-5.322823	-2.853504
38	1	0	-6.688838	1.356152	4.021892
39	1	0	-9.300209	0.542172	2.129176
40	1	0	-9.967321	5.473151	1.028268
41	1	0	-5.165676	10.31111	-1.998477
42	1	0	-1.245127	8.595371	-0.835731
43	1	0	-2.249783	4.570335	2.328899
44	1	0	3.018368	1.723134	-3.313166
45	1	0	7.154608	-0.076493	-4.714221
46	1	0	6.61942	4.928288	3.21356
47	1	0	-0.02204	-7.503378	-1.427219
48	1	0	0.440993	-5.109228	-3.686577
49	1	0	4.873118	-7.144873	-2.81445
50	1	0	9.003724	-6.049583	-0.934711
51	1	0	9.239887	-2.95371	2.598299
52	1	0	5.292087	-0.960135	4.188732
53	1	0	1.202309	-2.014081	2.301566
54	1	0	12.672215	0.866579	-1.035785
55	1	0	0.012905	-6.962846	3.221625
56	1	0	-2.818772	-6.669534	5.024263
57	1	0	-2.857381	-8.400263	2.135989
58	1	0	11.051875	4.097138	3.457699

Table S9. Gibbs free energies[a] and equilibrium populations[b] of low-energy conformers of 3a.

Conformers	ΔG(a.u.)	P(%)/100	G(a.u.)
**3a**000001.out	0.01091	0.0	-2624.757623
**3a**000003.out	0.0	95.63	-2624.768537
**3a**000004.out	0.019	0.0	-2624.749534
**3a**000005.out	0.00844	0.01	-2624.760097
**3a**000006.out	0.00847	0.01	-2624.760063
**3a**000007.out	0.01572	0.0	-2624.752818
**3a**000008.out	0.00399	1.4	-2624.764551
**3a**000010.out	0.01562	0.0	-2624.752912
**3a**000011.out	0.00359	2.14	-2624.76495
**3a**000012.out	0.01379	0.0	-2624.754746
**3a**000013.out	0.01919	0.0	-2624.749352
**3a**000014.out	0.00475	0.62	-2624.763788
**3a**000015.out	0.01462	0.0	-2624.753912
**3a**000016.out	0.00835	0.01	-2624.76019
**3a**000017.out	0.01921	0.0	-2624.749326
**3a**000018.out	0.00723	0.04	-2624.761304
**3a**000019.out	0.01096	0.0	-2624.757572
**3a**000020.out	0.00635	0.12	-2624.762191

[a]wB97M-V/def2-TZVP, in a.u.

[b]From ΔG values at 298.15 K.

Table S10. Cartesian coordinates for the low-energy reoptimized random research conformers of 3a at B3LYP-D3(BJ)/6-31G* level of theory in chloroform.

**3a**000001_en_		Standard Orientation (A.U.)			
Center number	Atomic number	Atomic Type	X	Y	Z
0	16	0	-1.5849	-3.942907	-1.549941
1	16	0	-5.055414	-2.291965	-2.816981
2	7	0	-4.746697	-4.790941	2.628459
3	8	0	10.573149	0.700747	-2.543307
4	8	0	0.221406	4.650357	1.633552
5	8	0	-9.643091	0.536503	-1.969622
6	1	0	-10.679112	-0.923716	-1.576927
7	8	0	-0.050767	6.396487	-2.269582
8	8	0	7.80187	-0.873258	1.322305
9	1	0	3.51969	1.703774	2.094898
10	8	0	-0.308215	-0.128919	3.883112
11	8	0	9.05693	-4.481696	-2.182304
12	1	0	9.833375	-2.8565	-1.772967
13	7	0	-3.937015	0.35714	1.555839
14	6	0	12.044672	1.285911	-4.700856
15	1	0	12.769397	3.238471	-4.612129
16	1	0	10.949747	1.035886	-6.457811
17	1	0	13.643301	-0.035411	-4.694019
18	6	0	8.408889	2.012082	-2.109943
19	6	0	7.527304	4.014788	-3.608106
20	1	0	4.435273	6.599952	-4.313206
21	6	0	5.186524	5.116186	-3.093504
22	1	0	8.624205	4.671467	-5.222365
23	6	0	3.746353	4.294066	-1.03357
24	6	0	1.138156	5.276127	-0.670515
25	6	0	-2.451169	4.898983	2.146602
26	1	0	-2.57905	4.305628	4.132207
27	6	0	-3.915846	2.982748	0.540355
28	1	0	-2.987327	2.910322	-1.327376
29	6	0	-6.694114	3.441385	0.079571
30	8	0	-7.166749	7.989148	-0.465168
31	6	0	-7.954666	5.560952	-0.473059
32	1	0	-9.93685	5.445332	-1.039706
33	6	0	-8.089692	0.938075	0.136324
34	1	0	-9.211529	0.801271	1.893761
35	6	0	-5.955439	-1.001331	0.407216
36	6	0	-3.19989	7.621807	1.924032
37	1	0	-5.133014	10.907871	0.801854
38	6	0	-5.09763	8.844466	0.784143
39	1	0	-1.813784	8.87645	2.805723
40	6	0	6.950717	1.192813	-0.032358
41	6	0	6.27288	-2.999801	1.079542
42	6	0	4.086827	-3.27531	2.496654

43	1	0	3.609763	-1.861183	3.906606
44	6	0	2.430373	-5.288185	2.035915
45	6	0	-0.121551	-5.427906	3.31906
46	1	0	-0.004919	-4.760058	5.285627
47	1	0	-0.711684	-7.410318	3.357426
48	6	0	-2.235845	-3.879159	2.034423
49	6	0	-2.013694	-1.016026	2.630275
50	6	0	-5.241548	-7.398712	3.369087
51	1	0	-4.477824	-8.722475	1.953951
52	1	0	-7.299658	-7.616613	3.455587
53	1	0	-4.432744	-7.829571	5.232482
54	6	0	-6.754279	-3.362351	1.816207
55	6	0	3.143574	-7.10639	0.237337
56	1	0	5.925044	-8.283498	-2.544194
57	6	0	5.388305	-6.880051	-1.130762
58	1	0	1.903273	-8.716262	-0.132253
59	6	0	6.960978	-4.780516	-0.775491
60	6	0	4.669082	2.357381	0.521867
61	8	0	-8.976064	-3.934729	2.047191

**3a**000003_en_		Standard Orientation (A.U.)			
Center number	Atomic number	Atomic Type	X	Y	Z
0	16	0	-5.283038	-5.317732	-2.595978
1	16	0	-8.019891	-2.954254	-0.81496
2	7	0	-2.811267	-4.478068	1.950541
3	8	0	11.286814	1.33801	0.355546
4	8	0	-0.13533	4.339218	-0.782754
5	8	0	-9.684277	0.561561	2.89745
6	1	0	-9.616947	-0.58598	4.325676
7	8	0	0.995531	7.485997	1.857913
8	8	0	7.608371	-0.870115	-2.160675
9	1	0	3.127623	1.507488	-2.019867
10	8	0	-0.809938	-0.742694	-3.346868
11	8	0	9.868129	-3.926593	1.345428
12	1	0	10.441316	-2.375551	0.523147
13	7	0	-3.736766	0.115714	-0.298951
14	6	0	13.348481	2.370635	1.714358
15	1	0	12.903728	2.562835	3.742801
16	1	0	13.896774	4.230803	0.949877
17	1	0	14.925746	1.044567	1.480579
18	6	0	9.047038	2.58828	0.339638
19	6	0	8.58112	4.881219	1.584207
20	1	0	5.74451	7.647767	2.608695
21	6	0	6.151911	5.906868	1.581363
22	1	0	10.087029	5.838769	2.611441
23	6	0	4.180193	4.685678	0.308493
24	6	0	1.576959	5.699148	0.554283
25	6	0	-2.730874	4.881833	-0.266813

26	1	0	-2.902205	5.113612	1.794193
27	6	0	-4.449427	2.674004	-1.123543
28	1	0	-4.581527	2.699424	-3.197156
29	6	0	-6.945406	3.083627	0.195919
30	8	0	-8.222616	6.96962	-1.7758
31	6	0	-8.553633	4.99077	-0.16874
32	1	0	-10.356687	5.088967	0.829691
33	6	0	-7.192341	1.137653	2.267218
34	1	0	-6.120947	1.788386	3.949327
35	6	0	-5.686437	-1.068389	1.159324
36	6	0	-3.574125	7.227026	-1.646086
37	1	0	-6.163151	9.668234	-3.45853
38	6	0	-5.904485	7.958686	-2.332091
39	1	0	-2.055098	8.476421	-2.264929
40	6	0	7.064986	1.394057	-0.990587
41	6	0	6.2813	-2.943389	-1.249004
42	6	0	3.83748	-3.45342	-2.038883
43	1	0	2.970969	-2.259261	-3.466149
44	6	0	2.463209	-5.460899	-0.989504
45	6	0	-0.236431	-5.932579	-1.829909
46	1	0	-0.762845	-7.90455	-1.452189
47	1	0	-0.351992	-5.668065	-3.887924
48	6	0	-2.347183	-4.230769	-0.738243
49	6	0	-2.124202	-1.440337	-1.601034
50	6	0	-2.184947	-6.801803	3.296005
51	1	0	-2.906105	-8.456573	2.258146
52	1	0	-3.117348	-6.704302	5.142547
53	1	0	-0.140922	-6.974006	3.569
54	6	0	-4.624821	-2.946135	3.016963
55	6	0	3.720778	-7.03928	0.734403
56	1	0	7.162057	-7.798058	2.841955
57	6	0	6.205812	-6.564864	1.492418
58	1	0	2.764926	-8.70678	1.487494
59	6	0	7.510928	-4.460751	0.555119
60	6	0	4.662485	2.442696	-1.020319
61	8	0	-5.415069	-3.103283	5.176475

**3a**000004_en_		Standard Orientation (A.U.)			
Center number	Atomic number	Atomic Type	X	Y	Z
0	16	0	-3.278278	-5.318535	-1.799461
1	16	0	-6.569271	-2.956709	-2.220358
2	7	0	-4.25461	-4.469232	3.230858
3	8	0	11.323969	1.511548	-1.559572
4	8	0	0.08912	4.567251	1.479631
5	8	0	-10.413806	0.346234	-0.358384
6	1	0	-11.282181	-0.917416	0.644692
7	8	0	0.340335	5.943856	-2.557146
8	8	0	8.040028	-0.820992	1.69471

9	1	0	3.603208	1.517402	2.211304
10	8	0	0.369375	-0.495098	0.173837
11	8	0	9.266794	-3.281486	-2.868714
12	1	0	10.136695	-1.827995	-2.130892
13	7	0	-3.804092	0.193897	0.947148
14	6	0	13.107734	1.653216	0.464575
15	1	0	13.520887	3.643824	0.9242
16	1	0	14.840168	0.728713	-0.208016
17	1	0	12.398827	0.670553	2.156391
18	6	0	9.008263	2.595812	-1.112057
19	6	0	8.222146	4.690421	-2.52211
20	1	0	5.048652	7.067981	-3.536085
21	6	0	5.742078	5.555221	-2.318233
22	1	0	9.548996	5.524375	-3.863727
23	6	0	4.07929	4.430327	-0.582413
24	6	0	1.340481	5.088421	-0.690204
25	6	0	-2.632229	4.823636	1.521117
26	1	0	-3.085871	4.438062	3.514273
27	6	0	-3.989853	2.797807	-0.086697
28	1	0	-3.178217	2.84944	-2.004061
29	6	0	-6.819326	3.202189	-0.15902
30	8	0	-7.185888	7.470053	-1.727975
31	6	0	-8.111483	5.189747	-1.029098
32	1	0	-10.164477	5.096097	-1.228752
33	6	0	-8.1805	0.939152	0.923973
34	1	0	-8.571075	1.272684	2.952439
35	6	0	-6.166601	-1.129475	0.840571
36	6	0	-3.321852	7.49787	0.848587
37	1	0	-5.103264	10.549212	-0.98685
38	6	0	-5.127861	8.522231	-0.598351
39	1	0	-1.956226	8.86944	1.570904
40	6	0	7.329249	1.44957	0.589842
41	6	0	6.556811	-2.812342	0.789238
42	6	0	4.365109	-3.494042	2.054477
43	1	0	3.940309	-2.545438	3.835906
44	6	0	2.645123	-5.19431	0.988905
45	6	0	0.141788	-5.634411	2.274453
46	1	0	0.35634	-5.197343	4.296555
47	1	0	-0.370393	-7.644246	2.147637
48	6	0	-2.209822	-4.104618	1.415261
49	6	0	-1.671333	-1.277472	0.840351
50	6	0	-4.48398	-6.816782	4.665737
51	1	0	-6.317329	-6.758477	5.626268
52	1	0	-2.978828	-7.005273	6.080302
53	1	0	-4.44318	-8.459277	3.385759
54	6	0	-6.416747	-3.076709	2.909165
55	6	0	3.28966	-6.342612	-1.306433
56	1	0	6.038481	-6.619267	-4.34066
57	6	0	5.54228	-5.745621	-2.538748

58	1	0	1.999577	-7.701367	-2.17715
59	6	0	7.18979	-3.918534	-1.549639
60	6	0	4.917532	2.438575	0.936833
61	8	0	-8.386928	-3.374765	4.067558

**3a**000005_en_		Standard Orientation (A.U.)			
Center number	Atomic number	Atomic Type	X	Y	Z
0	16	0	-4.423699	-4.327336	-3.137575
1	16	0	-7.518121	-2.55897	-1.307945
2	7	0	-2.612875	-4.132551	1.83
3	8	0	10.501898	1.713974	-2.944654
4	8	0	-0.325171	5.562712	-0.419714
5	8	0	-9.720067	0.44876	2.616288
6	1	0	-9.642797	-0.811947	3.946845
7	8	0	0.45605	3.7798	3.374318
8	8	0	8.343003	-0.92685	1.385938
9	1	0	4.449132	1.379659	3.263499
10	8	0	0.101047	0.313439	-2.418242
11	8	0	9.919508	-5.269291	3.383775
12	1	0	10.544372	-3.54592	3.418452
13	7	0	-3.52272	0.696978	-0.086518
14	6	0	12.414215	0.274551	-1.730895
15	1	0	12.866768	1.058517	0.146246
16	1	0	14.088157	0.409985	-2.951724
17	1	0	11.874386	-1.720929	-1.513332
18	6	0	8.407	2.466649	-1.709763
19	6	0	7.054702	4.485003	-2.808599
20	1	0	3.626911	6.685533	-2.831448
21	6	0	4.742284	5.261538	-1.845041
22	1	0	7.838974	5.32097	-4.523645
23	6	0	3.811477	4.172467	0.390775
24	6	0	1.201549	4.509334	1.33725
25	6	0	-2.962788	5.601379	0.155813
26	1	0	-3.150782	5.636492	2.223407
27	6	0	-4.402289	3.244948	-0.850898
28	1	0	-4.419221	3.341545	-2.928383
29	6	0	-7.010442	3.361263	0.306151
30	8	0	-8.590198	7.193773	-1.564256
31	6	0	-8.793222	5.099482	-0.084887
32	1	0	-10.657681	4.936917	0.783239
33	6	0	-7.246361	1.260754	2.216675
34	1	0	-6.340996	1.856876	4.012203
35	6	0	-5.507817	-0.725712	1.060228
36	6	0	-4.019212	7.916668	-1.105644
37	1	0	-6.737761	10.206997	-2.91999
38	6	0	-6.370418	8.454751	-1.893414
39	1	0	-2.599929	9.344047	-1.561675
40	6	0	7.411547	1.352853	0.494624

41	6	0	6.751586	-2.992562	1.088778
42	6	0	4.448711	-2.931802	-0.179318
43	1	0	3.784192	-1.214581	-1.087708
44	6	0	2.935839	-5.103073	-0.315093
45	6	0	0.448805	-5.055412	-1.740897
46	1	0	-0.207528	-7.008986	-1.990194
47	1	0	0.77474	-4.283282	-3.643973
48	6	0	-1.80398	-3.520783	-0.698658
49	6	0	-1.560412	-0.631504	-1.147351
50	6	0	-2.079195	-6.592512	2.958065
51	1	0	-2.604918	-8.116034	1.63963
52	1	0	-3.24215	-6.740606	4.665228
53	1	0	-0.078421	-6.764823	3.462517
54	6	0	-4.57569	-2.751442	2.832755
55	6	0	3.853758	-7.346003	0.756117
56	1	0	6.908497	-9.166028	2.820696
57	6	0	6.191593	-7.420126	1.987745
58	1	0	2.741749	-9.080945	0.62097
59	6	0	7.659532	-5.237235	2.185786
60	6	0	5.227046	2.307303	1.595929
61	8	0	-5.607864	-3.157197	4.853661

**3a**000006_en_		Standard Orientation (A.U.)			
Center number	Atomic number	Atomic Type	X	Y	Z
0	16	0	-5.000093	-5.762043	-1.822397
1	16	0	-7.824138	-3.324073	-0.283675
2	7	0	-2.634888	-4.438033	2.642814
3	8	0	11.668338	2.682105	-1.362573
4	8	0	-0.206537	4.167954	-1.137482
5	8	0	-9.628525	0.471927	3.076693
6	1	0	-9.533096	-0.524051	4.612901
7	8	0	0.946928	7.658797	1.017348
8	8	0	7.598738	-0.827868	-2.550228
9	1	0	3.112395	1.271279	-2.059731
10	8	0	-0.694348	-1.161097	-2.989602
11	8	0	9.905394	-2.503288	1.705404
12	1	0	10.476499	-3.158174	3.311451
13	7	0	-3.65168	-0.070908	-0.04214
14	6	0	12.579291	0.695653	-2.929866
15	1	0	11.64381	0.711144	-4.788586
16	1	0	12.300438	-1.156247	-2.039938
17	1	0	14.608341	1.068501	-3.176117
18	6	0	9.198804	3.246728	-1.166895
19	6	0	8.645382	5.675856	-0.217037
20	1	0	5.767492	8.350328	0.906676
21	6	0	6.177015	6.487294	0.123171
22	1	0	10.249836	6.879014	0.266582
23	6	0	4.15805	4.904735	-0.563484

24	6	0	1.533988	5.766342	-0.127982
25	6	0	-2.785513	4.693821	-0.551582
26	1	0	-2.886691	5.12254	1.48342
27	6	0	-4.455184	2.365478	-1.127649
28	1	0	-4.610605	2.158533	-3.190144
29	6	0	-6.956101	2.82841	0.166006
30	8	0	-8.388064	6.449547	-2.170143
31	6	0	-8.636762	4.627751	-0.377113
32	1	0	-10.440849	4.751861	0.616898
33	6	0	-7.153094	1.076577	2.407329
34	1	0	-6.118371	1.916108	4.027739
35	6	0	-5.567711	-1.169064	1.516511
36	6	0	-3.747795	6.871111	-2.120483
37	1	0	-6.434298	9.051331	-4.111429
38	6	0	-6.107741	7.45945	-2.839549
39	1	0	-2.285497	8.118791	-2.865456
40	6	0	7.152615	1.642248	-1.774132
41	6	0	6.422483	-2.639103	-1.090185
42	6	0	4.053317	-3.544516	-1.760032
43	1	0	3.188579	-2.827093	-3.48016
44	6	0	2.737269	-5.260499	-0.222613
45	6	0	0.087102	-6.082771	-0.94833
46	1	0	-0.27274	-8.020608	-0.299257
47	1	0	-0.053431	-6.108837	-3.019737
48	6	0	-2.132835	-4.403861	-0.052022
49	6	0	-1.991255	-1.702743	-1.179421
50	6	0	-2.144278	-6.705377	4.135888
51	1	0	-2.830982	-8.391664	3.125905
52	1	0	-3.192872	-6.504865	5.910654
53	1	0	-0.131945	-6.908743	4.568253
54	6	0	-4.484524	-2.840247	3.547164
55	6	0	3.985414	-6.18592	1.921445
56	1	0	7.326242	-6.068691	4.276679
57	6	0	6.392616	-5.325377	2.587681
58	1	0	3.106736	-7.635171	3.093892
59	6	0	7.616331	-3.495995	1.126316
60	6	0	4.67552	2.502267	-1.535897
61	8	0	-5.304188	-2.827858	5.700435

**3a**000007_en_		Standard Orientation (A.U.)			
Center number	Atomic number	Atomic Type	X	Y	Z
0	16	0	-2.645142	-4.50739	-0.973621
1	16	0	-6.411268	-3.183589	-0.850873
2	7	0	-3.590074	-3.664343	4.229766
3	8	0	11.045296	1.176902	0.718376
4	8	0	0.068762	5.445188	0.445376
5	8	0	-10.30443	0.328715	0.572717
6	1	0	-10.139413	0.107513	-1.239505

7	8	0	0.194143	4.09608	-3.604591
8	8	0	7.937882	-1.497183	-2.292378
9	1	0	3.742199	0.817931	-3.589987
10	8	0	0.737892	0.53082	1.662919
11	8	0	7.778735	-5.720181	-5.147115
12	1	0	8.779974	-4.18335	-5.201259
13	7	0	-3.582217	0.781241	1.331208
14	6	0	12.627412	2.365719	2.505359
15	1	0	13.231396	4.258502	1.86395
16	1	0	14.299826	1.15451	2.706793
17	1	0	11.689854	2.550138	4.361761
18	6	0	8.813474	2.255808	0.129551
19	6	0	7.904304	4.535722	1.150257
20	1	0	4.716437	7.052389	1.533593
21	6	0	5.468243	5.383526	0.584099
22	1	0	9.05559	5.598501	2.48668
23	6	0	3.980641	4.079759	-1.164927
24	6	0	1.260549	4.579865	-1.639719
25	6	0	-2.648576	5.550953	0.457457
26	1	0	-3.082174	5.943961	2.452986
27	6	0	-4.008514	3.052043	-0.283439
28	1	0	-3.416662	2.591224	-2.220788
29	6	0	-6.841934	3.348947	-0.126745
30	8	0	-7.750726	7.034357	-2.716325
31	6	0	-8.360813	5.022019	-1.266599
32	1	0	-10.409977	4.832368	-1.095024
33	6	0	-8.008797	1.276434	1.482619
34	1	0	-8.400892	1.927113	3.422796
35	6	0	-5.848162	-0.649439	1.672159
36	6	0	-3.418738	7.725608	-1.211272
37	1	0	-5.547639	9.930463	-3.754818
38	6	0	-5.4909	8.243654	-2.567963
39	1	0	-1.920067	9.131424	-1.413254
40	6	0	7.258318	0.92238	-1.578284
41	6	0	6.219573	-3.359064	-1.615128
42	6	0	4.570725	-3.121101	0.413606
43	1	0	4.615788	-1.426204	1.569709
44	6	0	2.739174	-4.970706	0.893361
45	6	0	0.841851	-4.62339	3.011261
46	1	0	1.689434	-3.532899	4.567684
47	1	0	0.363599	-6.497063	3.751926
48	6	0	-1.667671	-3.297198	2.338202
49	6	0	-1.325769	-0.453685	1.762765
50	6	0	-3.606273	-5.82888	5.933377
51	1	0	-3.576487	-7.61122	4.85543
52	1	0	-5.366355	-5.721637	7.020688
53	1	0	-2.000124	-5.789257	7.250071
54	6	0	-5.768335	-2.225187	4.073522
55	6	0	2.71186	-7.137647	-0.637928

56	1	0	4.385515	-9.086121	-3.845824
57	6	0	4.415026	-7.411995	-2.640654
58	1	0	1.332486	-8.632583	-0.282531
59	6	0	6.170082	-5.510966	-3.175375
60	6	0	4.943062	1.899055	-2.311868
61	8	0	-7.476612	-2.27368	5.601422

**3a**000008_en_		Standard Orientation (A.U.)			
Center number	Atomic number	Atomic Type	X	Y	Z
0	16	0	-2.610527	-4.633235	-0.875376
1	16	0	-6.417864	-3.353889	-1.16118
2	7	0	-3.961054	-3.914397	4.22654
3	8	0	11.187463	1.225499	-1.479025
4	8	0	-0.261997	4.395343	-1.100734
5	8	0	-10.54716	-0.078432	0.528308
6	1	0	-11.128741	-1.153884	1.896132
7	8	0	1.311345	7.66816	1.113661
8	8	0	7.088105	-1.339287	-2.690615
9	1	0	2.731775	1.179664	-2.240162
10	8	0	0.329652	0.601436	2.381031
11	8	0	7.0963	-5.68205	-5.201817
12	1	0	7.961045	-4.075606	-5.403808
13	7	0	-3.877388	0.54819	1.294445
14	6	0	13.455379	2.458593	-0.802789
15	1	0	13.775768	4.167916	-1.95709
16	1	0	14.987354	1.10482	-1.155541
17	1	0	13.467828	2.993687	1.214708
18	6	0	8.979881	2.465937	-1.213291
19	6	0	8.726312	4.9479	-0.306058
20	1	0	6.121679	7.901786	0.804803
21	6	0	6.336155	6.01786	-0.005694
22	1	0	10.394392	6.027878	0.23312
23	6	0	4.16584	4.675204	-0.698718
24	6	0	1.648425	5.771422	-0.122226
25	6	0	-2.795072	5.004373	-0.298645
26	1	0	-2.72359	5.584045	1.697883
27	6	0	-4.324894	2.57538	-0.603452
28	1	0	-3.817651	1.788735	-2.470921
29	6	0	-7.166982	2.890219	-0.458639
30	8	0	-8.030295	6.291417	-3.3987
31	6	0	-8.679464	4.408712	-1.790943
32	1	0	-10.728131	4.186572	-1.682596
33	6	0	-8.251515	0.94532	1.330281
34	1	0	-8.435867	1.771973	3.245949
35	6	0	-6.094499	-0.962907	1.500254
36	6	0	-3.694758	7.146899	-1.932808
37	1	0	-5.927815	9.242293	-4.470454
38	6	0	-5.804649	7.566501	-3.270863

39	1	0	-2.268518	8.624999	-2.136413
40	6	0	6.775165	1.116058	-1.892602
41	6	0	5.565988	-3.226548	-1.725781
42	6	0	4.080314	-3.005001	0.423716
43	1	0	4.043694	-1.249805	1.485702
44	6	0	2.495975	-4.995711	1.167607
45	6	0	0.619069	-4.646517	3.300996
46	1	0	1.413899	-3.455684	4.809958
47	1	0	0.201286	-6.506245	4.108091
48	6	0	-1.900913	-3.421143	2.495629
49	6	0	-1.634271	-0.531586	2.064999
50	6	0	-4.010721	-6.112391	5.886406
51	1	0	-3.78214	-7.866183	4.786158
52	1	0	-5.865997	-6.128377	6.808146
53	1	0	-2.530038	-6.005176	7.338519
54	6	0	-6.186995	-2.643157	3.811012
55	6	0	2.577597	-7.263805	-0.202468
56	1	0	4.202897	-9.274638	-3.398898
57	6	0	4.13847	-7.517856	-2.319836
58	1	0	1.379489	-8.847997	0.360669
59	6	0	5.62154	-5.491413	-3.128153
60	6	0	4.412921	2.239513	-1.715753
61	8	0	-8.124861	-2.904186	5.035361

**3a**000010_en_		Standard Orientation (A.U.)			
Center number	Atomic number	Atomic Type	X	Y	Z
0	16	0	-2.384797	-4.286682	-1.399983
1	16	0	-6.161882	-2.891245	-1.533223
2	7	0	-3.64768	-4.006873	3.75411
3	8	0	10.927284	1.962535	1.643737
4	8	0	-0.211653	5.592521	0.774652
5	8	0	-10.301478	0.087606	0.528015
6	1	0	-10.850497	-1.138424	1.777231
7	8	0	0.159757	4.412365	-3.315126
8	8	0	8.030585	-0.867461	-2.120045
9	1	0	3.90228	1.440962	-3.43476
10	8	0	0.668592	0.612157	1.932627
11	8	0	8.226239	-4.938087	-5.153828
12	1	0	9.074464	-3.311803	-5.144114
13	7	0	-3.62842	0.745438	1.339831
14	6	0	12.558281	0.353576	0.248453
15	1	0	11.946871	-1.632092	0.323303
16	1	0	14.428421	0.535837	1.132897
17	1	0	12.679728	0.943371	-1.745986
18	6	0	8.65955	2.687879	0.734006
19	6	0	7.545421	4.798544	1.922441
20	1	0	4.22676	7.118513	2.369297
21	6	0	5.123562	5.59837	1.304262

22	1	0	8.623362	5.704669	3.430001
23	6	0	3.81835	4.430935	-0.687786
24	6	0	1.109433	4.855541	-1.281435
25	6	0	-2.93174	5.582821	0.675986
26	1	0	-3.459459	5.884021	2.664816
27	6	0	-4.117191	3.05775	-0.208178
28	1	0	-3.424751	2.697034	-2.132596
29	6	0	-6.97478	3.188412	-0.180613
30	8	0	-7.903497	6.811793	-2.815295
31	6	0	-8.518195	4.754853	-1.420458
32	1	0	-10.555303	4.428092	-1.409734
33	6	0	-8.017744	1.065142	1.412737
34	1	0	-8.200575	1.69882	3.401879
35	6	0	-5.831381	-0.8097	1.377972
36	6	0	-3.754295	7.766458	-0.953962
37	1	0	-5.851946	9.894493	-3.581958
38	6	0	-5.759225	8.172669	-2.447866
39	1	0	-2.360252	9.289434	-0.994626
40	6	0	7.284913	1.476505	-1.197442
41	6	0	6.377511	-2.839304	-1.596158
42	6	0	4.634704	-2.785346	0.364754
43	1	0	4.52472	-1.149737	1.597773
44	6	0	2.902669	-4.763515	0.680506
45	6	0	0.900514	-4.639674	2.726331
46	1	0	1.634081	-3.6365	4.395085
47	1	0	0.468652	-6.580814	3.300858
48	6	0	-1.623044	-3.352107	2.028332
49	6	0	-1.356087	-0.444248	1.787565
50	6	0	-3.662669	-6.349341	5.206286
51	1	0	-2.15815	-6.369849	6.637032
52	1	0	-3.450242	-7.994473	3.946806
53	1	0	-5.501926	-6.456509	6.153511
54	6	0	-5.886086	-2.720119	3.499643
55	6	0	3.064911	-6.852499	-0.946072
56	1	0	4.980231	-8.548414	-4.163626
57	6	0	4.862089	-6.93583	-2.882703
58	1	0	1.758134	-8.435272	-0.720447
59	6	0	6.521878	-4.915084	-3.250555
60	6	0	4.9643	2.43972	-1.977873
61	8	0	-7.804572	-3.11675	4.718945

**3a**000011_en_		Standard Orientation (A.U.)			
Center number	Atomic number	Atomic Type	X	Y	Z
0	16	0	-4.202818	-4.081913	-3.807562
1	16	0	-7.385111	-2.290746	-2.092166
2	7	0	-2.848498	-4.630874	1.224829
3	8	0	11.067278	1.376375	-1.670728
4	8	0	0.142014	4.605634	1.48949

5	8	0	-9.981922	0.05086	1.991472
6	1	0	-10.203777	-1.471633	2.988217
7	8	0	0.307158	6.78022	-2.195186
8	8	0	7.899549	-0.502188	1.782993
9	1	0	3.647071	1.994576	2.370968
10	8	0	0.113241	0.416577	-2.207123
11	8	0	9.437607	-4.730487	4.056064
12	1	0	10.094518	-3.020262	3.950005
13	7	0	-3.483523	0.449539	0.178803
14	6	0	12.755688	2.178522	-3.576454
15	1	0	13.383354	4.145217	-3.269236
16	1	0	11.898806	2.029736	-5.473575
17	1	0	14.399748	0.917684	-3.472164
18	6	0	8.84854	2.592037	-1.392343
19	6	0	8.037772	4.650639	-2.864857
20	1	0	4.920656	7.130259	-3.793966
21	6	0	5.616199	5.643709	-2.544927
22	1	0	9.257503	5.415517	-4.337093
23	6	0	4.014087	4.705661	-0.662373
24	6	0	1.328645	5.514302	-0.595354
25	6	0	-2.528703	4.896195	1.802569
26	1	0	-2.811197	4.221295	3.75046
27	6	0	-4.111205	3.171589	0.049042
28	1	0	-3.902169	3.845489	-1.907668
29	6	0	-6.80806	3.220482	0.979813
30	8	0	-7.8326	7.592596	0.318525
31	6	0	-8.395512	5.180268	0.983772
32	1	0	-10.366115	4.961634	1.558828
33	6	0	-7.434439	0.708853	2.170908
34	1	0	-6.813418	0.727843	4.173446
35	6	0	-5.631688	-1.078678	0.795698
36	6	0	-3.335381	7.613034	1.535247
37	1	0	-5.670122	10.697751	0.576596
38	6	0	-5.528695	8.651705	0.804871
39	1	0	-1.835689	8.981663	1.899244
40	6	0	7.215593	1.66167	0.502326
41	6	0	6.459951	-2.6464	1.360715
42	6	0	4.322209	-2.731157	-0.157922
43	1	0	3.694563	-1.063644	-1.171556
44	6	0	2.921473	-4.967618	-0.415152
45	6	0	0.583778	-4.982958	-2.081038
46	1	0	0.060717	-6.941055	-2.52946
47	1	0	1.026847	-4.048494	-3.883735
48	6	0	-1.810103	-3.628948	-1.099417
49	6	0	-1.54174	-0.708146	-1.082134
50	6	0	-2.445996	-7.263605	1.944533
51	1	0	-3.770606	-7.682036	3.480192
52	1	0	-0.507476	-7.566181	2.60184
53	1	0	-2.853419	-8.519432	0.334278

54	6	0	-4.916385	-3.430403	2.237955
55	6	0	3.819687	-7.148016	0.790607
56	1	0	6.71285	-8.781103	3.212904
57	6	0	6.003952	-7.081464	2.28275
58	1	0	2.837647	-8.947676	0.547675
59	6	0	7.335229	-4.827028	2.610108
60	6	0	4.884506	2.773608	0.925328
61	8	0	-6.161559	-4.179316	4.028336

**3a**000012_en_		Standard Orientation (A.U.)			
Center number	Atomic number	Atomic Type	X	Y	Z
0	16	0	-1.819732	-4.637145	-0.832305
1	16	0	-5.713975	-3.872687	-1.332912
2	7	0	-3.613041	-3.767603	4.132773
3	8	0	11.476598	2.299085	-0.787485
4	8	0	-0.263491	4.61707	-1.24648
5	8	0	-10.149261	-0.819883	-0.731793
6	1	0	-9.689868	-0.938498	-2.501497
7	8	0	1.041376	7.934119	1.075639
8	8	0	7.325798	-0.744416	-2.574895
9	1	0	2.962825	1.616454	-2.350537
10	8	0	0.34869	1.004606	2.33267
11	8	0	8.145146	-5.27943	-4.453234
12	1	0	8.245673	-7.013641	-5.016142
13	7	0	-3.745499	0.492756	0.955589
14	6	0	12.139978	-0.306407	-0.702223
15	1	0	11.81222	-1.263379	-2.515112
16	1	0	11.077749	-1.305471	0.787468
17	1	0	14.162675	-0.347859	-0.23377
18	6	0	9.056733	3.088897	-0.827732
19	6	0	8.624056	5.566205	0.053
20	1	0	5.865443	8.424249	1.004349
21	6	0	6.202733	6.558815	0.193078
22	1	0	10.264781	6.632318	0.705999
23	6	0	4.127221	5.132643	-0.650952
24	6	0	1.53686	6.083025	-0.176331
25	6	0	-2.83628	5.03631	-0.463053
26	1	0	-2.834231	5.503748	1.56448
27	6	0	-4.220911	2.533457	-0.913492
28	1	0	-3.56512	1.838479	-2.772672
29	6	0	-7.073184	2.666598	-0.896839
30	8	0	-8.139827	6.355245	-3.436835
31	6	0	-8.64769	4.259584	-2.07581
32	1	0	-10.681698	3.902175	-2.026225
33	6	0	-8.183562	0.43004	0.530884
34	1	0	-8.973275	0.990557	2.373395
35	6	0	-5.841287	-1.200321	1.088541
36	6	0	-3.814863	7.233576	-1.97489

37	1	0	-6.151672	9.447895	-4.313847
38	6	0	-5.953058	7.69287	-3.24647
39	1	0	-2.436076	8.764277	-2.089222
40	6	0	6.962642	1.682879	-1.700151
41	6	0	6.062842	-2.706315	-1.447077
42	6	0	4.446455	-2.441714	0.607997
43	1	0	4.133882	-0.594841	1.443466
44	6	0	3.074208	-4.504636	1.55392
45	6	0	1.064818	-4.123543	3.558192
46	1	0	1.682358	-2.739266	4.982132
47	1	0	0.754938	-5.932038	4.514429
48	6	0	-1.497032	-3.194833	2.521127
49	6	0	-1.474843	-0.325104	1.939916
50	6	0	-3.590965	-5.843786	5.940718
51	1	0	-5.494872	-5.95372	6.7499
52	1	0	-2.227749	-5.516728	7.474124
53	1	0	-3.148733	-7.642841	4.987702
54	6	0	-5.91608	-2.66402	3.566027
55	6	0	3.487074	-6.891972	0.493454
56	1	0	5.520483	-9.064702	-2.315153
57	6	0	5.197425	-7.190832	-1.501767
58	1	0	2.451785	-8.534357	1.193839
59	6	0	6.483053	-5.118909	-2.510052
60	6	0	4.549676	2.736527	-1.675133
61	8	0	-7.823556	-2.916807	4.811074

**3a**000013_en_		Standard Orientation (A.U.)			
Center number	Atomic number	Atomic Type	X	Y	Z
0	16	0	-2.17883	-4.40685	-1.352804
1	16	0	-5.984164	-3.211161	-1.617789
2	7	0	-3.736727	-3.86466	3.738054
3	8	0	11.009083	1.78873	1.964008
4	8	0	-0.041928	5.527329	0.697921
5	8	0	-10.170708	0.053192	-0.469276
6	1	0	-9.799946	-0.028772	-2.262308
7	8	0	0.454063	4.380316	-3.387625
8	8	0	8.165856	-1.075209	-1.813587
9	1	0	4.13446	1.282232	-3.314718
10	8	0	0.654635	0.631365	1.898777
11	8	0	8.441764	-5.301691	-4.613837
12	1	0	9.294103	-3.678574	-4.667021
13	7	0	-3.605914	0.726115	1.074271
14	6	0	12.645906	0.111014	0.659203
15	1	0	12.842376	0.649476	-1.343758
16	1	0	11.988025	-1.858428	0.762077
17	1	0	14.491061	0.274479	1.597946
18	6	0	8.783008	2.533896	0.973041
19	6	0	7.672015	4.687889	2.083835

20	1	0	4.383718	7.071988	2.384744
21	6	0	5.284019	5.515413	1.376505
22	1	0	8.719673	5.606819	3.60496
23	6	0	4.019957	4.332038	-0.632603
24	6	0	1.33936	4.792995	-1.318125
25	6	0	-2.754754	5.551337	0.509243
26	1	0	-3.350687	5.811638	2.484594
27	6	0	-3.957118	3.057444	-0.471622
28	1	0	-3.165404	2.707887	-2.360364
29	6	0	-6.801078	3.250962	-0.586724
30	8	0	-7.608797	7.063735	-3.025961
31	6	0	-8.267898	4.939049	-1.772007
32	1	0	-10.31364	4.661395	-1.822106
33	6	0	-8.040924	1.034295	0.754177
34	1	0	-8.681602	1.543773	2.670019
35	6	0	-5.827549	-0.812215	1.081808
36	6	0	-3.463971	7.802301	-1.082535
37	1	0	-5.443201	10.100532	-3.663135
38	6	0	-5.424777	8.339178	-2.588699
39	1	0	-2.011808	9.269823	-1.051835
40	6	0	7.444051	1.305655	-0.972981
41	6	0	6.484674	-3.008736	-1.237426
42	6	0	4.676908	-2.844819	0.657781
43	1	0	4.527743	-1.144654	1.795604
44	6	0	2.932857	-4.804074	1.027275
45	6	0	0.854429	-4.559137	2.984306
46	1	0	1.520729	-3.452417	4.614914
47	1	0	0.394654	-6.460766	3.660501
48	6	0	-1.634085	-3.319824	2.097714
49	6	0	-1.360316	-0.437321	1.704511
50	6	0	-3.838253	-6.110305	5.330557
51	1	0	-5.71715	-6.141808	6.202474
52	1	0	-2.401719	-6.057177	6.829972
53	1	0	-3.586306	-7.832544	4.186305
54	6	0	-5.950773	-2.517183	3.392141
55	6	0	3.148718	-6.982676	-0.47059
56	1	0	5.166515	-8.854757	-3.524062
57	6	0	5.007201	-7.172333	-2.341148
58	1	0	1.835129	-8.552035	-0.197976
59	6	0	6.678475	-5.173762	-2.768778
60	6	0	5.167965	2.294502	-1.846583
61	8	0	-7.826094	-2.730867	4.692964

**3a**000014_en_		Standard Orientation (A.U.)			
Center number	Atomic number	Atomic Type	X	Y	Z
0	16	0	-4.165053	-5.718658	-0.941327
1	16	0	-7.298841	-3.128791	-1.054964
2	7	0	-3.584002	-3.656944	3.777668

3	8	0	11.700392	1.331957	0.900253
4	8	0	0.24802	4.170796	-0.485043
5	8	0	-10.318476	0.905503	1.010719
6	1	0	-10.842711	0.021786	2.52854
7	8	0	1.345915	7.537957	1.878106
8	8	0	8.016286	-1.266796	-1.113248
9	1	0	3.462532	1.073218	-1.203687
10	8	0	0.042763	-1.011913	-1.393902
11	8	0	7.917814	-3.941557	-5.431703
12	1	0	9.00573	-2.62023	-4.767618
13	7	0	-3.606914	0.30967	0.449303
14	6	0	13.782116	2.595954	1.986723
15	1	0	14.186832	4.395782	1.00946
16	1	0	15.412472	1.329875	1.778585
17	1	0	13.474653	2.98491	4.0146
18	6	0	9.421422	2.470815	0.90742
19	6	0	8.927597	4.873131	1.916922
20	1	0	6.081897	7.713443	2.692553
21	6	0	6.487808	5.872005	1.858176
22	1	0	10.445664	5.96083	2.783911
23	6	0	4.51737	4.514969	0.734392
24	6	0	1.928931	5.606907	0.796723
25	6	0	-2.361538	4.88316	-0.40382
26	1	0	-2.808457	5.454415	1.548223
27	6	0	-3.966827	2.568407	-1.141722
28	1	0	-3.518187	2.079315	-3.115352
29	6	0	-6.730305	3.162165	-0.760674
30	8	0	-7.237091	6.603148	-3.691511
31	6	0	-8.096053	4.901647	-1.972667
32	1	0	-10.131107	5.06706	-1.673938
33	6	0	-7.774434	1.521319	1.327482
34	1	0	-7.457957	2.463246	3.172916
35	6	0	-5.965599	-0.737229	1.277428
36	6	0	-2.82418	7.003951	-2.245034
37	1	0	-4.752118	9.13381	-5.009492
38	6	0	-4.851228	7.577361	-3.657544
39	1	0	-1.199597	8.239164	-2.543902
40	6	0	7.409338	1.091556	-0.196482
41	6	0	6.149436	-3.079667	-1.359411
42	6	0	4.361205	-3.58222	0.49076
43	1	0	4.389353	-2.481853	2.235093
44	6	0	2.412384	-5.311974	0.035869
45	6	0	0.292235	-5.500226	1.929558
46	1	0	1.008893	-4.843625	3.765485
47	1	0	-0.288111	-7.480757	2.189696
48	6	0	-2.14141	-3.933451	1.439529
49	6	0	-1.681535	-1.386643	0.055066
50	6	0	-3.482032	-5.605485	5.729739
51	1	0	-1.649531	-5.623902	6.70143

52	1	0	-3.847829	-7.481356	4.902535
53	1	0	-4.972504	-5.16917	7.099409
54	6	0	-5.69923	-2.150299	3.733014
55	6	0	2.420493	-6.668376	-2.234738
56	1	0	4.302225	-7.297116	-5.83172
57	6	0	4.288245	-6.254843	-4.051653
58	1	0	0.920902	-8.033201	-2.626099
59	6	0	6.149075	-4.41938	-3.654683
60	6	0	5.001528	2.128808	-0.33525
61	8	0	-7.269536	-1.987728	5.412043

**3a**000015_en_		Standard Orientation (A.U.)			
Center number	Atomic number	Atomic Type	X	Y	Z
0	16	0	-5.2659	-5.98762	0.355889
1	16	0	-7.572466	-3.264824	2.120766
2	7	0	-1.838682	-3.497294	3.554896
3	8	0	11.012606	3.054421	1.68969
4	8	0	-0.311139	3.563762	-2.315575
5	8	0	-8.95846	1.605199	3.829989
6	1	0	-10.16812	0.890391	2.653575
7	8	0	0.612782	7.705155	-1.974097
8	8	0	7.354946	-0.881259	1.103667
9	1	0	3.072074	0.75439	-1.052141
10	8	0	-1.180087	-2.174781	-2.966586
11	8	0	9.574153	-3.052385	-2.945967
12	1	0	10.096126	-3.903452	-4.47481
13	7	0	-3.673567	-0.225344	-0.020639
14	6	0	12.273584	0.678186	1.58448
15	1	0	11.590108	-0.612181	3.063299
16	1	0	14.281543	1.102901	1.906396
17	1	0	12.027172	-0.239234	-0.262252
18	6	0	8.693926	3.429302	0.710512
19	6	0	8.085344	5.950877	0.124425
20	1	0	5.238966	8.578969	-1.17967
21	6	0	5.723687	6.611782	-0.799862
22	1	0	9.547386	7.367532	0.45719
23	6	0	3.901508	4.731003	-1.228147
24	6	0	1.312014	5.527487	-1.911073
25	6	0	-2.906273	4.202495	-1.969253
26	1	0	-2.997106	5.33358	-0.225479
27	6	0	-4.612223	1.826701	-1.613193
28	1	0	-5.077349	1.056837	-3.484248
29	6	0	-6.84051	2.784941	-0.138386
30	8	0	-8.571514	5.646902	-3.226524
31	6	0	-8.516888	4.502446	-0.929711
32	1	0	-10.068729	5.138735	0.275546
33	6	0	-6.652488	1.848141	2.569419
34	1	0	-5.49396	3.140765	3.728811

35	6	0	-5.102399	-0.609396	2.240353
36	6	0	-4.01058	5.654209	-4.156336
37	1	0	-7.013576	7.120599	-6.359704
38	6	0	-6.453087	6.123694	-4.64325
39	1	0	-2.671416	6.332194	-5.567669
40	6	0	6.829958	1.550825	0.311826
41	6	0	6.131244	-2.834606	-0.094212
42	6	0	3.769229	-3.647093	0.738794
43	1	0	2.933632	-2.660594	2.344383
44	6	0	2.464906	-5.556419	-0.544899
45	6	0	-0.171073	-6.40444	0.159379
46	1	0	-0.131804	-7.883036	1.620468
47	1	0	-0.990128	-7.292842	-1.530592
48	6	0	-2.057384	-4.366922	0.985026
49	6	0	-2.176595	-2.154046	-0.906824
50	6	0	-0.765375	-5.120392	5.507147
51	1	0	-1.835102	-6.904454	5.64139
52	1	0	-0.921867	-4.100047	7.302828
53	1	0	1.233715	-5.537025	5.133175
54	6	0	-3.367793	-1.499194	4.346723
55	6	0	3.641881	-6.684569	-2.629884
56	1	0	6.907256	-6.821388	-5.08003
57	6	0	6.030935	-5.923358	-3.435945
58	1	0	2.654021	-8.161433	-3.682129
59	6	0	7.291198	-3.955914	-2.202846
60	6	0	4.490973	2.203128	-0.70916
61	8	0	-3.375835	-0.63395	6.465081

**3a**000016_en_		Standard Orientation (A.U.)			
Center number	Atomic number	Atomic Type	X	Y	Z
0	16	0	-4.183941	-5.763937	-0.515226
1	16	0	-7.435723	-3.368808	-0.425207
2	7	0	-3.474962	-3.336689	4.06017
3	8	0	11.710995	1.17816	0.606758
4	8	0	0.264002	4.079595	-0.582709
5	8	0	-10.415554	0.904252	0.806484
6	1	0	-10.593824	0.529856	-0.978099
7	8	0	1.426399	7.498151	1.675192
8	8	0	7.955459	-1.394963	-1.341195
9	1	0	3.440146	0.989459	-1.365574
10	8	0	-0.027306	-1.073458	-1.36627
11	8	0	7.660082	-4.311615	-5.497695
12	1	0	8.778689	-2.958833	-4.960871
13	7	0	-3.683818	0.312674	0.410964
14	6	0	13.817681	2.418292	1.673112
15	1	0	14.23925	4.20875	0.686002
16	1	0	15.429072	1.129433	1.4576
17	1	0	13.530155	2.818435	3.701709

18	6	0	9.445849	2.342469	0.63449
19	6	0	8.991102	4.756979	1.636399
20	1	0	6.186328	7.628373	2.44377
21	6	0	6.560792	5.778904	1.612299
22	1	0	10.532895	5.833796	2.474648
23	6	0	4.558715	4.433002	0.530344
24	6	0	1.980061	5.539997	0.629098
25	6	0	-2.326234	4.841678	-0.512359
26	1	0	-2.765864	5.456773	1.427625
27	6	0	-3.997179	2.546853	-1.208027
28	1	0	-3.574835	2.025168	-3.179575
29	6	0	-6.728665	3.223909	-0.792845
30	8	0	-7.20329	6.738933	-3.668134
31	6	0	-8.04986	5.045121	-1.9457
32	1	0	-10.064637	5.298598	-1.567706
33	6	0	-7.895604	1.580322	1.249784
34	1	0	-7.881789	2.527729	3.105946
35	6	0	-6.005591	-0.635883	1.442989
36	6	0	-2.733057	6.940384	-2.393276
37	1	0	-4.64884	9.119341	-5.133524
38	6	0	-4.771278	7.595548	-3.747701
39	1	0	-1.054536	8.074901	-2.779138
40	6	0	7.403617	0.979917	-0.431959
41	6	0	6.072131	-3.206885	-1.408834
42	6	0	4.353104	-3.581008	0.534314
43	1	0	4.457202	-2.382326	2.208869
44	6	0	2.370308	-5.307671	0.257724
45	6	0	0.322842	-5.33892	2.23759
46	1	0	1.110652	-4.546401	3.988451
47	1	0	-0.254217	-7.289296	2.672091
48	6	0	-2.126172	-3.805658	1.717728
49	6	0	-1.732356	-1.377172	0.123943
50	6	0	-3.299806	-5.110967	6.1665
51	1	0	-3.697693	-7.049015	5.516274
52	1	0	-4.732899	-4.546561	7.550355
53	1	0	-1.433958	-5.059032	7.073661
54	6	0	-5.536301	-1.726918	4.047355
55	6	0	2.280437	-6.800006	-1.924265
56	1	0	4.013909	-7.658625	-5.547967
57	6	0	4.078909	-6.512967	-3.833706
58	1	0	0.75804	-8.173028	-2.174259
59	6	0	5.966837	-4.674344	-3.621541
60	6	0	5.004322	2.039728	-0.536357
61	8	0	-6.854457	-1.27336	5.867259

**3a**000017_en_		Standard Orientation (A.U.)			
Center number	Atomic number	Atomic Type	X	Y	Z
0	16	0	-2.499088	-4.407191	-1.057465

1	16	0	-6.30562	-3.198187	-0.992993
2	7	0	-3.601413	-3.793473	4.145087
3	8	0	10.947434	1.761867	1.383394
4	8	0	-0.13418	5.546522	0.759424
5	8	0	-10.345615	0.124468	0.463516
6	1	0	-10.133642	-0.002814	-1.352655
7	8	0	0.097506	4.356667	-3.336722
8	8	0	7.896401	-1.084621	-2.23798
9	1	0	3.785527	1.28037	-3.500128
10	8	0	0.650714	0.63444	1.871759
11	8	0	7.906997	-5.265706	-5.130711
12	1	0	8.782699	-3.656275	-5.21385
13	7	0	-3.664835	0.760412	1.41723
14	6	0	12.512694	0.09685	-0.021752
15	1	0	11.868277	-1.875409	0.109135
16	1	0	14.408513	0.262727	0.809623
17	1	0	12.591238	0.645559	-2.029964
18	6	0	8.675603	2.520461	0.51346
19	6	0	7.637323	4.679	1.683953
20	1	0	4.376958	7.072032	2.170701
21	6	0	5.214936	5.51276	1.114165
22	1	0	8.773133	5.594347	3.142634
23	6	0	3.833783	4.329671	-0.816349
24	6	0	1.115432	4.787852	-1.333469
25	6	0	-2.852275	5.580024	0.736359
26	1	0	-3.324746	5.87925	2.739261
27	6	0	-4.126058	3.075139	-0.123686
28	1	0	-3.478972	2.704761	-2.062831
29	6	0	-6.969453	3.281357	-0.01929
30	8	0	-7.937605	7.045498	-2.471893
31	6	0	-8.514243	4.956016	-1.121451
32	1	0	-10.559717	4.697712	-1.00302
33	6	0	-8.107606	1.107505	1.472693
34	1	0	-8.570167	1.664281	3.426412
35	6	0	-5.890558	-0.755197	1.642056
36	6	0	-3.657157	7.799792	-0.854607
37	1	0	-5.805198	10.047556	-3.343824
38	6	0	-5.718979	8.31413	-2.228079
39	1	0	-2.197506	9.2557	-0.967436
40	6	0	7.226924	1.297413	-1.355629
41	6	0	6.234309	-3.009249	-1.580821
42	6	0	4.56889	-2.854934	0.440997
43	1	0	4.530096	-1.171962	1.613324
44	6	0	2.821866	-4.791936	0.898539
45	6	0	0.895429	-4.548365	3.005316
46	1	0	1.690064	-3.458095	4.589125
47	1	0	0.473309	-6.451168	3.703178
48	6	0	-1.645591	-3.289742	2.320971
49	6	0	-1.382653	-0.416339	1.86763

50	6	0	-3.587773	-6.025038	5.760347
51	1	0	-2.02357	-5.979867	7.126459
52	1	0	-3.459353	-7.758974	4.613092
53	1	0	-5.381901	-6.0258	6.796121
54	6	0	-5.823277	-2.423163	3.981201
55	6	0	2.895577	-6.945008	-0.649444
56	1	0	4.659354	-8.788801	-3.8721
57	6	0	4.612907	-7.126824	-2.651
58	1	0	1.580222	-8.500279	-0.311901
59	6	0	6.282838	-5.146183	-3.161708
60	6	0	4.905725	2.290428	-2.095552
61	8	0	-7.573409	-2.593171	5.451603

**3a**000018_en_		Standard Orientation (A.U.)			
Center number	Atomic number	Atomic Type	X	Y	Z
0	16	0	-2.348731	-4.641781	-0.468941
1	16	0	-6.166473	-3.582839	-1.005834
2	7	0	-4.085421	-3.456832	4.459262
3	8	0	11.390813	0.963431	-0.966982
4	8	0	-0.001898	4.37859	-1.244779
5	8	0	-10.339997	-0.137964	-0.522382
6	1	0	-9.888895	-0.415748	-2.27649
7	8	0	1.566005	7.67857	0.933968
8	8	0	7.272196	-1.609385	-2.124182
9	1	0	2.971698	1.020924	-2.052117
10	8	0	0.277696	0.893839	2.50369
11	8	0	7.446074	-6.226975	-4.057297
12	1	0	8.311147	-4.647279	-4.411148
13	7	0	-3.842063	0.680075	1.132836
14	6	0	13.659444	2.199354	-0.297457
15	1	0	13.61179	2.878241	1.675639
16	1	0	14.056929	3.813957	-1.559077
17	1	0	15.17321	0.794671	-0.495967
18	6	0	9.202507	2.260893	-0.875306
19	6	0	8.968331	4.805629	-0.154654
20	1	0	6.38587	7.868345	0.672573
21	6	0	6.59067	5.933085	-0.009308
22	1	0	10.637719	5.890779	0.369971
23	6	0	4.419018	4.585551	-0.69048
24	6	0	1.905743	5.743054	-0.239255
25	6	0	-2.537048	5.050591	-0.501857
26	1	0	-2.498824	5.613779	1.500468
27	6	0	-4.126765	2.655433	-0.84106
28	1	0	-3.524079	1.811754	-2.656183
29	6	0	-6.956734	3.035239	-0.862478
30	8	0	-7.676711	6.646864	-3.622382
31	6	0	-8.378764	4.683422	-2.152462
32	1	0	-10.436253	4.504865	-2.112439

33	6	0	-8.263075	0.985004	0.676203
34	1	0	-8.988687	1.709972	2.487781
35	6	0	-6.076069	-0.820387	1.315088
36	6	0	-3.315411	7.245471	-2.128347
37	1	0	-5.431381	9.520905	-4.613182
38	6	0	-5.392134	7.810391	-3.458959
39	1	0	-1.816824	8.654144	-2.292105
40	6	0	6.995413	0.908079	-1.539373
41	6	0	5.723186	-3.381352	-0.993427
42	6	0	4.130411	-2.915986	1.037383
43	1	0	4.020042	-1.04553	1.873412
44	6	0	2.532835	-4.825285	1.950256
45	6	0	0.545697	-4.242884	3.926653
46	1	0	1.26099	-2.889818	5.335168
47	1	0	0.077595	-6.002266	4.910764
48	6	0	-1.921063	-3.125884	2.845196
49	6	0	-1.650622	-0.294139	2.157755
50	6	0	-4.245469	-5.457662	6.344322
51	1	0	-3.957821	-7.322018	5.460827
52	1	0	-6.152585	-5.371594	7.14865
53	1	0	-2.860351	-5.191852	7.869904
54	6	0	-6.282024	-2.175635	3.845219
55	6	0	2.703603	-7.244418	0.879969
56	1	0	4.50398	-9.612347	-1.958245
57	6	0	4.369408	-7.736164	-1.111722
58	1	0	1.492237	-8.763409	1.577952
59	6	0	5.869908	-5.803154	-2.095751
60	6	0	4.653256	2.085633	-1.538295
61	8	0	-8.204288	-2.209355	5.093334

**3a**000019_en_		Standard Orientation (A.U.)			
Center number	Atomic number	Atomic Type	X	Y	Z
0	16	0	-5.321435	-5.934525	0.349955
1	16	0	-7.755777	-2.986183	1.597585
2	7	0	-2.028052	-3.461171	3.603569
3	8	0	10.904692	3.108263	2.060692
4	8	0	-0.253747	3.64112	-2.387778
5	8	0	-8.852609	1.779373	3.731285
6	1	0	-8.463528	1.371937	5.475207
7	8	0	0.700842	7.77746	-2.071907
8	8	0	7.228482	-0.808491	1.417497
9	1	0	3.054314	0.817832	-0.940965
10	8	0	-1.09267	-2.186482	-2.934774
11	8	0	9.594772	-3.00829	-2.536445
12	1	0	10.168407	-3.864836	-4.043593
13	7	0	-3.584585	-0.127731	-0.058266
14	6	0	12.136315	0.714449	2.089174
15	1	0	11.364491	-0.520569	3.571757

16	1	0	14.131906	1.124623	2.49566
17	1	0	11.966412	-0.255215	0.260771
18	6	0	8.632611	3.478588	0.975532
19	6	0	8.076341	5.990535	0.301774
20	1	0	5.312398	8.614851	-1.174533
21	6	0	5.760026	6.652438	-0.729867
22	1	0	9.540134	7.400005	0.656861
23	6	0	3.932747	4.781848	-1.181206
24	6	0	1.373445	5.593019	-1.962297
25	6	0	-2.854554	4.280636	-2.053383
26	1	0	-2.948144	5.419804	-0.313931
27	6	0	-4.535697	1.89978	-1.689452
28	1	0	-4.97604	1.100366	-3.554064
29	6	0	-6.810313	2.84405	-0.256099
30	8	0	-8.550403	5.567542	-3.443334
31	6	0	-8.528993	4.478672	-1.113147
32	1	0	-10.142189	5.063501	0.032922
33	6	0	-6.557672	1.969436	2.451717
34	1	0	-5.233123	3.246376	3.46158
35	6	0	-5.159882	-0.544001	2.098792
36	6	0	-3.961554	5.717851	-4.248645
37	1	0	-6.94714	7.10896	-6.51515
38	6	0	-6.407917	6.115702	-4.789073
39	1	0	-2.613652	6.464545	-5.616
40	6	0	6.765797	1.610287	0.546279
41	6	0	6.057909	-2.779733	0.196481
42	6	0	3.674156	-3.59741	0.959141
43	1	0	2.779325	-2.593901	2.522128
44	6	0	2.425066	-5.53068	-0.34376
45	6	0	-0.234204	-6.377212	0.271053
46	1	0	-0.247082	-7.842189	1.746003
47	1	0	-0.991832	-7.277865	-1.439624
48	6	0	-2.148234	-4.327126	1.003598
49	6	0	-2.139061	-2.11038	-0.90247
50	6	0	-1.031196	-5.09302	5.588748
51	1	0	-2.087419	-6.888435	5.658582
52	1	0	-1.286168	-4.095578	7.386071
53	1	0	0.986559	-5.483057	5.298739
54	6	0	-3.66539	-1.552329	4.31181
55	6	0	3.677496	-6.675511	-2.374751
56	1	0	7.022643	-6.818049	-4.714336
57	6	0	6.086895	-5.907463	-3.110439
58	1	0	2.732544	-8.169196	-3.442339
59	6	0	7.293462	-3.917837	-1.859804
60	6	0	4.474706	2.261236	-0.579968
61	8	0	-3.983453	-0.797882	6.463405

**3a**000020_en_		Standard Orientation (A.U.)			
Center	Atomic	Atomic	X	Y	Z

number	number	Type			
0	16	0	-5.644999	-5.295426	-2.04818
1	16	0	-8.114016	-3.010159	0.076379
2	7	0	-2.788521	-4.19226	2.293937
3	8	0	10.836502	1.019288	2.044009
4	8	0	-0.046269	4.13209	-1.398097
5	8	0	-9.572016	1.24138	3.002825
6	1	0	-10.606179	0.977828	1.513001
7	8	0	1.364369	8.091921	-0.622745
8	8	0	6.524968	-1.427108	2.499594
9	1	0	2.456727	1.034693	0.759268
10	8	0	-0.911742	-1.132394	-3.37187
11	8	0	9.885671	-4.019704	-0.392202
12	1	0	10.114082	-2.662382	0.836601
13	7	0	-3.778021	0.155804	-0.389316
14	6	0	13.227214	2.209279	1.87904
15	1	0	13.633336	2.833629	-0.069203
16	1	0	14.628269	0.791149	2.450797
17	1	0	13.344593	3.846587	3.164475
18	6	0	8.737021	2.340963	1.399373
19	6	0	8.710543	4.865338	0.587652
20	1	0	6.391197	7.978678	-0.708516
21	6	0	6.436271	6.002963	-0.119016
22	1	0	10.459162	5.949009	0.494229
23	6	0	4.187477	4.611447	-0.101161
24	6	0	1.751856	5.843989	-0.739698
25	6	0	-2.617895	4.882638	-1.112949
26	1	0	-2.823179	5.549668	0.849784
27	6	0	-4.4416	2.599508	-1.514246
28	1	0	-4.696297	2.334967	-3.558612
29	6	0	-6.813265	3.303369	-0.117398
30	8	0	-8.05144	7.009758	-2.470519
31	6	0	-8.312928	5.281573	-0.593646
32	1	0	-9.987028	5.659222	0.554955
33	6	0	-7.087533	1.637213	2.204364
34	1	0	-6.07887	2.450762	3.840823
35	6	0	-5.604706	-0.741468	1.394665
36	6	0	-3.42665	6.887219	-2.969145
37	1	0	-6.100248	9.06454	-4.994632
38	6	0	-5.778266	7.64508	-3.533211
39	1	0	-1.923368	7.78275	-4.053513
40	6	0	6.439474	0.992655	1.542594
41	6	0	5.608624	-3.307854	0.920673
42	6	0	3.039115	-3.835041	0.743996
43	1	0	1.742525	-2.79739	1.960074
44	6	0	2.162329	-5.607858	-1.00529
45	6	0	-0.614913	-6.130708	-1.460297
46	1	0	-1.127605	-8.029003	-0.780959
47	1	0	-0.910254	-6.146906	-3.518921

48	6	0	-2.542501	-4.252298	-0.412545
49	6	0	-2.235732	-1.58932	-1.566956
50	6	0	-2.141756	-6.40225	3.81125
51	1	0	-0.128412	-6.872537	3.632949
52	1	0	-3.296671	-8.038187	3.234161
53	1	0	-2.564706	-5.934313	5.783927
54	6	0	-4.328291	-2.360971	3.388791
55	6	0	3.958244	-6.904104	-2.46846
56	1	0	7.918184	-7.439482	-3.397446
57	6	0	6.540239	-6.433991	-2.236807
58	1	0	3.318289	-8.297751	-3.853909
59	6	0	7.407227	-4.580091	-0.556937
60	6	0	4.2107	2.097852	0.72901
61	8	0	-4.675306	-2.128336	5.639807

Table S11. Gibbs free energies[a] and equilibrium populations[b] of low-energy conformers of 3b.

Conformers	ΔG(a.u.)	P(%)/100	G(a.u.)
**3b**000001.out	0.0	14.83	-2624.773828
**3b**000002.out	0.00412	0.19	-2624.76971
**3b**000003.out	0.00418	0.18	-2624.769652
**3b**000004.out	0.00253	1.01	-2624.771295
**3b**000005.out	0.00587	0.03	-2624.767963
**3b**000006.out	0.00061	7.77	-2624.773217
**3b**000007.out	0.0	14.83	-2624.773828
**3b**000008.out	0.0	14.79	-2624.773825
**3b**000009.out	0.00412	0.19	-2624.76971
**3b**000010.out	0.00014	12.74	-2624.773685
**3b**000011.out	0.00418	0.18	-2624.769652
**3b**000012.out	0.00048	8.95	-2624.773352
**3b**000013.out	0.00253	1.01	-2624.771295
**3b**000014.out	0.00254	1.01	-2624.77129
**3b**000015.out	0.00587	0.03	-2624.767963
**3b**000016.out	8e-05	13.63	-2624.773748
**3b**000017.out	0.00061	7.77	-2624.773217
**3b**000018.out	0.00268	0.86	-2624.771145

[a]wB97M-V/def2-TZVP, in a.u.

[b]From ΔG values at 298.15 K.

Table S12. Cartesian coordinates for the low-energy reoptimized random research conformers of 3b at B3LYP-D3(BJ)/6-31G* level of theory in chloroform.

**3b**000001_en_		Standard Orientation (A.U.)			
Center number	Atomic number	Atomic Type	X	Y	Z
0	16	0	12.870554	14.695797	41.053954
1	16	0	11.592338	18.222469	39.561365
2	7	0	7.609671	14.332535	41.65239
3	8	0	15.958638	1.057572	35.416944
4	8	0	13.338376	12.399038	33.43448
5	8	0	4.892061	18.44992	35.856073
6	1	0	4.202175	18.734223	37.543043
7	8	0	10.911045	10.798711	30.29791
8	8	0	16.773244	5.18656	38.174879
9	1	0	15.355745	9.501502	36.040842
10	8	0	9.647333	10.364425	36.87649
11	8	0	20.030618	6.377022	41.858708
12	1	0	20.141865	5.375814	40.324087
13	7	0	9.183612	14.668151	36.589239
14	6	0	15.459192	-1.214845	34.111379
15	1	0	13.409907	-1.527101	33.866814
16	1	0	16.245396	-2.743573	35.272894
17	1	0	16.375351	-1.242984	32.235964
18	6	0	15.166411	3.259613	34.410753
19	6	0	13.866516	3.505082	32.105713
20	1	0	11.895806	6.044016	29.56869
21	6	0	13.017768	5.865262	31.289678
22	1	0	13.44413	1.834268	30.978871
23	6	0	13.559224	8.035742	32.698546
24	6	0	12.443549	10.498815	31.966684
25	6	0	12.410476	14.918744	33.077493
26	1	0	13.534557	16.016578	34.439447
27	6	0	9.604678	15.202032	33.875298
28	1	0	8.455255	13.911919	32.721642
29	6	0	8.783038	17.929957	33.66848
30	8	0	10.104561	19.302444	29.473846
31	6	0	9.010321	19.577866	31.760968
32	1	0	8.191152	21.465937	31.935223
33	6	0	7.513581	18.765652	36.081387
34	1	0	8.015904	20.726818	36.600331
35	6	0	8.637441	16.89086	38.017037
36	6	0	12.931632	15.753508	30.413097
37	1	0	12.552464	17.793967	27.00934
38	6	0	11.90175	17.540068	28.950088
39	1	0	14.423293	14.669559	29.486744
40	6	0	15.669651	5.464176	35.831079
41	6	0	15.868542	6.683621	40.117293
42	6	0	13.406857	7.590462	40.241283

43	1	0	12.056395	7.142954	38.758444
44	6	0	12.681628	9.222712	42.191868
45	6	0	10.063242	10.369488	42.230508
46	1	0	9.56649	10.731678	44.205154
47	1	0	8.667298	9.03551	41.457725
48	6	0	9.735883	12.861074	40.755539
49	6	0	9.506253	12.434396	37.854952
50	6	0	6.573899	14.070776	44.194442
51	1	0	8.057169	14.367713	45.625569
52	1	0	5.698585	12.20897	44.471808
53	1	0	5.127795	15.537906	44.406598
54	6	0	6.943647	16.427921	40.290388
55	6	0	14.44707	9.80509	44.086459
56	1	0	18.283261	9.285757	45.465617
57	6	0	16.90077	8.83299	44.003137
58	1	0	13.90919	11.050927	45.642658
59	6	0	17.649849	7.2839	41.998706
60	6	0	14.968412	7.818891	34.926506
61	8	0	5.250436	17.887688	40.884263

**3b**000002_en_		Standard Orientation (A.U.)			
Center number	Atomic number	Atomic Type	X	Y	Z
0	16	0	13.232612	14.483343	41.018309
1	16	0	11.995009	18.074227	39.655762
2	7	0	7.994781	14.241032	41.812331
3	8	0	15.395469	0.75921	34.86511
4	8	0	13.345913	12.369899	33.321475
5	8	0	5.160657	18.578729	36.234319
6	1	0	4.551757	18.845157	37.954858
7	8	0	10.740206	10.883894	30.272467
8	8	0	16.942078	5.148038	37.766532
9	1	0	15.53066	9.400011	35.688822
10	8	0	9.748194	10.338448	36.867608
11	8	0	20.282627	6.156604	41.420618
12	1	0	20.39255	5.33245	39.785332
13	7	0	9.376392	14.658044	36.696994
14	6	0	15.713248	0.053529	37.437525
15	1	0	15.509344	-2.012499	37.488874
16	1	0	14.252037	0.915323	38.647824
17	1	0	17.585538	0.582311	38.168274
18	6	0	14.860216	3.163018	34.199339
19	6	0	13.442038	3.480028	31.965408
20	1	0	11.453822	6.073572	29.509562
21	6	0	12.675803	5.854362	31.156016
22	1	0	12.881658	1.768287	30.959944
23	6	0	13.411956	8.01346	32.513035
24	6	0	12.33935	10.518179	31.863193
25	6	0	12.461562	14.915717	33.055819

26	1	0	13.668818	15.959748	34.388532
27	6	0	9.698921	15.244964	33.980082
28	1	0	8.471981	14.008939	32.847353
29	6	0	8.936385	17.995131	33.868143
30	8	0	10.092626	19.414224	29.641974
31	6	0	9.11323	19.673646	31.982376
32	1	0	8.348637	21.576548	32.228095
33	6	0	7.797991	18.810066	36.35258
34	1	0	8.382429	20.742565	36.892705
35	6	0	8.945268	16.858093	38.196253
36	6	0	12.884842	15.793183	30.387895
37	1	0	12.397216	17.905989	27.042643
38	6	0	11.828215	17.627622	29.005549
39	1	0	14.312147	14.69805	29.376927
40	6	0	15.619804	5.351369	35.516109
41	6	0	16.078821	6.555567	39.796359
42	6	0	13.626645	7.46949	40.012019
43	1	0	12.243317	7.106581	38.53676
44	6	0	12.954798	9.013125	42.053254
45	6	0	10.354994	10.194424	42.187616
46	1	0	9.905238	10.497741	44.182924
47	1	0	8.92271	8.908468	41.400509
48	6	0	10.040419	12.736503	40.796007
49	6	0	9.692097	12.38816	37.896793
50	6	0	7.08268	13.965791	44.40045
51	1	0	8.653194	14.164391	45.753042
52	1	0	6.140281	12.138133	44.686608
53	1	0	5.716566	15.488782	44.720334
54	6	0	7.333294	16.386823	40.526343
55	6	0	14.761856	9.490035	43.936106
56	1	0	18.624929	8.890523	45.20471
57	6	0	17.208343	8.510944	43.754159
58	1	0	14.267757	10.666624	45.559218
59	6	0	17.905916	7.058917	41.662476
60	6	0	14.961042	7.735614	34.624731
61	8	0	5.711669	17.879259	41.227006

**3b**000003_en_		Standard Orientation (A.U.)			
Center number	Atomic number	Atomic Type	X	Y	Z
0	16	0	13.111283	13.739633	40.490069
1	16	0	12.825216	17.435772	38.83608
2	7	0	8.244852	15.191991	41.968227
3	8	0	17.856251	1.999934	35.845299
4	8	0	11.918804	11.933756	33.027942
5	8	0	6.035874	20.214305	36.732708
6	1	0	6.073556	20.722017	38.509008
7	8	0	15.511153	12.727289	30.8747
8	8	0	13.389316	3.834508	37.596869

9	1	0	11.875598	8.169211	35.435326
10	8	0	8.094189	10.648086	37.187199
11	8	0	17.450791	3.880103	40.952779
12	1	0	17.326081	2.97331	39.347505
13	7	0	8.847437	14.880076	36.71673
14	6	0	16.624636	-0.322536	35.218048
15	1	0	17.637501	-1.823007	36.233098
16	1	0	16.744674	-0.673284	33.166991
17	1	0	14.63043	-0.296189	35.812485
18	6	0	16.87273	4.135888	34.751198
19	6	0	18.24236	5.464102	32.921054
20	1	0	18.437172	8.902838	30.695962
21	6	0	17.356288	7.787117	32.051765
22	1	0	20.041067	4.684151	32.279047
23	6	0	15.05866	8.753906	32.961426
24	6	0	14.247238	11.317828	32.153952
25	6	0	11.066713	14.420297	32.398043
26	1	0	12.60287	15.739308	32.863969
27	6	0	8.685796	15.154948	33.965316
28	1	0	7.07723	14.02801	33.290928
29	6	0	8.369858	17.973916	33.644226
30	8	0	7.998048	18.447088	29.096989
31	6	0	8.093845	19.297556	31.508044
32	1	0	7.834187	21.344727	31.569237
33	6	0	8.460126	19.320903	36.165821
34	1	0	9.869944	20.867275	36.173332
35	6	0	9.337589	17.197289	38.007049
36	6	0	10.378073	14.51038	29.634901
37	1	0	8.616528	15.920347	26.337693
38	6	0	9.002914	16.19904	28.344747
39	1	0	11.024213	12.903237	28.51718
40	6	0	14.56964	5.106472	35.64013
41	6	0	13.285984	5.324515	39.766249
42	6	0	11.249263	6.924299	40.16211
43	1	0	9.669049	6.888391	38.846093
44	6	0	11.296162	8.683284	42.133417
45	6	0	9.233446	10.63044	42.425846
46	1	0	9.1558	11.167051	44.419803
47	1	0	7.382586	9.834375	41.910406
48	6	0	9.546243	13.061636	40.845613
49	6	0	8.718939	12.67815	38.05915
50	6	0	7.753613	15.356724	44.680028
51	1	0	6.347773	13.95406	45.282492
52	1	0	7.018456	17.257271	45.043517
53	1	0	9.516005	15.093822	45.757125
54	6	0	8.132741	17.388298	40.61159
55	6	0	13.378349	8.665702	43.781522
56	1	0	17.027641	7.021081	44.686924
57	6	0	15.395451	7.00751	43.425503

58	1	0	13.443963	9.999683	45.357467
59	6	0	15.417538	5.345515	41.361737
60	6	0	13.640428	7.388239	34.722923
61	8	0	7.298371	19.380286	41.444513

**3b**000004_en_		Standard Orientation (A.U.)			
Center number	Atomic number	Atomic Type	X	Y	Z
0	16	0	13.274353	13.559082	41.52977
1	16	0	13.636825	17.271316	40.006022
2	7	0	8.337182	15.480612	41.831612
3	8	0	17.024586	1.773534	36.087911
4	8	0	11.917535	11.953925	32.720626
5	8	0	9.798556	21.830324	37.00493
6	1	0	8.517125	21.60073	38.322594
7	8	0	15.445916	12.313752	30.365594
8	8	0	12.804902	3.921803	37.628781
9	1	0	11.618721	8.28806	35.338236
10	8	0	8.329258	11.099752	37.213037
11	8	0	16.426916	3.27933	41.294973
12	1	0	16.204131	2.455529	39.656649
13	7	0	10.088982	15.016127	36.874193
14	6	0	19.323211	0.559069	35.450328
15	1	0	19.473965	-1.095851	36.691283
16	1	0	20.959396	1.812168	35.768941
17	1	0	19.315118	-0.072734	33.46395
18	6	0	16.368811	3.933645	34.864545
19	6	0	17.785747	5.073365	32.937356
20	1	0	18.126604	8.321927	30.471001
21	6	0	17.007339	7.380257	31.924522
22	1	0	19.524464	4.20105	32.261956
23	6	0	14.799388	8.552901	32.790242
24	6	0	14.142255	11.096765	31.797121
25	6	0	11.293127	14.541971	32.227371
26	1	0	13.04877	15.649763	32.38794
27	6	0	9.375307	15.421695	34.227887
28	1	0	7.595011	14.404397	33.885149
29	6	0	9.115739	18.23605	34.063535
30	8	0	7.217421	18.473651	29.957607
31	6	0	8.088786	19.510747	32.147505
32	1	0	7.865691	21.562579	32.195172
33	6	0	10.554687	19.448914	36.23703
34	1	0	12.541	19.609396	35.597076
35	6	0	10.550416	17.313699	38.274714
36	6	0	10.110715	14.819948	29.654548
37	1	0	7.568159	16.233486	26.924265
38	6	0	8.319849	16.424641	28.83736
39	1	0	10.738397	13.43991	28.255501
40	6	0	14.117941	5.097163	35.701106

41	6	0	12.645735	5.326253	39.841954
42	6	0	10.743384	7.090453	40.186874
43	1	0	9.303634	7.308733	38.737853
44	6	0	10.744231	8.668223	42.313853
45	6	0	8.8998	10.841417	42.534942
46	1	0	8.714202	11.346869	44.534254
47	1	0	7.019797	10.305248	41.8255
48	6	0	9.709818	13.228044	41.068663
49	6	0	9.320023	12.927194	38.186887
50	6	0	7.115653	15.703386	44.29201
51	1	0	6.365422	17.628913	44.426455
52	1	0	8.497921	15.399637	45.819263
53	1	0	5.551088	14.352658	44.492698
54	6	0	8.607123	17.620847	40.392654
55	6	0	12.618368	8.283732	44.154449
56	1	0	15.950535	6.162905	45.277079
57	6	0	14.488333	6.446213	43.849671
58	1	0	12.641065	9.46323	45.850103
59	6	0	14.56491	4.972043	41.652783
60	6	0	13.337067	7.377668	34.666466
61	8	0	7.480136	19.599338	40.81387

**3b**000005_en_		Standard Orientation (A.U.)			
Center number	Atomic number	Atomic Type	X	Y	Z
0	16	0	12.982965	14.547795	40.928287
1	16	0	11.765489	18.113287	39.474247
2	7	0	7.726369	14.27063	41.56664
3	8	0	15.438428	0.788004	35.102181
4	8	0	13.311582	12.340853	33.250612
5	8	0	5.021987	18.537894	35.875397
6	1	0	4.368246	18.815767	37.577894
7	8	0	10.729402	10.754012	30.233376
8	8	0	16.936073	5.265571	37.899717
9	1	0	15.585016	9.450744	35.65634
10	8	0	9.64077	10.331789	36.719488
11	8	0	20.104863	6.317576	41.678492
12	1	0	20.27897	5.480541	40.055847
13	7	0	9.24881	14.646301	36.492034
14	6	0	17.555322	0.209462	36.645746
15	1	0	19.264475	1.202321	35.985362
16	1	0	17.84593	-1.839092	36.467562
17	1	0	17.22418	0.692423	38.640117
18	6	0	14.924185	3.171076	34.355601
19	6	0	13.415702	3.412907	32.171721
20	1	0	11.34025	5.926014	29.70356
21	6	0	12.611449	5.758544	31.31843
22	1	0	12.839335	1.669547	31.231659
23	6	0	13.407469	7.963217	32.564166

24	6	0	12.323618	10.44282	31.840586
25	6	0	12.407086	14.871171	32.911697
26	1	0	13.581026	15.956143	34.241337
27	6	0	9.624644	15.199006	33.776557
28	1	0	8.42644	13.942729	32.635675
29	6	0	8.856681	17.944852	33.612504
30	8	0	10.101375	19.31539	29.394237
31	6	0	9.074672	19.601562	31.711807
32	1	0	8.305369	21.507133	31.918326
33	6	0	7.654202	18.784471	36.058643
34	1	0	8.214492	20.726812	36.589248
35	6	0	8.765298	16.861552	37.955537
36	6	0	12.864514	15.691832	30.231218
37	1	0	12.424236	17.751132	26.845498
38	6	0	11.833056	17.508471	28.806622
39	1	0	14.294213	14.569398	29.254514
40	6	0	15.682865	5.399521	35.602712
41	6	0	15.968965	6.677946	39.880308
42	6	0	13.502933	7.571731	39.992184
43	1	0	12.179278	7.193703	38.467185
44	6	0	12.733952	9.103425	42.008844
45	6	0	10.114749	10.248668	42.048836
46	1	0	9.597303	10.561891	44.026049
47	1	0	8.727045	8.937215	41.225238
48	6	0	9.816448	12.77447	40.626344
49	6	0	9.547071	12.392518	37.723937
50	6	0	6.712774	13.98917	44.115519
51	1	0	5.305361	15.486787	44.36999
52	1	0	8.218685	14.224939	45.534254
53	1	0	5.79558	12.144222	44.369744
54	6	0	7.091076	16.401492	40.245319
55	6	0	14.457588	9.602564	43.962516
56	1	0	18.273685	9.043996	45.384763
57	6	0	16.919652	8.650395	43.87935
58	1	0	13.883575	10.772776	45.563668
59	6	0	17.712195	7.201798	41.820071
60	6	0	15.014194	7.755149	34.642045
61	8	0	5.440009	17.889667	40.88469

**3b**000006_en_		Standard Orientation (A.U.)			
Center number	Atomic number	Atomic Type	X	Y	Z
0	16	0	13.045777	13.778387	41.541643
1	16	0	13.25506	17.49978	40.012797
2	7	0	8.006751	15.437444	41.691536
3	8	0	17.604986	2.204349	36.301727
4	8	0	12.0574	12.072272	32.71563
5	8	0	9.264577	21.834717	36.870157
6	1	0	7.962645	21.540575	38.15462

7	8	0	15.629308	12.612947	30.462691
8	8	0	13.227047	4.123771	37.694818
9	1	0	11.877479	8.409496	35.341097
10	8	0	8.369792	11.041416	37.098277
11	8	0	16.754828	3.689134	41.477238
12	1	0	16.628308	2.850549	39.836141
13	7	0	9.925265	15.046362	36.789824
14	6	0	19.990206	1.120966	35.751623
15	1	0	20.193074	-0.515641	37.009343
16	1	0	21.53983	2.46805	36.11654
17	1	0	20.086747	0.478903	33.770909
18	6	0	16.871517	4.320062	35.045409
19	6	0	18.285002	5.527675	33.157575
20	1	0	18.525524	8.778373	30.682438
21	6	0	17.413586	7.783156	32.105564
22	1	0	20.090156	4.749641	32.543953
23	6	0	15.11762	8.835462	32.893286
24	6	0	14.352326	11.333918	31.863727
25	6	0	11.306684	14.61901	32.186395
26	1	0	12.992005	15.823014	32.398154
27	6	0	9.278683	15.40126	34.119043
28	1	0	7.568434	14.287092	33.724817
29	6	0	8.875541	18.197124	33.928686
30	8	0	7.117276	18.329529	29.754138
31	6	0	7.853931	19.411986	31.971108
32	1	0	7.524514	21.449747	32.003354
33	6	0	10.174945	19.495037	36.141282
34	1	0	12.168998	19.762315	35.564184
35	6	0	10.22302	17.371006	38.189883
36	6	0	10.194288	14.820366	29.575431
37	1	0	7.675226	16.088216	26.753346
38	6	0	8.353936	16.329003	28.687974
39	1	0	10.932619	13.462664	28.208874
40	6	0	14.534356	5.361195	35.802936
41	6	0	12.919397	5.525692	39.893565
42	6	0	10.915085	7.186333	40.166658
43	1	0	9.514816	7.32407	38.670094
44	6	0	10.760356	8.76672	42.286195
45	6	0	8.794732	10.837453	42.438248
46	1	0	8.523066	11.341751	44.427938
47	1	0	6.968398	10.195317	41.677919
48	6	0	9.517497	13.257322	40.981025
49	6	0	9.229954	12.924279	38.0907
50	6	0	6.707109	15.606363	44.11602
51	1	0	5.205542	14.180911	44.278752
52	1	0	5.859655	17.492938	44.222182
53	1	0	8.059335	15.374636	45.68207
54	6	0	8.207516	17.584047	40.25063
55	6	0	12.589156	8.486825	44.190187

56	1	0	15.990245	6.550828	45.432847
57	6	0	14.563992	6.751443	43.955918
58	1	0	12.490778	9.66926	45.881081
59	6	0	14.793457	5.279212	41.76844
60	6	0	13.662961	7.590247	34.729642
61	8	0	6.970868	19.504429	40.631641

**3b**000007_en_		Standard Orientation (A.U.)			
Center number	Atomic number	Atomic Type	X	Y	Z
0	16	0	12.870554	14.695797	41.053954
1	16	0	11.592338	18.222469	39.561365
2	7	0	7.609671	14.332535	41.65239
3	8	0	15.958638	1.057572	35.416944
4	8	0	13.338376	12.399038	33.43448
5	8	0	4.892061	18.44992	35.856073
6	1	0	4.202175	18.734223	37.543043
7	8	0	10.911045	10.798711	30.29791
8	8	0	16.773244	5.18656	38.174879
9	1	0	15.355745	9.501502	36.040842
10	8	0	9.647333	10.364425	36.87649
11	8	0	20.030618	6.377022	41.858708
12	1	0	20.141865	5.375814	40.324087
13	7	0	9.183612	14.668151	36.589239
14	6	0	15.459192	-1.214845	34.111379
15	1	0	13.409907	-1.527101	33.866814
16	1	0	16.245396	-2.743573	35.272894
17	1	0	16.375351	-1.242984	32.235964
18	6	0	15.166411	3.259613	34.410753
19	6	0	13.866516	3.505082	32.105713
20	1	0	11.895806	6.044016	29.56869
21	6	0	13.017768	5.865262	31.289678
22	1	0	13.44413	1.834268	30.978871
23	6	0	13.559224	8.035742	32.698546
24	6	0	12.443549	10.498815	31.966684
25	6	0	12.410476	14.918744	33.077493
26	1	0	13.534557	16.016578	34.439447
27	6	0	9.604678	15.202032	33.875298
28	1	0	8.455255	13.911919	32.721642
29	6	0	8.783038	17.929957	33.66848
30	8	0	10.104561	19.302444	29.473846
31	6	0	9.010321	19.577866	31.760968
32	1	0	8.191152	21.465937	31.935223
33	6	0	7.513581	18.765652	36.081387
34	1	0	8.015904	20.726818	36.600331
35	6	0	8.637441	16.89086	38.017037
36	6	0	12.931632	15.753508	30.413097
37	1	0	12.552464	17.793967	27.00934
38	6	0	11.90175	17.540068	28.950088

39	1	0	14.423293	14.669559	29.486744
40	6	0	15.669651	5.464176	35.831079
41	6	0	15.868542	6.683621	40.117293
42	6	0	13.406857	7.590462	40.241283
43	1	0	12.056395	7.142954	38.758444
44	6	0	12.681628	9.222712	42.191868
45	6	0	10.063242	10.369488	42.230508
46	1	0	9.56649	10.731678	44.205154
47	1	0	8.667298	9.03551	41.457725
48	6	0	9.735883	12.861074	40.755539
49	6	0	9.506253	12.434396	37.854952
50	6	0	6.573899	14.070776	44.194442
51	1	0	8.057169	14.367713	45.625569
52	1	0	5.698585	12.20897	44.471808
53	1	0	5.127795	15.537906	44.406598
54	6	0	6.943647	16.427921	40.290388
55	6	0	14.44707	9.80509	44.086459
56	1	0	18.283261	9.285757	45.465617
57	6	0	16.90077	8.83299	44.003137
58	1	0	13.90919	11.050927	45.642658
59	6	0	17.649849	7.2839	41.998706
60	6	0	14.968412	7.818891	34.926506
61	8	0	5.250436	17.887688	40.884263

**3b**000008_en_		Standard Orientation (A.U.)			
Center number	Atomic number	Atomic Type	X	Y	Z
0	16	0	12.872142	14.696536	41.053372
1	16	0	11.596784	18.223574	39.559174
2	7	0	7.611219	14.338928	41.654505
3	8	0	15.944663	1.051814	35.422084
4	8	0	13.334368	12.394891	33.435192
5	8	0	4.894947	18.455285	35.856864
6	1	0	4.206066	18.741292	37.543954
7	8	0	10.904381	10.795257	30.300324
8	8	0	16.763512	5.181121	38.178209
9	1	0	15.349388	9.496553	36.042661
10	8	0	9.642726	10.365878	36.880013
11	8	0	20.023808	6.370049	41.859978
12	1	0	20.133276	5.367807	40.325904
13	7	0	9.183047	14.669887	36.590372
14	6	0	15.442962	-1.220679	34.117511
15	1	0	13.393354	-1.531265	33.873534
16	1	0	16.228114	-2.749635	35.279436
17	1	0	16.358668	-1.25033	32.241899
18	6	0	15.154071	3.254145	34.415239
19	6	0	13.853836	3.499814	32.110419
20	1	0	11.884759	6.039447	29.572825
21	6	0	13.006975	5.860404	31.293615

22	1	0	13.429727	1.828909	30.984358
23	6	0	13.550734	8.030981	32.701444
24	6	0	12.437177	10.494758	31.968723
25	6	0	12.40879	14.915314	33.077211
26	1	0	13.534463	16.012775	34.438147
27	6	0	9.603576	15.201799	33.875952
28	1	0	8.45243	13.912162	32.723485
29	6	0	8.784528	17.93042	33.667902
30	8	0	10.105848	19.299341	29.472032
31	6	0	9.012714	19.577058	31.759399
32	1	0	8.195449	21.46602	31.932937
33	6	0	7.51686	18.76872	36.080847
34	1	0	8.021229	20.729718	36.598424
35	6	0	8.639779	16.893992	38.017095
36	6	0	12.929759	15.74813	30.412167
37	1	0	12.551324	17.787087	27.00743
38	6	0	11.901091	17.534899	28.948561
39	1	0	14.419986	14.662193	29.48584
40	6	0	15.659568	5.458834	35.834585
41	6	0	15.861334	6.680342	40.120134
42	6	0	13.400706	7.589981	40.244687
43	1	0	12.049084	7.143092	38.762715
44	6	0	12.678132	9.224144	42.194659
45	6	0	10.061003	10.373775	42.233883
46	1	0	9.565688	10.737794	44.20855
47	1	0	8.663195	9.040795	41.462737
48	6	0	9.735502	12.864778	40.75751
49	6	0	9.504095	12.436576	37.857288
50	6	0	6.576527	14.079847	44.197271
51	1	0	8.060872	14.376161	45.627411
52	1	0	5.699448	12.219121	44.47629
53	1	0	5.132047	15.548599	44.409266
54	6	0	6.946628	16.434139	40.291539
55	6	0	14.445053	9.805642	44.088138
56	1	0	18.281257	9.282825	45.465936
57	6	0	16.897635	8.830767	44.004308
58	1	0	13.90925	11.052948	45.64388
59	6	0	17.64412	7.279691	42.000446
60	6	0	14.960249	7.813803	34.92917
61	8	0	5.255167	17.895966	40.885328

**3b**000009_en_		Standard Orientation (A.U.)			
Center number	Atomic number	Atomic Type	X	Y	Z
0	16	0	13.232612	14.483343	41.018309
1	16	0	11.995009	18.074227	39.655762
2	7	0	7.994781	14.241032	41.812331
3	8	0	15.395469	0.75921	34.86511
4	8	0	13.345913	12.369899	33.321475

5	8	0	5.160657	18.578729	36.234319
6	1	0	4.551757	18.845157	37.954858
7	8	0	10.740206	10.883894	30.272467
8	8	0	16.942078	5.148038	37.766532
9	1	0	15.53066	9.400011	35.688822
10	8	0	9.748194	10.338448	36.867608
11	8	0	20.282627	6.156604	41.420618
12	1	0	20.39255	5.33245	39.785332
13	7	0	9.376392	14.658044	36.696994
14	6	0	15.713248	0.053529	37.437525
15	1	0	15.509344	-2.012499	37.488874
16	1	0	14.252037	0.915323	38.647824
17	1	0	17.585538	0.582311	38.168274
18	6	0	14.860216	3.163018	34.199339
19	6	0	13.442038	3.480028	31.965408
20	1	0	11.453822	6.073572	29.509562
21	6	0	12.675803	5.854362	31.156016
22	1	0	12.881658	1.768287	30.959944
23	6	0	13.411956	8.01346	32.513035
24	6	0	12.33935	10.518179	31.863193
25	6	0	12.461562	14.915717	33.055819
26	1	0	13.668818	15.959748	34.388532
27	6	0	9.698921	15.244964	33.980082
28	1	0	8.471981	14.008939	32.847353
29	6	0	8.936385	17.995131	33.868143
30	8	0	10.092626	19.414224	29.641974
31	6	0	9.11323	19.673646	31.982376
32	1	0	8.348637	21.576548	32.228095
33	6	0	7.797991	18.810066	36.35258
34	1	0	8.382429	20.742565	36.892705
35	6	0	8.945268	16.858093	38.196253
36	6	0	12.884842	15.793183	30.387895
37	1	0	12.397216	17.905989	27.042643
38	6	0	11.828215	17.627622	29.005549
39	1	0	14.312147	14.69805	29.376927
40	6	0	15.619804	5.351369	35.516109
41	6	0	16.078821	6.555567	39.796359
42	6	0	13.626645	7.46949	40.012019
43	1	0	12.243317	7.106581	38.53676
44	6	0	12.954798	9.013125	42.053254
45	6	0	10.354994	10.194424	42.187616
46	1	0	9.905238	10.497741	44.182924
47	1	0	8.92271	8.908468	41.400509
48	6	0	10.040419	12.736503	40.796007
49	6	0	9.692097	12.38816	37.896793
50	6	0	7.08268	13.965791	44.40045
51	1	0	8.653194	14.164391	45.753042
52	1	0	6.140281	12.138133	44.686608
53	1	0	5.716566	15.488782	44.720334

54	6	0	7.333294	16.386823	40.526343
55	6	0	14.761856	9.490035	43.936106
56	1	0	18.624929	8.890523	45.20471
57	6	0	17.208343	8.510944	43.754159
58	1	0	14.267757	10.666624	45.559218
59	6	0	17.905916	7.058917	41.662476
60	6	0	14.961042	7.735614	34.624731
61	8	0	5.711669	17.879259	41.227006

**3b**000010_en_		Standard Orientation (A.U.)			
Center number	Atomic number	Atomic Type	X	Y	Z
0	16	0	13.223008	14.496751	40.99704
1	16	0	12.07384	18.116843	39.631896
2	7	0	7.987693	14.420674	41.849362
3	8	0	15.319142	0.820558	34.984421
4	8	0	13.224791	12.330197	33.357743
5	8	0	5.233985	18.787099	36.247555
6	1	0	4.639751	19.076988	37.969528
7	8	0	10.59297	10.928345	30.292212
8	8	0	16.441162	4.825959	37.808632
9	1	0	15.176003	9.265537	35.829542
10	8	0	9.576362	10.430479	36.916614
11	8	0	19.922828	5.761547	41.361267
12	1	0	19.90876	4.78235	39.808558
13	7	0	9.331103	14.757788	36.719019
14	6	0	14.664939	-1.389342	33.64042
15	1	0	15.417901	-2.984624	34.732375
16	1	0	15.511055	-1.409017	31.732313
17	1	0	12.595614	-1.598542	33.463954
18	6	0	14.603545	3.084467	34.061858
19	6	0	13.235659	3.453991	31.813905
20	1	0	11.307632	6.152919	29.413342
21	6	0	12.480028	5.873999	31.086705
22	1	0	12.688178	1.836206	30.663646
23	6	0	13.183987	7.977632	32.524869
24	6	0	12.172539	10.511838	31.89068
25	6	0	12.422369	14.90306	33.082936
26	1	0	13.658053	15.910928	34.41736
27	6	0	9.669722	15.320368	33.999637
28	1	0	8.407229	14.115461	32.87247
29	6	0	8.988473	18.09144	33.873418
30	8	0	10.200081	19.460857	29.645637
31	6	0	9.22094	19.756907	31.982003
32	1	0	8.513188	21.682844	32.219002
33	6	0	7.876792	18.951269	36.355145
34	1	0	8.513143	20.871158	36.881205
35	6	0	8.97866	16.980215	38.205625
36	6	0	12.883198	15.762116	30.415251

37	1	0	12.474263	17.880026	27.062836
38	6	0	11.886945	17.622993	29.023308
39	1	0	14.283386	14.624091	29.413845
40	6	0	15.269435	5.223315	35.51579
41	6	0	15.712634	6.337536	39.813165
42	6	0	13.314917	7.380759	40.061541
43	1	0	11.87895	7.032469	38.633037
44	6	0	12.771476	9.018525	42.065777
45	6	0	10.227877	10.311488	42.240448
46	1	0	9.838068	10.659871	44.241326
47	1	0	8.725263	9.074448	41.507358
48	6	0	9.978087	12.846764	40.825201
49	6	0	9.590659	12.486922	37.932693
50	6	0	7.084875	14.187017	44.444589
51	1	0	8.668524	14.351784	45.78622
52	1	0	6.096117	12.387033	44.74895
53	1	0	5.762205	15.74836	44.762421
54	6	0	7.370969	16.57052	40.549849
55	6	0	14.650487	9.465083	43.88679
56	1	0	18.5061	8.702939	45.085817
57	6	0	17.037474	8.355225	43.679609
58	1	0	14.256801	10.712334	45.484624
59	6	0	17.607237	6.801071	41.620901
60	6	0	14.659661	7.633146	34.693075
61	8	0	5.789799	18.108429	41.245939

**3b**000011_en_		Standard Orientation (A.U.)			
Center number	Atomic number	Atomic Type	X	Y	Z
0	16	0	13.111283	13.739633	40.490069
1	16	0	12.825216	17.435772	38.83608
2	7	0	8.244852	15.191991	41.968227
3	8	0	17.856251	1.999934	35.845299
4	8	0	11.918804	11.933756	33.027942
5	8	0	6.035874	20.214305	36.732708
6	1	0	6.073556	20.722017	38.509008
7	8	0	15.511153	12.727289	30.8747
8	8	0	13.389316	3.834508	37.596869
9	1	0	11.875598	8.169211	35.435326
10	8	0	8.094189	10.648086	37.187199
11	8	0	17.450791	3.880103	40.952779
12	1	0	17.326081	2.97331	39.347505
13	7	0	8.847437	14.880076	36.71673
14	6	0	16.624636	-0.322536	35.218048
15	1	0	17.637501	-1.823007	36.233098
16	1	0	16.744674	-0.673284	33.166991
17	1	0	14.63043	-0.296189	35.812485
18	6	0	16.87273	4.135888	34.751198
19	6	0	18.24236	5.464102	32.921054

20	1	0	18.437172	8.902838	30.695962
21	6	0	17.356288	7.787117	32.051765
22	1	0	20.041067	4.684151	32.279047
23	6	0	15.05866	8.753906	32.961426
24	6	0	14.247238	11.317828	32.153952
25	6	0	11.066713	14.420297	32.398043
26	1	0	12.60287	15.739308	32.863969
27	6	0	8.685796	15.154948	33.965316
28	1	0	7.07723	14.02801	33.290928
29	6	0	8.369858	17.973916	33.644226
30	8	0	7.998048	18.447088	29.096989
31	6	0	8.093845	19.297556	31.508044
32	1	0	7.834187	21.344727	31.569237
33	6	0	8.460126	19.320903	36.165821
34	1	0	9.869944	20.867275	36.173332
35	6	0	9.337589	17.197289	38.007049
36	6	0	10.378073	14.51038	29.634901
37	1	0	8.616528	15.920347	26.337693
38	6	0	9.002914	16.19904	28.344747
39	1	0	11.024213	12.903237	28.51718
40	6	0	14.56964	5.106472	35.64013
41	6	0	13.285984	5.324515	39.766249
42	6	0	11.249263	6.924299	40.16211
43	1	0	9.669049	6.888391	38.846093
44	6	0	11.296162	8.683284	42.133417
45	6	0	9.233446	10.63044	42.425846
46	1	0	9.1558	11.167051	44.419803
47	1	0	7.382586	9.834375	41.910406
48	6	0	9.546243	13.061636	40.845613
49	6	0	8.718939	12.67815	38.05915
50	6	0	7.753613	15.356724	44.680028
51	1	0	6.347773	13.95406	45.282492
52	1	0	7.018456	17.257271	45.043517
53	1	0	9.516005	15.093822	45.757125
54	6	0	8.132741	17.388298	40.61159
55	6	0	13.378349	8.665702	43.781522
56	1	0	17.027641	7.021081	44.686924
57	6	0	15.395451	7.00751	43.425503
58	1	0	13.443963	9.999683	45.357467
59	6	0	15.417538	5.345515	41.361737
60	6	0	13.640428	7.388239	34.722923
61	8	0	7.298371	19.380286	41.444513

**3b**000012_en_		Standard Orientation (A.U.)			
Center number	Atomic number	Atomic Type	X	Y	Z
0	16	0	13.197565	13.309909	40.174597
1	16	0	13.139175	17.096947	38.709941
2	7	0	8.5366	15.06493	41.945353

3	8	0	16.938445	1.624009	34.898558
4	8	0	11.586679	12.005722	32.717842
5	8	0	6.51121	20.506326	37.063876
6	1	0	6.675043	20.918466	38.857794
7	8	0	15.111691	12.689484	30.420082
8	8	0	12.797146	3.626421	36.861798
9	1	0	11.457146	8.118138	34.974772
10	8	0	7.80317	10.795953	36.968793
11	8	0	16.731086	3.053319	40.186842
12	1	0	16.355356	2.197908	38.594573
13	7	0	8.88107	14.969965	36.662615
14	6	0	19.224043	0.510436	34.061875
15	1	0	19.438921	-1.255844	35.12734
16	1	0	20.857622	1.749673	34.442691
17	1	0	19.148408	0.075939	32.024458
18	6	0	16.214153	3.878798	33.909853
19	6	0	17.551971	5.2236	32.057411
20	1	0	17.805019	8.723022	29.950716
21	6	0	16.733228	7.619978	31.324276
22	1	0	19.270979	4.44276	31.235382
23	6	0	14.546692	8.671336	32.381039
24	6	0	13.837061	11.296748	31.709727
25	6	0	10.86606	14.563094	32.227906
26	1	0	12.505635	15.762689	32.66146
27	6	0	8.617674	15.388896	33.939143
28	1	0	6.901506	14.414296	33.293971
29	6	0	8.500949	18.235112	33.761599
30	8	0	7.947643	18.939377	29.263245
31	6	0	8.224249	19.671566	31.69966
32	1	0	8.125103	21.727621	31.858329
33	6	0	8.822692	19.448291	36.332466
34	1	0	10.354756	20.873728	36.335759
35	6	0	9.60951	17.174729	38.032377
36	6	0	10.050352	14.819032	29.510258
37	1	0	8.237027	16.501764	26.371985
38	6	0	8.743467	16.659851	28.365419
39	1	0	10.518265	13.218526	28.298101
40	6	0	13.991128	4.933058	34.937619
41	6	0	12.776204	4.996222	39.105446
42	6	0	10.881598	6.732271	39.607637
43	1	0	9.299662	6.915043	38.306013
44	6	0	11.076378	8.347074	41.693809
45	6	0	9.165945	10.41927	42.134032
46	1	0	9.171816	10.857814	44.153052
47	1	0	7.252726	9.779049	41.629271
48	6	0	9.6097	12.897072	40.663074
49	6	0	8.63109	12.721934	37.904729
50	6	0	8.196547	15.135851	44.684149
51	1	0	6.702848	13.832601	45.299099

52	1	0	7.651739	17.072264	45.17242
53	1	0	9.98009	14.668963	45.651883
54	6	0	8.541251	17.32845	40.698259
55	6	0	13.150315	8.054031	43.324756
56	1	0	16.629702	6.025959	44.132483
57	6	0	15.012713	6.241618	42.869732
58	1	0	13.327717	9.281088	44.97722
59	6	0	14.876971	4.712492	40.714507
60	6	0	13.152375	7.290575	34.156405
61	8	0	7.910405	19.33626	41.662733

**3b**000013_en_		Standard Orientation (A.U.)			
Center number	Atomic number	Atomic Type	X	Y	Z
0	16	0	13.274353	13.559082	41.52977
1	16	0	13.636825	17.271316	40.006022
2	7	0	8.337182	15.480612	41.831612
3	8	0	17.024586	1.773534	36.087911
4	8	0	11.917535	11.953925	32.720626
5	8	0	9.798556	21.830324	37.00493
6	1	0	8.517125	21.60073	38.322594
7	8	0	15.445916	12.313752	30.365594
8	8	0	12.804902	3.921803	37.628781
9	1	0	11.618721	8.28806	35.338236
10	8	0	8.329258	11.099752	37.213037
11	8	0	16.426916	3.27933	41.294973
12	1	0	16.204131	2.455529	39.656649
13	7	0	10.088982	15.016127	36.874193
14	6	0	19.323211	0.559069	35.450328
15	1	0	19.473965	-1.095851	36.691283
16	1	0	20.959396	1.812168	35.768941
17	1	0	19.315118	-0.072734	33.46395
18	6	0	16.368811	3.933645	34.864545
19	6	0	17.785747	5.073365	32.937356
20	1	0	18.126604	8.321927	30.471001
21	6	0	17.007339	7.380257	31.924522
22	1	0	19.524464	4.20105	32.261956
23	6	0	14.799388	8.552901	32.790242
24	6	0	14.142255	11.096765	31.797121
25	6	0	11.293127	14.541971	32.227371
26	1	0	13.04877	15.649763	32.38794
27	6	0	9.375307	15.421695	34.227887
28	1	0	7.595011	14.404397	33.885149
29	6	0	9.115739	18.23605	34.063535
30	8	0	7.217421	18.473651	29.957607
31	6	0	8.088786	19.510747	32.147505
32	1	0	7.865691	21.562579	32.195172
33	6	0	10.554687	19.448914	36.23703
34	1	0	12.541	19.609396	35.597076

35	6	0	10.550416	17.313699	38.274714
36	6	0	10.110715	14.819948	29.654548
37	1	0	7.568159	16.233486	26.924265
38	6	0	8.319849	16.424641	28.83736
39	1	0	10.738397	13.43991	28.255501
40	6	0	14.117941	5.097163	35.701106
41	6	0	12.645735	5.326253	39.841954
42	6	0	10.743384	7.090453	40.186874
43	1	0	9.303634	7.308733	38.737853
44	6	0	10.744231	8.668223	42.313853
45	6	0	8.8998	10.841417	42.534942
46	1	0	8.714202	11.346869	44.534254
47	1	0	7.019797	10.305248	41.8255
48	6	0	9.709818	13.228044	41.068663
49	6	0	9.320023	12.927194	38.186887
50	6	0	7.115653	15.703386	44.29201
51	1	0	6.365422	17.628913	44.426455
52	1	0	8.497921	15.399637	45.819263
53	1	0	5.551088	14.352658	44.492698
54	6	0	8.607123	17.620847	40.392654
55	6	0	12.618368	8.283732	44.154449
56	1	0	15.950535	6.162905	45.277079
57	6	0	14.488333	6.446213	43.849671
58	1	0	12.641065	9.46323	45.850103
59	6	0	14.56491	4.972043	41.652783
60	6	0	13.337067	7.377668	34.666466
61	8	0	7.480136	19.599338	40.81387

**3b**000014_en_		Standard Orientation (A.U.)			
Center number	Atomic number	Atomic Type	X	Y	Z
0	16	0	13.26975	13.56793	41.535077
1	16	0	13.62672	17.280352	40.010599
2	7	0	8.328475	15.480069	41.830187
3	8	0	17.048531	1.786409	36.103415
4	8	0	11.92704	11.955969	32.725297
5	8	0	9.783207	21.83039	37.002343
6	1	0	8.500497	21.598785	38.318409
7	8	0	15.457596	12.321139	30.374346
8	8	0	12.822965	3.927689	37.638047
9	1	0	11.631608	8.290622	35.343959
10	8	0	8.334396	11.097295	37.213563
11	8	0	16.442036	3.293883	41.308641
12	1	0	16.222523	2.468728	39.670555
13	7	0	10.087367	15.01675	36.8751
14	6	0	19.350546	0.576292	35.469781
15	1	0	19.502886	-1.077654	36.711842
16	1	0	20.983702	1.832908	35.790087
17	1	0	19.346604	-0.056593	33.483737

18	6	0	16.390326	3.944774	34.878258
19	6	0	17.807612	5.086271	32.95238
20	1	0	18.145632	8.334365	30.485037
21	6	0	17.02623	7.391273	31.937529
22	1	0	19.548812	4.216878	32.279606
23	6	0	14.814989	8.560197	32.799882
24	6	0	14.154423	11.102426	31.804846
25	6	0	11.29848	14.54263	32.230046
26	1	0	13.051845	15.653747	32.392503
27	6	0	9.376376	15.419701	34.227656
28	1	0	7.598479	14.39883	33.883089
29	6	0	9.111688	18.233481	34.061606
30	8	0	7.218921	18.466094	29.952756
31	6	0	8.085156	19.505511	32.143573
32	1	0	7.858215	21.556945	32.190182
33	6	0	10.545228	19.450165	36.236552
34	1	0	12.532083	19.614424	35.599249
35	6	0	10.542499	17.315861	38.275165
36	6	0	10.119005	14.817278	29.655511
37	1	0	7.577781	16.225067	26.921028
38	6	0	8.32647	16.418456	28.835076
39	1	0	10.750978	13.437657	28.257983
40	6	0	14.136244	5.104499	35.711424
41	6	0	12.658666	5.332954	39.850324
42	6	0	10.752635	7.09378	40.192144
43	1	0	9.314162	7.308628	38.741344
44	6	0	10.748051	8.672563	42.318359
45	6	0	8.899221	10.842331	42.536336
46	1	0	8.710379	11.348337	44.535203
47	1	0	7.021052	10.302217	41.825011
48	6	0	9.706357	13.229824	41.069917
49	6	0	9.320627	12.926978	38.187799
50	6	0	7.103911	15.701767	44.289171
51	1	0	6.350163	17.626034	44.42198
52	1	0	8.485093	15.401108	45.818012
53	1	0	5.541475	14.348415	44.488837
54	6	0	8.595939	17.620137	40.390504
55	6	0	12.62074	8.29245	44.161333
56	1	0	15.955567	6.178462	45.288884
57	6	0	14.494509	6.458303	43.859622
58	1	0	12.639202	9.472759	45.856474
59	6	0	14.576418	4.983228	41.663533
60	6	0	13.352459	7.383078	34.674749
61	8	0	7.464492	19.596573	40.809373

**3b**000015_en_		Standard Orientation (A.U.)			
Center number	Atomic number	Atomic Type	X	Y	Z
0	16	0	12.982965	14.547795	40.928287

1	16	0	11.765489	18.113287	39.474247
2	7	0	7.726369	14.27063	41.56664
3	8	0	15.438428	0.788004	35.102181
4	8	0	13.311582	12.340853	33.250612
5	8	0	5.021987	18.537894	35.875397
6	1	0	4.368246	18.815767	37.577894
7	8	0	10.729402	10.754012	30.233376
8	8	0	16.936073	5.265571	37.899717
9	1	0	15.585016	9.450744	35.65634
10	8	0	9.64077	10.331789	36.719488
11	8	0	20.104863	6.317576	41.678492
12	1	0	20.27897	5.480541	40.055847
13	7	0	9.24881	14.646301	36.492034
14	6	0	17.555322	0.209462	36.645746
15	1	0	19.264475	1.202321	35.985362
16	1	0	17.84593	-1.839092	36.467562
17	1	0	17.22418	0.692423	38.640117
18	6	0	14.924185	3.171076	34.355601
19	6	0	13.415702	3.412907	32.171721
20	1	0	11.34025	5.926014	29.70356
21	6	0	12.611449	5.758544	31.31843
22	1	0	12.839335	1.669547	31.231659
23	6	0	13.407469	7.963217	32.564166
24	6	0	12.323618	10.44282	31.840586
25	6	0	12.407086	14.871171	32.911697
26	1	0	13.581026	15.956143	34.241337
27	6	0	9.624644	15.199006	33.776557
28	1	0	8.42644	13.942729	32.635675
29	6	0	8.856681	17.944852	33.612504
30	8	0	10.101375	19.31539	29.394237
31	6	0	9.074672	19.601562	31.711807
32	1	0	8.305369	21.507133	31.918326
33	6	0	7.654202	18.784471	36.058643
34	1	0	8.214492	20.726812	36.589248
35	6	0	8.765298	16.861552	37.955537
36	6	0	12.864514	15.691832	30.231218
37	1	0	12.424236	17.751132	26.845498
38	6	0	11.833056	17.508471	28.806622
39	1	0	14.294213	14.569398	29.254514
40	6	0	15.682865	5.399521	35.602712
41	6	0	15.968965	6.677946	39.880308
42	6	0	13.502933	7.571731	39.992184
43	1	0	12.179278	7.193703	38.467185
44	6	0	12.733952	9.103425	42.008844
45	6	0	10.114749	10.248668	42.048836
46	1	0	9.597303	10.561891	44.026049
47	1	0	8.727045	8.937215	41.225238
48	6	0	9.816448	12.77447	40.626344
49	6	0	9.547071	12.392518	37.723937

50	6	0	6.712774	13.98917	44.115519
51	1	0	5.305361	15.486787	44.36999
52	1	0	8.218685	14.224939	45.534254
53	1	0	5.79558	12.144222	44.369744
54	6	0	7.091076	16.401492	40.245319
55	6	0	14.457588	9.602564	43.962516
56	1	0	18.273685	9.043996	45.384763
57	6	0	16.919652	8.650395	43.87935
58	1	0	13.883575	10.772776	45.563668
59	6	0	17.712195	7.201798	41.820071
60	6	0	15.014194	7.755149	34.642045
61	8	0	5.440009	17.889667	40.88469

**3b**000016_en_		Standard Orientation (A.U.)			
Center number	Atomic number	Atomic Type	X	Y	Z
0	16	0	13.007482	14.5536	40.884904
1	16	0	11.754278	18.124554	39.479101
2	7	0	7.757699	14.263946	41.59744
3	8	0	15.806549	0.988069	34.965612
4	8	0	13.271765	12.388423	33.223514
5	8	0	4.972977	18.524613	35.939774
6	1	0	4.330145	18.792956	37.647809
7	8	0	10.753971	10.865135	30.119873
8	8	0	16.723561	5.060291	37.772566
9	1	0	15.311538	9.426032	35.739496
10	8	0	9.62752	10.345826	36.709971
11	8	0	20.0617	6.134146	41.419796
12	1	0	20.132422	5.161622	39.864491
13	7	0	9.219081	14.66006	36.505088
14	6	0	15.255869	-1.257322	33.634066
15	1	0	13.198701	-1.5457	33.429673
16	1	0	16.051954	-2.812818	34.752517
17	1	0	16.130389	-1.262901	31.7387
18	6	0	15.016216	3.214288	34.012828
19	6	0	13.669131	3.511548	31.74136
20	1	0	11.671013	6.113086	29.290742
21	6	0	12.828564	5.893875	30.983224
22	1	0	13.204225	1.864307	30.596649
23	6	0	13.425062	8.035039	32.4147
24	6	0	12.320947	10.521807	31.747746
25	6	0	12.369262	14.924771	32.924817
26	1	0	13.539059	15.987225	34.276226
27	6	0	9.586232	15.232908	33.791464
28	1	0	8.39335	13.977755	32.643373
29	6	0	8.799612	17.974972	33.647661
30	8	0	10.035372	19.389554	29.441373
31	6	0	9.002639	19.647607	31.759115
32	1	0	8.215883	21.544458	31.980819

33	6	0	7.604404	18.79193	36.104624
34	1	0	8.153263	20.73553	36.642189
35	6	0	8.742704	16.866771	37.981857
36	6	0	12.83929	15.792874	30.261568
37	1	0	12.405486	17.888069	26.897832
38	6	0	11.797544	17.613347	28.849594
39	1	0	14.295456	14.704954	29.284701
40	6	0	15.574247	5.389433	35.457777
41	6	0	15.873653	6.530421	39.759809
42	6	0	13.426766	7.466507	39.946382
43	1	0	12.044689	7.064268	38.479758
44	6	0	12.758187	9.069531	41.940986
45	6	0	10.156923	10.250359	42.048929
46	1	0	9.700051	10.58001	44.038785
47	1	0	8.729498	8.950483	41.275616
48	6	0	9.837867	12.77384	40.626772
49	6	0	9.537531	12.400798	37.725495
50	6	0	6.782482	13.979643	44.161076
51	1	0	5.376452	15.474802	44.436741
52	1	0	8.309424	14.217076	45.556777
53	1	0	5.872159	12.133206	44.429186
54	6	0	7.095816	16.393361	40.287018
55	6	0	14.564981	9.590509	43.814305
56	1	0	18.417318	8.99321	45.114145
57	6	0	17.003494	8.587872	43.667789
58	1	0	14.072694	10.812067	45.404551
59	6	0	17.696178	7.069391	41.620238
60	6	0	14.880445	7.766291	34.607054
61	8	0	5.443811	17.871914	40.946741

**3b**000017_en_		Standard Orientation (A.U.)			
Center number	Atomic number	Atomic Type	X	Y	Z
0	16	0	13.045777	13.778387	41.541643
1	16	0	13.25506	17.49978	40.012797
2	7	0	8.006751	15.437444	41.691536
3	8	0	17.604986	2.204349	36.301727
4	8	0	12.0574	12.072272	32.71563
5	8	0	9.264577	21.834717	36.870157
6	1	0	7.962645	21.540575	38.15462
7	8	0	15.629308	12.612947	30.462691
8	8	0	13.227047	4.123771	37.694818
9	1	0	11.877479	8.409496	35.341097
10	8	0	8.369792	11.041416	37.098277
11	8	0	16.754828	3.689134	41.477238
12	1	0	16.628308	2.850549	39.836141
13	7	0	9.925265	15.046362	36.789824
14	6	0	19.990206	1.120966	35.751623
15	1	0	20.193074	-0.515641	37.009343

16	1	0	21.53983	2.46805	36.11654
17	1	0	20.086747	0.478903	33.770909
18	6	0	16.871517	4.320062	35.045409
19	6	0	18.285002	5.527675	33.157575
20	1	0	18.525524	8.778373	30.682438
21	6	0	17.413586	7.783156	32.105564
22	1	0	20.090156	4.749641	32.543953
23	6	0	15.11762	8.835462	32.893286
24	6	0	14.352326	11.333918	31.863727
25	6	0	11.306684	14.61901	32.186395
26	1	0	12.992005	15.823014	32.398154
27	6	0	9.278683	15.40126	34.119043
28	1	0	7.568434	14.287092	33.724817
29	6	0	8.875541	18.197124	33.928686
30	8	0	7.117276	18.329529	29.754138
31	6	0	7.853931	19.411986	31.971108
32	1	0	7.524514	21.449747	32.003354
33	6	0	10.174945	19.495037	36.141282
34	1	0	12.168998	19.762315	35.564184
35	6	0	10.22302	17.371006	38.189883
36	6	0	10.194288	14.820366	29.575431
37	1	0	7.675226	16.088216	26.753346
38	6	0	8.353936	16.329003	28.687974
39	1	0	10.932619	13.462664	28.208874
40	6	0	14.534356	5.361195	35.802936
41	6	0	12.919397	5.525692	39.893565
42	6	0	10.915085	7.186333	40.166658
43	1	0	9.514816	7.32407	38.670094
44	6	0	10.760356	8.76672	42.286195
45	6	0	8.794732	10.837453	42.438248
46	1	0	8.523066	11.341751	44.427938
47	1	0	6.968398	10.195317	41.677919
48	6	0	9.517497	13.257322	40.981025
49	6	0	9.229954	12.924279	38.0907
50	6	0	6.707109	15.606363	44.11602
51	1	0	5.205542	14.180911	44.278752
52	1	0	5.859655	17.492938	44.222182
53	1	0	8.059335	15.374636	45.68207
54	6	0	8.207516	17.584047	40.25063
55	6	0	12.589156	8.486825	44.190187
56	1	0	15.990245	6.550828	45.432847
57	6	0	14.563992	6.751443	43.955918
58	1	0	12.490778	9.66926	45.881081
59	6	0	14.793457	5.279212	41.76844
60	6	0	13.662961	7.590247	34.729642
61	8	0	6.970868	19.504429	40.631641

**3b**000018_en_		Standard Orientation (A.U.)			
Center	Atomic	Atomic	X	Y	Z

number	number	Type			
0	16	0	13.041684	13.784311	41.545415
1	16	0	13.247793	17.505346	40.015234
2	7	0	8.000477	15.437122	41.690234
3	8	0	17.622046	2.215621	36.314405
4	8	0	12.06397	12.074225	32.719286
5	8	0	9.25449	21.834248	36.86762
6	1	0	7.952008	21.538955	38.151267
7	8	0	15.637109	12.619048	30.469288
8	8	0	13.240088	4.129288	37.702498
9	1	0	11.886797	8.412396	35.345998
10	8	0	8.37336	11.039763	37.098837
11	8	0	16.764022	3.700075	41.488897
12	1	0	16.640457	2.861128	39.84776
13	7	0	9.923711	15.046701	36.790364
14	6	0	20.009213	1.135253	35.766845
15	1	0	20.213164	-0.500611	37.025355
16	1	0	21.556706	2.48455	36.132647
17	1	0	20.108395	0.492588	33.786456
18	6	0	16.886818	4.329823	35.056588
19	6	0	18.300392	5.53868	33.169621
20	1	0	18.538647	8.788713	30.693388
21	6	0	17.42679	7.792505	32.115883
22	1	0	20.107216	4.762939	32.558015
23	6	0	15.128617	8.841886	32.901056
24	6	0	14.360711	11.338801	31.86974
25	6	0	11.310102	14.619664	32.188372
26	1	0	12.993509	15.82614	32.401254
27	6	0	9.279203	15.399815	34.118853
28	1	0	7.570814	14.283233	33.723406
29	6	0	8.872626	18.19509	33.927124
30	8	0	7.1183	18.323925	29.75077
31	6	0	7.85141	19.408004	31.968125
32	1	0	7.519417	21.445362	31.999367
33	6	0	10.168433	19.49544	36.140419
34	1	0	12.162654	19.764999	35.56496
35	6	0	10.217414	17.372206	38.189838
36	6	0	10.199761	14.818458	29.57634
37	1	0	7.681565	16.081909	26.7515
38	6	0	8.358308	16.324407	28.686603
39	1	0	10.940916	13.461002	28.211067
40	6	0	14.547476	5.367952	35.811504
41	6	0	12.928082	5.531392	39.9005
42	6	0	10.921308	7.189487	40.170916
43	1	0	9.522542	7.325087	38.672766
44	6	0	10.762127	8.770149	42.289929
45	6	0	8.793622	10.838355	42.439308
46	1	0	8.519433	11.34305	44.428549
47	1	0	6.96885	10.193496	41.677524

48	6	0	9.514571	13.258621	40.981826
49	6	0	9.230052	12.92416	38.091363
50	6	0	6.698388	15.605199	44.11347
51	1	0	5.1984	14.177973	44.2752
52	1	0	5.848568	17.490784	44.21831
53	1	0	8.049435	15.375529	45.680833
54	6	0	8.199993	17.583533	40.248873
55	6	0	12.589119	8.493033	44.196064
56	1	0	15.991255	6.561673	45.443073
57	6	0	14.566443	6.760121	43.964463
58	1	0	12.487285	9.675736	45.886565
59	6	0	14.800307	5.287718	41.777569
60	6	0	13.673981	7.595371	34.736544
61	8	0	6.960909	19.50266	40.62833

Table S13. Gibbs free energies[a] and equilibrium populations[b] of low-energy conformers of 11a.

Conformers	ΔG(a.u.)	P(%)/100	G(a.u.)
**11a**000001.log	0.00095	12.62	-3232.63687
**11a**000002.log	0.00266	2.06	-3232.635158
**11a**000003.log	0.0	34.53	-3232.637821
**11a**000004.log	0.00095	12.58	-3232.636868
**11a**000005.log	0.00095	12.58	-3232.636868
**11a**000006.log	0.00095	12.61	-3232.63687
**11a**000007.log	0.00426	0.38	-3232.633559
**11a**000008.log	0.00095	12.64	-3232.636872

[a]wB97M-V/def2-TZVP, in a.u.

[b]From ΔG values at 298.15 K.

Table S14. Cartesian coordinates for the low-energy reoptimized random research conformers of 11a at B3LYP-D3(BJ)/6-31G* level of theory in methanol.

**11a**000001_en_		Standard Orientation (A.U.)			
Center number	Atomic number	Atomic Type	X	Y	Z
0	7	0	-1.80614	1.589058	-0.047716
1	6	0	-1.537324	-0.652997	-1.274575
2	6	0	-2.297463	-3.081833	0.147038
3	7	0	-3.890386	-2.686454	2.310032
4	6	0	-4.595105	-0.366365	3.20674
5	6	0	-3.099648	1.909334	2.307912
6	6	0	-4.747347	4.261331	1.878363
7	6	0	-3.232968	5.757698	-0.03045
8	6	0	-1.405664	4.0145	-1.357746
9	6	0	-3.447625	8.25757	-0.284978
10	8	0	-2.203491	9.860169	-1.875609
11	6	0	0.058965	9.323915	-3.011146
12	6	0	1.630532	7.371161	-2.710518
13	6	0	1.318325	5.043817	-1.123325
14	8	0	-6.256269	-0.087649	4.785396
15	8	0	-0.735804	-0.795986	-3.439707
16	6	0	6.596567	0.567745	-1.43884
17	6	0	5.533228	-1.307003	-2.973246
18	6	0	6.862401	-3.529465	-3.441474
19	6	0	9.244995	-3.895099	-2.383544
20	6	0	10.305432	-2.005174	-0.834787
21	6	0	8.998275	0.20453	-0.363579
22	6	0	-3.47522	-4.944499	-1.786155
23	6	0	-6.114251	-4.179519	-2.602126
24	6	0	-6.556228	-2.031044	-4.090696
25	6	0	-9.012326	-1.333055	-4.742326
26	6	0	-11.069561	-2.777438	-3.944811
27	6	0	-10.649303	-4.948142	-2.512264
28	6	0	-8.191779	-5.636998	-1.853374
29	8	0	10.540584	-6.062386	-2.831449
30	6	0	-4.932168	-4.876078	3.646742
31	8	0	2.986705	3.100704	-2.001664
32	6	0	5.256141	2.927291	-0.806041
33	8	0	6.031615	4.543254	0.637281
34	8	0	12.662417	-2.575933	0.111053
35	1	0	-1.811116	3.706085	-3.35724
36	16	0	-0.834191	2.660497	5.035962
37	16	0	2.415249	0.693638	4.136242
38	16	0	1.501223	-3.177507	4.67115
39	16	0	0.884631	-4.575805	1.081956
40	1	0	-5.154132	5.278387	3.626971
41	1	0	-6.559236	3.615995	1.100139
42	1	0	-4.822566	9.317463	0.805667

43	1	0	0.535533	10.873258	-4.263195
44	1	0	3.359134	7.448118	-3.809478
45	1	0	1.752942	5.415347	0.857572
46	1	0	3.661371	-1.040666	-3.755517
47	1	0	6.070823	-5.015308	-4.61089
48	1	0	9.778463	1.674449	0.838748
49	1	0	-2.188742	-5.013082	-3.399656
50	1	0	-3.526988	-6.827701	-0.942897
51	1	0	-4.972065	-0.929506	-4.776955
52	1	0	-9.315151	0.337368	-5.897373
53	1	0	-12.980287	-2.228263	-4.455103
54	1	0	-12.229663	-6.109987	-1.906902
55	1	0	-7.880502	-7.345646	-0.756782
56	1	0	12.133659	-5.950465	-1.918925
57	1	0	-6.828754	-5.326798	2.969803
58	1	0	-3.675419	-6.484686	3.365546
59	1	0	-5.030381	-4.418215	5.651867
60	1	0	13.236846	-1.210019	1.186265

**11a**000002_en_		Standard Orientation (A.U.)			
Center number	Atomic number	Atomic Type	X	Y	Z
0	7	0	2.137554	0.997803	-0.196123
1	6	0	0.667867	-0.948774	0.608774
2	6	0	1.527386	-3.665535	-0.012363
3	7	0	4.051378	-3.912311	-0.997772
4	6	0	5.671738	-1.922012	-1.309211
5	6	0	4.503877	0.69223	-1.457674
6	6	0	6.166828	2.763163	-0.306664
7	6	0	4.272222	4.810206	0.316063
8	6	0	1.678304	3.647565	0.578269
9	6	0	4.936161	7.242841	0.405631
10	8	0	3.510555	9.335398	0.851507
11	6	0	0.940977	9.428596	0.557092
12	6	0	-0.69064	7.705907	-0.298325
13	6	0	-0.257378	5.029051	-1.13204
14	8	0	7.940772	-2.191168	-1.638916
15	8	0	-1.259568	-0.650121	1.838147
16	6	0	-6.224459	1.677389	0.250493
17	6	0	-6.417409	0.239887	-1.959622
18	6	0	-8.423991	-1.431025	-2.280464
19	6	0	-10.250145	-1.674507	-0.399388
20	6	0	-10.048412	-0.228209	1.825461
21	6	0	-8.055092	1.427824	2.152736
22	6	0	1.17037	-5.240198	2.440148
23	6	0	2.572469	-4.049799	4.638069
24	6	0	5.214431	-4.133438	4.804526
25	6	0	6.482198	-2.913892	6.768336
26	6	0	5.125496	-1.597335	8.605292

27	6	0	2.493587	-1.513135	8.466154
28	6	0	1.231645	-2.723711	6.496261
29	8	0	-12.211565	-3.302348	-0.70261
30	6	0	5.065602	-6.436147	-1.503291
31	8	0	-2.584015	3.649973	-1.339816
32	6	0	-4.097819	3.420993	0.730833
33	8	0	-3.743417	4.559001	2.69516
34	8	0	-11.952828	-0.626786	3.55704
35	1	0	0.93976	3.625333	2.504928
36	16	0	4.231732	1.325414	-5.036587
37	16	0	0.629772	0.118158	-5.928434
38	16	0	0.725611	-3.895714	-5.814809
39	16	0	-0.917617	-4.849723	-2.391285
40	1	0	7.686754	3.36624	-1.564617
41	1	0	7.043063	1.964008	1.396533
42	1	0	6.896113	7.797648	0.167712
43	1	0	0.298605	11.297312	1.094087
44	1	0	-2.637799	8.333453	-0.380446
45	1	0	0.464294	4.967023	-3.06362
46	1	0	-4.97909	0.411658	-3.403633
47	1	0	-8.600796	-2.574592	-3.972578
48	1	0	-7.856264	2.537831	3.86853
49	1	0	-0.848251	-5.31712	2.857158
50	1	0	1.812928	-7.169448	2.077934
51	1	0	6.310092	-5.147281	3.401091
52	1	0	8.53122	-3.000523	6.858922
53	1	0	6.111632	-0.645308	10.133439
54	1	0	1.417265	-0.490244	9.883421
55	1	0	-0.809305	-2.597993	6.356133
56	1	0	-13.252343	-3.194881	0.809764
57	1	0	6.445028	-6.276369	-3.022376
58	1	0	6.001052	-7.246471	0.15358
59	1	0	3.519015	-7.666399	-2.087121
60	1	0	-11.661816	0.418313	5.031577

**11a**000003_en_		Standard Orientation (A.U.)			
Center number	Atomic number	Atomic Type	X	Y	Z
0	7	0	-2.110298	1.151274	0.168622
1	6	0	-1.530236	-1.015169	-1.089224
2	6	0	-1.949235	-3.543109	0.298625
3	7	0	-3.465778	-3.40448	2.553512
4	6	0	-4.513428	-1.218751	3.457014
5	6	0	-3.352494	1.247341	2.567045
6	6	0	-5.29322	3.382618	2.260984
7	6	0	-4.062032	5.094164	0.329862
8	6	0	-2.072138	3.629899	-1.09884
9	6	0	-4.615798	7.54711	0.140012
10	8	0	-3.657113	9.329443	-1.456863

11	6	0	-1.380155	9.122124	-2.666874
12	6	0	0.445925	7.392113	-2.453008
13	6	0	0.496559	5.01075	-0.915644
14	8	0	-6.196076	-1.196024	5.037963
15	8	0	-0.724302	-1.032629	-3.251742
16	6	0	6.331193	1.316871	-1.465088
17	6	0	5.490507	-0.670428	-2.995752
18	6	0	7.100195	-2.681539	-3.535107
19	6	0	9.5423	-2.723808	-2.55362
20	6	0	10.378408	-0.721476	-1.00903
21	6	0	8.792317	1.279384	-0.466073
22	6	0	-3.080092	-5.418932	-1.650543
23	6	0	-5.449624	-4.37201	-2.876621
24	6	0	-5.389576	-3.614016	-5.411761
25	6	0	-7.514732	-2.565592	-6.561236
26	6	0	-9.73994	-2.238701	-5.184827
27	6	0	-9.82379	-2.981577	-2.657359
28	6	0	-7.698443	-4.0447	-1.514817
29	8	0	11.111086	-4.685814	-3.071018
30	6	0	-4.109688	-5.74051	3.889866
31	8	0	2.391391	3.337099	-1.886308
32	6	0	4.694112	3.456507	-0.750977
33	8	0	5.27823	5.135822	0.710657
34	8	0	12.820172	-0.968979	-0.141182
35	1	0	-2.504413	3.305689	-3.09046
36	16	0	-1.08645	2.174023	5.25484
37	16	0	2.346174	0.661447	4.168908
38	16	0	2.004067	-3.308295	4.639376
39	16	0	1.389686	-4.71791	1.044071
40	1	0	-5.750604	4.308794	4.047267
41	1	0	-7.040165	2.527151	1.539016
42	1	0	-6.076928	8.394068	1.302515
43	1	0	-1.15265	10.743235	-3.897953
44	1	0	2.114554	7.72108	-3.597131
45	1	0	0.933747	5.391064	1.063081
46	1	0	3.57578	-0.657972	-3.716911
47	1	0	6.485614	-4.251479	-4.701143
48	1	0	9.396929	2.829499	0.736732
49	1	0	-1.650333	-5.76777	-3.096192
50	1	0	-3.445153	-7.211544	-0.691716
51	1	0	-3.643954	-3.807915	-6.469686
52	1	0	-7.425159	-1.990551	-8.529843
53	1	0	-11.395017	-1.412178	-6.074563
54	1	0	-11.546126	-2.743043	-1.565982
55	1	0	-7.815599	-4.607427	0.451282
56	1	0	12.700437	-4.363256	-2.203289
57	1	0	-4.413129	-5.277117	5.871577
58	1	0	-5.830086	-6.614003	3.144617
59	1	0	-2.538377	-7.062302	3.710333

60	1	0	13.229057	0.447474	0.944295

**11a**000004_en_		Standard Orientation (A.U.)			
Center number	Atomic number	Atomic Type	X	Y	Z
0	7	0	-1.806116	1.589203	-0.047744
1	6	0	-1.537381	-0.652951	-1.274501
2	6	0	-2.297582	-3.081716	0.147227
3	7	0	-3.890548	-2.686099	2.310201
4	6	0	-4.594749	-0.365932	3.207142
5	6	0	-3.099252	1.909648	2.308041
6	6	0	-4.746939	4.261713	1.878589
7	6	0	-3.232645	5.757919	-0.030455
8	6	0	-1.405612	4.014561	-1.357913
9	6	0	-3.446979	8.25786	-0.284746
10	8	0	-2.202928	9.860462	-1.875427
11	6	0	0.059007	9.323848	-3.011843
12	6	0	1.63042	7.370909	-2.711601
13	6	0	1.318447	5.043859	-1.123896
14	8	0	-6.255633	-0.087073	4.786059
15	8	0	-0.736094	-0.796006	-3.439713
16	6	0	6.596319	0.567364	-1.438893
17	6	0	5.532884	-1.307448	-2.973155
18	6	0	6.862008	-3.529954	-3.441327
19	6	0	9.244647	-3.895579	-2.383487
20	6	0	10.305165	-2.005601	-0.834842
21	6	0	8.998074	0.204157	-0.363716
22	6	0	-3.475361	-4.944416	-1.785946
23	6	0	-6.114444	-4.179559	-2.601927
24	6	0	-6.556522	-2.031073	-4.090414
25	6	0	-9.012663	-1.333236	-4.742131
26	6	0	-11.069806	-2.777813	-3.944795
27	6	0	-10.649439	-4.948562	-2.512302
28	6	0	-8.191909	-5.637249	-1.853326
29	8	0	10.540184	-6.062893	-2.831355
30	6	0	-4.932524	-4.875611	3.646939
31	8	0	2.986701	3.100578	-2.002044
32	6	0	5.256052	2.927048	-0.806254
33	8	0	6.031582	4.543067	0.636962
34	8	0	12.662162	-2.576389	0.110944
35	1	0	-1.811244	3.706093	-3.357356
36	16	0	-0.833461	2.660978	5.035769
37	16	0	2.415669	0.693837	4.136024
38	16	0	1.5011	-3.177317	4.671298
39	16	0	0.884352	-4.575863	1.082285
40	1	0	-5.153428	5.278864	3.627213
41	1	0	-6.558949	3.616468	1.100602
42	1	0	-4.8216	9.317833	0.806227
43	1	0	0.53525	10.873006	-4.264244

44	1	0	3.35853	7.447377	-3.811373
45	1	0	1.753243	5.415781	0.856883
46	1	0	3.660959	-1.041164	-3.755293
47	1	0	6.070357	-5.015828	-4.610661
48	1	0	9.77837	1.674147	0.838462
49	1	0	-2.188901	-5.012875	-3.399471
50	1	0	-3.527029	-6.827654	-0.94276
51	1	0	-4.972412	-0.929317	-4.776464
52	1	0	-9.315545	0.337233	-5.897103
53	1	0	-12.980554	-2.228759	-4.455145
54	1	0	-12.229742	-6.110567	-1.907089
55	1	0	-7.880526	-7.345941	-0.756829
56	1	0	12.133301	-5.95095	-1.918903
57	1	0	-3.676262	-6.484497	3.365171
58	1	0	-5.03008	-4.418012	5.652161
59	1	0	-6.829397	-5.325759	2.970436
60	1	0	13.236657	-1.210463	1.186113

**11a**000005_en_		Standard Orientation (A.U.)			
Center number	Atomic number	Atomic Type	X	Y	Z
0	7	0	-1.806401	1.588718	-0.047902
1	6	0	-1.537435	-0.65315	-1.275059
2	6	0	-2.297111	-3.08228	0.146314
3	7	0	-3.889631	-2.68732	2.309722
4	6	0	-4.594953	-0.367418	3.206409
5	6	0	-3.09973	1.908532	2.307877
6	6	0	-4.74762	4.26048	1.878737
7	6	0	-3.233185	5.757394	-0.029615
8	6	0	-1.406251	4.014385	-1.357677
9	6	0	-3.447132	8.25754	-0.282295
10	8	0	-2.203048	9.861183	-1.871892
11	6	0	0.0579	9.324397	-3.010225
12	6	0	1.629157	7.371131	-2.711426
13	6	0	1.317727	5.043994	-1.123763
14	8	0	-6.256202	-0.089189	4.785061
15	8	0	-0.736064	-0.795787	-3.440276
16	6	0	6.596298	0.568323	-1.438663
17	6	0	5.533093	-1.306631	-2.972889
18	6	0	6.862477	-3.529028	-3.440949
19	6	0	9.245155	-3.894267	-2.383143
20	6	0	10.30552	-2.004124	-0.834538
21	6	0	8.998103	0.205446	-0.363466
22	6	0	-3.475231	-4.944493	-1.787095
23	6	0	-6.11436	-4.179127	-2.602375
24	6	0	-6.556327	-2.030618	-4.090878
25	6	0	-9.012491	-1.332138	-4.74179
26	6	0	-11.069782	-2.776063	-3.943605
27	6	0	-10.649544	-4.946806	-2.51109

28	6	0	-8.191972	-5.636143	-1.852912
29	8	0	10.541069	-6.061416	-2.830973
30	6	0	-4.931206	-4.87708	3.646377
31	8	0	2.986391	3.101122	-2.001975
32	6	0	5.255662	2.927763	-0.805923
33	8	0	6.030814	4.543722	0.637555
34	8	0	12.662556	-2.574615	0.11125
35	1	0	-1.81215	3.706208	-3.357113
36	16	0	-0.834119	2.659365	5.035822
37	16	0	2.415515	0.692817	4.135408
38	16	0	1.502196	-3.178341	4.669841
39	16	0	0.885047	-4.576321	1.080382
40	1	0	-5.15464	5.277128	3.627524
41	1	0	-6.559358	3.61508	1.100215
42	1	0	-4.821588	9.316902	0.809473
43	1	0	0.533511	10.873707	-4.262677
44	1	0	3.356389	7.447228	-3.812607
45	1	0	1.752463	5.415979	0.857019
46	1	0	3.661173	-1.040594	-3.755114
47	1	0	6.070955	-5.015115	-4.610089
48	1	0	9.778124	1.675574	0.83872
49	1	0	-2.189053	-5.012706	-3.40085
50	1	0	-3.52691	-6.827899	-0.944299
51	1	0	-4.972124	-0.929433	-4.77763
52	1	0	-9.31531	0.338311	-5.896803
53	1	0	-12.980545	-2.226506	-4.453345
54	1	0	-12.229959	-6.108309	-1.905214
55	1	0	-7.880714	-7.3448	-0.756333
56	1	0	12.133873	-5.94935	-1.917958
57	1	0	-5.02697	-4.420366	5.651899
58	1	0	-6.828792	-5.326315	2.97128
59	1	0	-3.67566	-6.486222	3.362963
60	1	0	13.236567	-1.208885	1.186912

**11a**000006_en_		Standard Orientation (A.U.)			
Center number	Atomic number	Atomic Type	X	Y	Z
0	7	0	-1.806524	1.588851	-0.048059
1	6	0	-1.537575	-0.653087	-1.275136
2	6	0	-2.29742	-3.082108	0.146266
3	7	0	-3.890022	-2.687008	2.309601
4	6	0	-4.595091	-0.367025	3.20634
5	6	0	-3.099719	1.90878	2.307714
6	6	0	-4.747346	4.260916	1.878699
7	6	0	-3.232824	5.757655	-0.029722
8	6	0	-1.406062	4.014462	-1.357789
9	6	0	-3.446488	8.257821	-0.282447
10	8	0	-2.20225	9.861265	-1.872115
11	6	0	0.058629	9.324259	-3.01044

12	6	0	1.629713	7.370856	-2.711583
13	6	0	1.318043	5.043821	-1.123812
14	8	0	-6.256288	-0.088633	4.785006
15	8	0	-0.736221	-0.79585	-3.440341
16	6	0	6.59648	0.568001	-1.438564
17	6	0	5.5333	-1.30697	-2.97281
18	6	0	6.862679	-3.529347	-3.440849
19	6	0	9.245309	-3.894718	-2.382908
20	6	0	10.305592	-2.004539	-0.834394
21	6	0	8.998253	0.205095	-0.363344
22	6	0	-3.475569	-4.944248	-1.787152
23	6	0	-6.114757	-4.178908	-2.602289
24	6	0	-8.19219	-5.636421	-1.853288
25	6	0	-10.649849	-4.947216	-2.511276
26	6	0	-11.070366	-2.776077	-3.943104
27	6	0	-9.013256	-1.331635	-4.740814
28	6	0	-6.557003	-2.030003	-4.090123
29	8	0	10.541005	-6.061993	-2.830594
30	6	0	-4.931791	-4.876711	3.646194
31	8	0	2.986564	3.10078	-2.001853
32	6	0	5.255911	2.927503	-0.805967
33	8	0	6.031268	4.543614	0.637234
34	8	0	12.662647	-2.575021	0.111354
35	1	0	-1.811961	3.706353	-3.357234
36	16	0	-0.833948	2.659492	5.035723
37	16	0	2.415537	0.692684	4.135266
38	16	0	1.502083	-3.178206	4.669796
39	16	0	0.884711	-4.576327	1.080389
40	1	0	-5.154171	5.277599	3.62751
41	1	0	-6.559206	3.615751	1.100256
42	1	0	-4.820791	9.317379	0.809323
43	1	0	0.534368	10.873487	-4.262943
44	1	0	3.356942	7.446735	-3.812784
45	1	0	1.752732	5.415874	0.856963
46	1	0	3.661392	-1.040883	-3.755041
47	1	0	6.071208	-5.015374	-4.610107
48	1	0	9.778368	1.675198	0.838824
49	1	0	-2.189445	-5.012399	-3.400964
50	1	0	-3.5272	-6.827691	-0.94444
51	1	0	-7.880723	-7.345358	-0.7572
52	1	0	-12.230101	-6.109153	-1.905807
53	1	0	-12.981192	-2.226585	-4.452682
54	1	0	-9.316289	0.339188	-5.895229
55	1	0	-4.972959	-0.928378	-4.776539
56	1	0	12.134155	-5.950002	-1.918246
57	1	0	-6.829048	-5.326306	2.970407
58	1	0	-3.675935	-6.485738	3.3635
59	1	0	-5.028393	-4.419663	5.651596
60	1	0	13.236524	-1.209444	1.187319

**11a**000007_en_		Standard Orientation (A.U.)			
Center number	Atomic number	Atomic Type	X	Y	Z
0	7	0	-1.807663	1.507968	-0.133595
1	6	0	-0.695747	-0.779954	-0.492885
2	6	0	-1.766451	-3.0708	0.955419
3	7	0	-4.259128	-2.7199	1.967173
4	6	0	-5.594707	-0.510612	1.819514
5	6	0	-4.135339	1.877013	1.191242
6	6	0	-5.600805	3.711105	-0.509507
7	6	0	-3.509018	5.263191	-1.684329
8	6	0	-1.088653	3.753406	-1.642862
9	6	0	-3.876391	7.648027	-2.422587
10	8	0	-2.231446	9.342801	-3.437549
11	6	0	0.345068	9.163502	-3.258407
12	6	0	1.808341	7.535348	-2.005246
13	6	0	1.093371	5.285708	-0.433464
14	8	0	-7.844837	-0.375022	2.315965
15	8	0	1.099153	-1.080929	-1.916333
16	6	0	6.448894	0.837311	-0.917766
17	6	0	6.588872	0.079906	1.61105
18	6	0	8.342957	-1.735328	2.35332
19	6	0	9.964338	-2.80466	0.575887
20	6	0	9.816212	-2.040795	-1.969674
21	6	0	8.076095	-0.240961	-2.71333
22	6	0	-1.573933	-5.4217	-0.785905
23	6	0	-3.493466	-5.385202	-2.914541
24	6	0	-5.505487	-7.10392	-2.895967
25	6	0	-7.339366	-7.075391	-4.78914
26	6	0	-7.185562	-5.317171	-6.745412
27	6	0	-5.165231	-3.623433	-6.810309
28	6	0	-3.327233	-3.661773	-4.922154
29	8	0	11.674314	-4.581137	1.287327
30	6	0	-5.565794	-4.854913	3.149193
31	8	0	3.224266	3.700875	0.119379
32	6	0	4.577939	2.695858	-1.829545
33	8	0	4.280601	3.303004	-4.025401
34	8	0	11.503743	-3.227617	-3.558714
35	1	0	-0.457612	3.098255	-3.494988
36	16	0	-3.661629	3.524723	4.389952
37	16	0	-0.195403	2.221156	5.613427
38	16	0	-0.770412	-1.630895	6.596276
39	16	0	0.678712	-3.650242	3.590839
40	1	0	-7.005075	4.815425	0.522268
41	1	0	-6.613275	2.568732	-1.915959
42	1	0	-5.741323	8.494779	-2.321501
43	1	0	1.172357	10.709453	-4.316006
44	1	0	3.819144	7.885456	-2.163555

45	1	0	0.47998	5.873581	1.445907
46	1	0	5.30372	0.891666	2.979373
47	1	0	8.472854	-2.360781	4.301139
48	1	0	7.918204	0.350115	-4.673085
49	1	0	0.353865	-5.451953	-1.524727
50	1	0	-1.836988	-7.118186	0.359586
51	1	0	-5.626745	-8.49713	-1.391478
52	1	0	-8.882676	-8.427801	-4.732667
53	1	0	-8.614054	-5.281964	-8.218926
54	1	0	-5.002904	-2.274853	-8.350074
55	1	0	-1.735213	-2.378148	-5.026891
56	1	0	12.595734	-5.090453	-0.220673
57	1	0	-4.160192	-6.179727	3.866078
58	1	0	-6.695215	-4.132137	4.712133
59	1	0	-6.81261	-5.797805	1.802276
60	1	0	11.235508	-2.647233	-5.274132

**11a**000008_en_		Standard Orientation (A.U.)			
Center number	Atomic number	Atomic Type	X	Y	Z
0	7	0	-1.806187	1.588651	-0.047102
1	6	0	-1.537252	-0.652883	-1.274841
2	6	0	-2.297036	-3.082469	0.145853
3	7	0	-3.889497	-2.688042	2.30939
4	6	0	-4.593952	-0.368363	3.20742
5	6	0	-3.098711	1.907861	2.309318
6	6	0	-4.746871	4.259908	1.881392
7	6	0	-3.233979	5.756893	-0.028158
8	6	0	-1.406388	4.014641	-1.356239
9	6	0	-3.450182	8.256565	-0.2833
10	8	0	-2.207414	9.859122	-1.875044
11	6	0	0.056192	9.324576	-3.009043
12	6	0	1.628814	7.372727	-2.707912
13	6	0	1.317311	5.044613	-1.121658
14	8	0	-6.254617	-0.090493	4.786724
15	8	0	-0.735928	-0.794951	-3.440098
16	6	0	6.596269	0.569433	-1.438753
17	6	0	5.533117	-1.305185	-2.97345
18	6	0	6.862604	-3.527361	-3.442191
19	6	0	9.245355	-3.892806	-2.384533
20	6	0	10.305596	-2.003008	-0.835486
21	6	0	8.99811	0.206386	-0.363726
22	6	0	-3.475426	-4.94397	-1.788194
23	6	0	-6.114601	-4.178328	-2.603035
24	6	0	-6.556715	-2.029603	-4.09124
25	6	0	-9.012923	-1.331034	-4.741762
26	6	0	-11.070156	-2.775078	-3.943547
27	6	0	-10.649795	-4.946012	-2.511402
28	6	0	-8.19212	-5.63545	-1.853585

29	8	0	10.541267	-6.059793	-2.832962
30	6	0	-4.930967	-4.878136	3.645636
31	8	0	2.9862	3.102308	-2.001106
32	6	0	5.255466	2.928593	-0.805271
33	8	0	6.030603	4.544052	0.638845
34	8	0	12.662709	-2.57365	0.110105
35	1	0	-1.811846	3.706898	-3.355829
36	16	0	-0.832368	2.657816	5.036586
37	16	0	2.416601	0.691133	4.13512
38	16	0	1.50256	-3.18022	4.668795
39	16	0	0.884936	-4.577119	1.079322
40	1	0	-5.152451	5.27654	3.630536
41	1	0	-6.559267	3.61469	1.104314
42	1	0	-4.82529	9.316082	0.807509
43	1	0	0.532585	10.874465	-4.260482
44	1	0	3.358216	7.451336	-3.80551
45	1	0	1.752221	5.415363	0.859301
46	1	0	3.661152	-1.039012	-3.755529
47	1	0	6.071145	-5.013115	-4.611809
48	1	0	9.778145	1.676145	0.8389
49	1	0	-2.189274	-5.011721	-3.401981
50	1	0	-3.527107	-6.827662	-0.946012
51	1	0	-4.972535	-0.928311	-4.777868
52	1	0	-9.315917	0.339621	-5.896438
53	1	0	-12.980967	-2.225436	-4.45304
54	1	0	-12.230142	-6.107651	-1.905597
55	1	0	-7.880754	-7.344274	-0.757298
56	1	0	12.134395	-5.947789	-1.920524
57	1	0	-5.026296	-4.421974	5.6513
58	1	0	-6.828694	-5.32707	2.970736
59	1	0	-3.675581	-6.487283	3.3615
60	1	0	13.237252	-1.207648	1.185158

Table S15. Gibbs free energies[a] and equilibrium populations[b] of low-energy conformers of 11b.

Conformers	ΔG(a.u.)	P(%)/100	G(a.u.)
**11b**000001.log	0.00198	3.83	-3232.624956
**11b**000002.log	0.00823	0.01	-3232.618708
**11b**000003.log	0.0	31.16	-3232.626936
**11b**000004.log	1e-05	30.8	-3232.626925
**11b**000005.log	0.00199	3.78	-3232.624945
**11b**000006.log	0.00795	0.01	-3232.618985
**11b**000007.log	2e-05	30.42	-3232.626914

[a]wB97M-V/def2-TZVP, in a.u.
[b]From ΔG values at 298.15 K.

Table S16. Cartesian coordinates for the low-energy reoptimized random research conformers of 11b at B3LYP-D3(BJ)/6-31G* level of theory in methanol.

**11b**000001_en_		Standard Orientation (A.U.)			
Center number	Atomic number	Atomic Type	X	Y	Z
0	7	0	-2.812537	0.886511	-0.015974
1	6	0	-1.399747	-0.662534	-1.539411
2	6	0	-0.71147	-3.263547	-0.389617
3	7	0	-1.82966	-3.776957	2.068897
4	6	0	-3.686723	-2.367546	3.206546
5	6	0	-4.672994	-0.138464	1.714797
6	6	0	-5.435293	2.190203	3.252164
7	6	0	-5.397102	4.215775	1.239174
8	6	0	-3.417138	3.524668	-0.698036
9	6	0	-6.735735	6.346896	1.293924
10	8	0	-6.555996	8.337333	-0.342819
11	6	0	-4.32869	9.023829	-1.477886
12	6	0	-2.043375	7.942673	-1.436556
13	6	0	-1.233945	5.447365	-0.350582
14	8	0	-4.557529	-2.838721	5.292782
15	8	0	-0.579244	-0.048128	-3.592757
16	6	0	5.174789	3.076815	-1.471524
17	6	0	4.896733	1.981802	-3.867047
18	6	0	6.739038	0.347435	-4.792705
19	6	0	8.832142	-0.252363	-3.302766
20	6	0	9.10834	0.864609	-0.904034
21	6	0	7.333156	2.556919	-0.020815
22	6	0	2.216751	-3.525268	-0.350401
23	6	0	3.621003	-2.452566	1.900395
24	6	0	2.786863	-0.368642	3.293519
25	6	0	4.226318	0.592587	5.280344
26	6	0	6.525987	-0.525719	5.920932
27	6	0	7.375281	-2.620499	4.553991
28	6	0	5.929982	-3.567383	2.563132
29	8	0	10.574865	-1.937512	-4.124987
30	6	0	-0.982745	-6.044471	3.413093
31	8	0	0.999336	4.610385	-1.63515
32	6	0	3.173926	4.58126	-0.250948
33	8	0	3.356017	5.583653	1.813405
34	8	0	11.202416	0.103325	0.456688
35	1	0	-4.090994	3.597581	-2.64805
36	16	0	-7.770423	-1.004384	0.042808
37	16	0	-7.523179	-4.758431	-0.986149
38	16	0	-5.082761	-4.72508	-4.156391
39	16	0	-1.615434	-5.83039	-2.725826
40	1	0	-7.233767	1.962583	4.23036
41	1	0	-3.968151	2.527185	4.683354
42	1	0	-8.188025	6.695677	2.69889

43	1	0	-4.632226	10.788627	-2.472446
44	1	0	-0.572148	8.958673	-2.440109
45	1	0	-0.762612	5.585233	1.654288
46	1	0	3.208591	2.34976	-4.959104
47	1	0	6.548065	-0.540299	-6.630503
48	1	0	7.510121	3.391352	1.840577
49	1	0	2.69723	-5.534923	-0.499868
50	1	0	2.886061	-2.641809	-2.096266
51	1	0	1.022644	0.54803	2.81263
52	1	0	3.559179	2.244891	6.29374
53	1	0	7.633353	0.211688	7.484507
54	1	0	9.135464	-3.550323	5.064934
55	1	0	6.602181	-5.194281	1.503778
56	1	0	11.801071	-2.12645	-2.761974
57	1	0	-0.882795	-7.623165	2.08838
58	1	0	0.86619	-5.730939	4.280121
59	1	0	-2.36574	-6.452328	4.878844
60	1	0	10.699398	-0.046777	2.216994

**11b**000002_en_		Standard Orientation (A.U.)			
Center number	Atomic number	Atomic Type	X	Y	Z
0	7	0	3.420994	0.328466	-0.165187
1	6	0	1.415655	-0.266148	1.354236
2	6	0	-0.359477	-2.354923	0.283602
3	7	0	0.626825	-3.650035	-1.945945
4	6	0	2.992023	-3.352637	-2.953316
5	6	0	4.763281	-1.625558	-1.52963
6	6	0	6.641453	-0.113206	-3.124679
7	6	0	7.283073	2.04283	-1.364447
8	6	0	5.018875	2.562751	0.288082
9	6	0	9.398842	3.40383	-1.431847
10	8	0	9.907021	5.542982	-0.069994
11	6	0	8.077957	7.260034	0.578747
12	6	0	5.567443	7.21433	0.272197
13	6	0	3.894794	5.115906	-0.6421
14	8	0	3.689741	-4.449746	-4.863002
15	8	0	0.838667	0.916977	3.236104
16	6	0	-3.031046	5.205913	0.041792
17	6	0	-3.260898	5.489458	2.663369
18	6	0	-5.570083	5.055681	3.846362
19	6	0	-7.643251	4.259243	2.422144
20	6	0	-7.416697	3.992001	-0.218929
21	6	0	-5.154514	4.522192	-1.404148
22	6	0	-2.783321	-0.794433	-0.330322
23	6	0	-5.252774	-2.007926	-1.071752
24	6	0	-6.880649	-3.025258	0.750528
25	6	0	-9.267691	-3.919152	0.076401
26	6	0	-10.085432	-3.774281	-2.426612

27	6	0	-8.493347	-2.733173	-4.258766
28	6	0	-6.097782	-1.856417	-3.578578
29	8	0	-9.859785	3.679925	3.551622
30	6	0	-0.945474	-5.550616	-3.214885
31	8	0	1.406303	5.580568	0.349859
32	6	0	-0.562434	5.154613	-1.250302
33	8	0	-0.285201	4.626282	-3.478636
34	8	0	-9.559591	3.137221	-1.442589
35	1	0	5.461075	2.710604	2.298914
36	16	0	6.888493	-3.582801	0.659593
37	16	0	4.781562	-6.667899	1.805907
38	16	0	2.325443	-5.242266	4.617135
39	16	0	-1.130331	-4.717426	2.808995
40	1	0	8.250898	-1.232005	-3.759374
41	1	0	5.638073	0.576581	-4.806247
42	1	0	10.999994	2.897725	-2.60843
43	1	0	8.996154	8.895183	1.403013
44	1	0	4.575258	8.898817	0.890725
45	1	0	3.719351	5.043438	-2.69548
46	1	0	-1.606993	5.946842	3.776499
47	1	0	-5.773643	5.22189	5.878983
48	1	0	-4.950722	4.27077	-3.427845
49	1	0	-3.115328	0.358775	1.344145
50	1	0	-2.19247	0.46645	-1.852944
51	1	0	-6.295072	-3.065654	2.716847
52	1	0	-10.503557	-4.697947	1.517996
53	1	0	-11.9515	-4.451418	-2.946268
54	1	0	-9.11097	-2.604544	-6.2126
55	1	0	-4.86654	-1.030624	-5.000543
56	1	0	-10.971315	3.07879	2.20583
57	1	0	-2.503738	-6.08064	-1.985672
58	1	0	-1.686228	-4.819964	-4.99923
59	1	0	0.219428	-7.204734	-3.612526
60	1	0	-9.097964	1.73492	-2.543719

**11b**000003_en_		Standard Orientation (A.U.)			
Center number	Atomic number	Atomic Type	X	Y	Z
0	7	0	-2.475425	1.117254	-0.178292
1	6	0	-1.567798	-1.196513	-0.892691
2	6	0	-2.646956	-3.489931	0.579764
3	7	0	-4.81981	-2.964924	2.192198
4	6	0	-5.879796	-0.635868	2.524334
5	6	0	-5.067798	1.372615	0.676348
6	6	0	-5.141133	4.071717	1.67586
7	6	0	-3.481334	5.401625	-0.217832
8	6	0	-1.538494	3.551427	-1.223495
9	6	0	-3.755593	7.817758	-0.863693
10	8	0	-2.226373	9.197392	-2.410347

11	6	0	0.323293	8.764454	-2.528744
12	6	0	1.750635	6.909509	-1.577994
13	6	0	1.0963	4.426388	-0.339095
14	8	0	-7.476251	-0.185348	4.134226
15	8	0	0.104499	-1.535009	-2.42744
16	6	0	6.992519	0.769498	-0.993275
17	6	0	6.191848	-1.173355	-2.601867
18	6	0	7.878626	-3.064127	-3.316639
19	6	0	10.363885	-3.022794	-2.445506
20	6	0	11.162393	-1.059498	-0.833296
21	6	0	9.496297	0.818154	-0.110358
22	6	0	-0.422447	-4.566715	2.181807
23	6	0	0.733144	-2.61986	3.936633
24	6	0	3.254302	-1.912708	3.567178
25	6	0	4.391079	-0.091008	5.094709
26	6	0	3.006273	1.084328	7.002887
27	6	0	0.494633	0.386502	7.400688
28	6	0	-0.62908	-1.460824	5.893731
29	8	0	12.007748	-4.863179	-3.140418
30	6	0	-5.849456	-5.007769	3.748546
31	8	0	2.944977	2.59253	-1.136903
32	6	0	5.276672	2.786128	-0.103986
33	8	0	5.86399	4.439145	1.39066
34	8	0	13.651879	-1.211004	-0.091684
35	1	0	-1.530693	3.444242	-3.28839
36	16	0	-7.384613	1.413107	-2.126595
37	16	0	-8.501951	-2.284312	-2.750324
38	16	0	-5.388486	-4.020145	-4.561482
39	16	0	-3.513549	-6.032889	-1.744794
40	1	0	-7.050515	4.827791	1.838959
41	1	0	-4.284822	4.064885	3.567145
42	1	0	-5.319154	8.955825	-0.180468
43	1	0	1.194822	10.326094	-3.529039
44	1	0	3.760662	7.190431	-1.846568
45	1	0	1.158474	4.52557	1.725112
46	1	0	4.249262	-1.217803	-3.242362
47	1	0	7.298893	-4.593874	-4.552222
48	1	0	10.079204	2.342582	1.135895
49	1	0	-1.114273	-6.211769	3.229857
50	1	0	1.011369	-5.232248	0.85692
51	1	0	4.331385	-2.781776	2.054573
52	1	0	6.341697	0.438982	4.759408
53	1	0	3.879247	2.527914	8.171345
54	1	0	-0.599176	1.270593	8.896516
55	1	0	-2.574879	-1.974949	6.263931
56	1	0	13.625662	-4.487803	-2.350257
57	1	0	-7.844405	-4.616404	4.06935
58	1	0	-5.645529	-6.790566	2.738164
59	1	0	-4.898691	-5.130566	5.583388

60	1	0	14.059841	0.213338	0.983717

**11b**000004_en_		Standard Orientation (A.U.)			
Center number	Atomic number	Atomic Type	X	Y	Z
0	7	0	2.475168	1.117064	0.178321
1	6	0	1.567476	-1.196566	0.892923
2	6	0	2.646961	-3.490274	-0.578751
3	7	0	4.820001	-2.965685	-2.191093
4	6	0	5.87925	-0.636459	-2.524472
5	6	0	5.067393	1.372405	-0.676833
6	6	0	5.140195	4.071252	-1.677102
7	6	0	3.480928	5.40148	0.216776
8	6	0	1.538339	3.551392	1.223246
9	6	0	3.755526	7.817589	0.862585
10	8	0	2.226658	9.197332	2.409503
11	6	0	-0.323033	8.764499	2.528449
12	6	0	-1.750635	6.909476	1.578248
13	6	0	-1.096607	4.426282	0.339293
14	8	0	7.475239	-0.186114	-4.134876
15	8	0	-0.105213	-1.534788	2.427329
16	6	0	-6.992969	0.769682	0.993091
17	6	0	-6.192682	-1.173006	2.60211
18	6	0	-7.879662	-3.063618	3.316791
19	6	0	-10.364741	-3.022296	2.445137
20	6	0	-11.162829	-1.059262	0.832402
21	6	0	-9.496528	0.81825	0.109562
22	6	0	0.422647	-4.567524	-2.18093
23	6	0	-0.732234	-2.620879	-3.936462
24	6	0	-3.252857	-1.911971	-3.56675
25	6	0	-4.388841	-0.090307	-5.094931
26	6	0	-3.003685	1.083299	-7.00393
27	6	0	-0.492554	0.383755	-7.401926
28	6	0	0.630324	-1.463587	-5.894359
29	8	0	-12.008839	-4.862487	3.140023
30	6	0	5.849952	-5.009024	-3.746552
31	8	0	-2.945343	2.59259	1.137275
32	6	0	-5.276901	2.786184	0.104024
33	8	0	-5.863892	4.439125	-1.390862
34	8	0	-13.652077	-1.21097	0.090043
35	1	0	1.531128	3.444643	3.288166
36	16	0	7.384622	1.413904	2.125636
37	16	0	8.501819	-2.283361	2.750925
38	16	0	5.388333	-4.018415	4.562651
39	16	0	3.513383	-6.032371	1.746773
40	1	0	7.049456	4.827463	-1.841051
41	1	0	4.28326	4.063757	-3.568105
42	1	0	5.318992	8.955592	0.179043
43	1	0	-1.194269	10.326286	3.528766

44	1	0	-3.760589	7.190461	1.847311
45	1	0	-1.159092	4.525551	-1.724895
46	1	0	-4.250202	-1.217367	3.242937
47	1	0	-7.300285	-4.593186	4.552758
48	1	0	-10.079113	2.342476	-1.137082
49	1	0	1.114705	-6.212862	-3.228341
50	1	0	-1.011511	-5.232484	-0.856138
51	1	0	-4.330158	-2.779665	-2.053503
52	1	0	-6.339083	0.441006	-4.759563
53	1	0	-3.876037	2.526864	-8.172882
54	1	0	0.601496	1.26649	-8.898376
55	1	0	2.57576	-1.979002	-6.264669
56	1	0	-13.626673	-4.486939	2.349775
57	1	0	4.897878	-5.134178	-5.580563
58	1	0	7.84432	-4.616303	-4.069234
59	1	0	5.648145	-6.791114	-2.734473
60	1	0	-14.060491	0.214602	-0.983556

**11b**000005_en_		Standard Orientation (A.U.)			
Center number	Atomic number	Atomic Type	X	Y	Z
0	7	0	-2.812403	0.886389	-0.015695
1	6	0	-1.399485	-0.662319	-1.539231
2	6	0	-0.711151	-3.263509	-0.389908
3	7	0	-1.829255	-3.777317	2.06865
4	6	0	-3.686328	-2.368093	3.206509
5	6	0	-4.672753	-0.138929	1.71508
6	6	0	-5.435166	2.189571	3.252601
7	6	0	-5.397565	4.215134	1.239608
8	6	0	-3.417413	3.524527	-0.697565
9	6	0	-6.736908	6.345809	1.29396
10	8	0	-6.557571	8.335968	-0.343209
11	6	0	-4.33014	9.023314	-1.477553
12	6	0	-2.044497	7.942861	-1.435675
13	6	0	-1.234609	5.447626	-0.349901
14	8	0	-4.556931	-2.839374	5.292825
15	8	0	-0.578906	-0.047602	-3.592483
16	6	0	5.174181	3.077182	-1.470863
17	6	0	4.896309	1.982744	-3.866667
18	6	0	6.738911	0.348977	-4.792802
19	6	0	8.83225	-0.250697	-3.303155
20	6	0	9.108234	0.865628	-0.904105
21	6	0	7.332693	2.557255	-0.020341
22	6	0	2.21698	-3.52537	-0.350758
23	6	0	3.621464	-2.452779	1.899983
24	6	0	5.930948	-3.56723	2.561724
25	6	0	7.376418	-2.620822	4.552671
26	6	0	6.526873	-0.526813	5.920673
27	6	0	4.226813	0.591164	5.281008

28	6	0	2.787113	-0.369668	3.294131
29	8	0	10.575418	-1.935098	-4.125992
30	6	0	-0.982189	-6.044959	3.412528
31	8	0	0.998894	4.611387	-1.634667
32	6	0	3.173108	4.580938	-0.249857
33	8	0	3.354778	5.581921	1.815246
34	8	0	11.20257	0.104585	0.456396
35	1	0	-4.091178	3.597443	-2.647615
36	16	0	-7.770263	-1.004714	0.043056
37	16	0	-7.522962	-4.75855	-0.986572
38	16	0	-5.08246	-4.724474	-4.156911
39	16	0	-1.615156	-5.830022	-2.726452
40	1	0	-7.23344	1.961676	4.231097
41	1	0	-3.96782	2.526775	4.683536
42	1	0	-8.189396	6.69447	2.698749
43	1	0	-4.633982	10.788105	-2.472032
44	1	0	-0.573289	8.959499	-2.438615
45	1	0	-0.763187	5.585345	1.654957
46	1	0	3.208073	2.350634	-4.958596
47	1	0	6.548028	-0.538341	-6.630811
48	1	0	7.509592	3.391208	1.841269
49	1	0	2.697293	-5.535072	-0.500131
50	1	0	2.886371	-2.64203	-2.096655
51	1	0	6.603431	-5.193449	1.501501
52	1	0	9.136907	-3.550468	5.062888
53	1	0	7.634382	0.210267	7.4843
54	1	0	3.559499	2.242962	6.295115
55	1	0	1.02257	0.546801	2.814048
56	1	0	11.801817	-2.124076	-2.763162
57	1	0	-2.364681	-6.45252	4.878832
58	1	0	-0.883049	-7.623704	2.087806
59	1	0	0.867138	-5.731721	4.278825
60	1	0	10.699412	-0.046702	2.216571

**11b**000006_en_		Standard Orientation (A.U.)			
Center number	Atomic number	Atomic Type	X	Y	Z
0	7	0	-3.374288	0.772471	0.326151
1	6	0	-1.293525	-0.435238	-0.60445
2	6	0	-0.584973	-2.964706	0.694536
3	7	0	-2.476657	-3.864873	2.505748
4	6	0	-4.767104	-2.776941	2.994998
5	6	0	-5.521473	-0.61385	1.302721
6	6	0	-7.114183	1.457531	2.525862
7	6	0	-6.700832	3.621075	0.713947
8	6	0	-4.105412	3.360362	-0.456765
9	6	0	-8.341108	5.491854	0.336619
10	8	0	-7.974668	7.618576	-1.080093
11	6	0	-5.655579	8.7467	-1.286221

12	6	0	-3.368686	8.008315	-0.50126
13	6	0	-2.467954	5.552386	0.602663
14	8	0	-6.197103	-3.548433	4.638138
15	8	0	0.092952	0.492886	-2.192034
16	6	0	4.334702	4.032644	0.544204
17	6	0	4.972286	4.751724	-1.925695
18	6	0	7.404799	4.372269	-2.839127
19	6	0	9.205316	3.210466	-1.299053
20	6	0	8.563154	2.478946	1.175833
21	6	0	6.168155	2.920023	2.108048
22	6	0	1.949543	-2.526207	2.173168
23	6	0	4.483187	-3.046825	0.938753
24	6	0	5.135201	-2.254129	-1.501632
25	6	0	7.567636	-2.651423	-2.432265
26	6	0	9.400859	-3.832745	-0.950527
27	6	0	8.776242	-4.635672	1.486703
28	6	0	6.33328	-4.245458	2.411067
29	8	0	11.549952	2.709227	-2.178919
30	6	0	-1.961838	-6.17818	3.946172
31	8	0	0.150039	5.387958	-0.118786
32	6	0	1.747339	4.265582	1.539462
33	8	0	1.062764	3.48783	3.6045
34	8	0	10.454252	1.275602	2.535753
35	1	0	-4.117482	3.478611	-2.519433
36	16	0	-7.64234	-1.858432	-1.359798
37	16	0	-6.390788	-5.469941	-2.176445
38	16	0	-3.02562	-4.905543	-4.269006
39	16	0	-0.054057	-5.47423	-1.770822
40	1	0	-9.08261	0.913427	2.796192
41	1	0	-6.300234	1.874681	4.389344
42	1	0	-10.222466	5.47917	1.152407
43	1	0	-5.899626	10.56458	-2.198828
44	1	0	-1.865106	9.360803	-0.841006
45	1	0	-2.544828	5.489379	2.664351
46	1	0	3.535695	5.569835	-3.131373
47	1	0	7.922581	4.894291	-4.752572
48	1	0	5.672651	2.348403	4.012021
49	1	0	1.880695	-0.569732	2.84266
50	1	0	1.849854	-3.682791	3.875981
51	1	0	3.743862	-1.281891	-2.639713
52	1	0	8.042704	-1.993798	-4.316315
53	1	0	11.294314	-4.130534	-1.685368
54	1	0	10.168128	-5.596953	2.652265
55	1	0	5.859031	-4.883483	4.30636
56	1	0	12.423151	1.861263	-0.788736
57	1	0	-0.38826	-7.183036	3.082128
58	1	0	-1.537031	-5.741715	5.921789
59	1	0	-3.640433	-7.373828	3.888121
60	1	0	9.907195	-0.459714	2.828534

**11b**000007_en_		Standard Orientation (A.U.)			
Center number	Atomic number	Atomic Type	X	Y	Z
0	7	0	2.474736	1.117158	0.178106
1	6	0	1.567403	-1.19657	0.89296
2	6	0	2.647142	-3.490308	-0.578588
3	7	0	4.820329	-2.965589	-2.190877
4	6	0	5.879124	-0.636185	-2.524467
5	6	0	5.06692	1.372604	-0.676876
6	6	0	5.140065	4.071574	-1.676634
7	6	0	3.48046	5.401529	0.21717
8	6	0	1.537755	3.5514	1.223135
9	6	0	3.755088	7.817575	0.863281
10	8	0	2.226028	9.197358	2.409897
11	6	0	-0.323585	8.764271	2.528809
12	6	0	-1.751149	6.909466	1.578097
13	6	0	-1.097151	4.426367	0.339005
14	8	0	7.474892	-0.185476	-4.134995
15	8	0	-0.105162	-1.53483	2.427488
16	6	0	-6.993381	0.769594	0.993127
17	6	0	-6.192695	-1.173569	2.601387
18	6	0	-7.879357	-3.064661	3.315536
19	6	0	-10.364525	-3.023389	2.444121
20	6	0	-11.162993	-1.059933	0.832092
21	6	0	-9.496987	0.818021	0.109709
22	6	0	0.423007	-4.567552	-2.181149
23	6	0	-0.731204	-2.62077	-3.936953
24	6	0	-3.251413	-1.910531	-3.566829
25	6	0	-4.386826	-0.088523	-5.095003
26	6	0	-3.001478	1.084112	-7.004485
27	6	0	-0.490776	0.383277	-7.402861
28	6	0	0.63151	-1.464425	-5.895264
29	8	0	-12.008328	-4.86397	3.1386
30	6	0	5.850627	-5.009118	-3.745741
31	8	0	-2.945769	2.592532	1.13689
32	6	0	-5.277694	2.786658	0.104568
33	8	0	-5.865175	4.440281	-1.389342
34	8	0	-13.652163	-1.211929	0.089521
35	1	0	1.530119	3.444442	3.288054
36	16	0	7.384068	1.413818	2.126067
37	16	0	8.501423	-2.283127	2.75129
38	16	0	5.387907	-4.01851	4.563037
39	16	0	3.513309	-6.032335	1.74682
40	1	0	7.049401	4.827752	-1.839936
41	1	0	4.283643	4.06457	-3.567854
42	1	0	5.318763	8.955515	0.180122
43	1	0	-1.19492	10.325794	3.529456
44	1	0	-3.761086	7.190469	1.847263

45	1	0	-1.159495	4.525649	-1.725209
46	1	0	-4.250215	-1.217872	3.242222
47	1	0	-7.299697	-4.594477	4.551068
48	1	0	-10.079868	2.342524	-1.136461
49	1	0	1.115057	-6.213099	-3.228218
50	1	0	-1.011456	-5.232171	-0.856525
51	1	0	-4.328774	-2.777561	-2.053261
52	1	0	-6.336753	0.443759	-4.759373
53	1	0	-3.873391	2.527883	-8.173513
54	1	0	0.603429	1.265254	-8.899645
55	1	0	2.576641	-1.980741	-6.265952
56	1	0	-13.626487	-4.487955	2.349245
57	1	0	7.844432	-4.615056	-4.070139
58	1	0	5.650909	-6.790744	-2.732389
59	1	0	4.897309	-5.136366	-5.578963
60	1	0	-14.06223	0.21669	-0.979388

Table S17. Gibbs free energies[a] and equilibrium populations[b] of low-energy conformers of 12a.

Conformers	ΔG(a.u.)	P(%)/100	G(a.u.)
**12a**2000002.out	0.0032	1.08	-2700.001726
**12a**2000005.out	0.00322	1.07	-2700.001711
**12a**2000006.out	0.00347	0.82	-2700.001459
**12a**2000007.out	0.01114	0.0	-2699.993789
**12a**2000009.out	0.00012	28.2	-2700.004804
**12a**2000013.out	0.00646	0.03	-2699.998471
**12a**2000016.out	0.00369	0.65	-2700.00124
**12a**2000017.out	0.0	32.08	-2700.004926
**12a**2000019.out	0.00779	0.01	-2699.997132
**12a**2000020.out	0.00354	0.75	-2700.001384
**12a**2000021.out	2e-05	31.45	-2700.004907
**12a**2000024.out	0.00727	0.01	-2699.997661
**12a**2000026.out	0.00461	0.24	-2700.000318
**12a**2000028.out	0.00239	2.56	-2700.00254
**12a**2000030.out	0.00323	1.05	-2700.001694

[a]wB97M-V/def2-TZVP, in a.u.

[b]From ΔG values at 298.15 K.

Table S18. Cartesian coordinates for the low-energy reoptimized random research conformers of 12a at B3LYP-D3(BJ)/6-31G* level of theory in chloroform.

**12a**000002_en_		Standard Orientation (A.U.)			
Center number	Atomic number	Atomic Type	X	Y	Z
0	16	0	3.958464	13.947228	40.173883
1	16	0	4.932146	17.667885	38.950478
2	7	0	8.870679	14.48926	42.01848
3	8	0	17.429063	2.265663	36.426414
4	8	0	11.84431	12.142609	32.853611
5	8	0	8.324537	21.54891	37.27314
6	1	0	8.985406	22.011929	38.919513
7	8	0	15.599428	12.848174	30.971081
8	8	0	12.728114	3.990261	37.499117
9	1	0	11.462346	8.326984	35.17385
10	8	0	7.800285	10.637257	36.500058
11	8	0	16.243139	4.343375	41.409418
12	1	0	16.397309	3.388702	39.83513
13	7	0	8.846811	14.820703	36.746263
14	6	0	16.348586	-0.103899	35.701727
15	1	0	16.743767	-0.492869	33.692581
16	1	0	14.294251	-0.121617	36.031745
17	1	0	17.259302	-1.553196	36.875296
18	6	0	16.553247	4.35025	35.159479
19	6	0	18.113989	5.656485	33.473312
20	1	0	18.524324	9.040227	31.195123
21	6	0	17.305065	7.941419	32.443132
22	1	0	19.988098	4.893162	33.072258
23	6	0	14.910572	8.903269	33.062193
24	6	0	14.209731	11.465887	32.146668
25	6	0	11.178344	14.730534	32.451248
26	1	0	12.793864	15.88157	33.082319
27	6	0	8.821	15.441107	34.025317
28	1	0	7.15469	14.547664	33.161222
29	6	0	8.70447	18.294129	34.045215
30	8	0	7.994917	19.076375	29.627826
31	6	0	8.305794	19.809338	32.070546
32	1	0	8.161017	21.855297	32.29518
33	6	0	9.367187	19.209242	36.664242
34	1	0	11.454851	19.234853	36.877911
35	6	0	8.417237	17.011116	38.274473
36	6	0	10.573365	15.198595	29.706166
37	1	0	8.79646	16.955126	26.594129
38	6	0	9.135263	16.989677	28.629461
39	1	0	11.35976	13.820365	28.390369
40	6	0	14.141558	5.296872	35.733245
41	6	0	12.185663	5.434524	39.62949
42	6	0	9.947055	6.787386	39.761966

43	1	0	8.602139	6.551673	38.228978
44	6	0	9.482801	8.45543	41.761309
45	6	0	7.073691	10.043775	41.782094
46	1	0	6.502782	10.352031	43.753982
47	8	0	5.019058	8.834989	40.613412
48	6	0	7.382151	12.730272	40.566583
49	6	0	8.10317	12.557818	37.74285
50	6	0	8.642439	14.541276	44.77525
51	1	0	9.68221	12.996321	45.673003
52	1	0	6.631856	14.447448	45.31108
53	1	0	9.443774	16.339113	45.417489
54	6	0	9.494181	16.767374	40.893806
55	6	0	11.283022	8.556417	43.70658
56	1	0	14.89927	7.24997	45.13481
57	6	0	13.510487	7.148072	43.612693
58	1	0	10.952989	9.730215	45.367589
59	6	0	14.037409	5.597061	41.531082
60	6	0	13.306773	7.556809	34.680042
61	8	0	10.600417	18.502543	41.924491
62	1	0	5.383115	8.817007	38.810447

**12a**2000005_en_		Standard Orientation (A.U.)			
Center number	Atomic number	Atomic Type	X	Y	Z
0	16	0	4.166147	12.616579	38.944287
1	16	0	4.376277	16.543073	38.019098
2	7	0	8.444268	14.103654	41.686911
3	8	0	16.020257	1.980029	36.107232
4	8	0	13.641307	13.24219	33.466957
5	8	0	6.936064	21.237808	37.228652
6	1	0	7.060087	21.702496	38.997602
7	8	0	11.709066	11.485655	30.073691
8	8	0	16.358152	6.213491	38.853935
9	1	0	15.395778	10.421847	36.420442
10	8	0	9.334172	10.631068	35.910086
11	8	0	17.412827	5.501788	43.737039
12	1	0	18.307735	5.162118	42.171227
13	7	0	9.18998	14.897843	36.553086
14	6	0	15.658677	-0.335853	34.827407
15	1	0	13.667017	-0.594532	34.261009
16	1	0	16.185767	-1.832142	36.16381
17	1	0	16.870196	-0.47526	33.133865
18	6	0	15.463657	4.154211	34.909079
19	6	0	14.576552	4.339713	32.410001
20	1	0	13.024559	6.807697	29.540919
21	6	0	13.866637	6.677712	31.419208
22	1	0	14.33811	2.643842	31.266609
23	6	0	14.155672	8.887495	32.842293
24	6	0	13.032207	11.286582	31.921524

25	6	0	12.515932	15.678425	33.135055
26	1	0	13.599696	16.841043	34.478174
27	6	0	9.710401	15.795265	33.953087
28	1	0	8.575925	14.700227	32.596821
29	6	0	8.963929	18.545854	34.097814
30	8	0	9.429405	19.750686	29.747063
31	6	0	8.788101	20.187946	32.191817
32	1	0	8.079426	22.103698	32.490823
33	6	0	8.667227	19.29037	36.832011
34	1	0	10.548687	19.802671	37.60439
35	6	0	7.957591	16.779863	38.064466
36	6	0	12.818521	16.637168	30.471766
37	1	0	11.882776	18.563895	27.109958
38	6	0	11.424092	18.24612	29.09665
39	1	0	14.388715	15.78818	29.438662
40	6	0	15.751277	6.398337	36.325351
41	6	0	14.480898	6.846502	40.56525
42	6	0	12.1284	7.799353	39.905757
43	1	0	11.640925	8.042406	37.932027
44	6	0	10.352194	8.448973	41.761256
45	6	0	7.748697	9.422649	40.99168
46	1	0	6.570876	9.45094	42.701629
47	8	0	6.492094	7.817814	39.286295
48	6	0	7.663227	12.182463	39.918821
49	6	0	8.884213	12.44234	37.274763
50	6	0	8.150189	13.756144	44.411153
51	1	0	8.41447	15.614112	45.286183
52	1	0	9.564053	12.442412	45.156686
53	1	0	6.234424	13.064928	44.843488
54	6	0	8.591812	16.55511	40.82964
55	6	0	10.972137	7.999001	44.295249
56	1	0	13.817326	6.655347	46.942539
57	6	0	13.334577	7.017422	44.969684
58	1	0	9.589634	8.371768	45.78276
59	6	0	15.12639	6.44973	43.117222
60	6	0	15.204177	8.728904	35.268641
61	8	0	9.03523	18.405953	42.129042
62	1	0	7.314869	8.029102	37.653139

**12a**2000006_en_		Standard Orientation (A.U.)			
Center number	Atomic number	Atomic Type	X	Y	Z
0	16	0	4.273213	14.010649	40.688073
1	16	0	5.312035	17.738477	39.724721
2	7	0	9.539483	14.267918	41.710423
3	8	0	17.614124	2.628796	36.414448
4	8	0	11.52543	12.096859	32.71698
5	8	0	8.090549	21.523537	37.073923
6	1	0	6.293493	21.467461	36.715695

7	8	0	15.185191	13.027532	30.739026
8	8	0	12.918871	4.013856	37.31667
9	1	0	11.393962	8.229013	34.946161
10	8	0	7.448412	10.591461	36.455642
11	8	0	15.752349	3.255186	41.577565
12	1	0	15.967691	2.657369	39.843403
13	7	0	8.553915	14.758119	36.630075
14	6	0	20.149394	1.820343	36.117591
15	1	0	21.493099	3.301968	36.707061
16	1	0	20.540537	1.28069	34.142623
17	1	0	20.3803	0.159678	37.33791
18	6	0	16.807978	4.70714	35.147776
19	6	0	18.291857	6.143513	33.485872
20	1	0	18.467468	9.518692	31.17674
21	6	0	17.309292	8.343277	32.413439
22	1	0	20.231588	5.590511	33.071577
23	6	0	14.836325	9.110678	32.956584
24	6	0	13.930897	11.576487	31.98246
25	6	0	10.683905	14.614295	32.241162
26	1	0	12.265173	15.881345	32.712216
27	6	0	8.377286	15.313158	33.919074
28	1	0	6.688007	14.378535	33.151301
29	6	0	8.268714	18.15211	33.880877
30	8	0	7.343215	18.817721	29.475532
31	6	0	7.807377	19.610979	31.871926
32	1	0	7.755755	21.669826	32.024191
33	6	0	9.120837	19.185734	36.418993
34	1	0	11.191225	19.443262	36.460986
35	6	0	8.509322	16.963763	38.206889
36	6	0	9.889848	14.925522	29.522806
37	1	0	7.884283	16.561278	26.475621
38	6	0	8.384923	16.675567	28.473734
39	1	0	10.554623	13.458983	28.234893
40	6	0	14.29547	5.4596	35.642178
41	6	0	12.225891	5.238861	39.532698
42	6	0	10.180731	6.871027	39.604294
43	1	0	9.052453	7.108851	37.90826
44	6	0	9.587501	8.203159	41.813122
45	6	0	7.341144	9.998739	41.886466
46	1	0	6.931899	10.412712	43.879169
47	8	0	5.113962	8.914925	40.908317
48	6	0	7.704735	12.630408	40.584756
49	6	0	7.940179	12.483281	37.688836
50	6	0	10.18754	14.074106	44.385791
51	1	0	11.342157	12.397843	44.762355
52	1	0	8.458305	14.023312	45.545157
53	1	0	11.282521	15.764984	44.865802
54	6	0	10.141809	16.537014	40.516524
55	6	0	11.021695	7.685244	43.983869

56	1	0	14.175153	5.63231	45.626735
57	6	0	13.062269	6.011207	43.931567
58	1	0	10.52568	8.576199	45.779109
59	6	0	13.737432	4.799717	41.676256
60	6	0	13.317864	7.627851	34.543213
61	8	0	11.687431	18.040072	41.284671
62	1	0	5.344852	8.77979	39.088093

**12a**2000007_en_		Standard Orientation (A.U.)			
Center number	Atomic number	Atomic Type	X	Y	Z
0	16	0	4.167145	12.625128	39.336091
1	16	0	4.465366	16.607779	39.305557
2	7	0	8.749591	13.644082	41.9293
3	8	0	15.314513	1.887203	34.360983
4	8	0	13.341117	13.576004	33.539062
5	8	0	6.783573	21.316288	38.275367
6	1	0	5.306487	21.013738	37.23262
7	8	0	11.149067	12.319597	30.084453
8	8	0	16.190996	6.01638	37.880541
9	1	0	15.313749	10.409364	35.915012
10	8	0	9.068216	10.900483	35.773347
11	8	0	17.28298	4.409542	42.529186
12	1	0	18.234805	4.537977	40.967577
13	7	0	9.009789	15.069697	36.904775
14	6	0	17.166926	1.120275	36.144569
15	1	0	16.520645	1.360658	38.105247
16	1	0	18.945584	2.174427	35.881158
17	1	0	17.507612	-0.894927	35.779323
18	6	0	14.915223	4.338577	33.802769
19	6	0	13.775049	4.839248	31.446797
20	1	0	12.063343	7.632489	29.004269
21	6	0	13.084857	7.274153	30.759479
22	1	0	13.367993	3.221794	30.23315
23	6	0	13.652283	9.319593	32.353421
24	6	0	12.571114	11.846056	31.804267
25	6	0	12.320714	16.075207	33.529039
26	1	0	13.476586	17.025877	34.974127
27	6	0	9.529029	16.227944	34.417635
28	1	0	8.329206	15.345933	32.965494
29	6	0	8.929784	18.967013	34.893787
30	8	0	9.471562	20.710108	30.72021
31	6	0	8.866442	20.844129	33.202284
32	1	0	8.294367	22.750931	33.755645
33	6	0	8.57646	19.462966	37.695146
34	1	0	10.351374	20.107055	38.579479
35	6	0	8.034048	16.805011	38.748122
36	6	0	12.637914	17.312539	30.984362
37	1	0	11.787446	19.687242	27.893679

38	6	0	11.343497	19.162728	29.838796
39	1	0	14.126049	16.482989	29.822144
40	6	0	15.446555	6.405569	35.398376
41	6	0	14.363132	6.422378	39.718451
42	6	0	12.045885	7.57314	39.287214
43	1	0	11.534405	8.177314	37.397975
44	6	0	10.327433	7.940522	41.269559
45	6	0	7.74779	9.125467	40.770454
46	1	0	6.666444	8.999637	42.538527
47	8	0	6.314645	7.801443	38.963881
48	6	0	7.736441	11.989364	40.047613
49	6	0	8.730916	12.545429	37.36409
50	6	0	8.675804	12.987045	44.61012
51	1	0	9.19859	14.692122	45.662295
52	1	0	10.020734	11.4783	45.05706
53	1	0	6.749951	12.408957	45.153747
54	6	0	9.058905	16.185788	41.349402
55	6	0	10.96829	7.028861	43.671072
56	1	0	13.799175	5.139766	45.979335
57	6	0	13.297545	5.855861	44.110946
58	1	0	9.628534	7.200711	45.233161
59	6	0	15.028393	5.55684	42.143247
60	6	0	14.926701	8.861041	34.617589
61	8	0	9.927527	17.751819	42.776867
62	1	0	7.083045	8.139487	37.324931

**12a**2000009_en_		Standard Orientation (A.U.)			
Center number	Atomic number	Atomic Type	X	Y	Z
0	16	0	4.369914	14.419555	40.669834
1	16	0	5.520667	18.040418	39.333323
2	7	0	9.565183	14.372091	41.754019
3	8	0	16.894872	2.19072	36.211783
4	8	0	11.413613	12.05644	32.564615
5	8	0	8.955522	21.585453	37.17988
6	1	0	9.863142	21.99368	38.719101
7	8	0	15.10213	12.701598	30.532131
8	8	0	12.342671	3.912336	37.226059
9	1	0	11.055761	8.216187	34.866645
10	8	0	7.327543	10.786751	36.439835
11	8	0	15.259896	3.081593	41.4151
12	1	0	15.386369	2.436893	39.688807
13	7	0	8.788765	14.843593	36.611565
14	6	0	19.35387	1.195652	35.849218
15	1	0	19.505105	-0.457304	37.092414
16	1	0	20.819901	2.585406	36.367071
17	1	0	19.639166	0.596439	33.873277
18	6	0	16.201614	4.306091	34.938319
19	6	0	17.731812	5.609542	33.210948

20	1	0	18.081111	8.940727	30.858729
21	6	0	16.879627	7.865824	32.14448
22	1	0	19.610605	4.909544	32.741355
23	6	0	14.489655	8.822247	32.75612
24	6	0	13.755366	11.350338	31.792584
25	6	0	10.788061	14.654877	32.170664
26	1	0	12.484279	15.759753	32.653615
27	6	0	8.587031	15.481515	33.911459
28	1	0	6.811765	14.698152	33.168564
29	6	0	8.67013	18.332269	33.939478
30	8	0	7.597997	19.122105	29.604767
31	6	0	8.19558	19.862675	31.994057
32	1	0	8.226265	21.916125	32.194266
33	6	0	9.712881	19.168671	36.459671
34	1	0	11.805372	19.017155	36.416062
35	6	0	8.780728	17.047407	38.187423
36	6	0	9.98208	15.122703	29.479412
37	1	0	8.015829	16.953846	26.524691
38	6	0	8.540863	16.977867	28.520301
39	1	0	10.58159	13.694127	28.119036
40	6	0	13.767303	5.245884	35.49932
41	6	0	11.773566	5.201384	39.44007
42	6	0	9.80511	6.922798	39.545263
43	1	0	8.640004	7.180867	37.877771
44	6	0	9.332677	8.310112	41.750235
45	6	0	7.167089	10.204368	41.853396
46	1	0	6.791118	10.62347	43.851497
47	8	0	4.889893	9.227274	40.887233
48	6	0	7.663818	12.825314	40.562089
49	6	0	7.963697	12.637925	37.666193
50	6	0	10.107693	14.155555	44.45213
51	1	0	11.230514	15.814961	44.975966
52	1	0	11.19641	12.445092	44.864561
53	1	0	8.3362	14.156889	45.546092
54	6	0	10.210126	16.626856	40.609111
55	6	0	10.802391	7.753779	43.887408
56	1	0	13.903482	5.580401	45.47385
57	6	0	12.765162	5.989891	43.802799
58	1	0	10.392638	8.68014	45.686149
59	6	0	13.32293	4.720472	41.547609
60	6	0	12.91642	7.47158	34.407555
61	8	0	11.648429	18.20338	41.475322
62	1	0	5.086251	9.126379	39.061144

**12a**2000013_en_		Standard Orientation (A.U.)			
Center number	Atomic number	Atomic Type	X	Y	Z
0	16	0	4.305938	12.916278	39.337072
1	16	0	4.57114	16.859367	38.765368

2	7	0	8.892239	14.322769	41.739281
3	8	0	15.699986	1.814842	35.928429
4	8	0	13.439481	13.121423	33.405624
5	8	0	6.850053	21.399478	37.070922
6	1	0	5.359284	20.938137	36.10869
7	8	0	11.320382	11.423003	30.096362
8	8	0	16.241128	6.039141	38.656321
9	1	0	15.268885	10.267806	36.260033
10	8	0	9.214938	10.74802	36.027071
11	8	0	17.501457	5.285842	43.478768
12	1	0	18.301043	4.904977	41.871414
13	7	0	9.1114	15.03433	36.562848
14	6	0	15.247318	-0.49315	34.663201
15	1	0	16.379197	-0.646439	32.916626
16	1	0	13.22862	-0.722081	34.187035
17	1	0	15.812578	-1.999366	35.972536
18	6	0	15.126935	3.999541	34.758277
19	6	0	14.126788	4.20373	32.303472
20	1	0	12.489916	6.702821	29.509126
21	6	0	13.417358	6.555587	31.345469
22	1	0	13.801647	2.513037	31.173918
23	6	0	13.822079	8.758317	32.751248
24	6	0	12.716547	11.183679	31.885351
25	6	0	12.403516	15.593865	33.070689
26	1	0	13.55376	16.730966	34.379172
27	6	0	9.612997	15.840461	33.936584
28	1	0	8.417776	14.754549	32.625264
29	6	0	8.98509	18.613438	34.030785
30	8	0	9.484299	19.760462	29.653424
31	6	0	8.885532	20.235363	32.095445
32	1	0	8.291041	22.193709	32.379996
33	6	0	8.648772	19.49159	36.738425
34	1	0	10.426906	20.261067	37.508781
35	6	0	8.133419	17.002389	38.153718
36	6	0	12.714739	16.49359	30.387004
37	1	0	11.841289	18.413777	27.001537
38	6	0	11.392361	18.146437	28.997339
39	1	0	14.229347	15.550331	29.352538
40	6	0	15.52403	6.23682	36.159284
41	6	0	14.480499	6.765395	40.453989
42	6	0	12.146373	7.832637	39.910625
43	1	0	11.568696	8.104302	37.964626
44	6	0	10.501475	8.566949	41.85283
45	6	0	7.915188	9.673438	41.22882
46	1	0	6.853893	9.791703	43.009583
47	8	0	6.459209	8.116635	39.634368
48	6	0	7.883374	12.411583	40.114326
49	6	0	8.86322	12.59544	37.373586
50	6	0	8.851439	14.050932	44.486693

51	1	0	10.24015	12.657618	45.129997
52	1	0	6.945985	13.504009	45.125343
53	1	0	9.332197	15.901433	45.281795
54	6	0	9.186416	16.757549	40.804524
55	6	0	11.227676	8.092491	44.35397
56	1	0	14.137007	6.619106	46.858288
57	6	0	13.570193	6.99813	44.911169
58	1	0	9.944672	8.544004	45.907521
59	6	0	15.234615	6.340942	42.971524
60	6	0	14.982987	8.577966	35.124083
61	8	0	10.065638	18.511855	41.985973
62	1	0	7.212387	8.222028	37.958054

**12a**2000016_en_		Standard Orientation (A.U.)			
Center number	Atomic number	Atomic Type	X	Y	Z
0	16	0	4.748731	14.8461	40.880861
1	16	0	6.206857	18.412053	39.855428
2	7	0	10.046948	14.429399	41.657671
3	8	0	16.356562	1.880225	36.012433
4	8	0	11.302683	12.03868	32.579479
5	8	0	9.319128	21.8159	37.071961
6	1	0	7.514609	21.987037	36.797847
7	8	0	14.941874	12.497477	30.410157
8	8	0	11.929966	3.841201	37.16304
9	1	0	10.807686	8.207054	34.847871
10	8	0	7.267036	11.055187	36.5207
11	8	0	14.872271	2.780301	41.272675
12	1	0	14.917022	2.149643	39.537415
13	7	0	8.89499	15.050905	36.623762
14	6	0	18.746697	0.75594	35.574777
15	1	0	18.941838	0.152146	33.589228
16	1	0	18.844202	-0.909102	36.80716
17	1	0	20.299744	2.062156	36.054782
18	6	0	15.740867	4.03506	34.767147
19	6	0	17.289824	5.266465	33.003096
20	1	0	17.752711	8.589608	30.658953
21	6	0	16.530621	7.571747	31.971396
22	1	0	19.115581	4.471232	32.479269
23	6	0	14.211434	8.648356	32.652241
24	6	0	13.578334	11.213612	31.720911
25	6	0	10.778507	14.648233	32.142463
26	1	0	12.532121	15.692027	32.54668
27	6	0	8.662729	15.633474	33.921965
28	1	0	6.831509	14.929634	33.237543
29	6	0	8.922514	18.462893	33.880142
30	8	0	7.897532	19.268158	29.519709
31	6	0	8.565681	19.980091	31.892938
32	1	0	8.789407	22.027217	32.047711

33	6	0	10.014534	19.369761	36.374376
34	1	0	12.100597	19.361648	36.321655
35	6	0	9.207333	17.239036	38.193387
36	6	0	9.914654	15.077167	29.462064
37	1	0	8.011519	16.972972	26.501854
38	6	0	8.606958	17.012653	28.476739
39	1	0	10.329878	13.545916	28.145313
40	6	0	13.375427	5.09884	35.397603
41	6	0	11.499938	5.155024	39.394217
42	6	0	9.666612	7.017298	39.551989
43	1	0	8.487365	7.378523	37.913339
44	6	0	9.34562	8.414754	41.776816
45	6	0	7.339817	10.470629	41.946982
46	1	0	7.076287	10.927139	43.954884
47	8	0	4.953575	9.673126	41.075184
48	6	0	7.970717	13.039558	40.622904
49	6	0	8.0496	12.869744	37.719731
50	6	0	10.778907	14.161184	44.305232
51	1	0	9.10321	14.303401	45.533325
52	1	0	12.075437	15.717897	44.734614
53	1	0	11.753751	12.366464	44.642433
54	6	0	10.877434	16.604587	40.42642
55	6	0	10.815797	7.736078	43.87795
56	1	0	13.788113	5.334319	45.37956
57	6	0	12.643704	5.836508	43.738286
58	1	0	10.514882	8.681916	45.688117
59	6	0	13.058325	4.549312	41.462406
60	6	0	12.613984	7.370607	34.33676
61	8	0	12.635682	17.897878	41.115403
62	1	0	5.081067	9.520246	39.246191

**12a**2000017_en_		Standard Orientation (A.U.)			
Center number	Atomic number	Atomic Type	X	Y	Z
0	16	0	4.260864	14.024338	40.680747
1	16	0	5.167443	17.687323	39.264875
2	7	0	9.43664	14.347978	41.783644
3	8	0	17.559579	2.545752	36.392612
4	8	0	11.484028	12.004636	32.62492
5	8	0	8.383333	21.425722	37.080641
6	1	0	9.246022	21.911539	38.623296
7	8	0	15.125536	12.855916	30.581251
8	8	0	12.921938	4.017584	37.41218
9	1	0	11.376267	8.205584	34.998274
10	8	0	7.472693	10.53179	36.519494
11	8	0	15.922923	3.498405	41.595278
12	1	0	16.079734	2.812593	39.887498
13	7	0	8.667019	14.676753	36.62818
14	6	0	20.060562	1.671351	36.007367

15	1	0	20.35129	1.072057	34.032239
16	1	0	20.308477	0.037445	37.26014
17	1	0	21.462421	3.135443	36.497424
18	6	0	16.739506	4.600175	35.095181
19	6	0	18.183909	5.966332	33.342494
20	1	0	18.328286	9.278306	30.94176
21	6	0	17.197264	8.153753	32.248817
22	1	0	20.098415	5.370396	32.873704
23	6	0	14.758582	8.977365	32.860436
24	6	0	13.867436	11.440736	31.862786
25	6	0	10.686392	14.548246	32.189117
26	1	0	12.303763	15.774141	32.649539
27	6	0	8.432279	15.254351	33.916593
28	1	0	6.716196	14.340359	33.184167
29	6	0	8.323217	18.104533	33.897416
30	8	0	7.209409	18.751303	29.54936
31	6	0	7.751883	19.568097	31.926798
32	1	0	7.644796	21.621885	32.094268
33	6	0	9.297987	19.05058	36.406116
34	1	0	11.396088	19.034934	36.372844
35	6	0	8.497456	16.902168	38.165835
36	6	0	9.850354	14.914813	29.490569
37	1	0	7.767867	16.562076	26.507192
38	6	0	8.29051	16.654905	28.501373
39	1	0	10.540766	13.50352	28.155491
40	6	0	14.258409	5.405985	35.658501
41	6	0	12.275971	5.310512	39.602392
42	6	0	10.188952	6.887332	39.685035
43	1	0	9.002034	7.027187	38.018582
44	6	0	9.630494	8.285752	41.862838
45	6	0	7.335647	10.024084	41.937978
46	1	0	6.928197	10.444029	43.929777
47	8	0	5.135064	8.87652	40.983642
48	6	0	7.651545	12.654816	40.607818
49	6	0	7.981487	12.440088	37.716961
50	6	0	9.965913	14.217126	44.490119
51	1	0	11.178813	12.602372	44.940416
52	1	0	8.187828	14.098583	45.566307
53	1	0	10.952116	15.966107	44.997049
54	6	0	9.930624	16.623002	40.605577
55	6	0	11.147374	7.88164	44.001262
56	1	0	14.407831	5.973477	45.61046
57	6	0	13.233643	6.264423	43.939385
58	1	0	10.679354	8.811079	45.783914
59	6	0	13.869981	4.991475	41.707061
60	6	0	13.274765	7.56224	34.540148
61	8	0	11.242415	18.313593	41.457636
62	1	0	5.34267	8.763626	39.159515

**12a**2000019_en_		Standard Orientation (A.U.)			
Center number	Atomic number	Atomic Type	X	Y	Z
0	16	0	4.271915	13.034501	39.327346
1	16	0	4.731942	16.952559	38.46351
2	7	0	8.822122	14.131529	41.806825
3	8	0	15.05685	1.457986	35.443314
4	8	0	13.388145	13.032545	33.243378
5	8	0	7.605414	21.445245	37.556786
6	1	0	7.894972	21.868408	39.316674
7	8	0	11.0672	11.417028	30.028648
8	8	0	16.04695	5.930892	38.463607
9	1	0	15.229584	10.102175	36.020627
10	8	0	9.068622	10.706597	35.944084
11	8	0	17.240314	4.946789	43.259888
12	1	0	18.153066	4.848119	41.672961
13	7	0	9.288586	14.960316	36.643451
14	6	0	16.932638	0.862131	37.267813
15	1	0	17.224844	-1.189097	37.133916
16	1	0	16.336747	1.344466	39.199547
17	1	0	18.726181	1.838061	36.847743
18	6	0	14.69996	3.838412	34.610202
19	6	0	13.559426	4.093018	32.215815
20	1	0	11.900645	6.628239	29.473182
21	6	0	12.918327	6.449323	31.257888
22	1	0	13.115627	2.357774	31.19257
23	6	0	13.535632	8.649838	32.607701
24	6	0	12.517614	11.123848	31.764289
25	6	0	12.411748	15.541043	32.994887
26	1	0	13.673306	16.619684	34.249608
27	6	0	9.689546	15.856639	34.022367
28	1	0	8.378532	14.872853	32.742249
29	6	0	9.173965	18.653738	34.252042
30	8	0	9.416182	19.892786	29.891745
31	6	0	8.996697	20.337081	32.382304
32	1	0	8.471797	22.29843	32.755213
33	6	0	9.137973	19.372752	37.00981
34	1	0	11.106603	19.715086	37.647347
35	6	0	8.316278	16.905388	38.259989
36	6	0	12.571004	16.489697	30.315478
37	1	0	11.522745	18.524265	27.051666
38	6	0	11.209595	18.227355	29.069508
39	1	0	13.965577	15.516742	29.148791
40	6	0	15.278177	6.06073	35.961536
41	6	0	14.27005	6.599897	40.272065
42	6	0	11.953714	7.710583	39.744754
43	1	0	11.404091	8.060069	37.803299
44	6	0	10.290343	8.372135	41.698508
45	6	0	7.720378	9.53632	41.094979

46	1	0	6.653399	9.621926	42.874513
47	8	0	6.251424	8.050504	39.45548
48	6	0	7.77857	12.312027	40.067973
49	6	0	8.843886	12.52419	37.354904
50	6	0	8.661607	13.762411	44.539235
51	1	0	6.712436	13.272371	45.085147
52	1	0	9.17805	15.564083	45.419469
53	1	0	9.970027	12.29171	45.175689
54	6	0	9.113749	16.579722	40.973129
55	6	0	10.97853	7.773936	44.183902
56	1	0	13.843406	6.16656	46.658234
57	6	0	13.305452	6.637004	44.723022
58	1	0	9.678066	8.162511	45.739946
59	6	0	14.986622	6.057442	42.775208
60	6	0	14.80478	8.423679	34.910175
61	8	0	9.791274	18.362646	42.265823
62	1	0	6.992847	8.211903	37.77804

**12a**000020_en_		Standard Orientation (A.U.)			
Center number	Atomic number	Atomic Type	X	Y	Z
0	16	0	4.088952	14.119818	40.149887
1	16	0	5.137272	17.832667	38.963517
2	7	0	9.028864	14.518515	41.961953
3	8	0	17.308007	2.205826	36.237785
4	8	0	11.938179	12.245433	32.788069
5	8	0	8.605068	21.656636	37.315846
6	1	0	9.279791	22.082992	38.966497
7	8	0	15.721384	12.916798	30.949279
8	8	0	12.637874	4.010001	37.311569
9	1	0	11.473665	8.407303	35.051438
10	8	0	7.833236	10.771469	36.400005
11	8	0	16.104696	4.151611	41.275194
12	1	0	16.259721	3.251882	39.66878
13	7	0	8.975747	14.925656	36.702051
14	6	0	16.184406	-0.124486	35.452766
15	1	0	16.588844	-0.478133	33.438972
16	1	0	14.12726	-0.10576	35.764717
17	1	0	17.054155	-1.617719	36.60207
18	6	0	16.486472	4.333448	35.006014
19	6	0	18.085053	5.637834	33.354131
20	1	0	18.577896	9.050566	31.136403
21	6	0	17.328433	7.955482	32.357537
22	1	0	19.946407	4.844383	32.952118
23	6	0	14.94794	8.952916	32.974487
24	6	0	14.298794	11.540433	32.091896
25	6	0	11.318118	14.849143	32.415629
26	1	0	12.951128	15.963611	33.066891
27	6	0	8.967686	15.581416	33.98989

28	1	0	7.288126	14.730241	33.109247
29	6	0	8.905922	18.435289	34.045703
30	8	0	8.210694	19.277176	29.638045
31	6	0	8.535515	19.979791	32.088428
32	1	0	8.430295	22.02572	32.334388
33	6	0	9.594395	19.3028	36.673943
34	1	0	11.682835	19.28029	36.880313
35	6	0	8.602186	17.105281	38.260431
36	6	0	10.73256	15.361631	29.674496
37	1	0	8.992456	17.184177	26.579365
38	6	0	9.324566	17.187275	28.616125
39	1	0	11.504485	13.989346	28.343847
40	6	0	14.090516	5.319006	35.580179
41	6	0	12.111559	5.419708	39.468778
42	6	0	9.916657	6.841829	39.60455
43	1	0	8.590512	6.6941	38.044813
44	6	0	9.47134	8.463535	41.646119
45	6	0	7.110501	10.121235	41.683246
46	1	0	6.549539	10.42234	43.659019
47	8	0	5.022078	8.982237	40.503625
48	6	0	7.485725	12.815248	40.49966
49	6	0	8.184952	12.666385	37.669497
50	6	0	8.858654	14.516969	44.72295
51	1	0	9.921988	12.956174	45.564364
52	1	0	6.860758	14.411086	45.30047
53	1	0	9.672423	16.302926	45.382845
54	6	0	9.689168	16.800848	40.869026
55	6	0	11.235835	8.44125	43.626286
56	1	0	14.784193	6.976276	45.072304
57	6	0	13.420975	6.96831	43.523936
58	1	0	10.905035	9.565021	45.321695
59	6	0	13.936749	5.468813	41.401963
60	6	0	13.307242	7.61149	34.558992
61	8	0	10.838057	18.497327	41.917952
62	1	0	5.380192	8.97681	38.699516

**12a**000021_en_		Standard Orientation (A.U.)			
Center number	Atomic number	Atomic Type	X	Y	Z
0	16	0	4.326536	14.185945	40.554957
1	16	0	5.371102	17.833703	39.199326
2	7	0	9.497255	14.324808	41.727918
3	8	0	17.284163	2.316572	36.343128
4	8	0	11.586536	12.013839	32.584941
5	8	0	8.728224	21.48646	37.093043
6	1	0	9.584973	21.930897	38.651343
7	8	0	15.283313	12.758613	30.601167
8	8	0	12.675519	3.9222	37.293953
9	1	0	11.309758	8.186033	34.907301

10	8	0	7.4795	10.634802	36.396581
11	8	0	15.574119	3.222195	41.52265
12	1	0	15.737089	2.559385	39.806331
13	7	0	8.802858	14.737995	36.569621
14	6	0	19.770285	1.380691	36.008879
15	1	0	19.94858	-0.265493	37.25741
16	1	0	21.196873	2.806506	36.538569
17	1	0	20.090502	0.784448	34.037401
18	6	0	16.552323	4.409364	35.054108
19	6	0	18.070085	5.74753	33.342216
20	1	0	18.362359	9.07873	30.982188
21	6	0	17.173221	7.977358	32.256975
22	1	0	19.972374	5.095493	32.899051
23	6	0	14.75178	8.872984	32.835728
24	6	0	13.959307	11.374898	31.850344
25	6	0	10.883046	14.587312	32.162128
26	1	0	12.53342	15.752292	32.662045
27	6	0	8.627301	15.351666	33.861957
28	1	0	6.892835	14.503482	33.094352
29	6	0	8.613039	18.203651	33.872135
30	8	0	7.594847	18.932518	29.513178
31	6	0	8.124074	19.705478	31.908041
32	1	0	8.082576	21.759873	32.096086
33	6	0	9.579196	19.091325	36.405559
34	1	0	11.676015	19.010843	36.402857
35	6	0	8.686302	16.950649	38.130182
36	6	0	10.103725	15.008723	29.454982
37	1	0	8.127107	16.756338	26.456812
38	6	0	8.620447	16.811085	28.459831
39	1	0	10.766766	13.588029	28.116062
40	6	0	14.08792	5.287401	35.581661
41	6	0	12.04011	5.207193	39.49203
42	6	0	10.014704	6.862733	39.561481
43	1	0	8.861166	7.068991	37.878778
44	6	0	9.473947	8.253968	41.748077
45	6	0	7.244399	10.074551	41.809125
46	1	0	6.823614	10.489352	43.79919
47	8	0	5.017741	9.015827	40.812315
48	6	0	7.67091	12.705848	40.511376
49	6	0	8.03246	12.512155	37.623005
50	6	0	9.993399	14.142474	44.437547
51	1	0	8.201228	14.080716	45.49516
52	1	0	11.041345	15.845977	44.97466
53	1	0	11.138053	12.476579	44.879813
54	6	0	10.0804	16.595996	40.582889
55	6	0	10.937998	7.765009	43.905485
56	1	0	14.091794	5.708368	45.543615
57	6	0	12.958795	6.066355	43.857223
58	1	0	10.477734	8.691	45.69204

59	6	0	13.582284	4.796669	41.619351
60	6	0	13.194157	7.487537	34.472855
61	8	0	11.438801	18.23108	41.469328
62	1	0	5.246176	8.91364	38.990156

**12a**000024_en_		Standard Orientation (A.U.)			
Center number	Atomic number	Atomic Type	X	Y	Z
0	16	0	4.030465	13.792047	40.331013
1	16	0	5.010743	17.567865	39.494796
2	7	0	9.160589	14.24139	41.819074
3	8	0	17.931963	2.685556	36.636846
4	8	0	11.899734	12.223756	32.89951
5	8	0	7.889384	21.498743	37.158312
6	1	0	6.127135	21.39368	36.663969
7	8	0	15.683718	13.241867	31.223644
8	8	0	13.064979	4.059272	37.406473
9	1	0	11.659803	8.359258	35.085489
10	8	0	7.692493	10.554372	36.359531
11	8	0	16.081224	3.80078	41.711294
12	1	0	16.493849	3.21323	40.007675
13	7	0	8.618665	14.758655	36.658975
14	6	0	17.12183	0.291502	35.675155
15	1	0	17.750432	0.055178	33.702239
16	1	0	15.051423	0.107172	35.782055
17	1	0	18.01649	-1.157144	36.861726
18	6	0	17.018467	4.765921	35.387269
19	6	0	18.599588	6.229597	33.856269
20	1	0	18.921896	9.689658	31.678847
21	6	0	17.70073	8.4762	32.813764
22	1	0	20.548264	5.613012	33.576123
23	6	0	15.20046	9.240035	33.265947
24	6	0	14.351913	11.745377	32.321677
25	6	0	11.055308	14.742751	32.429159
26	1	0	12.574336	16.019609	33.053633
27	6	0	8.619096	15.352199	33.946878
28	1	0	7.015281	14.382883	33.04881
29	6	0	8.426053	18.188108	33.94166
30	8	0	7.807965	18.916777	29.491625
31	6	0	8.066139	19.672461	31.930667
32	1	0	7.935514	21.724917	32.117929
33	6	0	9.040391	19.20475	36.554021
34	1	0	11.092135	19.521893	36.766449
35	6	0	8.359362	16.935561	38.252222
36	6	0	10.466335	15.104218	29.665189
37	1	0	8.660707	16.747261	26.500561
38	6	0	8.994535	16.83156	28.53473
39	1	0	11.281185	13.694811	28.3998
40	6	0	14.509022	5.522982	35.803069

41	6	0	12.328624	5.307213	39.601048
42	6	0	10.160948	6.77765	39.619289
43	1	0	9.019606	6.839624	37.915104
44	6	0	9.48977	8.183024	41.756876
45	6	0	7.15658	9.868666	41.72633
46	1	0	6.591015	10.211836	43.694172
47	8	0	5.056575	8.72204	40.562117
48	6	0	7.510717	12.546266	40.50994
49	6	0	8.00494	12.450079	37.643815
50	6	0	9.460978	14.110313	44.561099
51	1	0	10.32487	15.893192	45.164213
52	1	0	10.704414	12.553009	45.119084
53	1	0	7.601316	13.902143	45.473861
54	6	0	9.795363	16.538821	40.692491
55	6	0	10.972224	7.874103	43.935613
56	1	0	14.303071	6.177823	45.628125
57	6	0	13.143875	6.377463	43.933334
58	1	0	10.418325	8.796044	45.696252
59	6	0	13.914991	5.124574	41.728447
60	6	0	13.589669	7.740237	34.733106
61	8	0	11.208209	18.086786	41.610065
62	1	0	5.421706	8.658238	38.760248

**12a**000026_en_		Standard Orientation (A.U.)			
Center number	Atomic number	Atomic Type	X	Y	Z
0	16	0	4.533024	12.970179	39.353246
1	16	0	4.960251	16.881749	38.631573
2	7	0	9.334421	14.084794	41.460676
3	8	0	15.481472	1.933863	35.358543
4	8	0	12.38991	12.740581	31.759934
5	8	0	7.871584	21.259946	37.193905
6	1	0	8.535759	21.660768	38.85611
7	8	0	14.943941	13.874878	34.981136
8	8	0	16.219954	5.4108	38.985385
9	1	0	16.189501	10.122448	37.452446
10	8	0	9.203346	10.377522	35.838868
11	8	0	17.017082	4.355645	43.844549
12	1	0	17.94578	4.033515	42.295225
13	7	0	9.152244	14.687543	36.20857
14	6	0	14.822881	0.028476	33.608014
15	1	0	15.041778	-1.779428	34.601074
16	1	0	16.079175	0.048267	31.941512
17	1	0	12.845315	0.223878	32.971123
18	6	0	15.338728	4.371109	34.6493
19	6	0	14.602513	5.223821	32.239796
20	1	0	13.502598	8.432531	29.974896
21	6	0	14.27488	7.799693	31.777491
22	1	0	14.15215	3.867357	30.757415

23	6	0	14.873821	9.574898	33.64383
24	6	0	14.169369	12.278082	33.536127
25	6	0	11.32188	15.219174	31.783825
26	1	0	12.778173	16.516322	32.494457
27	6	0	8.961291	15.470001	33.523362
28	1	0	7.394326	14.419506	32.649278
29	6	0	8.473345	18.275332	33.741567
30	8	0	7.458666	19.273818	29.422569
31	6	0	7.795241	19.862626	31.905312
32	1	0	7.410839	21.851918	32.293522
33	6	0	9.136639	19.095652	36.388682
34	1	0	11.216553	19.329391	36.533399
35	6	0	8.490311	16.710695	37.866111
36	6	0	10.522887	15.788862	29.118879
37	1	0	8.379562	17.489259	26.211142
38	6	0	8.811712	17.438223	28.228489
39	1	0	11.399134	14.57895	27.695046
40	6	0	15.899069	6.188288	36.52049
41	6	0	14.365417	6.183199	40.667933
42	6	0	12.16508	7.451683	39.986221
43	1	0	11.766796	7.857863	38.01457
44	6	0	10.42958	8.218357	41.833334
45	6	0	7.958155	9.507297	41.114496
46	1	0	6.888734	9.757762	42.877449
47	8	0	6.413122	8.00461	39.553283
48	6	0	8.105653	12.21348	39.929832
49	6	0	8.937662	12.27942	37.127447
50	6	0	9.476823	13.846726	44.208119
51	1	0	7.588354	13.449323	44.991114
52	1	0	10.143774	15.667351	44.935299
53	1	0	10.799237	12.355124	44.765611
54	6	0	9.62848	16.475364	40.461232
55	6	0	10.92048	7.594193	44.359987
56	1	0	13.502921	5.809517	47.015026
57	6	0	13.117594	6.29732	45.047944
58	1	0	9.56103	8.107823	45.827663
59	6	0	14.876812	5.59221	43.211638
60	6	0	15.808583	8.741427	35.972018
61	8	0	10.528443	18.286362	41.564532
62	1	0	7.185444	8.018962	37.881463

**12a**000028_en_		Standard Orientation (A.U.)			
Center number	Atomic number	Atomic Type	X	Y	Z
0	16	0	3.889706	13.645191	39.993017
1	16	0	4.82129	17.411891	38.878214
2	7	0	8.740053	14.238032	41.979183
3	8	0	17.682759	2.311379	36.33371
4	8	0	12.015512	12.174946	32.851558

5	8	0	8.180303	21.398044	37.388354
6	1	0	8.786376	21.834606	39.062771
7	8	0	15.825676	13.0204	31.144973
8	8	0	12.920402	3.925129	37.315672
9	1	0	11.643551	8.29884	35.068716
10	8	0	7.895287	10.498134	36.345983
11	8	0	16.330764	4.259507	41.321243
12	1	0	16.540739	3.336922	39.734466
13	7	0	8.85003	14.695108	36.71784
14	6	0	16.664206	-0.05412	35.51268
15	1	0	14.603269	-0.11873	35.792093
16	1	0	17.574343	-1.521855	36.663512
17	1	0	17.113643	-0.374058	33.502811
18	6	0	16.806232	4.415736	35.100733
19	6	0	18.392892	5.79792	33.501466
20	1	0	18.810417	9.253809	31.335919
21	6	0	17.573818	8.09734	32.512748
22	1	0	20.291909	5.079121	33.13706
23	6	0	15.143394	8.997169	33.08308
24	6	0	14.421832	11.571798	32.219655
25	6	0	11.307275	14.758126	32.494453
26	1	0	12.878514	15.925557	33.202055
27	6	0	8.889937	15.381452	34.012765
28	1	0	7.268439	14.476593	33.078288
29	6	0	8.715132	18.230345	34.097009
30	8	0	8.1199	19.107027	29.680534
31	6	0	8.344541	19.785807	32.148299
32	1	0	8.153222	21.822512	32.417692
33	6	0	9.285638	19.093961	36.75465
34	1	0	11.365556	19.154506	37.028407
35	6	0	8.334989	16.839722	38.285022
36	6	0	10.775793	15.281813	29.744227
37	1	0	9.056146	17.077935	26.622297
38	6	0	9.333151	17.069129	28.667221
39	1	0	11.630087	13.951918	28.420806
40	6	0	14.361382	5.302838	35.627132
41	6	0	12.295067	5.30643	39.465456
42	6	0	10.026688	6.611271	39.571735
43	1	0	8.724512	6.383648	38.001004
44	6	0	9.4799	8.225127	41.594652
45	6	0	7.039489	9.765213	41.58972
46	1	0	6.411383	10.013878	43.55265
47	8	0	5.040433	8.545536	40.33882
48	6	0	7.325384	12.485893	40.446929
49	6	0	8.124923	12.395024	37.640111
50	6	0	8.440326	14.219926	44.729302
51	1	0	9.480469	12.669535	45.616778
52	1	0	6.418483	14.08359	45.21156
53	1	0	9.197074	16.01349	45.434557

54	6	0	9.348199	16.553561	40.925238
55	6	0	11.230406	8.319672	43.585037
56	1	0	14.837124	7.055491	45.073444
57	6	0	13.487848	6.958425	43.515931
58	1	0	10.837761	9.449957	45.26241
59	6	0	14.096433	5.463882	41.415408
60	6	0	13.516533	7.57611	34.611489
61	8	0	10.394915	18.283669	42.024295
62	1	0	5.451879	8.579032	38.546197

**12a**000030_en_		Standard Orientation (A.U.)			
Center number	Atomic number	Atomic Type	X	Y	Z
0	16	0	4.156129	12.561352	38.911364
1	16	0	4.33661	16.493588	38.00572
2	7	0	8.430783	14.065604	41.651516
3	8	0	16.071953	2.026316	36.021661
4	8	0	13.617695	13.280991	33.419943
5	8	0	6.859303	21.208234	37.228266
6	1	0	6.984479	21.667171	38.998624
7	8	0	11.693877	11.522348	30.023043
8	8	0	16.397203	6.255286	38.777624
9	1	0	15.405429	10.464015	36.357771
10	8	0	9.331668	10.625658	35.857079
11	8	0	17.453327	5.50721	43.655486
12	1	0	18.350904	5.185773	42.087347
13	7	0	9.158208	14.888219	36.519948
14	6	0	15.714418	-0.288465	34.738662
15	1	0	16.250847	-1.785362	36.070658
16	1	0	16.921122	-0.420617	33.041104
17	1	0	13.721891	-0.552917	34.178035
18	6	0	15.502083	4.200697	34.830087
19	6	0	14.606893	4.387748	32.334021
20	1	0	13.033445	6.854749	29.475844
21	6	0	13.881896	6.724473	31.351246
22	1	0	14.373737	2.693515	31.18711
23	6	0	14.162874	8.932189	32.779186
24	6	0	13.021126	11.326461	31.868264
25	6	0	12.472372	15.709492	33.09985
26	1	0	13.549234	16.875149	34.445886
27	6	0	9.667325	15.801009	33.923182
28	1	0	8.538956	14.703368	32.563955
29	6	0	8.900461	18.54519	34.081375
30	8	0	9.347904	19.771708	29.734803
31	6	0	8.708308	20.193944	32.182705
32	1	0	7.985934	22.103048	32.49132
33	6	0	8.604479	19.275787	36.819443
34	1	0	10.483719	19.799333	37.589703
35	6	0	7.916301	16.754815	38.042714

36	6	0	12.762594	16.681642	30.440058
37	1	0	11.805529	18.615205	27.088168
38	6	0	11.353083	18.285459	29.07434
39	1	0	14.337348	15.849099	29.400546
40	6	0	15.783034	6.442838	36.250822
41	6	0	14.514652	6.860084	40.493522
42	6	0	12.156079	7.802311	39.840724
43	1	0	11.66771	8.058459	37.868876
44	6	0	10.374111	8.423297	41.700473
45	6	0	7.763802	9.383416	40.937462
46	1	0	6.58719	9.396472	42.648456
47	8	0	6.516607	7.777578	39.226015
48	6	0	7.658404	12.147133	39.876682
49	6	0	8.87161	12.427349	37.231129
50	6	0	8.14825	13.703119	44.375033
51	1	0	8.406003	15.558138	45.258208
52	1	0	9.571425	12.392849	45.108929
53	1	0	6.23758	13.000027	44.810913
54	6	0	8.559443	16.521923	40.805189
55	6	0	10.995731	7.956628	44.231005
56	1	0	13.848626	6.609956	46.868521
57	6	0	13.3645	6.98553	44.898517
58	1	0	9.609879	8.307794	45.720754
59	6	0	15.161167	6.445618	43.042389
60	6	0	15.219801	8.773118	35.201939
61	8	0	8.994184	18.36979	42.111734
62	1	0	7.336839	8.001777	37.593257

Table S19. Gibbs free energies[a] and equilibrium populations[b] of low-energy conformers of 12b.

Conformers	ΔG(a.u.)	P(%)/100	G(a.u.)
**12b**2000002.out	0.00735	0.01	-2699.999995
**12b**2000003.out	4e-05	24.85	-2700.0073
**12b**2000005.out	2e-05	25.5	-2700.007324
**12b**2000009.out	0.00031	18.78	-2700.007035
**12b**2000010.out	0.00417	0.31	-2700.003168
**12b**2000012.out	0.0022	2.53	-2700.005142
**12b**2000014.out	0.0086	0.0	-2699.998738
**12b**2000016.out	0.0073	0.01	-2700.000043
**12b**2000018.out	0.0	25.98	-2700.007342
**12b**2000019.out	0.00241	2.02	-2700.00493

[a]wB97M-V/def2-TZVP, in a.u.
[b]From ΔG values at 298.15 K.

Table S20. Cartesian coordinates for the low-energy reoptimized random research conformers of 12b at B3LYP-D3(BJ)/6-31G* level of theory in chloroform.

**12b**000002_en_		Standard Orientation (A.U.)			
Center number	Atomic number	Atomic Type	X	Y	Z
0	16	0	16.44712	13.545697	40.783714
1	16	0	16.30272	17.294243	39.279898
2	7	0	11.548789	15.051999	42.128712
3	8	0	14.953941	1.265183	34.20544
4	8	0	12.387561	12.513152	31.719558
5	8	0	11.861034	21.850728	37.764729
6	1	0	10.907849	21.487849	39.31374
7	8	0	16.282459	13.192518	33.32258
8	8	0	17.566348	4.444791	37.130509
9	1	0	17.594399	9.217778	35.61605
10	8	0	11.121673	10.897775	37.226067
11	8	0	21.02154	3.993214	40.783332
12	1	0	20.856042	3.316445	39.085088
13	7	0	12.160335	15.069179	36.932775
14	6	0	13.408728	-0.469875	32.892371
15	1	0	11.387416	0.02228	33.061229
16	1	0	13.732126	-2.322381	33.767924
17	1	0	13.914295	-0.574431	30.870029
18	6	0	14.895626	3.721681	33.542892
19	6	0	13.427315	4.739252	31.574383
20	1	0	12.071493	8.13108	29.760318
21	6	0	13.350513	7.344848	31.171681
22	1	0	12.250601	3.504677	30.420768
23	6	0	14.880949	8.958823	32.602031
24	6	0	14.683576	11.753053	32.556368
25	6	0	11.775384	15.130115	32.02887
26	1	0	13.524114	16.233515	31.806736
27	6	0	10.639493	15.680793	34.670488
28	1	0	8.861136	14.610217	34.798632
29	6	0	10.290001	18.483967	34.908474
30	8	0	7.118001	19.110479	31.730414
31	6	0	8.669111	19.919044	33.619789
32	1	0	8.474503	21.938679	33.999595
33	6	0	12.331234	19.550772	36.619886
34	1	0	14.034756	19.784658	35.427251
35	6	0	12.904968	17.274068	38.389605
36	6	0	9.814555	15.697861	30.053745
37	1	0	6.499275	17.262163	28.48271
38	6	0	7.827121	17.278904	30.063141
39	1	0	9.9512	14.496356	28.380512
40	6	0	16.383793	5.383287	35.006294
41	6	0	17.086856	5.710656	39.368443
42	6	0	14.966875	7.201531	39.786349

43	1	0	13.540838	7.396322	38.31963
44	6	0	14.736081	8.582376	42.033526
45	6	0	12.587277	10.446947	42.443377
46	1	0	12.707312	10.982442	44.441181
47	8	0	10.144834	9.492958	42.067758
48	6	0	12.922408	12.981755	40.940728
49	6	0	12.004914	12.817638	38.168375
50	6	0	10.471872	14.878451	44.664187
51	1	0	9.203577	13.242035	44.78547
52	1	0	9.407009	16.624654	44.986696
53	1	0	11.969754	14.736305	46.103199
54	6	0	11.458313	17.314485	40.891903
55	6	0	16.604254	8.308778	43.894198
56	1	0	20.158623	6.532774	44.946838
57	6	0	18.694985	6.742768	43.508898
58	1	0	16.445419	9.359252	45.664896
59	6	0	18.97745	5.450647	41.224752
60	6	0	16.474254	7.940569	34.449428
61	8	0	10.360811	19.190655	41.697234
62	1	0	9.931905	9.300499	40.248289

**12b**000003_en_		Standard Orientation (A.U.)			
Center number	Atomic number	Atomic Type	X	Y	Z
0	16	0	15.650963	13.574545	40.481743
1	16	0	15.630029	17.371086	39.030967
2	7	0	11.200163	15.469986	42.640582
3	8	0	18.094481	1.624957	34.670153
4	8	0	13.191689	12.333944	33.141425
5	8	0	9.027318	21.036698	38.013846
6	1	0	9.350529	21.405873	39.79682
7	8	0	16.483433	12.834124	30.478924
8	8	0	14.341963	3.869987	37.104663
9	1	0	13.075951	8.442516	35.383807
10	8	0	9.847618	11.303866	37.774783
11	8	0	18.637054	3.095233	39.897865
12	1	0	18.010039	2.24733	38.382471
13	7	0	11.135032	15.413474	37.367879
14	6	0	20.183636	0.368716	33.563038
15	1	0	20.404959	-1.416909	34.594253
16	1	0	21.930332	1.493131	33.746149
17	1	0	19.834004	-0.041427	31.549522
18	6	0	17.402739	3.92635	33.771139
19	6	0	18.600574	5.200776	31.778404
20	1	0	18.836112	8.697996	29.667058
21	6	0	17.858392	7.651118	31.150363
22	1	0	20.161709	4.320944	30.76412
23	6	0	15.877577	8.827332	32.452771
24	6	0	15.270301	11.501586	31.884988

25	6	0	12.581538	14.934315	32.739925
26	1	0	14.328223	16.029396	32.993621
27	6	0	10.591066	15.880329	34.691315
28	1	0	8.757121	15.01583	34.241804
29	6	0	10.626763	18.729943	34.537963
30	8	0	9.719865	19.538808	30.109479
31	6	0	10.262481	20.20758	32.518239
32	1	0	10.310537	22.26292	32.705388
33	6	0	11.224038	19.900761	37.078551
34	1	0	12.803594	21.268231	36.957559
35	6	0	12.075554	17.583097	38.688265
36	6	0	11.489274	15.262247	30.129506
37	1	0	9.485941	17.109185	27.205261
38	6	0	10.220414	17.210469	29.130198
39	1	0	11.681291	13.630653	28.883956
40	6	0	15.383773	5.108842	35.049382
41	6	0	14.678202	5.23408	39.326114
42	6	0	12.952799	7.061812	40.056079
43	1	0	11.232914	7.298599	38.956772
44	6	0	13.494617	8.657168	42.093376
45	6	0	11.752496	10.812163	42.827013
46	1	0	12.268633	11.300315	44.772467
47	8	0	9.168649	10.215153	42.86291
48	6	0	12.126452	13.294172	41.266955
49	6	0	10.89224	13.182614	38.621512
50	6	0	10.689327	15.426275	45.353529
51	1	0	9.291417	13.95703	45.784523
52	1	0	9.938937	17.289862	45.852453
53	1	0	12.436768	15.104846	46.439753
54	6	0	11.219688	17.745428	41.437231
55	6	0	15.734111	8.248082	43.455743
56	1	0	19.181183	6.047092	43.827863
57	6	0	17.436356	6.3528	42.771013
58	1	0	16.168404	9.445968	45.08201
59	6	0	16.952469	4.842087	40.653911
60	6	0	14.609769	7.519396	34.372189
61	8	0	10.72645	19.761552	42.463779
62	1	0	8.65394	9.99565	41.111175

**12b**000005_en_		Standard Orientation (A.U.)			
Center number	Atomic number	Atomic Type	X	Y	Z
0	16	0	15.342174	13.715929	40.596873
1	16	0	15.417571	17.481142	39.064201
2	7	0	10.71603	15.617167	42.337661
3	8	0	18.337615	1.64787	35.273764
4	8	0	13.526272	12.29675	33.119731
5	8	0	8.852233	21.015497	37.420988
6	1	0	9.015362	21.441082	39.212904

7	8	0	17.020128	12.751018	30.720673
8	8	0	14.398547	3.918084	37.354118
9	1	0	13.253664	8.45345	35.442704
10	8	0	9.80896	11.339642	37.458752
11	8	0	18.425249	3.232203	40.537116
12	1	0	17.941334	2.353399	38.987441
13	7	0	11.090912	15.452578	37.078175
14	6	0	20.506104	0.374107	34.356177
15	1	0	20.308269	-0.085242	32.332564
16	1	0	20.653837	-1.385644	35.443524
17	1	0	22.232547	1.508612	34.64119
18	6	0	17.709848	3.924844	34.272878
19	6	0	19.053614	5.161968	32.350889
20	1	0	19.438471	8.613468	30.186616
21	6	0	18.353281	7.594052	31.613227
22	1	0	20.691647	4.268846	31.479477
23	6	0	16.273942	8.788357	32.732914
24	6	0	15.702477	11.44517	32.056222
25	6	0	12.945202	14.884995	32.609466
26	1	0	14.658269	15.994419	32.995896
27	6	0	10.784416	15.859321	34.353605
28	1	0	9.001278	14.973946	33.762975
29	6	0	10.82225	18.706189	34.144175
30	8	0	10.354193	19.433685	29.632172
31	6	0	10.657131	20.144679	32.070351
32	1	0	10.685292	22.203163	32.224313
33	6	0	11.15549	19.928957	36.706817
34	1	0	12.704289	21.335588	36.708952
35	6	0	11.901359	17.656327	38.427464
36	6	0	12.091772	15.153922	29.905339
37	1	0	10.374819	16.942315	26.77015
38	6	0	10.929756	17.083113	28.751828
39	1	0	12.388778	13.494243	28.719119
40	6	0	15.592899	5.123949	35.364027
41	6	0	14.525815	5.334466	39.563784
42	6	0	12.732997	7.165509	40.096188
43	1	0	11.114273	7.361593	38.845314
44	6	0	13.084955	8.812949	42.133776
45	6	0	11.26887	10.967035	42.660384
46	1	0	11.599147	11.495635	44.635278
47	8	0	8.697051	10.348662	42.468539
48	6	0	11.765528	13.421419	41.090443
49	6	0	10.760316	13.246037	38.352825
50	6	0	9.987531	15.625626	45.000464
51	1	0	8.55555	14.166449	45.346711
52	1	0	9.203201	17.498932	45.401039
53	1	0	11.641083	15.323385	46.229448
54	6	0	10.820642	17.866981	41.091815
55	6	0	15.200662	8.454892	43.694554

56	1	0	18.613903	6.287574	44.41916
57	6	0	16.965549	6.555658	43.208407
58	1	0	15.485355	9.695675	45.321706
59	6	0	16.675183	4.989223	41.096422
60	6	0	14.865805	7.515726	34.576895
61	8	0	10.228825	19.901004	42.027316
62	1	0	8.351401	10.093088	40.680138

**12b**000009_en_		Standard Orientation (A.U.)			
Center number	Atomic number	Atomic Type	X	Y	Z
0	16	0	16.57396	13.170514	40.329146
1	16	0	16.811363	16.820003	38.613798
2	7	0	12.495083	15.75798	42.521922
3	8	0	16.579892	1.336401	34.175287
4	8	0	11.626077	12.040594	32.855695
5	8	0	12.760017	21.963293	37.320799
6	1	0	12.112062	21.879461	39.055701
7	8	0	13.853412	12.033139	29.226695
8	8	0	13.541711	3.870679	37.196984
9	1	0	11.998468	8.378233	35.452311
10	8	0	10.030081	11.533237	38.449608
11	8	0	18.16343	2.677596	39.221682
12	1	0	17.243101	1.957813	37.790428
13	7	0	12.00944	15.183288	37.31278
14	6	0	18.282978	-0.108223	32.697759
15	1	0	18.751607	-1.787505	33.820722
16	1	0	20.027199	0.958817	32.288577
17	1	0	17.402186	-0.70431	30.905331
18	6	0	15.703507	3.564315	33.25253
19	6	0	16.331523	4.574186	30.884639
20	1	0	15.966661	7.794383	28.38527
21	6	0	15.441355	6.95831	30.195586
22	1	0	17.556707	3.536297	29.595695
23	6	0	13.898352	8.343392	31.838348
24	6	0	13.153427	10.951469	31.115556
25	6	0	11.160755	14.693246	32.586545
26	1	0	12.945476	15.578762	31.982392
27	6	0	10.349614	15.756812	35.169527
28	1	0	8.465225	14.99636	35.605315
29	6	0	10.433514	18.583329	35.04224
30	8	0	7.086036	19.191148	32.050473
31	6	0	8.906168	20.038282	33.662867
32	1	0	9.008657	22.099126	33.726846
33	6	0	12.793501	19.522216	36.391779
34	1	0	14.34184	19.416267	34.987257
35	6	0	13.333909	17.354912	38.324753
36	6	0	9.065268	15.175132	30.723801
37	1	0	5.832585	16.983923	29.240409

38	6	0	7.331334	17.029838	30.658996
39	1	0	8.860442	13.746459	29.24986
40	6	0	14.100107	4.941386	34.882096
41	6	0	14.408311	5.220637	39.273067
42	6	0	13.01359	7.189495	40.283373
43	1	0	11.167711	7.596195	39.482761
44	6	0	14.01587	8.68376	42.225091
45	6	0	12.672369	11.055329	43.134719
46	1	0	13.539224	11.562739	44.949905
47	8	0	10.073926	10.785238	43.581078
48	6	0	13.13504	13.364808	41.342124
49	6	0	11.580406	13.192887	38.880949
50	6	0	12.15712	16.021179	45.248086
51	1	0	11.578605	17.977348	45.602767
52	1	0	13.947598	15.65739	46.247173
53	1	0	10.689308	14.715144	45.911246
54	6	0	12.472818	17.875791	41.039636
55	6	0	16.378506	8.012781	43.228971
56	1	0	19.593797	5.464508	43.046049
57	6	0	17.75296	5.97487	42.267716
58	1	0	17.178597	9.119811	44.778622
59	6	0	16.809683	4.575541	40.228671
60	6	0	13.203966	7.303296	34.178401
61	8	0	11.893515	19.984113	41.804083
62	1	0	9.278775	10.578175	41.935115

**12b**000010_en_		Standard Orientation (A.U.)			
Center number	Atomic number	Atomic Type	X	Y	Z
0	16	0	15.681486	13.51864	41.143476
1	16	0	16.025855	17.200246	39.520701
2	7	0	10.905763	15.649604	42.044492
3	8	0	18.598722	1.738694	35.404212
4	8	0	13.224453	11.983825	32.589053
5	8	0	12.002726	21.918858	37.032816
6	1	0	10.900388	21.742038	38.512826
7	8	0	16.461531	12.243819	29.835631
8	8	0	14.475523	3.970218	37.429722
9	1	0	13.124365	8.366882	35.237149
10	8	0	10.173909	11.239312	37.500927
11	8	0	18.674769	3.413492	40.567501
12	1	0	18.413422	2.662764	38.896547
13	7	0	11.993258	15.099176	36.922327
14	6	0	17.193189	-0.518707	34.916273
15	1	0	18.151426	-2.0476	35.941761
16	1	0	17.196154	-0.94458	32.876197
17	1	0	15.233969	-0.337968	35.59536
18	6	0	17.686005	3.894829	34.285441
19	6	0	18.929003	4.981411	32.222847

20	1	0	19.089583	8.216741	29.70854
21	6	0	18.109274	7.298058	31.272815
22	1	0	20.576536	4.015974	31.44218
23	6	0	16.019787	8.516588	32.361647
24	6	0	15.306086	11.071628	31.420308
25	6	0	12.612297	14.58799	32.162818
26	1	0	14.404797	15.645048	32.104712
27	6	0	10.979267	15.526595	34.378639
28	1	0	9.137058	14.571378	34.261657
29	6	0	10.805987	18.34867	34.234088
30	8	0	8.420656	18.645234	30.39585
31	6	0	9.596834	19.656943	32.451401
32	1	0	9.458357	21.715828	32.508928
33	6	0	12.56182	19.508805	36.194433
34	1	0	14.452814	19.588992	35.30016
35	6	0	12.741911	17.381446	38.23549
36	6	0	11.125846	14.89372	29.757223
37	1	0	8.302616	16.381605	27.362082
38	6	0	9.29938	16.555125	29.161401
39	1	0	11.524684	13.486254	28.302657
40	6	0	15.577107	5.103948	35.351105
41	6	0	14.6669	5.386411	39.63641
42	6	0	12.836054	7.163961	40.221451
43	1	0	11.21639	7.340431	38.971692
44	6	0	13.135534	8.767311	42.303194
45	6	0	11.362073	10.981236	42.757338
46	1	0	11.616029	11.550034	44.736362
47	8	0	8.784923	10.45281	42.439955
48	6	0	12.09204	13.349887	41.13553
49	6	0	11.343361	13.051573	38.333643
50	6	0	9.787207	15.845119	44.56019
51	1	0	8.94945	17.73291	44.70651
52	1	0	11.24843	15.641675	46.029779
53	1	0	8.321607	14.398215	44.802871
54	6	0	11.09459	17.774632	40.584255
55	6	0	15.230832	8.379006	43.885671
56	1	0	18.691183	6.27198	44.546588
57	6	0	17.045424	6.54713	43.333903
58	1	0	15.476702	9.561124	45.56169
59	6	0	16.837976	5.063531	41.147702
60	6	0	14.733195	7.402602	34.390929
61	8	0	10.115086	19.803958	41.120815
62	1	0	8.523596	10.190528	40.638111

**12b**000012_en_		Standard Orientation (A.U.)			
Center number	Atomic number	Atomic Type	X	Y	Z
0	16	0	15.49874	13.576565	41.323152
1	16	0	15.899192	17.286145	39.775802

2	7	0	10.698598	15.695687	42.108076
3	8	0	18.570581	1.920451	35.508961
4	8	0	13.355322	12.20942	32.68328
5	8	0	11.948544	22.056978	37.25014
6	1	0	10.805212	21.851323	38.69496
7	8	0	16.688685	12.552171	30.056465
8	8	0	14.522362	4.107065	37.412433
9	1	0	13.220437	8.518672	35.274528
10	8	0	10.099947	11.379033	37.460629
11	8	0	18.38702	3.32262	40.783767
12	1	0	18.026362	2.538333	39.150331
13	7	0	11.94765	15.241841	37.015776
14	6	0	20.785517	0.674987	34.664214
15	1	0	21.007517	-1.000865	35.865358
16	1	0	22.465395	1.894339	34.862494
17	1	0	20.598075	0.074792	32.677055
18	6	0	17.852504	4.105337	34.37108
19	6	0	19.125654	5.250515	32.349109
20	1	0	19.314783	8.523878	29.900262
21	6	0	18.302102	7.58009	31.428651
22	1	0	20.790584	4.363329	31.523984
23	6	0	16.188792	8.771691	32.483211
24	6	0	15.488566	11.333975	31.574087
25	6	0	12.746386	14.816311	32.278386
26	1	0	14.533863	15.883326	32.301737
27	6	0	11.028381	15.713815	34.445675
28	1	0	9.193491	14.759113	34.241889
29	6	0	10.856688	18.537633	34.345595
30	8	0	8.611629	18.894317	30.429098
31	6	0	9.710452	19.873815	32.542138
32	1	0	9.566136	21.931302	32.628586
33	6	0	12.54112	19.663517	36.386817
34	1	0	14.46125	19.762937	35.558937
35	6	0	12.656019	17.497798	38.393186
36	6	0	11.347577	15.158523	29.825168
37	1	0	8.608833	16.681733	27.355067
38	6	0	9.539976	16.826479	29.191652
39	1	0	11.801376	13.775344	28.363601
40	6	0	15.694948	5.290906	35.401517
41	6	0	14.557388	5.475455	39.651517
42	6	0	12.726776	7.272405	40.165965
43	1	0	11.192474	7.52196	38.824922
44	6	0	12.922399	8.806817	42.314355
45	6	0	11.136768	11.015769	42.746631
46	1	0	11.329825	11.54826	44.74245
47	8	0	8.569931	10.492803	42.344371
48	6	0	11.913215	13.412259	41.193324
49	6	0	11.248895	13.16945	38.365037
50	6	0	9.499797	15.842765	44.589708

51	1	0	8.655566	17.726861	44.745636
52	1	0	10.913136	15.611215	46.101349
53	1	0	8.029067	14.390368	44.756695
54	6	0	10.937193	17.84856	40.697214
55	6	0	14.911271	8.34326	44.008494
56	1	0	18.267584	6.129281	44.847154
57	6	0	16.716085	6.47724	43.533177
58	1	0	15.073984	9.480518	45.725201
59	6	0	16.597788	5.046901	41.309589
60	6	0	14.866818	7.593628	34.458899
61	8	0	9.945128	19.869244	41.243288
62	1	0	8.361271	10.255256	40.532309

**12b**000014_en_		Standard Orientation (A.U.)			
Center number	Atomic number	Atomic Type	X	Y	Z
0	16	0	16.478693	13.464384	40.655963
1	16	0	16.297023	17.252793	39.262468
2	7	0	11.579726	14.891817	42.079702
3	8	0	14.450819	1.24683	33.671885
4	8	0	12.311554	12.622411	31.622547
5	8	0	11.804218	21.818663	37.916918
6	1	0	10.86269	21.400364	39.458823
7	8	0	16.253848	13.210847	33.144356
8	8	0	17.680831	4.479575	36.741684
9	1	0	17.70735	9.165854	35.180841
10	8	0	11.145338	10.881933	37.06246
11	8	0	21.129213	3.944225	40.375932
12	1	0	21.016228	3.403741	38.626671
13	7	0	12.152324	15.068568	36.881953
14	6	0	16.358804	-0.231909	34.843906
15	1	0	15.915273	-2.205148	34.373448
16	1	0	16.370297	0.002574	36.909158
17	1	0	18.246011	0.243516	34.099913
18	6	0	14.711159	3.75539	33.321029
19	6	0	13.097244	4.852229	31.504548
20	1	0	11.661729	8.275765	29.83058
21	6	0	13.039838	7.444485	31.116603
22	1	0	11.817831	3.58523	30.498173
23	6	0	14.750494	9.012089	32.408722
24	6	0	14.609839	11.809823	32.401292
25	6	0	11.735617	15.239409	31.99056
26	1	0	13.498355	16.32501	31.79145
27	6	0	10.613237	15.734478	34.646802
28	1	0	8.836734	14.658762	34.761588
29	6	0	10.258856	18.529865	34.95848
30	8	0	7.083854	19.241139	31.800287
31	6	0	8.632365	19.996441	33.712359
32	1	0	8.431436	22.003285	34.152361

33	6	0	12.293475	19.559748	36.700496
34	1	0	13.993048	19.846905	35.513729
35	6	0	12.891347	17.234686	38.398024
36	6	0	9.779909	15.874522	30.031186
37	1	0	6.469835	17.487588	28.499803
38	6	0	7.7944	17.456969	30.082798
39	1	0	9.916476	14.722888	28.323021
40	6	0	16.358637	5.364732	34.658235
41	6	0	17.183068	5.685018	39.014617
42	6	0	15.056584	7.15428	39.471919
43	1	0	13.629765	7.390407	38.012391
44	6	0	14.814625	8.453609	41.767644
45	6	0	12.651949	10.287344	42.244918
46	1	0	12.779454	10.762548	44.257513
47	8	0	10.217367	9.320976	41.855489
48	6	0	12.958007	12.869281	40.820258
49	6	0	12.022383	12.780898	38.051711
50	6	0	10.515368	14.632227	44.613223
51	1	0	9.44056	16.360504	44.994273
52	1	0	12.020187	14.45584	46.041333
53	1	0	9.258086	12.984425	44.689464
54	6	0	11.463821	17.189769	40.911314
55	6	0	16.679205	8.125594	43.621856
56	1	0	20.246354	6.341309	44.619246
57	6	0	18.781856	6.588262	43.188024
58	1	0	16.510501	9.113917	45.427062
59	6	0	19.073357	5.375541	40.863627
60	6	0	16.462045	7.933034	34.094909
61	8	0	10.359386	19.032609	41.780872
62	1	0	9.992067	9.186439	40.031913

**12b**000016_en_		Standard Orientation (A.U.)			
Center number	Atomic number	Atomic Type	X	Y	Z
0	16	0	16.493995	13.647719	40.694478
1	16	0	16.190627	17.39871	39.219813
2	7	0	11.524935	14.9165	42.017807
3	8	0	15.602203	1.360113	33.964996
4	8	0	12.563258	12.518215	31.591956
5	8	0	11.546295	21.764482	37.718826
6	1	0	10.604181	21.341395	39.259406
7	8	0	16.411827	13.345063	33.23817
8	8	0	18.02705	4.616327	36.966721
9	1	0	17.863381	9.405267	35.508319
10	8	0	11.320777	10.794467	37.071355
11	8	0	21.469413	4.267569	40.642591
12	1	0	21.342506	3.599106	38.937739
13	7	0	12.167389	15.012593	36.826199
14	6	0	14.171635	-0.427156	32.593531

15	1	0	14.717102	-0.466774	30.579272
16	1	0	12.124923	-0.043097	32.734303
17	1	0	14.575596	-2.276727	33.441399
18	6	0	15.437885	3.820849	33.335461
19	6	0	13.948293	4.797489	31.362294
20	1	0	12.462385	8.147987	29.573452
21	6	0	13.758436	7.402074	30.991138
22	1	0	12.842524	3.526552	30.178376
23	6	0	15.198988	9.064156	32.459017
24	6	0	14.882206	11.847789	32.442036
25	6	0	11.835404	15.103409	31.920328
26	1	0	13.537609	16.283545	31.733927
27	6	0	10.644493	15.575943	34.552892
28	1	0	8.915448	14.424331	34.65115
29	6	0	10.165666	18.358219	34.812811
30	8	0	7.010672	18.880966	31.598362
31	6	0	8.499168	19.734317	33.517821
32	1	0	8.210989	21.738984	33.917116
33	6	0	12.136959	19.500906	36.557149
34	1	0	13.83871	19.828416	35.383973
35	6	0	12.801598	17.234763	38.308961
36	6	0	9.876948	15.607456	29.925444
37	1	0	6.516734	17.040475	28.324984
38	6	0	7.821497	17.099196	29.923526
39	1	0	10.087362	14.43069	28.242327
40	6	0	16.83016	5.528862	34.839539
41	6	0	17.481843	5.84264	39.211659
42	6	0	15.300394	7.242644	39.62685
43	1	0	13.876866	7.390309	38.152412
44	6	0	14.99876	8.595034	41.882815
45	6	0	12.767817	10.360076	42.295136
46	1	0	12.850722	10.880578	44.298741
47	8	0	10.373053	9.301842	41.89328
48	6	0	12.997994	12.922172	40.819103
49	6	0	12.10761	12.743752	38.038466
50	6	0	10.446417	14.671303	44.546584
51	1	0	9.311661	16.369949	44.884454
52	1	0	11.943825	14.572103	45.989541
53	1	0	9.243314	12.984648	44.6455
54	6	0	11.340774	17.184884	40.802859
55	6	0	16.864704	8.382918	43.753772
56	1	0	20.479469	6.741173	44.818325
57	6	0	19.018778	6.904479	43.371341
58	1	0	16.651626	9.411405	45.531645
59	6	0	19.367792	5.642025	41.07974
60	6	0	16.813712	8.094392	34.313834
61	8	0	10.158206	19.002952	41.62108
62	1	0	10.180939	9.117904	40.070635

**12b**000018_en_		Standard Orientation (A.U.)			
Center number	Atomic number	Atomic Type	X	Y	Z
0	16	0	15.176203	13.547097	40.48646
1	16	0	15.296186	17.298094	38.923793
2	7	0	10.554848	15.50488	42.179606
3	8	0	18.061566	1.387426	35.275673
4	8	0	13.399424	12.079515	33.014067
5	8	0	8.770629	20.871443	37.203309
6	1	0	8.923121	21.314087	38.991889
7	8	0	16.915283	12.475664	30.63699
8	8	0	14.141452	3.728178	37.317556
9	1	0	13.065712	8.258714	35.364333
10	8	0	9.643694	11.194333	37.330147
11	8	0	18.152894	3.029399	40.522809
12	1	0	17.663183	2.137118	38.982713
13	7	0	10.964745	15.292594	36.924457
14	6	0	20.215385	0.075578	34.377724
15	1	0	21.956839	1.187209	34.661124
16	1	0	20.019617	-0.397069	32.356904
17	1	0	20.333162	-1.677697	35.479134
18	6	0	17.471414	3.665693	34.255279
19	6	0	18.841928	4.871807	32.332328
20	1	0	19.28402	8.302861	30.146064
21	6	0	18.17775	7.307512	31.573493
22	1	0	20.472405	3.950712	31.475872
23	6	0	16.107069	8.536418	32.671479
24	6	0	15.573292	11.195237	31.972927
25	6	0	12.849163	14.668714	32.476506
26	1	0	14.569668	15.764466	32.868904
27	6	0	10.683975	15.679377	34.19405
28	1	0	8.897071	14.806858	33.595712
29	6	0	10.751733	18.524012	33.961132
30	8	0	10.33026	19.217867	29.43946
31	6	0	10.619118	19.946543	31.874086
32	1	0	10.666185	22.005914	32.010458
33	6	0	11.072308	19.764224	36.516745
34	1	0	12.631028	21.159891	36.522221
35	6	0	11.78618	17.499548	38.261005
36	6	0	12.021512	14.923088	29.762693
37	1	0	10.350642	16.703633	26.598088
38	6	0	10.88952	16.854464	28.58348
39	1	0	12.312118	13.250374	28.593425
40	6	0	15.364309	4.900536	35.324857
41	6	0	14.277421	5.159807	39.516753
42	6	0	12.501453	7.014138	40.025596
43	1	0	10.889596	7.215449	38.766677
44	6	0	12.862266	8.677307	42.048464
45	6	0	11.065223	10.854218	42.54666

46	1	0	11.390763	11.396335	44.518514
47	8	0	8.487992	10.258773	42.346983
48	6	0	11.593472	13.289369	40.958603
49	6	0	10.605697	13.099825	38.215042
50	6	0	9.802773	15.542062	44.835491
51	1	0	8.356977	14.096412	45.181067
52	1	0	9.028645	17.424573	45.212353
53	1	0	11.442777	15.239139	46.082332
54	6	0	10.688688	17.742937	40.91546
55	6	0	14.968094	8.312231	43.621246
56	1	0	18.354902	6.114967	44.37991
57	6	0	16.714463	6.389776	43.159912
58	1	0	15.260604	9.565149	45.237638
59	6	0	16.417039	4.807556	41.060584
60	6	0	14.67179	7.295361	34.515957
61	8	0	10.107423	19.789977	41.828685
62	1	0	8.149077	9.994314	40.558548

**12b**000019_en_		Standard Orientation (A.U.)			
Center number	Atomic number	Atomic Type	X	Y	Z
0	16	0	15.588009	13.408429	40.953771
1	16	0	16.075261	17.047391	39.2666
2	7	0	10.91818	15.752857	41.872136
3	8	0	17.945391	1.498672	35.375753
4	8	0	12.965766	11.873512	32.450455
5	8	0	12.227768	21.906699	36.767472
6	1	0	11.137029	21.796672	38.262362
7	8	0	16.122025	11.968235	29.594748
8	8	0	14.062609	3.884228	37.380729
9	1	0	12.808186	8.263907	35.151331
10	8	0	9.930928	11.3311	37.399069
11	8	0	18.036141	3.03635	40.606426
12	1	0	17.576454	2.227687	39.010091
13	7	0	11.920956	15.093059	36.747565
14	6	0	20.081634	0.153979	34.481874
15	1	0	19.788432	-0.508631	32.527671
16	1	0	20.304839	-1.485336	35.732308
17	1	0	21.805145	1.324881	34.563754
18	6	0	17.245291	3.665857	34.1933
19	6	0	18.456543	4.694866	32.073371
20	1	0	18.629604	7.869619	29.496227
21	6	0	17.663527	7.015735	31.105501
22	1	0	20.051155	3.723356	31.20542
23	6	0	15.643385	8.315377	32.214067
24	6	0	14.981964	10.864332	31.24069
25	6	0	12.44399	14.488952	31.987065
26	1	0	14.273287	15.476343	31.876197
27	6	0	10.889171	15.532555	34.213529

28	1	0	9.004915	14.65915	34.13702
29	6	0	10.842521	18.356958	34.030339
30	8	0	8.434543	18.720219	30.208684
31	6	0	9.680409	19.696561	32.239157
32	1	0	9.643582	21.760325	32.270719
33	6	0	12.671821	19.463603	35.954831
34	1	0	14.553444	19.450758	35.037456
35	6	0	12.785622	17.356532	38.022004
36	6	0	10.924229	14.815663	29.604714
37	1	0	8.1357	16.399709	27.230937
38	6	0	9.176255	16.558514	29.006552
39	1	0	11.215402	13.355605	28.176881
40	6	0	15.178382	4.959029	35.278796
41	6	0	14.253019	5.316293	39.57164
42	6	0	12.521667	7.202812	40.108449
43	1	0	10.941254	7.479372	38.828112
44	6	0	12.871167	8.786548	42.200918
45	6	0	11.194617	11.078595	42.641105
46	1	0	11.49696	11.662192	44.608979
47	8	0	8.593979	10.648064	42.365891
48	6	0	11.997123	13.39326	40.980185
49	6	0	11.196949	13.09491	38.19275
50	6	0	9.821147	16.022822	44.390546
51	1	0	11.281926	15.790933	45.856412
52	1	0	8.309424	14.629245	44.659474
53	1	0	9.048398	17.939792	44.513482
54	6	0	11.186092	17.850449	40.385171
55	6	0	14.909516	8.286256	43.824412
56	1	0	18.204841	5.956674	44.587589
57	6	0	16.614415	6.33344	43.329243
58	1	0	15.191061	9.462703	45.498855
59	6	0	16.343402	4.84861	41.155176
60	6	0	14.381696	7.254751	34.292877
61	8	0	10.304607	19.927737	40.908243
62	1	0	8.296937	10.36673	40.57239

Table S21. Gibbs free energies[a] and equilibrium populations[b] of low-energy conformers of 13a.

Conformers	ΔG(a.u.)	P(%)/100	G(a.u.)
**13a**000001.log	0.0	24.23	-2436.229543
**13a**000002.log	0.00027	18.31	-2436.229278
**13a**000003.log	0.00026	18.35	-2436.22928
**13a**000004.log	0.00021	19.49	-2436.229337
**13a**000005.log	0.00475	0.16	-2436.224792
**13a**000006.log	0.00021	19.47	-2436.229336

[a]wB97M-V/def2-TZVP, in a.u.

[b]From ΔG values at 298.15 K.

Table S22. Cartesian coordinates for the low-energy reoptimized random research conformers of 13a at B3LYP-D3(BJ)/6-31G* level of theory in methanol.

**13a**000001_en_		Standard Orientation (A.U.)			
Center number	Atomic number	Atomic Type	X	Y	Z
0	7	0	2.748375	-1.706919	0.687064
1	6	0	0.225636	-1.119159	0.664845
2	6	0	-0.984206	-1.79475	-1.899164
3	7	0	-0.484569	-4.399114	-2.512698
4	6	0	1.955927	-5.279368	-2.172224
5	6	0	3.776517	-3.222704	-1.292997
6	6	0	6.289727	-4.175406	-0.237276
7	6	0	6.955931	-2.229896	1.762474
8	6	0	4.667922	-0.631896	2.363304
9	6	0	9.296851	-1.924035	2.646046
10	8	0	10.173372	-0.207112	4.362884
11	6	0	9.066868	2.088773	4.804232
12	6	0	7.080623	3.203784	3.710701
13	6	0	5.283736	2.18389	1.773493
14	8	0	2.646057	-7.427973	-2.636142
15	8	0	-0.913497	-0.151126	2.406812
16	6	0	-0.303766	5.765483	-0.214712
17	6	0	-1.984143	5.124576	1.732216
18	6	0	-4.561672	5.523033	1.450514
19	6	0	-5.48684	6.565398	-0.795673
20	6	0	-3.819942	7.271093	-2.72939
21	6	0	-1.242992	6.874089	-2.427766
22	6	0	-3.750336	-0.973851	-2.028284
23	6	0	-5.555178	-1.999043	-0.050114
24	6	0	-5.215178	-4.282792	1.240495
25	6	0	-6.986538	-5.14444	2.983331
26	6	0	-9.154525	-3.721378	3.471116
27	6	0	-9.519915	-1.429576	2.210472
28	6	0	-7.727682	-0.587434	0.477016
29	8	0	-8.0456	6.815984	-1.015302
30	6	0	-2.13775	-5.78697	-4.238083
31	8	0	2.969083	3.596541	1.882209
32	6	0	2.410279	5.094765	-0.14558
33	8	0	3.95821	5.694205	-1.729049
34	1	0	4.009837	-0.771595	4.314825
35	8	0	-10.83661	-4.652737	5.199602
36	16	0	4.263648	-1.314386	-4.32283
37	16	0	0.688981	0.460506	-4.24148
38	1	0	7.730342	-4.390937	-1.700336
39	1	0	5.946232	-6.055455	0.563502
40	1	0	10.823834	-3.170196	2.080271
41	1	0	10.149256	3.058339	6.247932
42	1	0	6.645487	5.090924	4.384405

43	1	0	6.016925	2.332622	-0.148843
44	1	0	-1.272218	4.23261	3.425532
45	1	0	-5.884343	4.981199	2.918604
46	1	0	-4.547956	8.092318	-4.468345
47	1	0	0.074276	7.348596	-3.924029
48	1	0	-3.745351	1.088517	-1.868241
49	1	0	-4.446144	-1.405938	-3.930108
50	1	0	-3.522186	-5.396698	0.932483
51	1	0	-6.706248	-6.907007	3.992602
52	1	0	-11.191305	-0.294027	2.594927
53	1	0	-8.022914	1.216186	-0.461073
54	1	0	-8.434574	7.564545	-2.643173
55	1	0	-2.207868	-4.836352	-6.0775
56	1	0	-1.328199	-7.664892	-4.467912
57	1	0	-4.038939	-5.938754	-3.453367
58	1	0	-12.220878	-3.465084	5.374166

**13a**000002_en_		Standard Orientation (A.U.)			
Center number	Atomic number	Atomic Type	X	Y	Z
0	7	0	-1.930351	-2.494641	-0.646091
1	6	0	0.19557	-1.053801	-0.880135
2	6	0	1.851691	-1.17891	1.516397
3	7	0	2.288919	-3.746509	2.277902
4	6	0	0.268722	-5.402545	2.221786
5	6	0	-2.206672	-4.132528	1.480777
6	6	0	-4.326694	-5.949953	0.735185
7	6	0	-5.733315	-4.545981	-1.33368
8	6	0	-4.242627	-2.250515	-2.141022
9	6	0	-8.046329	-5.177185	-2.109072
10	8	0	-9.539487	-3.982041	-3.845898
11	6	0	-9.429911	-1.439293	-4.319437
12	6	0	-7.980514	0.368135	-3.310406
13	6	0	-5.823793	0.136032	-1.485219
14	8	0	0.377851	-7.621137	2.835487
15	8	0	0.7009	0.236784	-2.723327
16	6	0	-2.360655	5.970736	0.037041
17	6	0	-0.490388	5.913674	-1.843479
18	6	0	1.408939	7.723282	-1.878229
19	6	0	1.448461	9.624043	-0.036905
20	6	0	-0.417595	9.70023	1.840586
21	6	0	-2.30453	7.877686	1.871118
22	6	0	4.211888	0.499169	1.2808
23	6	0	6.436424	-0.559294	-0.190714
24	6	0	8.824519	-0.595394	0.959128
25	6	0	10.955179	-1.489403	-0.292713
26	6	0	10.727239	-2.387743	-2.765216
27	6	0	8.366922	-2.358067	-3.94891
28	6	0	6.250661	-1.443735	-2.676442

29	8	0	3.354225	11.350283	-0.160418
30	6	0	4.41738	-4.392858	3.924695
31	8	0	-4.236954	2.323418	-1.702601
32	6	0	-4.365921	4.036803	0.224053
33	8	0	-5.952144	3.942428	1.881229
34	1	0	-3.745648	-2.20301	-4.143012
35	8	0	12.864118	-3.262279	-3.921819
36	16	0	-3.029288	-2.265803	4.463465
37	16	0	-0.28123	0.587878	3.944099
38	1	0	-5.51897	-6.46375	2.340956
39	1	0	-3.434155	-7.692642	0.059416
40	1	0	-8.990837	-6.862758	-1.42177
41	1	0	-10.867062	-0.980518	-5.705133
42	1	0	-8.343839	2.270253	-3.984635
43	1	0	-6.450788	0.017599	0.475068
44	1	0	-0.506329	4.414856	-3.234025
45	1	0	2.876652	7.698081	-3.309296
46	1	0	-0.378057	11.177707	3.270356
47	1	0	-3.752389	7.888141	3.321563
48	1	0	3.557575	2.26337	0.419401
49	1	0	4.854583	0.961513	3.189001
50	1	0	9.021921	0.090677	2.886301
51	1	0	12.79493	-1.50831	0.612021
52	1	0	8.179857	-3.039605	-5.880696
53	1	0	4.447774	-1.386992	-3.640421
54	1	0	3.160774	12.553971	1.208678
55	1	0	4.446971	-3.115182	5.554517
56	1	0	4.113593	-6.32327	4.568684
57	1	0	6.199917	-4.265794	2.904042
58	1	0	12.435849	-3.814644	-5.615874

**13a**000003_en_		Standard Orientation (A.U.)			
Center number	Atomic number	Atomic Type	X	Y	Z
0	7	0	-1.930477	-2.494647	-0.646821
1	6	0	0.195434	-1.053731	-0.880296
2	6	0	1.85165	-1.179628	1.516181
3	7	0	2.288705	-3.747226	2.277264
4	6	0	0.268567	-5.403397	2.220241
5	6	0	-2.206775	-4.133288	1.479499
6	6	0	-4.326707	-5.950441	0.733248
7	6	0	-5.733883	-4.545291	-1.334363
8	6	0	-4.242909	-2.249925	-2.141517
9	6	0	-8.047522	-5.175615	-2.108677
10	8	0	-9.541454	-3.979783	-3.844252
11	6	0	-9.430989	-1.437182	-4.318235
12	6	0	-7.980475	0.369735	-3.309894
13	6	0	-5.823542	0.136826	-1.485079
14	8	0	0.377756	-7.622072	2.833598

15	8	0	0.701	0.23723	-2.723174
16	6	0	-2.359905	5.971154	0.037393
17	6	0	-0.489959	5.914401	-1.843442
18	6	0	1.409553	7.723815	-1.878014
19	6	0	1.449623	9.624035	-0.036138
20	6	0	-0.416113	9.6999	1.841685
21	6	0	-2.303271	7.877587	1.871986
22	6	0	4.211756	0.498578	1.281246
23	6	0	6.436391	-0.559602	-0.190371
24	6	0	8.824344	-0.596514	0.959686
25	6	0	10.954983	-1.490394	-0.292291
26	6	0	10.727135	-2.387746	-2.765159
27	6	0	8.366948	-2.357196	-3.949099
28	6	0	6.250721	-1.443008	-2.676482
29	8	0	3.355573	11.350074	-0.159474
30	6	0	4.416732	-4.393953	3.924506
31	8	0	-4.236237	2.323886	-1.702264
32	6	0	-4.36541	4.037456	0.224161
33	8	0	-5.951747	3.943228	1.881269
34	1	0	-3.746104	-2.202222	-4.143547
35	8	0	12.86399	-3.262197	-3.921868
36	16	0	-3.029178	-2.267534	4.463114
37	16	0	-0.282121	0.586922	3.944052
38	1	0	-5.518512	-6.465557	2.338937
39	1	0	-3.434019	-7.692544	0.056102
40	1	0	-8.992024	-6.861126	-1.421215
41	1	0	-10.868354	-0.978023	-5.703571
42	1	0	-8.34302	2.271895	-3.984424
43	1	0	-6.450271	0.018043	0.475257
44	1	0	-0.506295	4.415968	-3.234404
45	1	0	2.877006	7.698841	-3.309353
46	1	0	-0.376155	11.176951	3.271886
47	1	0	-3.750916	7.887832	3.322649
48	1	0	3.557491	2.262937	0.420181
49	1	0	4.854278	0.960431	3.189619
50	1	0	9.021659	0.088806	2.88714
51	1	0	12.794651	-1.509947	0.612601
52	1	0	8.17999	-3.037923	-5.881185
53	1	0	4.447934	-1.385567	-3.640616
54	1	0	3.162549	12.553354	1.21005
55	1	0	4.44644	-3.11599	5.554092
56	1	0	4.112267	-6.324169	4.568781
57	1	0	6.199464	-4.267596	2.904105
58	1	0	12.435801	-3.813884	-5.616171

**13a**000004_en_		Standard Orientation (A.U.)			
Center number	Atomic number	Atomic Type	X	Y	Z
0	7	0	-1.928589	-2.497326	-0.648873

1	6	0	0.194658	-1.052683	-0.884428
2	6	0	1.855161	-1.178754	1.509068
3	7	0	2.296402	-3.745699	2.26897
4	6	0	0.277906	-5.404464	2.215128
5	6	0	-2.199459	-4.137834	1.476105
6	6	0	-4.317558	-5.957801	0.73163
7	6	0	-5.729721	-4.553411	-1.333076
8	6	0	-4.242893	-2.255769	-2.141293
9	6	0	-8.043987	-5.186508	-2.103288
10	8	0	-9.543177	-3.99255	-3.835501
11	6	0	-9.434602	-1.450444	-4.312294
12	6	0	-7.985013	0.358428	-3.306175
13	6	0	-5.827008	0.128111	-1.482338
14	8	0	0.390548	-7.622113	2.831112
15	8	0	0.695013	0.242727	-2.72506
16	6	0	-2.373618	5.968373	0.041303
17	6	0	-0.503237	5.915192	-1.839208
18	6	0	1.391997	7.72919	-1.8742
19	6	0	1.4268	9.630448	-0.033396
20	6	0	-0.439486	9.702847	1.843933
21	6	0	-2.322128	7.875848	1.874882
22	6	0	4.212808	0.502343	1.269978
23	6	0	6.43783	-0.554251	-0.20205
24	6	0	6.249257	-1.455046	-2.688232
25	6	0	8.355949	-2.366573	-3.966954
26	6	0	10.72163	-2.379095	-2.790966
27	6	0	10.954161	-1.467123	-0.325006
28	6	0	8.823459	-0.572857	0.939041
29	8	0	3.328803	11.361344	-0.156581
30	6	0	4.425056	-4.38884	3.916055
31	8	0	-4.243543	2.318123	-1.698611
32	6	0	-4.374899	4.030125	0.228607
33	8	0	-5.959743	3.932156	1.886993
34	1	0	-3.747858	-2.20675	-4.143692
35	8	0	12.72336	-3.296757	-4.140143
36	16	0	-3.020141	-2.274224	4.462807
37	16	0	-0.277112	0.584109	3.941735
38	1	0	-5.50631	-6.476068	2.338568
39	1	0	-3.422833	-7.697808	0.051796
40	1	0	-8.984718	-6.873613	-1.414605
41	1	0	-10.872805	-0.993674	-5.697524
42	1	0	-8.34847	2.259634	-3.982859
43	1	0	-6.45264	0.006877	0.478183
44	1	0	-0.515776	4.41636	-3.229811
45	1	0	2.859366	7.707061	-3.305667
46	1	0	-0.403621	11.180966	3.273176
47	1	0	-3.770188	7.88329	3.325145
48	1	0	3.555076	2.263639	0.405256
49	1	0	4.855832	0.969031	3.177141

50	1	0	4.441047	-1.40976	-3.642797
51	1	0	8.202982	-3.064539	-5.889033
52	1	0	12.787065	-1.458009	0.607571
53	1	0	9.029598	0.122136	2.861952
54	1	0	3.128518	12.567637	1.209224
55	1	0	6.207488	-4.256293	2.895933
56	1	0	4.450813	-3.112708	5.547323
57	1	0	4.1251	-6.320693	4.557604
58	1	0	14.235222	-3.183863	-3.110864

**13a**000005_en_		Standard Orientation (A.U.)			
Center number	Atomic number	Atomic Type	X	Y	Z
0	7	0	-3.391898	-0.243473	-0.394316
1	6	0	-1.402756	-1.051924	1.032657
2	6	0	-1.269477	-3.965109	1.173745
3	7	0	-1.772706	-5.118896	-1.245574
4	6	0	-3.837924	-4.277365	-2.597485
5	6	0	-5.18392	-2.109517	-1.266811
6	6	0	-7.068691	-0.62404	-2.868961
7	6	0	-6.844931	2.022591	-1.81732
8	6	0	-4.106509	2.381625	-1.142378
9	6	0	-8.865416	3.514917	-1.604574
10	8	0	-9.107624	5.936225	-0.795905
11	6	0	-7.182394	7.38285	0.124741
12	6	0	-4.804046	6.821129	0.748899
13	6	0	-3.314547	4.417712	0.765809
14	8	0	-4.560975	-5.168115	-4.596653
15	8	0	0.108357	0.278956	2.14907
16	6	0	3.598323	4.865314	0.766133
17	6	0	4.056315	4.946	-1.841794
18	6	0	6.453698	4.465213	-2.791723
19	6	0	8.402358	3.827174	-1.128487
20	6	0	7.980649	3.765294	1.477379
21	6	0	5.585997	4.30213	2.413978
22	6	0	1.192908	-4.804868	2.42592
23	6	0	3.658785	-4.144082	1.109849
24	6	0	3.854306	-2.764525	-1.131443
25	6	0	6.203921	-2.094401	-2.115184
26	6	0	8.415447	-2.772583	-0.847459
27	6	0	8.259235	-4.218751	1.357204
28	6	0	5.90334	-4.885978	2.306047
29	8	0	10.697985	3.216342	-2.175802
30	6	0	-0.700899	-7.583609	-1.884917
31	8	0	-0.758462	5.025546	0.00665
32	6	0	1.033923	5.206217	1.845501
33	8	0	0.549456	5.571498	4.052632
34	1	0	-3.046531	2.824143	-2.871192
35	8	0	10.766944	-2.079423	-1.673433

36	16	0	-6.988604	-3.648511	1.413117
37	16	0	-3.835752	-4.817638	3.591053
38	1	0	-8.97666	-1.405752	-2.789203
39	1	0	-6.439784	-0.737989	-4.842419
40	1	0	-10.706268	2.829166	-2.192562
41	1	0	-7.881992	9.291804	0.377707
42	1	0	-3.730663	8.406521	1.487466
43	1	0	-3.240777	3.644416	2.679034
44	1	0	2.517492	5.376255	-3.122048
45	1	0	6.838183	4.523172	-4.8053
46	1	0	9.504762	3.247572	2.754854
47	1	0	5.2048	4.228838	4.425858
48	1	0	1.209406	-3.900864	4.285666
49	1	0	1.089507	-6.844861	2.756398
50	1	0	2.173456	-2.170625	-2.140112
51	1	0	6.309431	-0.989196	-3.841178
52	1	0	9.987131	-4.763485	2.318021
53	1	0	5.801278	-5.97047	4.049278
54	1	0	11.841303	2.711588	-0.830705
55	1	0	-1.324429	-9.015846	-0.524603
56	1	0	-1.400317	-8.0754	-3.756695
57	1	0	1.358328	-7.482436	-1.908341
58	1	0	10.582645	-0.70831	-2.885831

**13a**000006_en_		Standard Orientation (A.U.)			
Center number	Atomic number	Atomic Type	X	Y	Z
0	7	0	-1.927389	-2.498304	-0.648672
1	6	0	0.195365	-1.052725	-0.884132
2	6	0	1.855629	-1.177981	1.509434
3	7	0	2.297367	-3.744727	2.269975
4	6	0	0.27913	-5.403943	2.216657
5	6	0	-2.198274	-4.138004	1.476833
6	6	0	-4.316236	-5.958415	0.733036
7	6	0	-5.72805	-4.555165	-1.332761
8	6	0	-4.241878	-2.25712	-2.140925
9	6	0	-8.041493	-5.189435	-2.104446
10	8	0	-9.539841	-3.996065	-3.837818
11	6	0	-9.432895	-1.453649	-4.313339
12	6	0	-7.98479	0.355805	-3.306117
13	6	0	-5.826778	0.126332	-1.482132
14	8	0	0.392212	-7.621388	2.833219
15	8	0	0.695284	0.242742	-2.724806
16	6	0	-2.375747	5.968009	0.040931
17	6	0	-0.504858	5.914766	-1.839073
18	6	0	1.38992	7.729246	-1.874077
19	6	0	1.423723	9.631056	-0.033825
20	6	0	-0.443112	9.703525	1.842967
21	6	0	-2.325263	7.876028	1.873964

22	6	0	4.212984	0.503423	1.270094
23	6	0	6.437907	-0.552912	-0.202336
24	6	0	6.249405	-1.453215	-2.688699
25	6	0	8.356135	-2.364543	-3.967481
26	6	0	10.721762	-2.377448	-2.791382
27	6	0	10.954247	-1.465945	-0.325244
28	6	0	8.823508	-0.571854	0.938867
29	8	0	3.325306	11.362401	-0.156976
30	6	0	4.426045	-4.387097	3.917327
31	8	0	-4.244171	2.316872	-1.698495
32	6	0	-4.376372	4.029122	0.22842
33	8	0	-5.96143	3.930987	1.886588
34	1	0	-3.746966	-2.207979	-4.143353
35	8	0	12.723465	-3.295045	-4.140637
36	16	0	-3.019406	-2.27364	4.463179
37	16	0	-0.277184	0.585062	3.941332
38	1	0	-5.505251	-6.475705	2.34009
39	1	0	-3.421472	-7.69882	0.054293
40	1	0	-8.982026	-6.876893	-1.416351
41	1	0	-10.871066	-0.997218	-5.698721
42	1	0	-8.349588	2.25706	-3.981958
43	1	0	-6.452533	0.005211	0.478365
44	1	0	-0.516643	4.415536	-3.22925
45	1	0	2.857692	7.707054	-3.305131
46	1	0	-0.408045	11.182092	3.27177
47	1	0	-3.773688	7.883481	3.323867
48	1	0	3.555037	2.264793	0.405635
49	1	0	4.856253	0.97	3.177182
50	1	0	4.441228	-1.407621	-3.643336
51	1	0	8.203244	-3.062088	-5.889723
52	1	0	12.787119	-1.457	0.607403
53	1	0	9.029631	0.122869	2.861876
54	1	0	3.124318	12.569057	1.208403
55	1	0	6.208531	-4.254419	2.897305
56	1	0	4.451446	-3.110707	5.548394
57	1	0	4.126422	-6.318888	4.559221
58	1	0	14.235272	-3.182545	-3.111237

Table S23. Gibbs free energies[a] and equilibrium populations[b] of low-energy conformers of 13b.

Conformers	ΔG(a.u.)	P(%)/100	G(a.u.)
**13b**000001.log	0.00281	4.14	-2436.234366
**13b**000002.log	0.00757	0.03	-2436.22961
**13b**000003.log	0.00319	2.79	-2436.233994
**13b**000004.log	0.0	81.65	-2436.237181
**13b**000005.log	0.00186	11.36	-2436.235319
**13b**000006.log	0.00757	0.03	-2436.229614

[a]wB97M-V/def2-TZVP, in a.u.
[b]From ΔG values at 298.15 K.

Table S24. Cartesian coordinates for the low-energy reoptimized random research conformers of 13b at B3LYP-D3(BJ)/6-31G* level of theory in methanol.

**13b**000001_en_		Standard Orientation (A.U.)			
Center number	Atomic number	Atomic Type	X	Y	Z
0	7	0	-3.539229	-0.082299	-0.224763
1	6	0	-1.680596	-1.28053	-1.572803
2	6	0	-1.478034	-4.106662	-0.879719
3	7	0	-2.046378	-4.630312	1.742628
4	6	0	-4.140598	-3.493738	2.80586
5	6	0	-5.402656	-1.672526	0.987174
6	6	0	-7.310538	0.162431	2.117182
7	6	0	-7.064721	2.458981	0.438335
8	6	0	-4.497532	2.461271	-0.803389
9	6	0	-8.785923	4.285231	0.256534
10	8	0	-8.539736	6.532942	-0.995038
11	6	0	-6.287251	7.804866	-1.14697
12	6	0	-3.942225	7.126176	-0.489272
13	6	0	-2.920493	4.616113	0.359466
14	8	0	-4.944287	-3.9169	4.922964
15	8	0	-0.408844	-0.325706	-3.232625
16	6	0	4.027179	4.331718	0.164964
17	6	0	4.343956	3.04129	-2.124497
18	6	0	6.739701	2.270335	-2.868754
19	6	0	8.813875	2.768899	-1.318647
20	6	0	8.536309	4.129213	0.9274
21	6	0	6.140396	4.893491	1.663302
22	6	0	1.045267	-5.173712	-1.789448
23	6	0	3.415569	-4.078678	-0.58503
24	6	0	5.730003	-4.56844	-1.771681
25	6	0	7.999485	-3.701549	-0.76503
26	6	0	7.99727	-2.294249	1.474138
27	6	0	5.70915	-1.787121	2.678469
28	6	0	3.450425	-2.664877	1.650998
29	8	0	11.18127	1.835022	-1.859279
30	6	0	-1.088022	-6.931884	2.938068
31	8	0	-0.3637	4.37451	-0.517367
32	6	0	1.497438	4.99738	1.145047
33	8	0	1.080219	5.946784	3.196762
34	1	0	-4.571278	2.717151	-2.853333
35	8	0	10.173023	-1.375031	2.526789
36	16	0	-7.168512	-3.745484	-1.362653
37	16	0	-4.00602	-5.615643	-2.997012
38	1	0	-6.722176	0.556967	4.066563
39	1	0	-9.218967	-0.620973	2.20881
40	1	0	-10.645376	4.132543	1.108714
41	1	0	-6.659138	9.681157	-1.881427
42	1	0	-2.522279	8.587794	-0.717152

43	1	0	-2.897799	4.430952	2.416712
44	1	0	2.709155	2.559857	-3.253161
45	1	0	6.978936	1.178037	-4.592414
46	1	0	10.180628	4.478765	2.098082
47	1	0	5.856446	5.891927	3.43058
48	1	0	1.011179	-7.230069	-1.550791
49	1	0	1.120551	-4.807686	-3.820211
50	1	0	5.75813	-5.654658	-3.516647
51	1	0	9.780034	-4.117248	-1.70331
52	1	0	5.717376	-0.643933	4.378527
53	1	0	1.698171	-2.198286	2.604913
54	1	0	11.127824	1.036152	-3.509519
55	1	0	0.971271	-6.89391	3.027475
56	1	0	-1.865572	-6.985783	4.842252
57	1	0	-1.724892	-8.601709	1.891195
58	1	0	11.301363	-0.864264	1.165892

**13b**000002_en_		Standard Orientation (A.U.)			
Center number	Atomic number	Atomic Type	X	Y	Z
0	7	0	-3.68923	-0.154463	-0.451571
1	6	0	-1.713208	-1.156295	-1.770983
2	6	0	-1.179868	-3.912872	-0.989835
3	7	0	-1.424285	-4.257652	1.701004
4	6	0	-3.498186	-3.228816	2.922259
5	6	0	-5.189212	-1.772366	1.12698
6	6	0	-7.17563	-0.040127	2.293423
7	6	0	-7.34379	2.097656	0.404191
8	6	0	-4.845151	2.298758	-0.944752
9	6	0	-9.205606	3.778833	0.226591
10	8	0	-9.176752	5.946934	-1.200006
11	6	0	-7.038959	7.37676	-1.521477
12	6	0	-4.639419	6.957535	-0.833441
13	6	0	-3.508496	4.6116	0.282452
14	8	0	-3.992791	-3.522959	5.151722
15	8	0	-0.579752	-0.031062	-3.435499
16	6	0	3.349183	3.875906	1.159527
17	6	0	4.29086	5.240518	-0.900278
18	6	0	6.884704	5.453054	-1.263473
19	6	0	8.533084	4.26957	0.425783
20	6	0	7.619831	2.975391	2.537291
21	6	0	5.0327	2.772453	2.882735
22	6	0	1.218423	-5.004261	-2.246107
23	6	0	3.807575	-3.983626	-1.545768
24	6	0	4.768777	-1.793151	-2.682327
25	6	0	7.223461	-0.98588	-2.213381
26	6	0	8.768287	-2.301036	-0.520177
27	6	0	7.873702	-4.534848	0.565858
28	6	0	5.438263	-5.367798	0.018079

29	8	0	11.096693	4.168598	0.008318
30	6	0	-0.054053	-6.248619	3.036834
31	8	0	-0.831319	4.691542	-0.169999
32	6	0	0.615156	3.526632	1.60797
33	8	0	-0.261764	2.348835	3.378142
34	1	0	-4.985885	2.600779	-2.981351
35	8	0	11.13535	-1.506708	0.12425
36	16	0	-6.950137	-4.289131	-0.742603
37	16	0	-3.832847	-5.72477	-2.781385
38	1	0	-6.437571	0.616408	4.117052
39	1	0	-8.966689	-0.998615	2.661269
40	1	0	-10.995897	3.575037	1.205049
41	1	0	-7.568759	9.127332	-2.444734
42	1	0	-3.314621	8.466358	-1.248824
43	1	0	-3.756151	4.479147	2.325792
44	1	0	2.988175	6.08918	-2.231945
45	1	0	7.624652	6.46055	-2.895944
46	1	0	8.947508	2.055154	3.797078
47	1	0	4.265865	1.722539	4.465837
48	1	0	1.190575	-7.046606	-1.935807
49	1	0	0.926856	-4.709855	-4.271372
50	1	0	3.57386	-0.717078	-3.946994
51	1	0	7.929698	0.699089	-3.142014
52	1	0	9.101347	-5.594539	1.820911
53	1	0	4.814557	-7.156427	0.808444
54	1	0	11.51795	5.224295	-1.431764
55	1	0	-0.950279	-6.458769	4.877421
56	1	0	-0.187814	-8.033945	2.00028
57	1	0	1.920861	-5.725316	3.289504
58	1	0	11.372244	0.241223	-0.403391

**13b**000003_en_		Standard Orientation (A.U.)			
Center number	Atomic number	Atomic Type	X	Y	Z
0	7	0	-3.520629	-0.140751	-0.213091
1	6	0	-1.642833	-1.262678	-1.601284
2	6	0	-1.313837	-4.08221	-0.920119
3	7	0	-1.815635	-4.631697	1.710862
4	6	0	-3.938689	-3.585708	2.809722
5	6	0	-5.301118	-1.81084	1.017736
6	6	0	-7.265214	-0.057996	2.180561
7	6	0	-7.141847	2.248291	0.500586
8	6	0	-4.596876	2.358176	-0.782367
9	6	0	-8.943078	3.998899	0.351962
10	8	0	-8.821303	6.255386	-0.900628
11	6	0	-6.62778	7.618637	-1.111791
12	6	0	-4.242968	7.043691	-0.499777
13	6	0	-3.097841	4.587323	0.348368
14	8	0	-4.690848	-4.048444	4.937568

15	8	0	-0.440604	-0.253825	-3.279472
16	6	0	3.850971	4.438471	0.105085
17	6	0	4.183416	3.248986	-2.242179
18	6	0	6.57134	2.483725	-3.006324
19	6	0	8.64844	2.882406	-1.425124
20	6	0	8.352822	4.196466	0.860103
21	6	0	5.96001	4.949806	1.624149
22	6	0	1.236629	-5.039626	-1.87429
23	6	0	3.576454	-3.937662	-0.618861
24	6	0	5.935182	-4.572177	-1.648489
25	6	0	8.177875	-3.670071	-0.622217
26	6	0	8.089403	-2.100759	1.49517
27	6	0	5.772244	-1.455067	2.559436
28	6	0	3.5373	-2.353703	1.492179
29	8	0	10.923222	1.910895	-2.145642
30	6	0	-0.747752	-6.898399	2.879079
31	8	0	-0.544183	4.467249	-0.55927
32	6	0	1.320503	5.014641	1.129097
33	8	0	0.901027	5.824432	3.241379
34	1	0	-4.713917	2.602016	-2.831713
35	8	0	10.347997	-1.18277	2.416887
36	16	0	-7.016438	-3.940172	-1.318203
37	16	0	-3.808406	-5.693639	-2.994431
38	1	0	-6.662983	0.358772	4.121052
39	1	0	-9.137835	-0.920027	2.300851
40	1	0	-10.778052	3.763724	1.237763
41	1	0	-7.093516	9.471216	-1.852615
42	1	0	-2.888729	8.558275	-0.777912
43	1	0	-3.039061	4.413973	2.405655
44	1	0	2.548792	2.817618	-3.39077
45	1	0	6.848255	1.454391	-4.756079
46	1	0	10.000612	4.585334	2.024561
47	1	0	5.674545	5.902405	3.41747
48	1	0	1.266737	-7.104151	-1.724423
49	1	0	1.297624	-4.580789	-3.88726
50	1	0	6.016201	-5.785417	-3.304905
51	1	0	9.991463	-4.127021	-1.460471
52	1	0	5.689321	-0.188141	4.172497
53	1	0	1.759905	-1.787409	2.337348
54	1	0	11.792469	1.349453	-0.624712
55	1	0	-1.504649	-7.002042	4.789506
56	1	0	-1.321025	-8.585472	1.823009
57	1	0	1.308565	-6.771595	2.94832
58	1	0	9.977619	-0.116102	3.861517

**13b**000004_en_		Standard Orientation (A.U.)			
Center number	Atomic number	Atomic Type	X	Y	Z
0	7	0	-3.582695	0.007155	-0.279485

1	6	0	-1.804575	-1.303564	-1.625321
2	6	0	-1.754426	-4.126845	-0.885561
3	7	0	-2.307237	-4.557128	1.757233
4	6	0	-4.33253	-3.303702	2.818146
5	6	0	-5.522195	-1.455045	0.975605
6	6	0	-7.311939	0.505304	2.093512
7	6	0	-6.928044	2.771989	0.400191
8	6	0	-4.365299	2.610049	-0.838062
9	6	0	-8.52375	4.707029	0.204709
10	8	0	-8.112343	6.926673	-1.055131
11	6	0	-5.779511	8.051448	-1.16667
12	6	0	-3.492991	7.218426	-0.480409
13	6	0	-2.655437	4.637703	0.360014
14	8	0	-5.130015	-3.64163	4.953228
15	8	0	-0.480793	-0.431399	-3.291269
16	6	0	4.289061	4.197693	0.201544
17	6	0	4.630071	2.749023	-1.990689
18	6	0	7.045742	2.046559	-2.737212
19	6	0	9.144811	2.790301	-1.308292
20	6	0	8.81947	4.249652	0.881106
21	6	0	6.401118	4.938233	1.62303
22	6	0	0.693793	-5.34549	-1.799656
23	6	0	3.11604	-4.418369	-0.562847
24	6	0	5.400198	-5.454297	-1.418365
25	6	0	7.714086	-4.662607	-0.463062
26	6	0	7.78735	-2.795221	1.406302
27	6	0	5.533281	-1.788988	2.327331
28	6	0	3.22814	-2.580749	1.329634
29	8	0	11.549951	2.147871	-1.936765
30	6	0	-1.404109	-6.848101	3.012916
31	8	0	-0.106412	4.218025	-0.480496
32	6	0	1.760512	4.907122	1.146399
33	8	0	1.349143	5.970159	3.144841
34	1	0	-4.413732	2.892695	-2.884943
35	8	0	10.108269	-2.029183	2.237971
36	16	0	-7.420195	-3.456925	-1.322525
37	16	0	-4.382645	-5.538977	-2.938905
38	1	0	-6.698281	0.876603	4.039605
39	1	0	-9.265308	-0.15715	2.193005
40	1	0	-10.393011	4.687048	1.048861
41	1	0	-6.018718	9.952059	-1.893857
42	1	0	-1.979162	8.587994	-0.669668
43	1	0	-2.675858	4.436121	2.415961
44	1	0	2.998846	2.120729	-3.055818
45	1	0	7.305719	0.876773	-4.40633
46	1	0	10.477406	4.830928	1.940309
47	1	0	6.101237	6.052387	3.317787
48	1	0	0.533411	-7.398432	-1.594046
49	1	0	0.800813	-4.949631	-3.82513

50	1	0	5.364693	-6.891744	-2.887377
51	1	0	9.477923	-5.448158	-1.154937
52	1	0	5.572377	-0.325391	3.765685
53	1	0	1.514134	-1.725327	2.054897
54	1	0	11.50876	0.948838	-3.322711
55	1	0	-2.119403	-8.530353	2.038891
56	1	0	0.656412	-6.876387	3.056865
57	1	0	-2.143619	-6.809847	4.932548
58	1	0	9.914711	-0.434968	3.124265

**13b**000005_en_		Standard Orientation (A.U.)			
Center number	Atomic number	Atomic Type	X	Y	Z
0	7	0	-3.569067	0.011727	-0.239901
1	6	0	-1.768037	-1.309462	-1.549899
2	6	0	-1.750077	-4.133015	-0.823112
3	7	0	-2.336855	-4.575947	1.810307
4	6	0	-4.351548	-3.290758	2.862311
5	6	0	-5.515793	-1.436427	1.011954
6	6	0	-7.308398	0.533877	2.104415
7	6	0	-6.93781	2.767381	0.364563
8	6	0	-4.367773	2.597109	-0.858279
9	6	0	-8.547174	4.686096	0.125047
10	8	0	-8.152958	6.880915	-1.183378
11	6	0	-5.827386	8.016835	-1.327948
12	6	0	-3.536246	7.218199	-0.616643
13	6	0	-2.685869	4.669006	0.303632
14	8	0	-5.16317	-3.621385	4.992824
15	8	0	-0.414652	-0.445346	-3.194611
16	6	0	4.256047	4.168957	0.289769
17	6	0	4.640663	2.772166	-1.93037
18	6	0	7.068854	2.062146	-2.629695
19	6	0	9.113597	2.729709	-1.102449
20	6	0	8.756436	4.152848	1.093081
21	6	0	6.328556	4.861764	1.783768
22	6	0	0.687786	-5.379687	-1.733046
23	6	0	3.143127	-4.360369	-0.644215
24	6	0	5.364249	-4.840617	-1.999108
25	6	0	7.709109	-4.054784	-1.112712
26	6	0	7.875598	-2.732483	1.169278
27	6	0	5.685427	-2.229087	2.543881
28	6	0	3.34727	-3.032078	1.635546
29	8	0	11.461208	1.837796	-1.784132
30	6	0	-1.523763	-6.914704	3.040582
31	8	0	-0.123672	4.254978	-0.493149
32	6	0	1.711356	4.89318	1.187559
33	8	0	1.268544	5.930214	3.192207
34	1	0	-4.407729	2.83354	-2.911259
35	8	0	10.139072	-1.883295	2.095191

36	16	0	-7.418899	-3.456325	-1.275716
37	16	0	-4.376662	-5.502798	-2.918085
38	1	0	-6.685582	0.94608	4.039538
39	1	0	-9.258451	-0.134273	2.225619
40	1	0	-10.417533	4.669696	0.966737
41	1	0	-6.078475	9.893265	-2.111683
42	1	0	-2.028575	8.587583	-0.852397
43	1	0	-2.728957	4.523446	2.363501
44	1	0	3.032156	2.193786	-3.055204
45	1	0	7.400056	0.93476	-4.307397
46	1	0	10.361115	4.633202	2.284006
47	1	0	5.99395	5.929674	3.500597
48	1	0	0.544186	-7.417982	-1.399909
49	1	0	0.737545	-5.113612	-3.779958
50	1	0	5.256612	-5.846984	-3.787534
51	1	0	9.411403	-4.436472	-2.197922
52	1	0	5.827194	-1.146158	4.277815
53	1	0	1.666835	-2.564554	2.711649
54	1	0	12.720058	2.669777	-0.740027
55	1	0	0.534419	-7.0169	3.107558
56	1	0	-2.280224	-6.879023	4.953755
57	1	0	-2.285481	-8.5567	2.033846
58	1	0	11.231731	-1.532493	0.659569

**13b**000006_en_		Standard Orientation (A.U.)			
Center number	Atomic number	Atomic Type	X	Y	Z
0	7	0	-3.68847	-0.154637	-0.450582
1	6	0	-1.712765	-1.156481	-1.770679
2	6	0	-1.179515	-3.91318	-0.990053
3	7	0	-1.423559	-4.257987	1.701217
4	6	0	-3.497814	-3.230112	2.922291
5	6	0	-5.188637	-1.773076	1.12731
6	6	0	-7.175104	-0.041104	2.293975
7	6	0	-7.343894	2.096444	0.40467
8	6	0	-4.84535	2.298003	-0.944654
9	6	0	-9.205919	3.777397	0.227418
10	8	0	-9.177984	5.94537	-1.19954
11	6	0	-7.040519	7.37531	-1.52287
12	6	0	-4.640586	6.956979	-0.835719
13	6	0	-3.509317	4.611828	0.281485
14	8	0	-3.992968	-3.525095	5.151574
15	8	0	-0.579686	-0.031165	-3.435328
16	6	0	3.348221	3.876991	1.159698
17	6	0	4.290036	5.241258	-0.900256
18	6	0	6.883902	5.453831	-1.263222
19	6	0	8.53218	4.270527	0.426217
20	6	0	7.618779	2.97665	2.537865
21	6	0	5.031647	2.773772	2.883155

22	6	0	1.219091	-5.004161	-2.246206
23	6	0	3.808049	-3.983395	-1.545797
24	6	0	4.76885	-1.792404	-2.681733
25	6	0	7.223569	-0.985199	-2.213062
26	6	0	8.768738	-2.30081	-0.520505
27	6	0	7.874614	-4.53509	0.56486
28	6	0	5.439122	-5.368052	0.01718
29	8	0	11.095753	4.169421	0.008783
30	6	0	-0.051829	-6.247787	3.037099
31	8	0	-0.832151	4.691936	-0.170558
32	6	0	0.614151	3.528022	1.608154
33	8	0	-0.262921	2.351556	3.379113
34	1	0	-4.98652	2.598899	-2.981383
35	8	0	11.135946	-1.50648	0.12365
36	16	0	-6.949647	-4.288862	-0.743604
37	16	0	-3.831304	-5.725567	-2.780537
38	1	0	-6.436691	0.615662	4.117399
39	1	0	-8.96591	-0.999872	2.662342
40	1	0	-10.995948	3.573562	1.206321
41	1	0	-7.571226	9.125211	-2.44688
42	1	0	-3.316093	8.465503	-1.252997
43	1	0	-3.757304	4.480441	2.324841
44	1	0	2.987478	6.089734	-2.232145
45	1	0	7.623932	6.461007	-2.895857
46	1	0	8.946393	2.056409	3.797718
47	1	0	4.264713	1.724236	4.466456
48	1	0	1.191347	-7.046549	-1.936076
49	1	0	0.927559	-4.709722	-4.271501
50	1	0	3.573611	-0.716139	-3.945934
51	1	0	7.929674	0.699959	-3.141426
52	1	0	9.102592	-5.595261	1.819153
53	1	0	4.815805	-7.157179	0.80674
54	1	0	11.517063	5.224846	-1.431482
55	1	0	-0.946716	-6.457086	4.878438
56	1	0	-0.185868	-8.033837	2.001784
57	1	0	1.923138	-5.72379	3.287896
58	1	0	11.372277	0.241891	-0.402874
